# Impact of the COVID-19 Pandemic on Cancer Researchers in 2020: A Qualitative Study of Events to Inform Mitigation Strategies

**DOI:** 10.3389/fpubh.2021.741223

**Published:** 2021-11-24

**Authors:** Louis Fox, Katharina Beyer, Elke Rammant, Esme Morcom, Mieke Van Hemelrijck, Richard Sullivan, Verna Vanderpuye, Dorothy Lombe, Audrey Tieko Tsunoda, Tezer Kutluk, Nirmala Bhoo-Pathy, Shanmugham C. Pramesh, Aasim Yusuf, Christopher M. Booth, Omar Shamieh, Sabine Siesling, Deborah Mukherji

**Affiliations:** ^1^School of Cancer and Pharmaceutical Sciences, King's College London, London, United Kingdom; ^2^Department of Human Structure and Repair, Ghent University, Ghent, Belgium; ^3^National Centre for Radiotherapy, Oncology and Nuclear Medicine, Korle Bu Teaching Hospital, Accra, Ghana; ^4^Cancer Diseases Hospital & Research Centre, Lahore, Zambia; ^5^Hospital Erasto Gaertner, PPGTS / Pontifícia Universidade Católica do Paraná, Curitiba, Brazil; ^6^Department of Pediatric Oncology, Hacettepe University Faculty of Medicine, Ankara, Turkey; ^7^Centre for Epidemiology and Evidence-Based Practice, Faculty of Medicine, Universiti Malaya, Kuala Lumpur, Malaysia; ^8^Tata Memorial Centre, Homi Bhabha National Institute, Mumbai, India; ^9^Shaukat Khanum Memorial Cancer Hospital and Research Centre, Lahore, Pakistan; ^10^Department of Oncology, Queen's University, Kingston, ON, Canada; ^11^Department of Palliative Medicine, King Hussein Cancer Center, Amman, Jordan; ^12^Department of Research and Development, Netherlands Comprehensive Cancer Organisation (IKNL), Utrecht, Netherlands; ^13^Department of Health Technology and Services Research, Technical Medical Centre, University of Twente, Enschede, Netherlands; ^14^American University of Beirut Medical Center, Beirut, Lebanon

**Keywords:** cancer, COVID-19, impact, funding, mitigation, epidemic, pandemic, oncology

## Abstract

**Introduction:** The COVID-19 pandemic has had an unprecedented impact on global health systems and economies. With ongoing and future challenges posed to the field due to the pandemic, re-examining research priorities has emerged as a concern. As part of a wider project aiming to examine research priorities, here we aimed to qualitatively examine the documented impacts of the COVID-19 pandemic on cancer researchers.

**Materials and Methods:** We conducted a literature review with the aim of identifying non-peer-reviewed journalistic sources and institutional blog posts which qualitatively documented the effects of the COVID-19 pandemic on cancer researchers. We searched on 12th January 2021 using the LexisNexis database and Google, using terms and filters to identify English-language media reports and blogs, containing references to both COVID-19 and cancer research. The targeted search returned 751 results, of which 215 articles met the inclusion criteria. These 215 articles were subjected to a conventional qualitative content analysis, to document the impacts of the pandemic on the field of cancer research.

**Results:** Our analysis yielded a high plurality of qualitatively documented impacts, from which seven categories of direct impacts emerged: (1) COVID measures halting cancer research activity entirely; (2) COVID measures limiting cancer research activity; (3) forced adaptation of research protocols; (4) impacts on cancer diagnosis, cases, and services; (5) availability of resources for cancer research; (6) disruption to the private sector; and (7) disruption to supply chains. Three categories of consequences from these impacts also emerged: (1) potential changes to future research practice; (2) delays to the progression of the field; and (3) potential new areas of research interest.

**Discussion:** The COVID-19 pandemic had extensive practical and economic effects on the field of cancer research in 2020 that were highly plural in nature. Appraisal of cancer research strategies in a post-COVID world should acknowledge the potential for substantial limitations (such as on financial resources, limited access to patients for research, decreased patient access to cancer care, staffing issues, administrative delays, or supply chain issues), exacerbated cancer disparities, advances in digital health, and new areas of research related to the intersection of cancer and COVID-19.

## Introduction

On 11th March 2020, the emergence and prolific spread of the novel and potentially lethal coronavirus SARS-CoV-2 led the World Health Organization to declare a pandemic (1). The virus and its resultant disease, Coronavirus Disease 2019 (COVID-19), spread globally and has since had dramatic and widespread effects. Globally, as of May 2021, total registered cases of COVID-19 are nearing 170 million, and more than 3.5 million deaths have been registered as attributable to the disease (2).

For many countries, the COVID-19 pandemic has impacted virtually every aspect of life, due to disruptive non-pharmaceutical interventions that have been deployed in efforts to control the spread of the virus, such as “stay at home” orders and the widespread closure of businesses deemed non-essential (3). Patients were reluctant in visiting their GP in case of complaints and screening programs for cancer were temporarily halted, leading to a decrease in cancer diagnosis. Furthermore, healthcare systems have been strained to their limits supporting large volumes of people presenting with COVID-19, and this has at times seriously impacted the provision of regular non-COVID-19 care to their patients, including cancer treatments (4). Economic activity in many countries has at times all but ceased due to the attempts to control the pandemic, precipitating substantial increases in unemployment and raising the specter of a longer-term economic downturn (5).

As countries adjust to the disruption wreaked by the COVID-19 pandemic, it is vital that the progress of cancer research be protected and strengthened as far as possible under the circumstances. Hence, as part of the COVID-19 and Cancer Global Task Force (https://covidcancertaskforce.org/) we initiated a project, named “REdefining cancer research PRIoritieS in the Emerging context of the COVID-19 pandemic” (REPRISE). The project aims to examine priorities for cancer research, to ensure that future research strategies in cancer yield the most value for patients, given the potential for funding squeezes.

In order to reach an informed consensus about research priorities, there is a need to understand the present situation in terms of: (i) where research priorities have historically been placed; and (ii) the impact of the COVID-19 pandemic on the cancer research field. Hence, the REPRISE project consists of three sub-projects that will feed information into a final consensus-building exercise. The sub-projects consist of: (1) a global snapshot survey of cancer researchers to examine the impacts that they may personally have experienced (6); (2) a bibliometric analysis to examine how cancer research resources have historically been allocated around the world (and how this might have been impacted by the COVID-19 pandemic) (7); and (3) a qualitative analysis and documentation of the events of 2020, focusing on the ways in which cancer researchers were impacted by the COVID-19 pandemic. Findings from the latter sub-project, the qualitative analysis, are presented in this article.

For the qualitative analysis, we reasoned peer-reviewed studies would not be the most effective method of documenting the landscape of the events of 2020, as peer-reviewed studies have a specific scope and research question(s), tend to lack narrative detail about events, and introduce a large time lag between data collection and publication. Hence, we decided to primarily collate media articles and blog posts, which we reasoned would provide a more up-to-date and comprehensive documentation of the impacts of the COVID-19 pandemic on cancer research. The method is analogous to a typical qualitative study. A strength of this strategy is that we were able to collate and analyse data that was produced by many efforts within a large social infrastructure comprised of reporters, researchers, and industry professionals. Using a targeted search strategy, this approach enabled an informative cross-section of sources encompassing popular news media, medical news media, blog posts from cancer research institutions and charities, and comment pieces/editorials in specialist medical or cancer publications, such as *the Lancet Oncology* and *Nature*. The events documented in these types of sources primarily convey the experiences of professionals working in the cancer research field. The scope of our analysis was hence limited to the events that unfolded within the professional environment, as opposed to investigating the direct impacts on patients with cancer. The study reported herein aimed to use this approach to qualitatively document the impacts of the COVID-19 pandemic on cancer researchers in 2020.

## Methods

### Search Strategy

To collate data on the events of 2020, we searched the Lexis Nexis database on 12th December 2020, using the following search terms, incorporating all terms recommended by Lexis Nexis staff for searching for COVID-19-related material:

(covid or “covid-19” or covid19 or coronavirus or “SARS-CoV-2” or “corona virus”) and [(cancer or oncology or oncological)/1 (research or studies or study)].

The search was filtered to only return records classified as “blog posts”, as examination of the search results showed that this filter function retained the type of media we were seeking (e.g. health-based news articles), whilst keeping the number of results at manageable levels. Further filters were used to narrow the results to articles in English, and articles under the topic of “Medicine and Health”.

We also used the Google search engine (without using a Google account), to supplement the above search, on 12th January 2021. The Google search used the terms:

(covid OR covid19 OR covid-19 OR coronavirus OR “corona virus” OR “SARS-CoV-2” OR “SARS-CoV2”) AND (“cancer research” OR “cancer studies” OR “oncology research” OR “oncology studies” OR “oncological research” OR “oncological studies”) AND (blog OR news).

### Screening and Inclusion Criteria

The article body text of all articles was screened by two independent reviewers (LF, with either KB, ER, or EM as second reviewer). Disagreements on inclusion were initially resolved by discussion, with persistent disagreement resolved by the inclusion of a third reviewer (one of the above reviewers not included in the original decision). The inclusion criteria were defined as:

“*Any news article, blog post, or scientific journal commentary/editorial which documents disruption to (or continuity of) the regular conduct of cancer research or the provision of cancer care caused by (or in spite of) the SARS-CoV-2 pandemic* **OR** *any news article, blog post, or scientific journal commentary/editorial which reports, documents, or communicates funding shortfalls experienced by cancer research funders, that are thought to result, directly or indirectly, from the effects of the SARS-CoV-2 pandemic.”*

Articles were excluded if they were not seen to meet the inclusion criteria.

### Analysis

Included articles were analysed in the NVivo software package using a conventional qualitative content analysis, which is an appropriate method when a researcher aims to simply describe a phenomenon, and when existing literature or theory is limited (8). The approach produces findings directly derived from the text analysed, while allowing for inductive categories to emerge. The analysis aimed to address the following research question:

“*What were the impacts of the COVID-19 pandemic on the field of cancer research, from March 2020 to January 2021?”*

**Figure 1:**
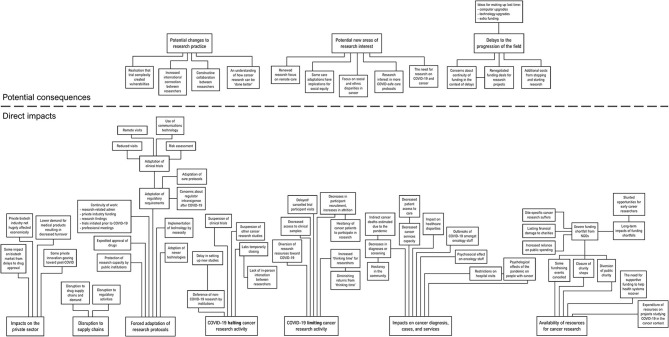
A conceptual mind map of the categories produced by the content analysis, with their subthemes.

Articles were deductively coded, to identify specific portions of text within them that were relevant to the research question. Individual codes, comprising distinct events or concepts, were derived from the data and given descriptors that precisely fit the data represented (which were modified as appropriate in line with emergent data). Elements of data that consistently described a particular type of event or concept were grouped under individual codes. The individual codes were then horizontally analysed to identify groups of codes that described events with similar implications, in terms of their impact on cancer research. In this process of inductive categorisation, codes were organised into precisely defined categories to describe them, and these categories were refined as the analysis proceeded, to ensure that how they were logically organised was reflective of the data being coded. Some categories were organised into hierarchies, based on logical relationships between different codes inferred from the raw data they represented. The process enabled an integrated description of the events underpinning the broader impacts, and how these events were related to one another (or contrasted with one another). Some codes were merged, or discarded, based on their logical relationship (or lack thereof) to the emergent categories. The emergent categories (and their subordinate subcategories) were arranged into a spatial “mind map”, consistent with the logical relationships inferred from the raw data, to visualise their relationships to one another (see Figure 1). Results are presented below in terms of the overarching emergent categories, with context and detail provided that is consistent with the way in which the categories, and their subordinate subcategories, were deemed to be related to one another.

## Results

The search of Lexis Nexis produced 618 articles. The Google search returned an additional 144 results. Eleven of the records returned were duplicated across the two search platforms and hence these were omitted. Therefore, the final search result consisted of 751 articles in total. The screening process resulted in 517 articles being excluded, with 234 articles being initially included. During analysis, a further 19 articles were found to be duplicate reports of others, and these were excluded. Therefore, 215 unique reports were analysed (see **Appendix** in Supplementary Material).

The articles analysed demonstrated a high plurality of issues caused by the COVID-19 crisis that impacted, or have the potential to impact, cancer research. The impacts that emerged from the analysis can be best understood in terms of (1) *direct impacts* from the COVID-19 crisis, were segregated into seven categories; and (2) *potential consequences* of these impacts, which could be segregated into three categories. The following section will focus first on the direct impacts, before reporting on the potential consequences of these impacts. Although it was clear from the data that certain impacts were highly significant, the analysis did not aim to quantify the frequency or magnitude of relevant events or offer comparisons between them, in terms of impacts on the field. Hence, the following findings can be considered purely qualitative observations to identify areas that are of potential concern.

The direct impacts could be segregated into:

(1) COVID-19 halting cancer research activity.
(2) COVID-19 limiting cancer research activity.
(3) Forced adaptation of research protocols.
(4) Disruption to supply chains.
(5) Impacts on cancer diagnosis, cases, and services.
(6) Availability of resources for cancer research.
(7) Impacts on the private sector.

The potential consequences could be segregated into:

(1) Potential changes to future research practice.
(2) Delays to the progression of the field.
(3) Potential new areas of research interest.

The segregated areas are identified with subheadings below, along with descriptive summaries of the content analysed within and illustrative quotes, with sources and dates in square brackets.

### Direct Impacts

#### COVID-19 Halting Cancer Research Activity

The initial onset of the COVID-19 pandemic in “Western” countries in March 2020 saw the immediate suspension of a large amount of cancer studies. The extent of the disruption was reported in April and early May of 2020, when three surveys of cancer researchers were conducted by the American Cancer Society (ACS), the American Society of Clinical Oncology (ASCO), and a collaboration between The Cancer Research Institute (CRI) and IQVIA, a private company. It was consistently reported that approximately 50–60% of cancer researchers surveyed said that at least one of their research studies had been completely suspended. In some centres “all clinical studies were put on hold during the early days of the COVID-19 outbreak to maximize hospital capacity for COVID-19 cases” *[European Organisation for Research and Treatment of Cancer (EORTC), 30th* *June 2020]*. One researcher reported the motivation behind this decision as the “uncertainty of the effect of experimental medication on the risk of infection and severe sequalae and the fact that they could not ensure compliance to trials due to the outbreak” [*EORTC, 30*th *June 2020]*. In some centres, cancer clinical trials were “cut to ‘almost zero' and [were] allowed only when a participant [was] deemed to have exceptional need” [*Nature, 25th* *Mar 2020]*. Institutions “prioritized enrolment for some trials based on patients' needs, safety, and disease severity; the burden enrolment would have on patients and the trial site; and availability of resources” [*Precision Oncology News, 14th* *Sept 2020]*. Private pharmaceutical firms took “proactive steps to minimise further stress on healthcare systems including delaying initiation of new studies and pausing enrolment of new patients” *[Living Beyond Breast Cancer, 1st* *May 2020]*.

The suspension of many clinical trials was accompanied by widespread, immediate lab closures. For instance, it was reported that a glioma researcher in California “had less than 12 hours to shut down her lab when California put shelter-in-place orders in effect on March 16” *[Alex's Lemonade Stand Foundation (ALSF), 25th* *Sept 2020]*. The extra work involved in having to stop and re-start labs, and the disruption to the flow of translational research, was described at the time by one researcher as “a three-month shutdown, that will lead to a six-month lag” *[ALSF, 25th* *Sept 2020]*. When labs re-opened, the impacts of COVID-19 restrictions on “[the ability] to enrol patients on clinical trials, access to clinical samples or interaction in person with colleagues” were cited as continuing barriers to productivity amongst researchers *[PharmaTimes Online, 30th* *Nov 2020]*.

Furthermore, many research governance entities, who are key to the setup of new cancer studies, made the decision to defer non-COVID-19 research projects, and to prioritise COVID-19-related research. It appears that these decisions created a bottleneck, as researchers were continuing to produce ideas but could not investigate them: “We're thinking about new trials, but we can't start them … It's like designing a car but not being able to build it – only a million times more important” *[CU Anschutz Medical Campus, University of Colorado, 16th* *July 2020]*.

#### COVID-19 Limiting Cancer Research Activity

In addition to the top-down cessation of research activities, there was also a conscious effort by many cancer researchers to focus their energies on SARS-CoV-2. As one UK researcher put it: “there has been a significant reduction in clinical trial capacity and activity as hospitals, cancer researchers and research infrastructure have refocused on COVID-19” *[Pharmafile, 14th* *Sept 2020]*. The articles analysed contained many reports of cancer researchers diverting their focus away from cancer research, for example:

“[Name] is a PhD student who normally works on lung cancer research, but since the pandemic, has returned to the frontline as a doctor.” *[Cancer Research UK, 1st* *June 2020]*.

“Other examples [of NCI initiatives] include … pivoting some cancer research activities to COVID-19/SARS-CoV-2 research.” *[National Cancer Institute (NCI), 14th* *April 2020]*.

“When the COVID-19 pandemic started, researchers at the Case Comprehensive Cancer Center immediately created plans to shift activities toward combating this deadly disease.” *[Case Western Reserve University, 29th* *June 2020]*.

Some researchers who were not occupied with SARS-CoV-2 reported that the shutdown of labs had produced some much needed “thinking time” that they would otherwise not have had. Two researchers said: “We can rethink things, analyze data every which way to see if it's telling us things we hadn't seen before … Having the time to think about your projects – that downtime can really be valuable. It can give you the headspace to think about something in a new way and re-evaluate your approach … My lab is getting way more thinking time … they're reading all the papers they never had time for when they were focused on experimental work.” But another researcher stated that this opportunity was only useful for a few weeks: “Now we're six weeks in and at this point, we're getting diminishing returns from taking time off to think, and it's time to get back to doing. People are itching to get back into the lab.” *[CU Anschutz Medical Campus, University of Colorado, 16th* *July 2020]*.

COVID-19 restrictions reportedly also affected the confidence of potential research participants to take part in on-site clinical research, as well as the confidence of clinicians to enrol potential participants:

“In March, [the interviewee] received a prognosis of six to 12 months. Still receiving chemo, she wasn't looking to enrol in a trial at the height of the pandemic.” *[NBC News, 21st* *Oct 2020]*.

“Although cancer patients are used to accepting a high level of risk in their care, the added fear of a potentially deadly viral infection likely made patients more cautious. ‘Patients weren't eager to get into planes, trains, and automobiles to get access to their studies' said [researcher].” *[Precision Oncology News, 14th* *Sept 2020]*.

“In the midst of a pandemic, standard risk benefit ratios have shifted, as physicians and patients must now also consider the potential increase in risk of contagion associated with the hospital and lab visits needed for assessments. Without a guaranteed return of therapeutic benefit, this risk may outweigh the benefits for some prospective clinical trial participants.” *[Aptitude Health, 1st* *December 2020]*.

In addition, participants already enrolled on trials experienced significant disruptions to study protocols. One of the previously mentioned surveys found that “nearly 60 percent of the investigators … reported that the pandemic had a moderate or a high impact in delaying or cancelling patient visits to these trials.” The source quotes two figures in the field, who say that this was “a significant disruption in terms of collecting crucial patient data,” and that “it will be very difficult to measure the impact these disruptions will have on the advancement of medicine and on the lives of patients in the months and years ahead” *[NBC News, 21st* *October 2020]*.

#### Forced Adaptation of Research Protocols

Many cancer research studies continued operating throughout the initial onset of the pandemic (or were re-started) subject to additional safety considerations and protocols. It was reported by the NCI that “accrual to certain trials has continued — for example, those offering life-saving therapies or those for patients who have no other options for therapy” *[NCI, 17th* *April 2020]*. It seems that many clinicians and researchers were faced with difficult decisions during this time, trying to “find the balance between treating patients for their cancers and ensuring that the patients were safe while traveling … [with] limited information to guide their decisions” *[NCI, 29th* *June 2020]*. Guidance on conducting phase I cancer clinical trials during the pandemic was not proposed until August 2020, when investigators had accumulated enough experience to be able to make recommendations *[European Society for Medical Oncology (ESMO), 5th* *August 2020]*. The extended period of uncertainty made the need for swift adaption necessary.

Adaptations were facilitated by some flexibility that was introduced to regulatory structures. In March 2020, the NCI issued guidance, giving trial sites “flexibility in the operations of trials, including in the timing of when patient tests and assessments must be done” *[NCI, 29th* *June 2020]*. Furthermore, “patients were able to provide consent remotely, utilise telemedicine for appointments, and receive oral medications and other treatments at home. For procedures that required in-person visits, such as imaging or blood tests, patients were able to visit local facilities rather than having to travel to distant research facilities” *[American Association for Cancer Research (AACR), 11th* *Jan 2021]*. Private pharmaceutical firms provided support for their studies to continue to recruit participants safely. For instance, one biotechnology company created “a COVID-19 task force to ensure that appropriate medical centres hosting the trials are implementing safety measures so that testing could continue,” and subsequently reported achieving recruitment targets despite the pandemic *[The Jerusalem Post, 25th* *May 2020]*.

It is reported that the flexibilities around remote protocols were greatly utilised by clinical researchers, who pursued “emergency amendments enabling virtual evaluations, local monitoring, and at-home treatment [enabling] some research to continue despite lockdowns and safety measures limiting in-person care” *[Precision Oncology News, 24th* *Sept 2020]*. For example, at one US hospital, “instead of having patients come to the hospital for routine assessments, nurses went to the patients … teams of nurses, carrying backpacks with medical supplies and personal protective equipment, met with the children and provided basic care, such as collecting blood samples … the nurses essentially brought the clinical trials to the patients” *[NCI, 29th* *June 2020]*. Such adaptations extended to the adoption of existing technologies. For example, it was reported that “laboratories have been implementing digital pathology to maintain operations during the COVID-19 pandemic” *[HIT Consultant Media, 1st* *Dec 2020]*.

#### Disruption to Supply Chains

Pharmaceutical supply chains were disrupted by the onset of the pandemic. It was reported by a market research firm that “the impact of the [COVID-19] health crisis has been massive on the Cancer Immunotherapy sector with disruption in the supply chains” *[PR Newswire, 16th* *Oct 2020]*. It was also reported that regulatory approval for a B-cell lymphoma treatment in the US would be indefinitely postponed “due to the agency's inability to inspect a third-party manufacturing facility in Texas during the current review cycle due to travel restrictions related to the COVID-19 pandemic” *[Benzinga, 17th* *Nov 2020]*. Such delays may have had downstream effects on the delivery of cancer drug trials.

#### Impacts on Cancer Diagnosis, Cases, and Services

Cancer research priorities may be affected by the fact that the onset of the COVID-19 pandemic, and resulting non-pharmaceutical interventions introduced to control it, resulted in widespread deficits in healthcare as a result of decreased access to cancer care, and decreased rates of early cancer detection. For example, it was reported that a survey in the UK found that “69% of oncology staff believe patients' access to ‘standard of care treatment' … has been compromised as a result of the COVID-19 pandemic” *[National Cancer Research Institute (NCRI), 29th* *Oct 2020]*. Patients reported similar experiences, with one US survey of breast cancer patients showing that “30% of respondents reported delays in hospital- or clinic-based cancer therapies, including radiation, infusion therapies, and surgical tumour removal” *[University of Illinois, 21st* *Aug 2020]*. Similar observations of approximately one-third of patients experiencing treatment delays were reported in the Netherlands *[medwire News, 27th* *Nov 2020]*. It was reported by Cancer Research UK that in the first 18 weeks of the “lockdown” in the UK, an estimated 38,000 fewer cancer treatments took place than normally would *[Cancer Research UK, 28th* *July 2020]*. It was suggested by Cancer Research UK that in addition to less patients presenting, “staff needing to isolate or shield could also have contributed toward [reductions in care capacity]” *[Cancer Research UK, 18th* *Sept 2020]*. Direct impacts of these disruptions on research have been documented. For example, a blog post from May 2020 tells how one research team “typically recruits patients to their studies soon after surgery for early-stage breast cancer, but these processes have been disrupted under current best practices during the COVID-19 pandemic. The research team does not expect to begin recruiting study participants until the pandemic threat has passed” *[University of Delaware, 13th* *May 2020]*.

As has been well documented, the articles analysed here also described the dramatic reductions in referrals from early detection services and suspected cancer referrals from primary care. In the UK, bowel, breast, and cervical cancer screening programmes were suspended upon the onset of the epidemic there *[Cancer Research UK, 23rd* *June 2020]*. In the US at the initial onset of the epidemic, the American Cancer Society recommended that the public postpone their screening appointments *[DC Medical Malpractice and Patient Safety Blog, 21st* *Apr 2020]*. Although the programmes were eventually restarted, it became apparent in late 2020 that referrals for suspected cancer were not catching up with those that were “lost”, creating a missing cohort of individuals that are eventually expected to present *[RT Magazine, 12th* *Oct 2020]*. The problem persisted into January 2021, with one report from the AACR noting that “screening rates are still far below pre-pandemic levels” *[AACR, 11th* *Jan 2021]*. The situation appears to be ongoing and could in future present an unmet research need.

#### Availability of Resources for Cancer Research

Delays caused by the stoppage of clinical studies and closure of labs prompted immediate concerns about active research grants and funding, expressed by one researcher at the time as “wide, sweeping effects … I don't think we know what the impact will be, but I think everyone is bracing for a difficult time” *[Cancer Discovery, 1st* *June 2020]*. Some funder institutions, such as NCI, responded by “providing flexibility on how money is spent, allowing investigators to use grants to pay salaries and stipends or to cover unanticipated research costs” *[Cancer Discovery, 1st* *June 2020]*.

A potentially longer-term problem regarding funding from non-governmental organisations (NGOs) was materialising, however. Large charity organisations that fund cancer research were having to close their retail stores and cancel fundraisers due to the need for social distancing, and the dramatic economic fallout from the impact of the pandemic led to fewer donations from the public. It was observed that “the pandemic [was] a ‘perfect storm' … an increase in demand for services at the same time as volunteers are forced to stay at home and donations are drying up” *[The Week, 8th* *Apr 2020]*. The impact of these events on charities' economic circumstances was severe. By July, Cancer Research UK – the largest independent funder of cancer research in the world – had announced cuts to research spending of £194 million over four years, and 345 redundancies in 2020 (around a quarter of its workforce). This was due to internal forecasts of a £300 million decrease in income in the period 2020–2022 *[Science | Business, 16th* *July 2020]*. The Canadian Cancer Society predicted that “the pandemic [would] cost them CA$100 million in lost donations during the ongoing financial year, which amounts to more than half their budget.” The American Cancer Society (ACS) saw “a decrease in revenue of around US$200 million … [and] cut its expenditure on new research from $100 million to $50 million” *[The Lancet, 17th* *Dec 2020]*, and consequently was unable to accept any applications for the autumn 2020 grant cycle *[Dana-Farber Cancer Institute, 8th* *Oct 2020]*.

Financial support provided by the UK government appeared unable to adequately ameliorate the problem of charity funding: a £750 million “bail out” package offered early on to support the entire charity sector was described by one non-cancer charity chief executive as “a sticking plaster for a critical wound” *[The Week, 9th* *Apr 2020]*. The Association of Medical Research Charities, a body representing 160 charities that between them put £1.9 billion into R&D in 2019, estimated that, “without government support, it [would] take four and a half years for spending to return to the 2019 level” *[Science | Business, 16th* *July 2020]*. NGOs were forced to make such decisions due to the realisation that the pandemic was going to precipitate a global economic downturn, as noted by Cancer Research UK's chief executive: “We're living through a global crisis unlike any other and, as it's unfolded, it's become clear that there'll be a huge economic impact for years to come” *[Science | Business, 16th* *July 2020]*. In the autumn of 2020, it was apparent that the UK had seen “overall public investment in cancer research drop by 24%” *[NCRI, 24th* *Sept 2020]*, and American Cancer Society staff were quoted as saying that “we are possibly going to lose a decade of research because of COVID” *[ABC 15 News, 1st* *Oct 2020]*. The assessment was shared by Cancer Research UK's chief clinician, who stated that the losses amounted to “the equivalent of 10 years' worth of clinical trials going unfunded” *[PharmaTimes Online, 24th* *June 2020]*. Furthermore, it was forecast that “the greatest impact [would] be seen on research focused on specific cancer types, as a large proportion of site-specific cancer research funding comes from charities (70%)” *[NCRI, 24th* *Sept 2020]*.

On top of this, the emergence of a novel and widespread infectious disease meant that resources that may have initially been earmarked for cancer research were allocated toward projects that studied the intersection between COVID-19 and cancer. For example, this analysis found reports of research studies of COVID-19 in cancer patients that had been funded by Cancer Research UK *[PMLive, 5th* *Jan 2021]*, Yorkshire Cancer Research *[Yorkshire Cancer Research, 15th* *Apr 2020]*, the National Cancer Institute, Aim Immunotech Inc., and Roswell Park Comprehensive Cancer Center *[Proactive Investors, 9th* *July 2020]*.

#### Impacts on the Private Sector

Some articles alluded to impacts of the pandemic on the biotechnology sector. It appears in the articles that the private biotechnology sector was also economically affected by the pandemic, with some companies “implement[ing] a review of operations in order to reduce or defer spending where possible, while maintaining key clinical and business program priorities” *[Proactive Investors, 2nd* *Apr 2020]*. It was also reported in November 2020 that “delays [to clinical trials] caused due to pandemic crisis also leads to a backlog of approvals, and eventually delays the product launches” *[Fact.MR, 24th* *Nov 2020]*. The impact on the sector would however appear to be mixed. For example, in December 2020 it was reported that the biotech industry's overall share price had risen by 16%, and one company was reported as exiting the third quarter of 2020 with “better-than-expected earnings and revenues” *[Zachs Equity Research, 8th* *Dec 2020]*. In a sign of confidence, the company in question was reported to have returned their government-funded support package. The positive trend was attributed to “diagnostics revenues … as a result of growing demand for COVID-19 testing … also, there was sequential recovery in base business with people resuming their routine preventive visits, actively caring for their chronic conditions and moving ahead with elective surgeries and other procedures” *[Zachs Equity Research, 8th* *Dec 2020]*.

It seems that the pandemic has also spurred some private innovation with relevance to the cancer research field. For example, it was reported that “in response to the challenges brought on by the pandemic,” one company had developed a product that “offers cancer care teams in-app features such as patient screening, real-time symptom reporting, secure care team messaging, telephone triage workflow automation, and patient access to medical records, thereby helping them with the new short and long-term healthcare changes resulting from the coronavirus pandemic” *[Zachs Equity Research, 22nd* *Oct 2020]*.

### Potential Consequences

#### Potential Changes to Research Practice

The articles analysed showed that there has been an acknowledgement in the cancer research field that some of the adaptations that were forced upon researchers represented improvements in the way that research studies are conducted. An article by representatives from a UK cancer charity summarised this attitude: “COVID-19 stands as a litmus test for pushing the boundaries of standardised research processes. Studies have been expedited, drugs have been made available to patients who might not otherwise participate in trials, and researchers are collaborating with competitors from other labs and companies” *[MedCity News, 27th* *Dec 2020]*. The pandemic exposed some vulnerabilities in the complex way that modern cancer trials have been conducted. As one researcher put it, “the complexity of modern trials means that actually you need all of the [medical] systems to be functioning … it's not just the question of the oncology department being involved.” Some complex trials ceased being able to operate as certain non-oncology hospital departments “were either overwhelmed by coronavirus patients or simply shut down” *[NBC News, 21st* *Oct 2020]*. A clinical trials analyst interviewed for an article observed that “clinical research disruptions involving experimental cancer therapeutics raise severe challenges, as these trials can be highly time-sensitive and even small administrative delays can prove highly detrimental” *[Pharmafile, 14th* *Sept 2020]*.

Repeatedly, researchers have called for efforts to maintain the spirit of scientific co-operation that was triggered by the initial onset of the pandemic. The sentiment was in part driven by a rapid realisation that existing digital platforms can be wielded to foster better accessibility and more international collaboration *[Science, 28th* *Apr 2020]*. As early as July 2020, there was speculation that the crisis might “usher in a new type of scientific collectivism where we more openly share ideas and approaches to rapidly develop effective therapies and diagnostics” *[Cancer Discovery, 15th* *July 2020]*. Such discourse continued unabated throughout 2020, apparently reflecting a genuine desire in the field for change:

“It is hoped that these last 2 months represent not merely lost time but rather a revolutionary inflection point that will increase productivity by stimulating open cooperation between groups, including those in industry, who were formerly competitors or had limited collaborations because of intellectual property considerations.” *[Cancer Discovery, 15th* *July 2020]*.

“The collaboration and partnership … during this time has demonstrated what can be achieved. It is therefore paramount that we continue to work together, identifying what has worked well, and drive further positive change so we can, together, transform cancer treatment and improve outcomes for cancer patients.” *[Pharmafile, 14th* *Sept 2020]*.

“Let's start making this cooperative spirit the rule rather than the exception in science and medicine.” *[MedCity News, 27th* *Dec 2020]*.

Such statements may reflect a sociocultural development in the way that scientific advancement is collectively pursued in the cancer field.

#### Delays to the Progression of the Field

In addition to the immediate delays to research studies documented above, the pandemic is considered likely to result in longer term delays to the scientific progression to the field, due to more systemic factors.

First, there are additional time costs associated with re-starting studies that have been stopped:

“‘It's like ice,' says [researcher], ‘a quick freeze and a slow thaw to carefully and safely reopen the hospital services, basic science and clinical research. It's not easy to restart everything and in some ways getting back up to speed is more complex than the closing. The ramifications of that will be seen much longer than anything else.”' *[CU Anschutz Medical Campus, University of Colorado, 16th* *July 2020]*.

Second, such delays to research programmes are expected to have had an outsized impact on delays to scientific progress more widely. As late as November 2020, researchers at one large UK institute taking part in a survey estimated that their own research advances would “be delayed by six months – and that the wider impact, because of the interconnectedness of science, is likely to push back major advances for patients by nearly a year and a half” *[PharmaTimes Online, 30th* *Nov 2020]*.

Third, concerns have been raised about the career progression of some early career cancer researchers due to the disruption. For instance, one early career researcher who was interviewed expressed their concerns that “there's no guarantee that if you fall beyond that five-year [grant] period that you're actually going to get any teaching money, any grants or support from the university” *[Cal Matters, 26th* *Mar 2020]*. In January 2021, the President of the AACR acknowledged that “the pandemic will continue to be particularly challenging for early-career investigators who are facing a lack of funding and job opportunities in the next year due to budgeting shortfalls at foundations, universities, and research institutions … other pandemic-related challenges include reduced time in the lab to generate data and the inability to travel to job interviews or establish connections at in-person conferences” *[AACR, 11th* *Jan 2021]*. Another piece noted how “new labs are like start-up businesses and finances can be extremely tight … once those labs shut down, young researchers were put in shaky financial situations,” quoting a senior researcher as saying “we are at a real risk of losing an entire generation of cancer researchers” *[ALSF, 25th* *Sept 2020]*. An article in The Lancet in December 2020 highlighted that “those in the early stages of their career are particularly susceptible to budget cuts,” as they are “most dependent on institutional support … if we increase the disincentives to stay in academia, or to enter the field in the first place, we risk losing a lot of talent” *[The Lancet, 17th* *Dec 2020]*. It has also been noted that the closure of labs amidst the onset of the pandemic was “particularly damaging to trainees, many of whom are dependent upon continuous research productivity” *[Cancer Discovery, 15th* *July 2020]*.

Fourth, there is concern that the funding shortfalls that are being experienced by NGOs that support research are going to have long term effects on research capacity due to such concerns about trainees. The issue was outlined starkly in August 2020 by the head of clinical research at Cancer Research UK, who was discussing the funding cuts reported above:

“‘That is £150m of research on treatments with the potential to save or lengthen lives that will not now take place.' And that cutback is expected to persist for four or five more years, she added. ‘That means hundreds of millions of pounds will be stripped from cancer research in the UK in coming years and the impact will send ripples beyond that. Where are we going to find the funds for the next generation of medical researchers? Where will they work? COVID-19 is going to have a major impact on the research landscape for a long time.”' *[The Observer, 22nd* *Aug 2020]*.

Some ideas have been proposed by some researchers to help mitigate for the lost time, including appeals for computer and technology upgrades (e.g. robotics), but the main appeal appears to have been for additional funding for extra staffing *[Institute of Cancer Research, 30th* *Nov 2020]*.

#### Potential New Areas of Research Interest

The pandemic has focused interest within the research community on certain research areas. Some of these are new, or young, areas of research. Some are not entirely new but have received renewed interest. One new area of focus is on the clinical interaction of COVID-19 and cancer. In mid-2020, data that had been accumulated on cancer patients with COVID-19 began to be reported. For instance, it was reported that “the death rate for [cancer] patients as a whole was 13%, more than twice that reported for all patients with COVID-19,” and that “certain subgroups, such as patients with active (measurable) cancer and those with an impaired performance status, fared much, much worse” *[Vanderbilt University Medical Center, 29th* *May 2020]*. It was suggested by researchers that there was an “urgent need for more data” *[HealthDay News, 2nd* *June 2020]*. Further reports of emerging data in July 2020 quoted researchers as stating that the emergent data “revealed a stark reality that people with cancer are at an increased risk of more serious outcomes from COVID-19” *[HealthDay News, 22nd* *July 2020]*. Such findings coincided with the establishment of studies examining the intersection of COVID-19 and cancer. For example, in April 2020 two NCI-sponsored studies were announced, to examine the genetics of COVID-19 outcomes in cancer patients, and investigate the use of drugs specifically for cancer patients with COVID-19 *[NCI, 17th* *Apr 2020]*.

A related area that received research focus due to the pandemic was the examination of alternative cancer treatment protocols that were deemed to be more COVID-19-safe. For example, an October 2020 article reported that “the COVID-19 pandemic has encouraged greater consideration of shorter-course therapies, as medical institutions look for ways to reduce potential exposure, especially among vulnerable patients with cancer,” quoting an oncologist as saying “in a pandemic, the idea of a single, non-invasive outpatient treatment that doesn't require anaesthesia is appealing in the sense of reducing patient time and transmission risk in the clinic” *[American Society for Radiation Oncology (ASTRO), 26th* *October 2020]*.

The necessitation of providing remote consultations for cancer patients amidst the pandemic placed a renewed research focus on the existing area of telehealth. The increased usage of telehealth amongst cancer clinicians created an unmet research need to properly evaluate telehealth protocols for all its potential advantages and drawbacks. One such study was reported by an article in this review, which opened with “the seemingly endless COVID-19 lockdown has had one potential benefit: the convenience of an online telehealth visit to a health care provider. No waiting room, no parking hassle and costs, no exposure to other people's germs, and no list of things to remember to ask.” The article then reports on the findings of an interventional study in which the value of a digital telehealth system was appraised by patients and healthcare workers, before quoting a study researcher who commented that “there is clearly a lot of enthusiasm … but we know it isn't perfect. Our findings lay a path forward for determining the best ways to integrate patient-reported outcomes in oncology practice” *[Medical Daily, 3rd* *Nov 2020]*. It was hoped by some that telehealth would “make trials more accessible to people ‘who don't have the means to get in the car and drive all day to an appointment”' *[NCI, 29th* *June 2020]*. However, it was also reported that despite telehealth protocols being “an important way of continuing medical care during the pandemic,” a study in the US found that “fewer Black and Hispanic people with cancer used telehealth visits compared with white people with cancer as this year's COVID-19 pandemic unfolded, despite substantial growth in the number of patients who used telehealth” *[ASCO, 5th* *Oct 2020]*. The questions about feasibility, acceptability and social equity regarding telehealth have hence created a renewed research interest in this area.

More widely, there has been a renewed research focus on social and ethnic disparities in cancer. The renewed focus appears to have emerged in part from observations of the way in which COVID-19 disproportionately affected people of colour, women, and deprived communities. As one blog post put it:

“The COVID-19 pandemic has brought to the forefront the longstanding health care disparities faced by communities of colour in the U.S. Many of the same economic and social conditions that are actively contributing to COVID-19 testing and treatment inequities have created disparities in cancer care for decades.” *[Stand Up To Cancer, 16th* *July 2020]*.

The discussion around social disparities was not limited to specialist publications, illustrating the awareness in the general public of this issue. For example, an early 2021 piece in a popular publication, reflecting on the impacts COVID-19 has had on the cancer research community, notes this:

“[The work of the interviewed researchers] aims to develop new approaches to cancer prevention and improvement of long-term cancer survival in minority and economically disadvantaged people, an issue brought into sharper focus during the pandemic as it is now well-known that these people also have the highest risk of contracting, and dying from, COVID-19.” *[Forbes, 13th* *Jan 2021]*.

In the UK, the government was challenged by a major cancer charity “to tackle this cancer inequality when the health service and society begin to recover from the effects of COVID-19 … ‘the pandemic has exposed long-standing inequalities in healthcare across the UK”' *[PharmaTimes Online, 30th* *Sept 2020]*. In addition to the socially uneven effects of the pandemic, it seems that the renewed calls for research addressing cancer health disparities were in part reinforced by high-profile civil rights protests taking place in the US from May 2020 onward, creating a national discussion about systemic inequalities that influenced the research community. The NCI director stated that “as cancer researchers, we each have a role to play in confronting systemic racism and injustice. [we can] commit to taking action to make things better in terms of cancer disparities” *[Axios, 17th* *Sept 2020]*. In a statement following a deal to collaborate with a US University on cancer research, the CEO of a major US healthcare firm wrote:

“The COVID-19 pandemic and the ongoing injustices and recent protests in cities across our nation have amplified the importance of and urgency for innovation and discovery that radically improves the health of all of the communities we serve.” *[The Center Square, 9th* *June 2020]*.

These statements illustrate an apparent overall sentiment that there is a pressing need for cancer researchers to address “growing social inequities” *[Oncology Nursing Society, 6th* *Jan 2021]* and “the widening gap between richer and poorer areas” *[PharmaTimes Online, 30th* *Sept 2020]*.

## Discussion

Our findings show that the field of cancer research has experienced many different types of impact due to the COVID-19 pandemic. The impacts encompass COVID-19 halting cancer research activity; COVID-19 limiting cancer research activity; impacts on cancer diagnosis, cases, and services; forced adaptation of research protocols; impacts on private sector; disruption to supply chains; and availability of resources for cancer research. Consequences from the impacts may be changes to potential changes to research practice; delays to the progression of the field; and potential new areas of research interest.

The difficulties in clinical study activity described here are consistent with studies showing that the onset of the pandemic coincided with decreases to clinical trial accrual rates of around one half (9, 10). Much of this decrease may be attributable to the suspension of trials and a decreased amount of new cancer diagnoses. Issues concerning the maintenance of trial administration during COVID-19 pressures are complex (11), particularly given that COVID-19 caused the disruption of clinical workforce management due to government orders to “stay at home”, restricted working hours, and the need for self-isolation due to a positive COVID-19 test (or contact with someone who tested positive). However, some centres have reported sustainable adaptations to the way they conduct cancer clinical trials, and successful maintenance of pre-pandemic accrual rates despite persistently challenging conditions (12). Such centres offer exemplary approaches for navigating clinical trials through the COVID-19 crisis (should the capacity for such adaptations exist locally). On the other hand, as documented above, there is also evidence that some patients with cancer are more hesitant to participate in research during the pandemic (although it seems that the majority tend to report that their attitude to participation is unchanged by COVID-19) (13, 14). Notwithstanding an apparent marginal tendency for women to be disproportionately less likely to be recruited during the pandemic (9, 13, 14), willingness to participate does not seem to be associated with demographic or clinical factors (13) and may be predominantly driven by the psychological disposition of the individual, and institutional support (15). Qualitative research has indicated that provisions could be put in place to ameliorate peoples' concerns about participating, such as providing detailed information on COVID-19 safety protocols in advance, providing transport, and minimising the number of in-person visits required (15).

Potentially, the great concern is the impact that the pandemic has had on funding provided for cancer research by NGOs, as illustrated by the arresting statements quoted in the analysis above. Since this review was conducted, the Global Cancer Coalitions Network (GCCN) has published a report of a survey conducted of all its member organisations, which examined economic issues (16). The GCCN received survey responses from 104 patient organisations from 46 countries across all continents, representing a variety of cancer types. It was reported that most patient organisations had experienced a fall in income in 2020, with an average decrease of 48% (individual organisations' reports ranged from 10 to 100%). It was further reported that most respondents (59%) did not have emergency government funding available to them, and that all organisations that received emergency funding were from high-income countries. Most respondents (88%) reported that they do not expect their income to return to pre-pandemic levels in 2021 (16). Taken together with the qualitative insights reported above – and given that NGO funding accounts for a substantial portion of cancer research funding – it seems that the economic impacts of the pandemic on NGO funding are widespread and significant enough to have systemic effects on the research ecosystem that one suspects will be long term effects. As of June 2021, the pandemic continues to severely affect many countries' prospects of resuming “normal” economic activity (17). Given that more than one year has now passed since the initial onset of the pandemic, one might now expect to begin to see the effects of decreased funding opportunities for postgraduate researchers, as described in this review.

The account presented here of alarming reductions in cancer diagnoses is consistent with analyses by investigators from several countries (18–22). It is estimated by these reports that thousands of cancer cases may have been missed that would not otherwise have been, and that this situation is likely to translate into excess mortality from cancer after a lag period (22). A study using computer modelling techniques has compared several approaches to alleviating the impact of this backlog, and concluded that, given no additional resources for “catch-up” screening, the approach which prevents the most excess mortality is one in which all screening appointments are delayed and the stopping age is increased to account for the delay (23). There are some considerations with regards to cancer research. Epidemiological cancer studies utilising data from this time period will need to account for the impact of COVID-19 on incidence rates, and potentially for “stage migration” (22). There may be a need for bespoke epidemiological surveillance studies to discern the outcomes of “catch-up” mitigation strategies, such as that outlined above, if these are to be deployed. Furthermore, there may be a rationale for research projects aimed at designing and piloting cancer screening programmes that have resilience to epidemics, or other disruptive environmental threats.

Our tentative finding regarding the impacts on the private sector is roughly consistent with other information indicating a mixed impact on the biopharmaceutical sector (24), with some negative impacts but also fresh market opportunities. It seems that some firms may be showing an increased interest in infectious diseases and immunology research (24). However, it should be noted that the economic impacts on the private biopharmaceutical sector are likely to be experienced unevenly across regions of the world (25).

The apparent shift toward remote meetings between clinicians/researchers and patients documented in this review marks a significant change in the climate. The change may have significant advantages but perhaps should be approached cautiously, given limited data on important psychological and social issues. The move to remote technology could also have important implications for research capacity, since adaptation to this “new normal” may vary between different health/research centres, depending on access to digital resources (both on the part of the centre and the community it serves). It is encouraging to note that in 2021 to date, there has been much investigation of the practicality of telemedicine protocols [e.g. (26–28)]. Findings in this regard appear to be somewhat mixed, with some studies suggesting that most participants were happy to continue telemedicine visits [e.g. (28)], and others suggesting that most participants prefer in-person visits [e.g. (26)]. It should be noted many of these studies are from different parts of the world and differ in the cancer type studied, and importantly, the timing of the study with regards to the COVID-19 public health situation at the time in that country. Remote consultation care protocols perhaps should be deployed with caution, due to the potential to exacerbate health inequalities. One strategy that has been proposed to maintain parity of health access (in a situation where remote consultations are needed) is to ensure that telephone consultations are available for all patients (i.e. not just video consultations) (29). It should of course be noted that telephone consultations do not necessarily confer the same benefit as a video consultation. It is hoped that further work in this area can determine when, where, and for whom such telemedicine approaches are appropriate.

Encouragingly, research focus on social disparities in cancer also seems to have persisted into 2021, with investigators continuing to make the case that “systemic structural socioeconomic disadvantages” produce risk factors for COVID-19 and cancer are similar (30) (p.25). It has further been argued recently that, up to now, “social determinants have received less attention than have genetics and individual health behaviours … we must refine medical training to root out both racial bias and the over-reliance on race over racism as a risk factor for illness” (31) (p.2–5). We agree with this statement and would furthermore advocate for a like-minded attitude to be taken toward gender-based cancer disparities (32). Amongst other propositions, it has been proposed that to facilitate progression in this area, cancer researchers should proactively protect cancer disparity research projects and community outreach efforts, instead of regarding such efforts as “expendable” (30) (p. 26). Policy propositions such as improving access to digital health, financial support for public hospitals, and the expansion of healthcare insurance programmes have also been suggested (30). The issue of underrepresentation in cancer research studies is also a contributing factor and should be actively addressed (32). A continued research focus on social cancer disparities can help provide the empirical data that can be used to continually make the case for beneficial policies in this regard.

### Strengths and Limitations

The aim of this review was to narratively summarise the events of the COVID-19 pandemic in 2020 that have made impacts on the cancer research field. To this end, a strength of this review is the qualitative analysis approach that used a large amount of documentary data from a diverse range of relevant sources, and the time period studied, that included both the first and second waves of the pandemic. There were some limitations to this review. First, the sources used were all in the English language, which meant that the data were skewed toward events taking place in English-speaking countries. Second, with regards to impacts on the private sector, the sources reviewed did not seem to reach any discernible “saturation point” and hence it is assumed the information produced by the analysis was limited in this regard.

## Conclusions

The COVID-19 pandemic had extensive practical and budgetary effects on the field of cancer research in 2020 that were highly plural in nature. The impacts encompass the halting or limiting of research activity; the forced adaptation of research protocols; disruption to supply chains; impacts on cancer diagnosis, cases, and services; availability of resources for cancer research; and impacts on the private sector. Future consequences of the pandemic on the field may include potential changes to future research practice; delays to the progression of the field; and potential new areas of research interest (in particular, digital health, social disparities in cancer, and intersection of cancer and COVID-19). Appraisal of cancer research strategies in a post-COVID world should acknowledge the diversity of the impacts of the pandemic in 2020. Such an understanding might consider substantial limitations (such as on financial resources, limited access to patients for research, decreased patient access to cancer care, staffing issues, administrative delays, or supply chain issues), exacerbated cancer disparities, advances in digital health, and new areas of research related to the intersection of cancer and COVID-19.

## Data Availability Statement

The original contributions presented in the study are included in the article/Supplementary Material, further inquiries can be directed to the corresponding author/s.

## Author Contributions

All authors contributed to the writing of the manuscript. LF, MV, VV, and DM conceived of the study concept and design. LF, ER, KB, and EM screened articles for inclusion. LF conducted the study analysis.

## Funding

This publication was funded through the UK Research and Innovation GCRF Grant [ES/P010962/1].

## Conflict of Interest

The authors declare that the research was conducted in the absence of any commercial or financial relationships that could be construed as a potential conflict of interest.

## Publisher's Note

All claims expressed in this article are solely those of the authors and do not necessarily represent those of their affiliated organizations, or those of the publisher, the editors and the reviewers. Any product that may be evaluated in this article, or claim that may be made by its manufacturer, is not guaranteed or endorsed by the publisher.
