# Supplementary material for: Impact of the COVID-19 Pandemic on Cancer Researchers in 2020: A Qualitative Study of Events to Inform Mitigation Strategies
Source: Front Public Health. 2021 Nov 24;9:741223. doi: 10.3389/fpubh.2021.741223 (PMC8711079; doi:10.3389/fpubh.2021.741223)
Supplement: Supplementary file 1 [file Table_1.DOCX]

Supplementary Material

**Appendix 1** *Sources of data analysed, with number of unique sources analysed.*

| Source of data | Web address of data | Sources | Source of data | Web address of data | Sources |
| --- | --- | --- | --- | --- | --- |
| ABC 15 News | wpde.com | 1 | **Memorial Sloan Kettering Cancer Center** | mskcc.org | 1 |
| ABC 6 News | kaaltv.com | 1 | **Michigan Medicine, University of Michigan** | med.umich.edu | 1 |
| Against Breast Cancer | [General press release] | 1 | **Mr Leigh Geary (Founder, coolsmartphone.com)** | coolsmartphone.com | 1 |
| Alex's Lemonade Stand Foundation (ALSF) | alexslemonade.org | 1 | **MultiCare** | multicare.org | 1 |
| American Association for Cancer Research (AACR) | aacr.org | 1 | **National Cancer Institute (NCI)** | cancer.gov | 3 |
| American Association for the Advancement of Science (AAAS) / Science | sciencemag.org | 1 | **National Cancer Research Institute (NCRI)** | ncri.org.uk | 2 |
| American Cancer Society | cancer.org | 2 | **Nature** | nature.com | 1 |
| American College of Surgeons | facs.org | 1 | **NBC News** | nbcnews.com | 1 |
| American Society for Radiation Oncology (ASTRO) | astro.org | 1 | **Netherlands Cancer Institute** | [General press release] | 1 |
| American Society of Clinical Oncology (ASCO) | cancer.net | 1 | **NJ Spotlight News** | njspotlight.com | 1 |
| Aptitude Health | aptitudehealth.com | 1 | **NPR** | npr.org | 2 |
| AUTO Connected Car News | autoconnectedcar.com | 2 | **OncLive** | onclive.com | 1 |
| Axios | axios.com | 2 | **Oncology Nursing Society** | ons.org | 1 |
| BBC News | bbc.co.uk | 2 | **Ontario Institute for Cancer Research** | oicr.on.ca | 1 |
| Be The Difference Foundation | [General press release] | 1 | **Pancreatic Cancer Action Network** | [General press release] | 1 |
| Benzinga | benzinga.com | 3 | **Patch** | patch.com | 1 |
| BioInvent | [General press release] | 1 | **Pediatric Cancer Research Foundation** | [General press release] | 2 |
| BioPharma Dive | biopharmadive.com | 1 | **Pharmafile** | pharmafile.com | 1 |
| Bloomberg | bloomberg.com | 1 | **pharmaphorum** | pharmaphorum.com | 2 |
| Boston 25 News | boston25news.com | 1 | **PharmaTimes Online** | pharmatimes.com | 18 |
| Boy Genius Report | bgr.com | 1 | **Post Bulletin** | postbulletin.com | 1 |
| Breastcancer.org | breastcancer.org | 1 | **PR Newswire** | prnewswire.co.uk | 1 |
| BriaCell Therapeutics | [General press release] | 2 | **Proactive Investors** | proactiveinvestors.com | 2 |
| Business Matters | bmmagazine.co.uk | 1 | **Proscia** | proscia.com | 1 |
| Cal Matters | calmatters.org | 1 | **Pseudomyxoma Survivor** | [General press release] | 1 |
| Cancer Discovery | cancerdiscovery.aacrjournals.org | 2 | **Research Professional News** | researchprofessionalnews.com | 1 |
| Cancer Research UK | cancerresearchuk.org | 14 | **Rogel Cancer Center, University of Michigan** | rogelcancercenter.org | 1 |
| Cardiff University | cardiff.ac.uk | 1 | **Roundups** | [General press release] | 1 |
| Case Western Reserve University | case.edu | 2 | **RT Magazine** | rtmagazine.com | 3 |
| CTV News | ctvnews.ca | 1 | **Science \| Business** | sciencebusiness.net | 1 |
| CU Anschutz Medical Campus, University of Colorado | cuanschutz.edu | 1 | **Society of Interventional Oncology (SIO)** | sio-central.org | 1 |
| Dana-Farber Cancer Institute | dana-farber.org | 1 | **Stand Up To Cancer** | standuptocancer.org | 1 |
| DC Medical Malpractice and Patient Safety Blog | protectpatientsblog.com | 1 | **STAT** | statnews.com | 1 |
| Dickinson Wright | dickinson-wright.com | 1 | **SWOG Cancer Research Network** | [General press release] | 1 |
| DocWire News | docwirenews.com | 1 | **TangerOutlets** | tangeroutlet.com | 1 |
| ECOG-ACRIN Cancer Research Group | blog-ecog-acrin.org | 1 | **TechBullion** | techbullion.com | 1 |
| EU Reporter | eureporter.co | 2 | **The ASCO Post** | ascopost.com | 1 |
| European Alliance for Personalised Medicine (EAPM) | euapm.eu | 1 | **The Associated Press** | apnews.com | 5 |
| European Alliance for Personalised Medicine (EAPM) | eureporter.co | 1 | **The Associated Press** | [Syndicated content] | 3 |
| European Organisation for Research and Treatment of Cancer (EORTC) | eortc.org | 1 | **The Center Square** | thecentersquare.com | 1 |
| European Pharmaceutical Manufacturer | epmmagazine.com | 1 | **The Cure Starts Now** | thecurestartsnow.org | 1 |
| European Society for Medical Oncology (ESMO) | esmo.org | 3 | **The Economist** | economist.com | 1 |
| Fact.MR | sbwire.com | 1 | **The Francis Crick Institute** | crick.ac.uk | 1 |
| Female First | femalefirst.co.uk | 1 | **The Institute of Cancer Research** | icr.ac.uk | 6 |
| Fierce Biotech | fiercebiotech.com | 1 | **The Jerusalem Post** | jpost.com | 1 |
| Foley & Lardner | foley.com | 1 | **The Lancet** | thelancet.com | 2 |
| Food and Drug Administration (FDA) | fda.gov | 1 | **The Mark Foundation for Cancer Research** | themarkfoundation.org | 1 |
| Forbes | forbes.com | 1 | **The Mercury News** | mercurynews.com | 1 |
| Greenberg Traurig | gtlaw.com | 1 | **The Observer** | theguardian.com | 1 |
| Healio | healio.com | 1 | **The Royal Marsden NHS Foundation Trust** | royalmarsden.nhs.uk | 1 |
| Health Care Compliance Association (HCCA) | jdsupra.com | 1 | **The Simple Dollar** | thesimpledollar.com | 1 |
| HealthDay News | healthday.com | 6 | **The Times of India** | timesofindia.indiatimes.com | 1 |
| HIT Consultant Media | hitconsultant.net | 1 | **The Week** | theweek.co.uk | 3 |
| Imaging Technology News | itnonline.com | 1 | **Thomson Reuters** | reuters.com | 1 |
| International Business Times | ibtimes.com | 4 | **TrialSite News** | trialsitenews.com | 1 |
| Investor Strategy News | ioandc.com | 2 | **University of Birmingham** | birmingham.ac.uk | 1 |
| Jefferson Health / Sidney Kimmel Cancer Center | sidneykimmelcancercenter.  jeffersonhealth.org | 1 | **University of Cincinnati** | uc.edu | 1 |
| Jewish Telegraphic Agency | jta.org | 1 | **University of Delaware** | udel.edu | 1 |
| Juno PR | [General press release] | 1 | **University of Illinois** | uic.edu | 2 |
| KIMT3 News | kimt.com | 1 | **University of Louisville** | louisville.edu | 1 |
| Leicester Mercury | leicestermercury.co.uk | 1 | **Vanderbilt University Medical Center** | vumc.org | 2 |
| Living Beyond Breast Cancer | lbbc.org | 1 | **VentureBeat** | venturebeat.com | 1 |
| Ludwig Cancer Research | ludwigcancerresearch.org | 1 | **Voice of America** | voanews.com | 1 |
| LuxuryLaunches | luxurylaunches.com | 1 | **Walter and Eliza Hall Institute** | wehi.edu.au | 1 |
| MedCity News | medcitynews.com | 1 | **Yorkshire Cancer Research** | yorkshirecancerresearch.org.uk | 1 |
| Medical Daily | medicaldaily.com | 2 | **Zacks Equity Research** | zacks.com | 5 |
| medwire News | medwirenews.com | 2 |  |  |  |
