# Supplementary material for: Impact of the COVID-19 Pandemic on Cancer Researchers in 2020: A Qualitative Study of Events to Inform Mitigation Strategies
Source: Front Public Health. 2021 Nov 24;9:741223. doi: 10.3389/fpubh.2021.741223 (PMC8711079; doi:10.3389/fpubh.2021.741223)
Supplement: Supplementary file 2 [file Data_Sheet_1.DOCX]

| 14/07/2021 10:54 | | | | | | | | | | | | | | | | | |
| --- | --- | --- | --- | --- | --- | --- | --- | --- | --- | --- | --- | --- | --- | --- | --- | --- | --- |
| Coding Summary By Code | | | | | | | | | | | | | | | | | |
| REPRISE content analysis | | | | | | | | | | | | | | | | | |
| 14/07/2021 10:54 | | | | | | | | | | | | | | | | | |
|  | | | **Aggregate** |  | **Classification** |  | **Coverage** |  | **Number Of Coding References** | |  | **Reference Number** |  | **Coded By Initials** |  | **Modified On** |  |
| **Node** | | | | | | | | | | | | | | | | |  |
|  | **Nodes\\Direct impacts\Availability of resources for cancer research\Expenditure of resources on projects studying COVID mitigation in the context of cancer** | | | | | | | | | | | | | | | |  |
|  | | **Document** | | | | | | | | | | | | | | |  |
|  | | | **Files\\180 [9July20]** | | | | | | | | | | | | | |  |
|  |  |  | No |  | All articles |  | 0.0698 |  | 1 | |  | | | | | |  |
|  | | |  |  |  |  |  |  |  | |  | | | | | | |
|  | | | | | | | | | | | | 1 |  | LF |  | 17/02/2021 10:59 |  |
|  | AIM ImmunoTech Inc (NYSEAMERICAN:AIM) revealed on Thursday that it has struck a clinical trial agreement (CTA) with Roswell Park Comprehensive Cancer Center to support its Phase 1/2a trial of Ampligen (rintatolimod) in combination with interferon alfa-2b, in cancer patients with COVID-19, the disease caused by the SARS-CoV-2 coronavirus. Funding for the clinical trial is being provided, in part, through grants from the National Cancer Institute and AIM, as well as institutional support from Roswell Park. In a statement, AIM ImmunoTech CEO Thomas K Equels said: "We are excited to team up with Roswell Park, a leading cancer research center, to test Ampligen as part of an antiviral treatment combination for COVID-19 among cancer patients.' | | | | | | | | | | | | | | | |  |
|  |  | | | | | | | | | | | | | | | |  |
|  | | **PDF** | | | | | | | | | | | | | | |  |
|  | | | **Files\\123 [no date]** | | | | | | | | | | | | | |  |
|  |  |  | No |  | All articles |  | 0.3030 |  | 1 | |  | | | | | |  |
|  | | |  |  |  |  |  |  |  | |  | | | | | | |
|  | | | | | | | | | | | | 1 |  | LF |  | 15/02/2021 16:28 |  |
|  | Yorkshire Cancer Research has teamed up with NHS Trusts across the region to understand the impact of COVID-19 on the care of cancer patients needing life-saving surgery.  The charity is supporting a global study called CovidSurg-Cancer led by Mr Aneel Bhangu, a National Institute for Health Research Clinician Scientist in Global Surgery based at the University of Birmingham.  Researchers across the world will collect data to help answer questions about how hospitals are managing surgery during the pandemic and monitor the effect on patients.  John Edwards, Consultant Thoracic Surgeon at Sheffield Teaching Hospitals NHS Foundation Trust, is leading Yorkshire’s contribution to the research.  Funding from Yorkshire Cancer Research will help coordinate the involvement of hospitals in the region and analyse the data collected.  The results of the study will help develop policy at regional and hospital level for both this outbreak and future pandemics.  1/2  Mr Edwards said: “It is important in these unprecedented times that we conduct research which can inform our decision making and practice not just during this outbreak but for the future too. We are very grateful for the support from Yorkshire Cancer Research.”  Dr Stuart Griffiths, Director of Research and Services at Yorkshire Cancer Research, said: “This research is clearly needed. The findings are urgently required to ensure we can manage cancer as well as possible in the context of the pandemic.” | | | | | | | | | | | | | | | |  |
|  |  |  |  |  |  |  |  |  |  |  |  |  |  |  |  |  |  |
|  |  |  |  |  |  |  |  |  |  |  |  |  |  |  |  |  |  |
|  |  | | | | | | | | | | | | | | | |  |
| Reports\\Coding Summary By Code Report | | | | | | | | | | Page 1 of 123 | | | | | | | |
| 14/07/2021 10:54 | | | | | | | | | | | | | | | | | |
|  | | | **Aggregate** |  | **Classification** |  | **Coverage** |  | **Number Of Coding References** | |  | **Reference Number** |  | **Coded By Initials** |  | **Modified On** |  |
|  | | | **Files\\33 [no date]** | | | | | | | | | | | | | |  |
|  |  |  | No |  | All articles |  | 0.4577 |  | 1 | |  | | | | | |  |
|  | | |  |  |  |  |  |  |  | |  | | | | | | |
|  | | | | | | | | | | | | 1 |  | LF |  | 19/02/2021 16:47 |  |
|  | A study funded by Cancer Research UK (CRUK) has found differing immune responses to COVID-19 among cancer patients, with certain cancer patients experiencing similar response to those without cancer, while others took five times longer than average to recover.  The study, undertaken by a team of researchers led by CRUK clinician scientist Dr Sheeba Irshad, shows that the immune response to COVID-19 is the same in cancer patients with solid tumours when compared to individuals who do not have cancer.  The researchers set out to address two key questions revolving around COVID-19 and cancer care – first, if the immune response to COVID-19 in cancer patients differs to those without cancer; and second, to discover the long-term impact of COVID-19 on the immune systems of cancer patients.  To do this, the researchers analysed the blood of 76 cancer patients, including 41 who had COVID-19 and 35 who had not been exposed to the virus.  Of the participants with cancer, 23 had solid tumours and 18 had blood cancer. The samples from the cancer patients were compared to samples from the blood of people who didn’t have cancer and who were already taking part in the previously published COVID-IP study.  The COVID-IP study examined the impact of COVID-19 on the immune system in cancer patient populations compared to those without cancer.  The CRUK researchers discovered that patients with solid tumours had high levels of COVID19 antibodies that were sustained for up to 78 days following initial exposure to the novel coronavirus.  The study also found that when these patients had recovered from COVID-19, their immune systems returned to pre-COVID-19 functioning.  However, the researchers also found that blood cancer patients had differing responses to the virus, with some taking up to 90 days to recover after the initial signs of infection.  Participants with B cell-related blood cancer fell into three groups of responses to COVID-19 infection. The first group developed antibodies and cleared the virus, similar to the response seen in solid tumour cancer patients and people without cancer.  The second group did not develop antibodies, even after >75 days after first exposure to the virus and they continued to be unable to clear the virus.  The final group developed antibodies against COVID-19 but still remained unable to clear the virus from their system.  "While we need to maintain caution, our study provides some confidence and reassurance to care providers that many of our patients with solid cancers will mount a good immune response against the virus, develop antibodies that last and hopefully resume their cancer treatment as soon as possible," said Dr Sheeba Irshad.  “These conclusions imply that many patients despite being on immunosuppressive therapies will respond satisfactorily to COVID-19 vaccines. For patients with blood cancers, especially those with B-cell malignancies, this may not hold true even in the era of COVID-19 vaccines. Our work suggests that they may be susceptible to persistent infection despite developing antibodies, so the next stage of our study will focus on monitoring their response to the vaccines," she added. | | | | | | | | | | | | | | | |  |
|  |  |  |  |  |  |  |  |  |  |  |  |  |  |  |  |  |  |
|  |  |  |  |  |  |  |  |  |  |  |  |  |  |  |  |  |  |
|  |  |  |  |  |  |  |  |  |  |  |  |  |  |  |  |  |  |
|  |  |  |  |  |  |  |  |  |  |  |  |  |  |  |  |  |  |
|  |  | | | | | | | | | | | | | | | |  |
|  | **Nodes\\Direct impacts\Availability of resources for cancer research\Extreme funding shortfall from NGOs** | | | | | | | | | | | | | | | |  |
|  | | **Document** | | | | | | | | | | | | | | |  |
|  | | | **Files\\117 [24 Sept 20]** | | | | | | | | | | | | | |  |
|  |  |  | No |  | All articles |  | 0.1888 |  | 1 | |  | | | | | |  |
|  | | |  |  |  |  |  |  |  | |  | | | | | | |
|  | | | | | | | | | | | | 1 |  | LF |  | 15/02/2021 15:10 |  |
|  | Due to the impact of the COVID-19 pandemic, cancer research charities are predicting their spending on research could drop by 46%, equating to 167m. Charities play a vital role in UK cancer research. Data collected by the National Cancer Research Institute (NCRI) shows that in the year 2018/19, charities accounted for over 50% of publicly funded cancer research, a total of 362m. With the financial impact of COVID-19, cancer research charities are projecting a 46% decrease in research spend over the next year, and some expect a more significant effect on research spend in the future. This reduction will see overall public investment in cancer research drop by 24%. This projected drop in research funding[1] is reflected nationally across the medical research sector, as data from the Association of Medical Research Charities (AMRC) shows. | | | | | | | | | | | | | | | |  |
|  |  | | | | | | | | | | | | | | | |  |
| Reports\\Coding Summary By Code Report | | | | | | | | | | Page 2 of 123 | | | | | | | |
| 14/07/2021 10:54 | | | | | | | | | | | | | | | | | |
|  | | | **Aggregate** |  | **Classification** |  | **Coverage** |  | **Number Of Coding References** | |  | **Reference Number** |  | **Coded By Initials** |  | **Modified On** |  |
|  | | | **Files\\153 [7Apr20]** | | | | | | | | | | | | | |  |
|  |  |  | No |  | All articles |  | 0.7321 |  | 1 | |  | | | | | |  |
|  | | |  |  |  |  |  |  |  | |  | | | | | | |
|  | | | | | | | | | | | | 1 |  | LF |  | 17/02/2021 10:20 |  |
|  | Cancer Research UK (CRUK) will cut funding to its existing grants and institutes by up to 10% and its national network of Centres by around 20%, driven by a loss in fundraising income as researchers focus efforts on COVID-19.In an open letter to the researcher community, the charity's Iain Foulkes, Professor Karen Vousden, and Professor Charles Swanton said the cuts equate to 44 million.They note that 'universities have closed, laboratories have wound down their activities, experiments have stopped,' and that 'most of our clinical academics, including our Clinical Research Fellows, have been called to the frontline in hospitals all over the country'.They also stress that, around the country, 'CRUK institutes and laboratories are repurposing their resources to help the COVID-19 response'..Further explaining the move, Foulkes, who is executive director of research and innovation at the charity, said: 'COVID-19 has left the whole world in uncharted waters. And the unprecedented measures to control the global COVID-19 pandemic have had a huge impact on both our researchers' ability to carry on in the lab, and on our ability to fundraise.  Faced with a predicted loss of 20-25% of fundraising income, we are forced to look for savings across our current portfolio.''Cancer Research UK funds nearly 50% of the cancer research in the UK and making cuts to research funding is the most difficult decision we have had to make. We don't do so lightly. We have worked hard to ensure the cuts are limited and give our researchers flexibility in how to make them. Ultimately, it is our research that delivers benefit to people affected by cancer, and this remains our first priority. We are hopeful that limiting our spending now will enable us to continue funding life-saving research in the long run.'Cancer doesn't go away during or after COVID-19, but we're incredibly proud of our community of researchers who have been very quick to respond to the crisis, using their kit, skills and talent to support the NHS and the COVID-19 response.'Our mission is so important to people all over the UK and by helping the global effort of tackling COVID-19, we hope we can get back to beating cancer as soon as possible.'WASH | | | | | | | | | | | | | | | |  |
|  |  |  |  |  |  |  |  |  |  |  |  |  |  |  |  |  |  |
|  |  |  |  |  |  |  |  |  |  |  |  |  |  |  |  |  |  |
|  |  | | | | | | | | | | | | | | | |  |
|  | | | **Files\\188 [8Apr20]** | | | | | | | | | | | | | |  |
|  |  |  | No |  | All articles |  | 0.4755 |  | 2 | |  | | | | | |  |
|  | | |  |  |  |  |  |  |  | |  | | | | | | |
|  | | | | | | | | | | | | 1 |  | LF |  | 17/02/2021 11:18 |  |
|  | Cancer Research UK says the fight against cancer could be set back by several years due to a dramatic fall in income during the coronavirus pandemic. The leading charity is cutting research funding by 44m because it expects income to drop by up to 25% as a result of the crisis. See related Will China be sued over coronavirus?  [2] Are countries being honest about coronavirus?[3] Iain Foulkes, executive director of research and innovation, explained that the charity funds nearly 50% of cancer research in the UK, and that 'making cuts to research funding is the most difficult decision we have had to make'. He added: 'We are hopeful that limiting our spending now will enable us to continue funding life-saving research in the long run.' The charity told The Guardian[4] the impact of the new coronavirus has been significant, saying its 'shops had closed, mass fundraising events had stopped and legacies had reduced'. This has led to a reduction of around 120 million in income. | | | | | | | | | | | | | | | |  |
|  |  |  |  |  |  |  |  |  |  |  |  |  |  |  |  |  |  |
|  |  | | | | | | | | | | | | | | | |  |
|  | | | | | | | | | | | | 2 |  | LF |  | 17/02/2021 11:19 |  |
|  | Several charities have complained of a fall in income recently. The Conversation[7] says the pandemic is a 'perfect storm' for charities: an increase in demand for services at the same time as volunteers are forced to stay at home and donations are drying up. 'From food banks to mental health experts, the charitable sector is providing plenty of people with support in these troubled times,' says the BBC[8], 'but charities themselves are reeling from the financial impact of the crisis'. This perfect storm is expected to lead to a total loss in charity income of 4.3 billion over a 12-week period. The first aid charity St John Ambulance Association, which is helping the NHS cope with coronavirus cases, said it could go bust in August unless it receives state aid. Caron Bradshaw, chief executive of the Charity Finance Group, said onTwitter[9]that the charity sector requires its own package of support from the government. 'Please recognise the impact losing charities - big and small - will have on our society,' she wrote. 'Charities are to society what bees are to the environment. We work away, often in the background, often unnoticed.' Pressure is now mounting on chancellor Rishi Sunak after more than 100 parliamentarians - including several Conservatives - demanded an 'immediate injection of money' to stop charities going to the wall, reports PoliticsHome[10]. | | | | | | | | | | | | | | | |  |
|  |  |  |  |  |  |  |  |  |  |  |  |  |  |  |  |  |  |
|  |  | | | | | | | | | | | | | | | |  |
|  | | | | | | | | | | | | | | | | | |
|  | | | | | | | | | | | | | | | | | |
| Reports\\Coding Summary By Code Report | | | | | | | | | | Page 3 of 123 | | | | | | | |
| 14/07/2021 10:54 | | | | | | | | | | | | | | | | | |
|  | | | **Aggregate** |  | **Classification** |  | **Coverage** |  | **Number Of Coding References** | |  | **Reference Number** |  | **Coded By Initials** |  | **Modified On** |  |
|  | | | **Files\\221 [12Aug20]** | | | | | | | | | | | | | |  |
|  |  |  | No |  | All articles |  | 0.2489 |  | 1 | |  | | | | | |  |
|  | | |  |  |  |  |  |  |  | |  | | | | | | |
|  | | | | | | | | | | | | 1 |  | LF |  | 17/02/2021 14:15 |  |
|  | The financial fall-out from COVID-19 is having a dramatic impact on pediatric cancer research. Donations supporting this already under-funded area of research are sharply declining, with grave implications. In addition to interrupting research now underway and losing the benefits that might be gained, diminished funding puts at risk the lives of a generation of children diagnosed with cancer.'We're fighting for the future of kids with cancer. We're helpless if we can't raise the funds to continue advancing exploration of treatments that are less toxic, more targeted, and improve quality of life,' said Jeri Wilson, Executive Director, Pediatric Cancer Research Foundation.'The need for generating financial support for pediatric cancer research has never been greater,' said Dr. Theodore Moore, UCLA Mattel Children's Hospital. "Opportunities to harness the power of one's own immune system to clear cancer rather than chemotherapy have never been greater. We're at a real turning point now and we are reliant on contributions from the public to enable us to continue funding research at pre-COVID-19 levels. The government has never adequately funded pediatric cancer and now we face the tragic reality that budget shortfalls may impact research that will save children's lives and minimize the negative effects of chemotherapy.'Born | | | | | | | | | | | | | | | |  |
|  |  |  |  |  |  |  |  |  |  |  |  |  |  |  |  |  |  |
|  |  | | | | | | | | | | | | | | | |  |
|  | | | **Files\\222 [18Nov20]** | | | | | | | | | | | | | |  |
|  |  |  | No |  | All articles |  | 0.1782 |  | 1 | |  | | | | | |  |
|  | | |  |  |  |  |  |  |  | |  | | | | | | |
|  | | | | | | | | | | | | 1 |  | LF |  | 17/02/2021 14:18 |  |
|  | PanCAN leads research initiatives working to transform pancreatic cancer outcomes through early detection and new treatment approaches. Since 2003, the organization has invested $126 million in pancreatic cancer research. This year, it will invest $23 million toward its research initiatives. PanCAN is able to do this through donor support. Funding for pancreatic cancer research programs and services has been reduced significantly due to the COVID-19 pandemic. "We are so grateful to Mindy for her ongoing generosity and support and her vulnerability in sharing her story with us this November. She'll undoubtedly bring more urgency and attention to this deadly disease that is in critical need of additional research and funding," said Julie Fleshman, PanCAN's president and CEO. | | | | | | | | | | | | | | | |  |
|  |  | | | | | | | | | | | | | | | |  |
|  | | | **Files\\282 [8Dec20]** | | | | | | | | | | | | | |  |
|  |  |  | No |  | All articles |  | 0.4717 |  | 1 | |  | | | | | |  |
|  | | |  |  |  |  |  |  |  | |  | | | | | | |
|  | | | | | | | | | | | | 1 |  | LF |  | 17/02/2021 16:59 |  |
|  | Cancer Research UK has announced further catastrophic cuts in the wake of the coronavirus pandemic, slashing 45 million from its research budget, leaving dozens of potential life-saving projects and hundreds of world-class scientists unfunded.This move follows an earlier 44 million cut made to current grants at the start of the pandemic, and the charity saying it would not be able to fund any new clinical trials this year.The new cuts have led to 24 fewer research programmes, 68 fewer projects and 12 fewer fellowships, and CR UK says there will be around 328 fewer researchers working on their research.The charity stresses that these cuts to the research portfolio 'will not only slow down future breakthroughs for people with cancer but could seriously reduce the chances of reaching the charity's goal of three in four surviving their cancer by 2034.'The drop in income caused by the pandemic means that around 100 fewer grants will be funded, spanning longer-term multi-million-pound research programmes, specific research projects and fellowships that support scientists at all career stages.Moreover, the charity said it will have to continue looking at ongoing reductions in other parts of the research budget.If the situation remains unchanged, CR UK has warned that it could be spending 150 million less per year by 2024 as it faces a potential 300 million decline in fundraising income over the next three years. | | | | | | | | | | | | | | | |  |
|  |  |  |  |  |  |  |  |  |  |  |  |  |  |  |  |  |  |
|  |  | | | | | | | | | | | | | | | |  |
|  | | | | | | | | | | | | | | | | | |
|  | | | | | | | | | | | | | | | | | |
| Reports\\Coding Summary By Code Report | | | | | | | | | | Page 4 of 123 | | | | | | | |
| 14/07/2021 10:54 | | | | | | | | | | | | | | | | | |
|  | | | **Aggregate** |  | **Classification** |  | **Coverage** |  | **Number Of Coding References** | |  | **Reference Number** |  | **Coded By Initials** |  | **Modified On** |  |
|  | | | **Files\\299 [24Jun20]** | | | | | | | | | | | | | |  |
|  |  |  | No |  | All articles |  | 0.5558 |  | 1 | |  | | | | | |  |
|  | | |  |  |  |  |  |  |  | |  | | | | | | |
|  | | | | | | | | | | | | 1 |  | LF |  | 19/02/2021 15:01 |  |
|  | Charities including Cancer Research UK (CR UK) and the British Heart Foundation are warning that research funding will take a substantial hit in the wake of COVID-19 and that this will likely have significant consequences on survival.CR UK said it could be forced to cut 150 million per year from its research funding 'as the COVID-19 pandemic decimates its income', which could 'set back the progress we have seen in survival for people with cancer'.Back in April the charity announced a cut of 44 million in funding across its research portfolio because of the pandemic, but is now preparing for a 30% fall in income in the 2020/21 financial year, with further losses in the next year, following temporary closures of its shops and major fundraising events being cancelled during the COVID-19 crisis.Cutting 150 million each year would mean: a major contraction in the charity's research infrastructure with potential closure of sites around the country; thousands of early-career scientists left unsupported, and their ideas for beating cancer unfunded; and cancelling plans to fund new projects in the short term, including new clinical trials, holding back the development of new cancer treatments, it said.'Cuts of this magnitude to Cancer Research UK's research funding will have a deep and long-lasting impact on our vibrant life-sciences industry, a sector that provided science-led solutions to the pandemic, highlighting the need for us to keep this strong and intact.  A loss of 150 million is the equivalent of 10 years' worth of clinical trials going unfunded,' noted Professor Charles Swanton, CR UK's chief clinician.The BHF has warned that the impact of the pandemic means that its net income, and resulting investment in new research, is likely to drop by up to 50% this year, from 100 million to around 50 million.Such a sharp fall 'could have a catastrophic impact on UK cardiovascular research, the research careers of thousands of young scientists, and advances in diagnostics, treatments and cures for people with heart and circulatory diseases', it said.The charity funds over half of non-commercial research into heart and circulatory diseases in the UK, but the loss of income from shop closures and cancellation of fundraising events 'has created the biggest crisis in its 60-year history'.Furthermore, it could take several years for funding to return to pre-pandemic levels, the charity warned.'Ultimately | | | | | | | | | | | | | | | |  |
|  |  |  |  |  |  |  |  |  |  |  |  |  |  |  |  |  |  |
|  |  |  |  |  |  |  |  |  |  |  |  |  |  |  |  |  |  |
|  |  | | | | | | | | | | | | | | | |  |
|  | | | **Files\\341 [8Oct20]** | | | | | | | | | | | | | |  |
|  |  |  | No |  | All articles |  | 0.4734 |  | 1 | |  | | | | | |  |
|  | | |  |  |  |  |  |  |  | |  | | | | | | |
|  | | | | | | | | | | | | 1 |  | LF |  | 19/02/2021 17:04 |  |
|  | Twenty charities representing people affected by rare and less common cancers have come together to respond to and highlight the disruption caused by the COVID-19 pandemic.The charities, which include Target Ovarian Cancer, blood cancer charity DKMS and Brain Tumour Research, launched a campaign asking their supporter to take on '20 for 20' on the 20 September 2020.The campaign is supported by biopharma company Takeda as its headline supporter, alongside Novartis as its campaign partner.Those partaking in the campaign are asked to complete a challenge or activity based on the number 20 for 20 consecutive days, e.g. running for 20 minutes a day or baking 20 cakes.The '20 for 20' charities support the over 87,000 patients diagnosed every year who are diagnosed with rare and less common cancers in the UK and are also key drivers of cancer research.Together, these charities have funded around 6m in research - although this has been greatly impacted by the COVID-19 pandemic, with the charities reporting a sharp fall in income since lockdown began in March. | | | | | | | | | | | | | | | |  |
|  |  |  |  |  |  |  |  |  |  |  |  |  |  |  |  |  |  |
|  |  | | | | | | | | | | | | | | | |  |
|  | | | **Files\\381 [29Nov20]** | | | | | | | | | | | | | |  |
|  |  |  | No |  | All articles |  | 0.1446 |  | 1 | |  | | | | | |  |
|  | | |  |  |  |  |  |  |  | |  | | | | | | |
|  | | | | | | | | | | | | 1 |  | LF |  | 07/03/2021 11:18 |  |
|  | In 2020, the global Covid-19 pandemic has caused dramatic repercussions around the world. According to CAF research, charities have reported a significant increase in demand for their services while more than half (53%) have suffered a drop in donations. Charities estimate they will see a 10bn funding gap and without help, many may be forced to close their doors and Pseudomyxoma Survivor is no different having experienced a 50% drop in donations so far this year.A very small charity with a big heart, Pseudomyxoma Survivor supports those affected by the rare cancer pseudomyxoma peritonei (PMP), appendix cancers and other rare peritoneal malignancies. | | | | | | | | | | | | | | | |  |
|  |  | | | | | | | | | | | | | | | |  |
|  | | | | | | | | | | | | | | | | | |
| Reports\\Coding Summary By Code Report | | | | | | | | | | Page 5 of 123 | | | | | | | |
| 14/07/2021 10:54 | | | | | | | | | | | | | | | | | |
|  | | | **Aggregate** |  | **Classification** |  | **Coverage** |  | **Number Of Coding References** | |  | **Reference Number** |  | **Coded By Initials** |  | **Modified On** |  |
|  | | | **Files\\391 [1Dec20]** | | | | | | | | | | | | | |  |
|  |  |  | No |  | All articles |  | 0.2930 |  | 1 | |  | | | | | |  |
|  | | |  |  |  |  |  |  |  | |  | | | | | | |
|  | | | | | | | | | | | | 1 |  | LF |  | 07/03/2021 11:20 |  |
|  | Several London charities have teamed up with a growing number of UK charities in joining forces to ask the public in a video appeal, to donate digital pennies to help them survive the pandemic. London charities including World Cancer Research Fund (WCRF), CLIC Sargent, BEfriend, Education Support, Beam, Missing People, Only Connect and the National Emergencies Trust, are just some of the London charities involved in forming the alliance with Roundups. The video (shown below), features over 20 charity representatives from all over the UK, who tell the story of this year's Covid-19 impact on the sector.  Charity bosses are hoping that by asking for very small donations, they will be able to reach more people and raise vital funds.Roundups is an app that links with your card, rounds up your payments and donates the difference to your chosen charity as you spend.The #RoundupsPennyAppeal comes at a time where charities are having to search for new ways to plug the anticipated 12.4 billion shortfall caused by Covid-19, where the lack of funding could see 10% of charities bankrupt and 60,000 charity jobs lost. | | | | | | | | | | | | | | | |  |
|  |  |  |  |  |  |  |  |  |  |  |  |  |  |  |  |  |  |
|  |  | | | | | | | | | | | | | | | |  |
|  | | | **Files\\465 [9Apr20]** | | | | | | | | | | | | | |  |
|  |  |  | No |  | All articles |  | 0.0969 |  | 1 | |  | | | | | |  |
|  | | |  |  |  |  |  |  |  | |  | | | | | | |
|  | | | | | | | | | | | | 1 |  | LF |  | 07/03/2021 16:53 |  |
|  | The chancellor has unveiled 750m of extra funding for charities amid the coronavirus outbreak, but his measure has already been dismissed as a 'sticking plaster'. The Guardian[2] says 'some of the best-known national charities are in dire straits' as revenue normally generated by charity shops and fundraising events 'dries up' during the Covid-19 lockdown[3]. Rishi Sunak said that 360m would be directly allocated by government departments to charities providing key services and supporting the vulnerable during the pandemic. | | | | | | | | | | | | | | | |  |
|  |  | | | | | | | | | | | | | | | |  |
|  | | **PDF** | | | | | | | | | | | | | | |  |
|  | | | **Files\\1 (2) [8th Oct 20]** | | | | | | | | | | | | | |  |
|  |  |  | No |  | All articles |  | 0.0237 |  | 1 | |  | | | | | |  |
|  | | |  |  |  |  |  |  |  | |  | | | | | | |
|  | | | | | | | | | | | | 1 |  | LF |  | 15/02/2021 12:03 |  |
|  | For the most part, research projects funded by government appropriations have not been hobbled by the pandemic, but some projects supported by private philanthropy face a funding gap. COVID-19 has drastically decreased donations to cancer-focused philanthropic organizations. The American Cancer Society, for example, expects a $200 million decrease in donations this year and has not been able to accept applications for research grants for the Fall grant cycle. | | | | | | | | | | | | | | | |  |
|  |  | | | | | | | | | | | | | | | |  |
|  | | | **Files\\10 [21 Oct 20]** | | | | | | | | | | | | | |  |
|  |  |  | No |  | All articles |  | 0.0293 |  | 2 | |  | | | | | |  |
|  | | |  |  |  |  |  |  |  | |  | | | | | | |
|  | | | | | | | | | | | | 1 |  | LF |  | 15/02/2021 12:10 |  |
|  | Research projects have been delayed, scientists have had to renegotiate funding deals and cancer charities face huge economic challenges. | | | | | | | | | | | | | | | |  |
|  |  | | | | | | | | | | | | | | | |  |
|  | | | | | | | | | | | | | | | | | |
| Reports\\Coding Summary By Code Report | | | | | | | | | | Page 6 of 123 | | | | | | | |
| 14/07/2021 10:54 | | | | | | | | | | | | | | | | | |
|  | | | **Aggregate** |  | **Classification** |  | **Coverage** |  | **Number Of Coding References** | |  | **Reference Number** |  | **Coded By Initials** |  | **Modified On** |  |
|  | | | | | | | | | | | | | | | | | |
|  | | | | | | | | | | | | 2 |  | LF |  | 15/02/2021 12:21 |  |
|  | Research funding more broadly, however, is looking less certain as medical charities cancel fundraisers and the economic toll leads to fewer donations.  In the U.K., cancer charities — which fund over half of publicly-run cancer research — are anticipated to see a $216 million drop in contributions available for research over the next year,  the National Cancer Research Institute has said. The American Cancer Society has issued an appeal for donations.  “This will undoubtedly delay discovery of new therapies, and it will undoubtedly, have had a really signiﬁcant impact on cancer patients,” Walker from Cancer Research U.K. said. | | | | | | | | | | | | | | | |  |
|  |  |  |  |  |  |  |  |  |  |  |  |  |  |  |  |  |  |
|  |  | | | | | | | | | | | | | | | |  |
|  | | | **Files\\101 [16 July 20]** | | | | | | | | | | | | | |  |
|  |  |  | No |  | All articles |  | 0.2924 |  | 3 | |  | | | | | |  |
|  | | |  |  |  |  |  |  |  | |  | | | | | | |
|  | | | | | | | | | | | | 1 |  | LF |  | 15/02/2021 12:22 |  |
|  | The largest charitable funder of cancer research in the world is to make signi~cant reductions in research spending and cut hundreds of jobs, after a battering by the COVID-19 pandemic.  Cancer Research UK (CRUK) said its planned research spend over the next four to ~ve years needs to be reduced by £150 million. The charity has already cut £44 million from research this year.  It also announced plans to cut almost a quarter of its workforce, with up to 345 redundancies over the next six months.  The charity has projected a £300 million drop in fundraising income overall in the next three years, including £160 million in this year alone.  “We’re living through a global crisis unlike any other and, as it’s unfolded, it’s become clear that there’ll be a huge economic impact for years to come,” said Michelle Mitchell, CRUK chief executive. | | | | | | | | | | | | | | | |  |
|  |  |  |  |  |  |  |  |  |  |  |  |  |  |  |  |  |  |
|  |  | | | | | | | | | | | | | | | |  |
|  | | | | | | | | | | | | 2 |  | LF |  | 15/02/2021 12:25 |  |
|  | UK medical research charities are reporting huge coronavirus losses and are collectively appealing (https://sciencebusiness.net/news/uk-medical-researchcharities-make-appeal-ps620m-rescue-fund-after-income-falls-41) to the government to co-fund a £620 million relief fund to help them weather the 41 per cent fall in incomes they have su~ered. | | | | | | | | | | | | | | | |  |
|  |  | | | | | | | | | | | | | | | |  |
|  | | | | | | | | | | | | 3 |  | LF |  | 15/02/2021 12:27 |  |
|  | The Association of Medical Research Charities, a body representing 160 charities that between them put £1.9 billion into R&D last year, estimates that, without government support, it will take four and a half years for spending to return to the 2019 level. | | | | | | | | | | | | | | | |  |
|  |  | | | | | | | | | | | | | | | |  |
|  | | | **Files\\104 [1 Oct 20]** | | | | | | | | | | | | | |  |
|  |  |  | No |  | All articles |  | 0.0684 |  | 1 | |  | | | | | |  |
|  | | |  |  |  |  |  |  |  | |  | | | | | | |
|  | | | | | | | | | | | | 1 |  | LF |  | 15/02/2021 13:01 |  |
|  | The American Cancer Society has experienced major fundraising losses during the coronavirus pandemic.  "Donations are key right now," said Matheny. "We are possibly going to lose a decade of research because of COVID." | | | | | | | | | | | | | | | |  |
|  |  | | | | | | | | | | | | | | | |  |
|  | | | **Files\\7 (2) [no date]** | | | | | | | | | | | | | |  |
|  |  |  | No |  | All articles |  | 0.0650 |  | 1 | |  | | | | | |  |
|  | | |  |  |  |  |  |  |  | |  | | | | | | |
|  | | | | | | | | | | | | 1 |  | LF |  | 08/03/2021 16:49 |  |
|  | Cancer Research Funding Challenge  Due to the economic effects of COVID-19, your American Cancer Society is facing a significant funding shortage that is threatening our ability to invest in vital cancer research.  Without your help, our cancer research funding could be cut in half.  Please help us ensure lifesaving cancer research doesn't pause during the pandemic. | | | | | | | | | | | | | | | |  |
|  |  | | | | | | | | | | | | | | | |  |
| Reports\\Coding Summary By Code Report | | | | | | | | | | Page 7 of 123 | | | | | | | |
| 14/07/2021 10:54 | | | | | | | | | | | | | | | | | |
|  | | | **Aggregate** |  | **Classification** |  | **Coverage** |  | **Number Of Coding References** | |  | **Reference Number** |  | **Coded By Initials** |  | **Modified On** |  |
|  | | | **Files\\8 (2) [17Dec20]** | | | | | | | | | | | | | |  |
|  |  |  | No |  | All articles |  | 0.1199 |  | 2 | |  | | | | | |  |
|  | | |  |  |  |  |  |  |  | |  | | | | | | |
|  | | | | | | | | | | | | 1 |  | LF |  | 08/03/2021 17:42 |  |
|  | On Dec 8, 2020, Cancer Research UK (CRUK) announced cuts of £45 million to its research budget. The charity has been badly a~ected by the COVID-19 pandemic, which forced the cancellation of fundraising events and the suspension of trading at the 600 CRUK shops across the country. Earlier this year, CRUK applied £44 million worth of cuts. It expects to make a further set of reductions in April, 2021. “We are planning for a managed decrease in spending on research from pre-COVID levels of £400–450 million to somewhere around £250 million”, said Iain Foulkes, CRUK's executive director of research and innovation. CRUK is responsible for roughly half of publicly funded research into cancer in the UK.  The latest round of cuts amount to 12 fewer fellowships, 24 fewer 5-year research programmes, and 68 fewer projects, which are typically 3-year programmes. Several hundred researchers will have to look elsewhere for funding. CRUK has not funded any new clinical trials this year. The cuts that are projected for 2021 will hit the institutions supported by the charity. It is hard to imagine a scenario in which these measures do not have serious consequences. | | | | | | | | | | | | | | | |  |
|  |  |  |  |  |  |  |  |  |  |  |  |  |  |  |  |  |  |
|  |  | | | | | | | | | | | | | | | |  |
|  | | | | | | | | | | | | 2 |  | LF |  | 08/03/2021 17:43 |  |
|  | The situation is similar outside the UK. The Canadian Cancer Society has predicted that the pandemic will cost them CA$100 million in lost donations during the ongoing financial year, which amounts to more than half their budget. The American Cancer Society (ACS) has seen a decrease in revenue of around US$200 million. “The pandemic struck during our peak fundraising season”, said William Cance, Chief Medical and Scientific O~icer at ACS. The charity has cut its expenditure on new research from $100 million to $50 million. | | | | | | | | | | | | | | | |  |
|  |  | | | | | | | | | | | | | | | |  |
|  | **Nodes\\Direct impacts\Availability of resources for cancer research\Extreme funding shortfall from NGOs\Cancer site-specific research suffers** | | | | | | | | | | | | | | | |  |
|  | | **Document** | | | | | | | | | | | | | | |  |
|  | | | **Files\\117 [24 Sept 20]** | | | | | | | | | | | | | |  |
|  |  |  | No |  | All articles |  | 0.0772 |  | 1 | |  | | | | | |  |
|  | | |  |  |  |  |  |  |  | |  | | | | | | |
|  | | | | | | | | | | | | 1 |  | LF |  | 15/02/2021 15:11 |  |
|  | The greatest impact will be seen on research focussed on specific cancer types, as a large proportion of site-specific cancer research funding[2] comes from charities (70%). Health departments and medical research councils are large funders of non-site-specific research and the research infrastructure that supports all types of cancer research. | | | | | | | | | | | | | | | |  |
|  |  | | | | | | | | | | | | | | | |  |
|  | | | | | | | | | | | | | | | | | |
|  | | | | | | | | | | | | | | | | | |
|  | | | | | | | | | | | | | | | | | |
| Reports\\Coding Summary By Code Report | | | | | | | | | | Page 8 of 123 | | | | | | | |
| 14/07/2021 10:54 | | | | | | | | | | | | | | | | | |
|  | | | **Aggregate** |  | **Classification** |  | **Coverage** |  | **Number Of Coding References** | |  | **Reference Number** |  | **Coded By Initials** |  | **Modified On** |  |
|  | | | **Files\\221 [12Aug20]** | | | | | | | | | | | | | |  |
|  |  |  | No |  | All articles |  | 0.2489 |  | 1 | |  | | | | | |  |
|  | | |  |  |  |  |  |  |  | |  | | | | | | |
|  | | | | | | | | | | | | 1 |  | LF |  | 17/02/2021 14:15 |  |
|  | The financial fall-out from COVID-19 is having a dramatic impact on pediatric cancer research. Donations supporting this already under-funded area of research are sharply declining, with grave implications. In addition to interrupting research now underway and losing the benefits that might be gained, diminished funding puts at risk the lives of a generation of children diagnosed with cancer.'We're fighting for the future of kids with cancer. We're helpless if we can't raise the funds to continue advancing exploration of treatments that are less toxic, more targeted, and improve quality of life,' said Jeri Wilson, Executive Director, Pediatric Cancer Research Foundation.'The need for generating financial support for pediatric cancer research has never been greater,' said Dr. Theodore Moore, UCLA Mattel Children's Hospital. "Opportunities to harness the power of one's own immune system to clear cancer rather than chemotherapy have never been greater. We're at a real turning point now and we are reliant on contributions from the public to enable us to continue funding research at pre-COVID-19 levels. The government has never adequately funded pediatric cancer and now we face the tragic reality that budget shortfalls may impact research that will save children's lives and minimize the negative effects of chemotherapy.'Born | | | | | | | | | | | | | | | |  |
|  |  |  |  |  |  |  |  |  |  |  |  |  |  |  |  |  |  |
|  |  | | | | | | | | | | | | | | | |  |
|  | **Nodes\\Direct impacts\Availability of resources for cancer research\Extreme funding shortfall from NGOs\Closure of charity shops** | | | | | | | | | | | | | | | |  |
|  | | **Document** | | | | | | | | | | | | | | |  |
|  | | | **Files\\188 [8Apr20]** | | | | | | | | | | | | | |  |
|  |  |  | No |  | All articles |  | 0.1981 |  | 1 | |  | | | | | |  |
|  | | |  |  |  |  |  |  |  | |  | | | | | | |
|  | | | | | | | | | | | | 1 |  | LF |  | 17/02/2021 11:19 |  |
|  | Cancer Research UK says the fight against cancer could be set back by several years due to a dramatic fall in income during the coronavirus pandemic. The leading charity is cutting research funding by 44m because it expects income to drop by up to 25% as a result of the crisis. See related Will China be sued over coronavirus?  [2] Are countries being honest about coronavirus?[3] Iain Foulkes, executive director of research and innovation, explained that the charity funds nearly 50% of cancer research in the UK, and that 'making cuts to research funding is the most difficult decision we have had to make'. He added: 'We are hopeful that limiting our spending now will enable us to continue funding life-saving research in the long run.' The charity told The Guardian[4] the impact of the new coronavirus has been significant, saying its 'shops had closed, mass fundraising events had stopped and legacies had reduced'. This has led to a reduction of around 120 million in income. | | | | | | | | | | | | | | | |  |
|  |  |  |  |  |  |  |  |  |  |  |  |  |  |  |  |  |  |
|  |  | | | | | | | | | | | | | | | |  |
|  | | | **Files\\282 [8Dec20]** | | | | | | | | | | | | | |  |
|  |  |  | No |  | All articles |  | 0.2210 |  | 1 | |  | | | | | |  |
|  | | |  |  |  |  |  |  |  | |  | | | | | | |
|  | | | | | | | | | | | | 1 |  | LF |  | 17/02/2021 17:00 |  |
|  | CR UK has warned that it could be spending 150 million less per year by 2024 as it faces a potential 300 million decline in fundraising income over the next three years.'COVID-19 has slowed down our efforts to beat cancer.  The closures of our charity shops and the cancellation of our fundraising events across the country means we have less money available for life-saving research, but we will never stop,' said Michelle Mitchell, the charity's chief executive.'We have always relied on the generous donations of all our supporters, but we need them now more than ever so we can continue to achieve these ambitions and so that together, we can still beat cancer.'Dr | | | | | | | | | | | | | | | |  |
|  |  | | | | | | | | | | | | | | | |  |
|  | | | | | | | | | | | | | | | | | |
| Reports\\Coding Summary By Code Report | | | | | | | | | | Page 9 of 123 | | | | | | | |
| 14/07/2021 10:54 | | | | | | | | | | | | | | | | | |
|  | | | **Aggregate** |  | **Classification** |  | **Coverage** |  | **Number Of Coding References** | |  | **Reference Number** |  | **Coded By Initials** |  | **Modified On** |  |
|  | | | **Files\\299 [24Jun20]** | | | | | | | | | | | | | |  |
|  |  |  | No |  | All articles |  | 0.1828 |  | 1 | |  | | | | | |  |
|  | | |  |  |  |  |  |  |  | |  | | | | | | |
|  | | | | | | | | | | | | 1 |  | LF |  | 19/02/2021 15:01 |  |
|  | The BHF has warned that the impact of the pandemic means that its net income, and resulting investment in new research, is likely to drop by up to 50% this year, from 100 million to around 50 million.Such a sharp fall 'could have a catastrophic impact on UK cardiovascular research, the research careers of thousands of young scientists, and advances in diagnostics, treatments and cures for people with heart and circulatory diseases', it said.The charity funds over half of non-commercial research into heart and circulatory diseases in the UK, but the loss of income from shop closures and cancellation of fundraising events 'has created the biggest crisis in its 60-year history'.Furthermore, it could take several years for funding to return to pre-pandemic levels, the charity warned.'Ultimately | | | | | | | | | | | | | | | |  |
|  |  | | | | | | | | | | | | | | | |  |
|  | | **PDF** | | | | | | | | | | | | | | |  |
|  | | | **Files\\101 [16 July 20]** | | | | | | | | | | | | | |  |
|  |  |  | No |  | All articles |  | 0.1339 |  | 1 | |  | | | | | |  |
|  | | |  |  |  |  |  |  |  | |  | | | | | | |
|  | | | | | | | | | | | | 1 |  | LF |  | 15/02/2021 12:24 |  |
|  | The charity has projected a £300 million drop in fundraising income overall in the next three years, including £160 million in this year alone.  “We’re living through a global crisis unlike any other and, as it’s unfolded, it’s become clear that there’ll be a huge economic impact for years to come,” said Michelle Mitchell, CRUK chief executive.  As the UK went into lockdown in March, charity shops were closed, fundraising events were cancelled, and donations were diverted to National Health Service charities. All sta~ have gone to 80 per cent pay.  CRUK does not have an endowment and is dependent on its shops, events and donations for all of its income. | | | | | | | | | | | | | | | |  |
|  |  | | | | | | | | | | | | | | | |  |
|  | **Nodes\\Direct impacts\Availability of resources for cancer research\Extreme funding shortfall from NGOs\Diversion of public donations** | | | | | | | | | | | | | | | |  |
|  | | **PDF** | | | | | | | | | | | | | | |  |
|  | | | **Files\\101 [16 July 20]** | | | | | | | | | | | | | |  |
|  |  |  | No |  | All articles |  | 0.1339 |  | 1 | |  | | | | | |  |
|  | | |  |  |  |  |  |  |  | |  | | | | | | |
|  | | | | | | | | | | | | 1 |  | LF |  | 15/02/2021 12:24 |  |
|  | The charity has projected a £300 million drop in fundraising income overall in the next three years, including £160 million in this year alone.  “We’re living through a global crisis unlike any other and, as it’s unfolded, it’s become clear that there’ll be a huge economic impact for years to come,” said Michelle Mitchell, CRUK chief executive.  As the UK went into lockdown in March, charity shops were closed, fundraising events were cancelled, and donations were diverted to National Health Service charities. All sta~ have gone to 80 per cent pay.  CRUK does not have an endowment and is dependent on its shops, events and donations for all of its income. | | | | | | | | | | | | | | | |  |
|  |  | | | | | | | | | | | | | | | |  |
|  | | | | | | | | | | | | | | | | | |
| Reports\\Coding Summary By Code Report | | | | | | | | | | Page 10 of 123 | | | | | | | |
| 14/07/2021 10:54 | | | | | | | | | | | | | | | | | |
|  | | | **Aggregate** |  | **Classification** |  | **Coverage** |  | **Number Of Coding References** | |  | **Reference Number** |  | **Coded By Initials** |  | **Modified On** |  |
|  | **Nodes\\Direct impacts\Availability of resources for cancer research\Extreme funding shortfall from NGOs\Some fundraising events cancelled** | | | | | | | | | | | | | | | |  |
|  | | **Document** | | | | | | | | | | | | | | |  |
|  | | | **Files\\188 [8Apr20]** | | | | | | | | | | | | | |  |
|  |  |  | No |  | All articles |  | 0.1981 |  | 1 | |  | | | | | |  |
|  | | |  |  |  |  |  |  |  | |  | | | | | | |
|  | | | | | | | | | | | | 1 |  | LF |  | 17/02/2021 11:19 |  |
|  | Cancer Research UK says the fight against cancer could be set back by several years due to a dramatic fall in income during the coronavirus pandemic. The leading charity is cutting research funding by 44m because it expects income to drop by up to 25% as a result of the crisis. See related Will China be sued over coronavirus?  [2] Are countries being honest about coronavirus?[3] Iain Foulkes, executive director of research and innovation, explained that the charity funds nearly 50% of cancer research in the UK, and that 'making cuts to research funding is the most difficult decision we have had to make'. He added: 'We are hopeful that limiting our spending now will enable us to continue funding life-saving research in the long run.' The charity told The Guardian[4] the impact of the new coronavirus has been significant, saying its 'shops had closed, mass fundraising events had stopped and legacies had reduced'. This has led to a reduction of around 120 million in income. | | | | | | | | | | | | | | | |  |
|  |  |  |  |  |  |  |  |  |  |  |  |  |  |  |  |  |  |
|  |  | | | | | | | | | | | | | | | |  |
|  | | | **Files\\249 [10May20]** | | | | | | | | | | | | | |  |
|  |  |  | No |  | All articles |  | 0.1584 |  | 1 | |  | | | | | |  |
|  | | |  |  |  |  |  |  |  | |  | | | | | | |
|  | | | | | | | | | | | | 1 |  | LF |  | 17/02/2021 15:00 |  |
|  | COVID-19 has also dealt a severe blow to America's cancer research non-profits. The Lymphoma Research Foundation has postponed all in-person programevents, including free patient and professional education programs and fundraising events, at least until this summer, to protect the patients who attend these events and who are at higher risk for severe complications from the novel coronavirus. With hospitals canceling non-essential visits, cancer patients will rely on organizations like the Lymphoma Research Foundation to provide information about how to manage their disease. And since cancer patients are particularly vulnerable to COVID-19, they'll look to non-profit organizations like LRF for support and ways to stay healthy throughout the pandemic. | | | | | | | | | | | | | | | |  |
|  |  | | | | | | | | | | | | | | | |  |
|  | | | **Files\\282 [8Dec20]** | | | | | | | | | | | | | |  |
|  |  |  | No |  | All articles |  | 0.2210 |  | 1 | |  | | | | | |  |
|  | | |  |  |  |  |  |  |  | |  | | | | | | |
|  | | | | | | | | | | | | 1 |  | LF |  | 17/02/2021 17:00 |  |
|  | CR UK has warned that it could be spending 150 million less per year by 2024 as it faces a potential 300 million decline in fundraising income over the next three years.'COVID-19 has slowed down our efforts to beat cancer.  The closures of our charity shops and the cancellation of our fundraising events across the country means we have less money available for life-saving research, but we will never stop,' said Michelle Mitchell, the charity's chief executive.'We have always relied on the generous donations of all our supporters, but we need them now more than ever so we can continue to achieve these ambitions and so that together, we can still beat cancer.'Dr | | | | | | | | | | | | | | | |  |
|  |  | | | | | | | | | | | | | | | |  |
|  | | | **Files\\299 [24Jun20]** | | | | | | | | | | | | | |  |
|  |  |  | No |  | All articles |  | 0.1828 |  | 1 | |  | | | | | |  |
|  | | |  |  |  |  |  |  |  | |  | | | | | | |
|  | | | | | | | | | | | | 1 |  | LF |  | 19/02/2021 15:01 |  |
|  | The BHF has warned that the impact of the pandemic means that its net income, and resulting investment in new research, is likely to drop by up to 50% this year, from 100 million to around 50 million.Such a sharp fall 'could have a catastrophic impact on UK cardiovascular research, the research careers of thousands of young scientists, and advances in diagnostics, treatments and cures for people with heart and circulatory diseases', it said.The charity funds over half of non-commercial research into heart and circulatory diseases in the UK, but the loss of income from shop closures and cancellation of fundraising events 'has created the biggest crisis in its 60-year history'.Furthermore, it could take several years for funding to return to pre-pandemic levels, the charity warned.'Ultimately | | | | | | | | | | | | | | | |  |
|  |  | | | | | | | | | | | | | | | |  |
| Reports\\Coding Summary By Code Report | | | | | | | | | | Page 11 of 123 | | | | | | | |
| 14/07/2021 10:54 | | | | | | | | | | | | | | | | | |
|  | | | **Aggregate** |  | **Classification** |  | **Coverage** |  | **Number Of Coding References** | |  | **Reference Number** |  | **Coded By Initials** |  | **Modified On** |  |
|  | | | **Files\\602 [16Oct20]** | | | | | | | | | | | | | |  |
|  |  |  | No |  | All articles |  | 0.4619 |  | 1 | |  | | | | | |  |
|  | | |  |  |  |  |  |  |  | |  | | | | | | |
|  | | | | | | | | | | | | 1 |  | LF |  | 08/03/2021 15:31 |  |
|  | Peter has rarely appeared on TV screens since 2017, when he cancelled his long-awaited stand-up tour for family reasons.The 'Phoenix Nights' star said at the time: "My family must always come first."And earlier this year, he was forced to postpone his much-anticipated Dance For Life comeback events due to the coronavirus pandemic.Announcing the news in March, he said: "Obviously this is disappointing news but well and truly justified given the circumstances."We'll get through this, together and then we'll all have a great big dance for life.  Stay safe and look after yourselves and the people around you."The popular comedian had originally planned to stage six shows in Liverpool, London and Manchester in April and May this year.The dance-a-thon events were being organised in aid of Cancer Research UK and would have been his first live performances since he cancelled his stand-up tour. | | | | | | | | | | | | | | | |  |
|  |  |  |  |  |  |  |  |  |  |  |  |  |  |  |  |  |  |
|  |  | | | | | | | | | | | | | | | |  |
|  | | | **Files\\611 [6May20]** | | | | | | | | | | | | | |  |
|  |  |  | No |  | All articles |  | 0.2626 |  | 1 | |  | | | | | |  |
|  | | |  |  |  |  |  |  |  | |  | | | | | | |
|  | | | | | | | | | | | | 1 |  | LF |  | 08/03/2021 15:32 |  |
|  | "Peter was set to perform in Manchester on April 10 and 11, with subsequent shows announced for Liverpool (May 8 and 9) and London (29 and 30).His representatives - who said the decision followed government advice to avoid "mass gatherings" - confirmed tickets will remain valid for rescheduled dates.They commented: "Due to the ongoing coronavirus outbreak and following Government advice, it is with deep regret that the forthcoming Peter Kay's 'Dance For Life' shows are being postponed."We are looking to schedule the shows so please hold onto your tickets as they will be valid for the new dates."We apologise for any inconvenience this may cause but the safety of the public is paramount." | | | | | | | | | | | | | | | |  |
|  |  | | | | | | | | | | | | | | | |  |
|  | | **PDF** | | | | | | | | | | | | | | |  |
|  | | | **Files\\101 [16 July 20]** | | | | | | | | | | | | | |  |
|  |  |  | No |  | All articles |  | 0.0824 |  | 1 | |  | | | | | |  |
|  | | |  |  |  |  |  |  |  | |  | | | | | | |
|  | | | | | | | | | | | | 1 |  | LF |  | 15/02/2021 12:23 |  |
|  | “We’re living through a global crisis unlike any other and, as it’s unfolded, it’s become clear that there’ll be a huge economic impact for years to come,” said Michelle Mitchell, CRUK chief executive.  As the UK went into lockdown in March, charity shops were closed, fundraising events were cancelled, and donations were diverted to National Health Service charities. All sta~ have gone to 80 per cent pay. | | | | | | | | | | | | | | | |  |
|  |  | | | | | | | | | | | | | | | |  |
|  | | | **Files\\108 (2) [no date]** | | | | | | | | | | | | | |  |
|  |  |  | No |  | All articles |  | 0.0532 |  | 1 | |  | | | | | |  |
|  | | |  |  |  |  |  |  |  | |  | | | | | | |
|  | | | | | | | | | | | | 1 |  | LF |  | 15/02/2021 13:03 |  |
|  | Some traditional Paint the Town Pink events have been cancelled for 2021 due to the coronavirus pandemic. Paint the Town Pink has raised over $2 million for cancer research at The Hormel Institute since 2011. | | | | | | | | | | | | | | | |  |
|  |  | | | | | | | | | | | | | | | |  |
|  | | | | | | | | | | | | | | | | | |
| Reports\\Coding Summary By Code Report | | | | | | | | | | Page 12 of 123 | | | | | | | |
| 14/07/2021 10:54 | | | | | | | | | | | | | | | | | |
|  | | | **Aggregate** |  | **Classification** |  | **Coverage** |  | **Number Of Coding References** | |  | **Reference Number** |  | **Coded By Initials** |  | **Modified On** |  |
|  | | | **Files\\111 [19 Jan 21]** | | | | | | | | | | | | | |  |
|  |  |  | No |  | All articles |  | 0.3813 |  | 1 | |  | | | | | |  |
|  | | |  |  |  |  |  |  |  | |  | | | | | | |
|  | | | | | | | | | | | | 1 |  | LF |  | 15/02/2021 13:11 |  |
|  | Despite the virus and the restrictions, Austin is still finding a way to Paint the Town Pink this year. But, it will look a little different.  "Our organizers recognize that COVID has been a huge strain on our local small businesses, which give and give and give to Paint the Town Pink every year," Director of Paint the Town Pink, Kathi Finley, said.  Plunging for Pink and Fishing for a Cure have been canceled, but the Radio-Thon and a couple of other events will go on as planned.  All of the fundraisers and events typically lead up to the Austin Bruins Paint the Rink Pink Game.  "What started out as just that one year and that one hockey game turned into 'well how can we make this into a winter festival for our community?,'" Finley said.  1/3  That game on the pink ice is one of the biggest revenue drivers of the whole event. From a sold-out arena to the specially made jerseys that are auctioned off after the game, all of the money goes to the Hormel Institute.  Since they are not allowed to pack the arena, the Bruins decided to postpone the game. Because for them, it's more than just a game.  "The whole town gets behind it, neighboring communities get behind it. We raise a lot of money for the Hormel Institute and cancer research and we're just hoping we can have it so that's why we keep pushing it back," Bruins Head Coach, Steve Howard, said.  "I mean the atmosphere is just unreal. You don't really see 2,000 fans in your average game. Just being able to play in front of all those people, rooting you on. It's something special, especially for the message it sends and everything," Bruins player, Cullen Rush, said.  The game is postponed until the spring when it will hopefully be safer to have more fans. | | | | | | | | | | | | | | | |  |
|  |  |  |  |  |  |  |  |  |  |  |  |  |  |  |  |  |  |
|  |  |  |  |  |  |  |  |  |  |  |  |  |  |  |  |  |  |
|  |  | | | | | | | | | | | | | | | |  |
|  | | | **Files\\127 (2) [no date]** | | | | | | | | | | | | | |  |
|  |  |  | No |  | All articles |  | 0.1211 |  | 1 | |  | | | | | |  |
|  | | |  |  |  |  |  |  |  | |  | | | | | | |
|  | | | | | | | | | | | | 1 |  | LF |  | 15/02/2021 16:51 |  |
|  | Chapman said the Eagles Cancer Telethon is about supporting its motto, “Together we can make a difference,” and raising funds for cancer research. In a time marked by division, that sense of community spirit and coming together over something bigger than any one individual is more important than ever, she said.  The money raised through the telethon will go to the Mayo Clinic Cancer Center, the Hormel Institute at the University of Minnesota, and the Masonic Cancer Center at the University of Minnesota.  This year, usual methods of raising money, such as an in-person auction, the kickoff diner, and other in-person fundraisers have been canceled. That means the telethon needs even more help to meet its fundraising goals. Ninety percent of the funds raised by the  2/3  telethon go directly to cancer research because of all the volunteer support the telethon receives. | | | | | | | | | | | | | | | |  |
|  |  |  |  |  |  |  |  |  |  |  |  |  |  |  |  |  |  |
|  |  | | | | | | | | | | | | | | | |  |
|  | | | **Files\\130 [11 Jan 21]** | | | | | | | | | | | | | |  |
|  |  |  | No |  | All articles |  | 0.0143 |  | 1 | |  | | | | | |  |
|  | | |  |  |  |  |  |  |  | |  | | | | | | |
|  | | | | | | | | | | | | 1 |  | LF |  | 15/02/2021 17:00 |  |
|  | Race for Life events across Leicestershire and the rest of the country were cancelled in 2020, due to the coronavirus pandemic. | | | | | | | | | | | | | | | |  |
|  |  | | | | | | | | | | | | | | | |  |
|  | | | **Files\\26 (2) [no date]** | | | | | | | | | | | | | |  |
|  |  |  | No |  | All articles |  | 0.0491 |  | 1 | |  | | | | | |  |
|  | | |  |  |  |  |  |  |  | |  | | | | | | |
|  | | | | | | | | | | | | 1 |  | LF |  | 17/02/2021 15:31 |  |
|  | Some of our fundraising events have been disrupted. If you were due to take part in the following events on behalf of the ICR, please contact sports@icr.ac.uk (mailto:sports@icr.ac.uk). We also suggest that people visit the official websites for any events for the latest updates. Vitality London 10K will not go ahead in its usual format (https://www.vitalitylondon10000.co.uk/news-media/latest-news/2020-vitality-london-10kimportant-update/index.html). | | | | | | | | | | | | | | | |  |
|  |  | | | | | | | | | | | | | | | |  |
| Reports\\Coding Summary By Code Report | | | | | | | | | | Page 13 of 123 | | | | | | | |
| 14/07/2021 10:54 | | | | | | | | | | | | | | | | | |
|  | | | **Aggregate** |  | **Classification** |  | **Coverage** |  | **Number Of Coding References** | |  | **Reference Number** |  | **Coded By Initials** |  | **Modified On** |  |
|  | | | **Files\\75 [4Aug20]** | | | | | | | | | | | | | |  |
|  |  |  | No |  | All articles |  | 0.0393 |  | 1 | |  | | | | | |  |
|  | | |  |  |  |  |  |  |  | |  | | | | | | |
|  | | | | | | | | | | | | 1 |  | LF |  | 08/03/2021 17:33 |  |
|  | Progress in cancer research may be delayed due to the impact of Covid-19 on charity fundraising, some experts have warned.  Large-scale fundraising events like the annual Race for Life had to be cancelled when the pandemic hit.  p  That meant Cancer Research UK, which funds many studies and trials in Belfast, immediately lost out on £44m. | | | | | | | | | | | | | | | |  |
|  |  | | | | | | | | | | | | | | | |  |
|  | **Nodes\\Direct impacts\Availability of resources for cancer research\Extreme funding shortfall from NGOs\Some fundraising events continued** | | | | | | | | | | | | | | | |  |
|  | | **Document** | | | | | | | | | | | | | | |  |
|  | | | **Files\\221 [12Aug20]** | | | | | | | | | | | | | |  |
|  |  |  | No |  | All articles |  | 0.3729 |  | 1 | |  | | | | | |  |
|  | | |  |  |  |  |  |  |  | |  | | | | | | |
|  | | | | | | | | | | | | 1 |  | LF |  | 17/02/2021 14:16 |  |
|  | You don't have to be the next Lew Alcindor (Kareem Abdul-Jabbar) to team up with the UCLA Athletic Department and Bruin Basketball this October. These acclaimed college athletes are urging college basketball fans and hoops-players of all ages and levels to join them and the not-for-profit Pediatric Cancer Research Foundation (PCRF) in a one-of-a kind challenge to raise crucial funding for pediatric cancer research. Fans from Los Angeles to Larchmont and Laredo to Laramie are encouraged to join the 13th Annual Dribble for the Cure, taking place October 11, in support of the Cancer Research Program at UCLA Mattel Children's Hospital.Participation is as fun, easy and purposeful as it sounds.  All it takes is a commitment to dribble a basketball - no endurance training or time trials required. This year, for the first time, the Dribble for the Cure is accessible via a newly created fitness tracker mobile app, called the PCRF Dribble App powered by MoveSpring. It allows participants to dribble on their own while following a course map that will take them throughout the UCLA campus. Organizers hope big-hearted basketball enthusiasts will donate their time to help supplement dwindling research dollars. Every $50 raised supports an hour of research and every $25,000 raised makes it possible for a child to participate in a clinical trial that could lead to therapeutic breakthroughs. One hundred percent (100%) of all fundraising dollars will go straight to scientific research.'If it takes a village to raise a child, it takes cities and communities of caring people to help effectively treat childhood cancer,' said Tyus Edney, Director of Engagement at UCLA Athletics and former UCLA Basketball Coach. 'We need families, teenagers, and folks from across the country to take part in this year's Dribble for the Cure to make it possible for children with cancer to get the care they need. Your involvement is critical!''This is an activity everyone can get behind, even while social distancing. | | | | | | | | | | | | | | | |  |
|  |  |  |  |  |  |  |  |  |  |  |  |  |  |  |  |  |  |
|  |  |  |  |  |  |  |  |  |  |  |  |  |  |  |  |  |  |
|  |  | | | | | | | | | | | | | | | |  |
|  | | | | | | | | | | | | | | | | | |
|  | | | | | | | | | | | | | | | | | |
|  | | | | | | | | | | | | | | | | | |
| Reports\\Coding Summary By Code Report | | | | | | | | | | Page 14 of 123 | | | | | | | |
| 14/07/2021 10:54 | | | | | | | | | | | | | | | | | |
|  | | | **Aggregate** |  | **Classification** |  | **Coverage** |  | **Number Of Coding References** | |  | **Reference Number** |  | **Coded By Initials** |  | **Modified On** |  |
|  | | | **Files\\270 [23Oct20]** | | | | | | | | | | | | | |  |
|  |  |  | No |  | All articles |  | 0.2874 |  | 1 | |  | | | | | |  |
|  | | |  |  |  |  |  |  |  | |  | | | | | | |
|  | | | | | | | | | | | | 1 |  | LF |  | 17/02/2021 15:39 |  |
|  | Be the Difference Foundation is proud to present the second annual Runway for Hope fashion show in a brand new, virtual format. On Thursday, November 18 at 12:00 noon, this event will bring together fashion influencers and ovarian cancer survivor models to benefit Mary Crowley Cancer Research.While COVID-19 may have paused many aspects of life, it unfortunately has not stopped cancer. Women are still getting diagnosed with ovarian cancer, still undergoing treatment and trying to survive daily life on top of being immunocompromised.  Throughout the pandemic, Be the Difference Foundation has remained devoted to improving outcomes for ovarian cancer patients announcing new support initiatives, pivoting their events to safer environments, and ensuring that women get the opportunity to enroll in a clinical trial.Runway for Hope provides a platform to raise awareness of ovarian cancer while also raising vital funds for the latest in ovarian cancer research. Over 22,000 women are diagnosed with ovarian cancer yearly and more than 50% will die within five years of diagnosis. Be the Difference Foundation has committed to changing the outcome for those diagnosed, putting a focus on clinical trials to find new treatment options for the patients that desperately need them by generously supporting Mary Crowley Cancer Research. The two organizations collaborated on the sold-out 2019 Runway for Hope event, as well as the Caring for Her initiative to help bring support resources to ovarian cancer patients enrolled in clinical trials.This year's Runway for Hope fashion show is transformed into an exciting virtual event featuring four models and four fashion influencers. | | | | | | | | | | | | | | | |  |
|  |  |  |  |  |  |  |  |  |  |  |  |  |  |  |  |  |  |
|  |  | | | | | | | | | | | | | | | |  |
|  | | | **Files\\375 [10Oct20]** | | | | | | | | | | | | | |  |
|  |  |  | No |  | All articles |  | 0.0913 |  | 1 | |  | | | | | |  |
|  | | |  |  |  |  |  |  |  | |  | | | | | | |
|  | | | | | | | | | | | | 1 |  | LF |  | 23/02/2021 15:35 |  |
|  | Tanger Outlets has launched its 27th annual Tanger Pink Campaign to support breast cancer research. The outlet centers will offer shoppers savings during the campaign.Tanger has been involved in the fight against breast cancer for the last 27 years and this year is no exception, even amid the pandemic. | | | | | | | | | | | | | | | |  |
|  |  | | | | | | | | | | | | | | | |  |
|  | | | **Files\\502 [5Sept20]** | | | | | | | | | | | | | |  |
|  |  |  | No |  | All articles |  | 0.2478 |  | 1 | |  | | | | | |  |
|  | | |  |  |  |  |  |  |  | |  | | | | | | |
|  | | | | | | | | | | | | 1 |  | LF |  | 08/03/2021 11:21 |  |
|  | The St. John's men's and women's basketball programs are set to host the 10th annual Dribble for the Cure, which will be held virtually for the first time, on Saturday, Sept. 26. All proceeds will support scientific research conducted by the Pediatric Cancer Research Foundation (PCRF) Laboratory at New York Medical College.Due to the ongoing effects of the COVID-19 pandemic, the charitable event will take place through the PCRF Dribble App powered by MoveSpring.  The new format features a five-day activity challenge from Sept. 21-25 that leads up to the official Dribble for the Cure on Sept. 26.'We look forward to this year's Dribble for the Cure, which has been a tremendous community service event for our basketball program over the past decade,' said St. John's Head Coach Mike Anderson. 'Dribble for the Cure has helped raise great awareness and funds for children's cancer research, contributing to an increase in survival rates. While this year's event will be different than any of its predecessors, we look forward to seeing everyone join us virtually to support such a worthy cause.'Through the newly created fitness tracker PCRF Dribble App, participants will dribble a basketball at their own pace following a course map that will take them throughout the St. John's campus. The PCRF Dribble App will offer live streaming content and unlockable milestones, as well as special messages from teams, coaches and sponsors throughout the event.'We | | | | | | | | | | | | | | | |  |
|  |  |  |  |  |  |  |  |  |  |  |  |  |  |  |  |  |  |
|  |  | | | | | | | | | | | | | | | |  |
|  | | | | | | | | | | | | | | | | | |
|  | | | | | | | | | | | | | | | | | |
| Reports\\Coding Summary By Code Report | | | | | | | | | | Page 15 of 123 | | | | | | | |
| 14/07/2021 10:54 | | | | | | | | | | | | | | | | | |
|  | | | **Aggregate** |  | **Classification** |  | **Coverage** |  | **Number Of Coding References** | |  | **Reference Number** |  | **Coded By Initials** |  | **Modified On** |  |
|  | | | **Files\\531 [14Jun20]** | | | | | | | | | | | | | |  |
|  |  |  | No |  | All articles |  | 0.6737 |  | 1 | |  | | | | | |  |
|  | | |  |  |  |  |  |  |  | |  | | | | | | |
|  | | | | | | | | | | | | 1 |  | LF |  | 08/03/2021 14:33 |  |
|  | People in the investment industry, as well as media and events, are taking stock of what can be learned and retained from how we coped with COVID-19 so far - as has been going on in most other industries. Perhaps the best-case study for very big events was provided by the organisers of Mothers Day Classic last week (June 9). The Mothers Day Classic, in its 23rd year, has raised $37 million for breast cancer research. The event, involving co-ordinated fun runs, walks, and other activities involving various groups of superannuation and investment industry, family, friends and even pets, traditionally attracted up to 100,000 people at about 100 locations around Australia.  https://ioandc.com/wp-content/uploads/2020/06/5-Waffles-the-rabbit-has-raised-800-and-counting.jpg Waffles the rabbit has raised $800 and counting It was to be held on Mothers Day, as always, which this year was on May 10, but Australia was still in lock-down. We were still allowed to exercise and have fun at home, though, and that's exactly what the Mothers Day Classic Foundation (MDC) managed to adjust to in very quick time. It managed to sign up more than 50,000 people for the 'event', which included an important build-up campaign. It also retained all of its major sponsors. MDC, in a sense, turned the one-day event into a celebration, raising money over about two months. Donations are still being accepted up to June 30.   https://ioandc.com/wp-content/uploads/2020/06/5-Great-energy-...-and-photo.jpg Great energy and photo Zara Lawless, the MDC chief executive, who joined the organisation only six months earlier, examined what was achieved under trying circumstances, presenting lots of ideas for the future in a webinar presented by Women in Super (WIS) on June 9 as part of the WIS 'State of Play' series. Sandra Buckley, the WIS chief executive, said MDC was the first national event to commit to continue as a virtual event. And it changed tack in just 48 hours. WIS founded MDC, under the WIS founder, the late Mavis Robertson, together with Louise Davidson, in 1998, and remains its major supporter. 'For us, cancellation was never an option,' Lawless said. 'Less than two months out, by mid-March, a lot of work had already been done on the [traditional]event. You can imagine, something of that size and complexity takes six-eight months to produce. Then at the 'pivot point' decisiveness was essential. A lot of events were looking at whether to cancel or postpone. We decided within 48 hours of the first warnings. We had to provide clear communications. For instance, we already had people preparing signage for all the locations. We had to articulate the 'North Star'.'   https://ioandc.com/wp-content/uploads/2020/06/5-MDC-supporters-on-NSW-South-Coast.jpg MDC supporters on NSW South Coast She said MDC had to engage with and respect all the feedback and complaints it would get. She advised people in that position to not be afraid to ask for help. Partnerships were vital, especially with the media, including television and radio and corporate partners. 'You embrace the uncertainty and be empowered by it, allowing innovation and creativity to flourish.' Post the decision to pivot, the key principles of corporate success held truer than ever,' Lawless said. A lot had to change, requiring accountability and clarity of roles. 'We had to have value over activity. We had to recognise 'busy work' and stop it. MDC could not afford to waste any time. It was excellence over perfection. We needed to challenge decisions by encouraging respectful robust debate.' MDC managed to double its usual number of media mentions over the course of the campaign because of its 'fantastic new story to tell'. Lawless said: 'We have a fantastic media manager and I think I spoke to nearly every ABC regional radio station. We became a bit addicted to our Facebook page too. Some of the best stories were posted there, logging people's virtual progress. People were getting out and about in pink gear everywhere.' It became a virtual platform curating content that was engaging, inspiring and informative for the exclusive use of participants. 'One of my favourite comments was people saying: 'it felt like an event'. People dressed up in incredible outfits.' | | | | | | | | | | | | | | | |  |
|  |  |  |  |  |  |  |  |  |  |  |  |  |  |  |  |  |  |
|  |  |  |  |  |  |  |  |  |  |  |  |  |  |  |  |  |  |
|  |  |  |  |  |  |  |  |  |  |  |  |  |  |  |  |  |  |
|  |  |  |  |  |  |  |  |  |  |  |  |  |  |  |  |  |  |
|  |  | | | | | | | | | | | | | | | |  |
|  | | | **Files\\540 [14Jul20]** | | | | | | | | | | | | | |  |
|  |  |  | No |  | All articles |  | 0.2998 |  | 1 | |  | | | | | |  |
|  | | |  |  |  |  |  |  |  | |  | | | | | | |
|  | | | | | | | | | | | | 1 |  | LF |  | 08/03/2021 14:41 |  |
|  | HomeMany charities host social events to raise awareness for their causes, to increase visibility and to raise funding for their needs. Undoubtedly, participation in these social events breeds a community spirit and with many in-person events rescheduled or cancelled to adhere to social distancing rules and prevent the spread of COVID-19, innovative charities are finding ways to volunteer from home. Through active participation in digital volunteer opportunities, you can foster a sense of community with other volunteers and supporters of other worthy causes. Involved with Cancer Research UK, Horn points out the organization's shift[3] to digital volunteer methods throughout the pandemic. While the organization regularly accepts volunteers for a myriad of in-person opportunities, these opportunities have pivoted to a digital model. To support the charity's life-saving research and cancer treatment goals, Horn suggests partaking in one of the organization's digital volunteer opportunities. In light of the growing popularity of video calls for bonding with friends, family, and loved ones, the organization has created a digital quiz named The Big Quiz for Cancer Research UK. Volunteers can find all of the needed materials online for a video call that marries fun, communication and safety awareness. Additionally, Joey Horn points to the organization's e-campaigner option, which allows interested parties to participate in digital letter writing campaigns, awareness building, and other digitally based activities to share your voice, alongside a dedicated community of thousands of other volunteers.Get CreativeIn the spirit of raising awareness for a cause that's near to your heart and bringing like-minded people together to share in your goal, many charities are welcoming personalized fundraising options and are inviting innovative involvement from interested parties. This ability to simultaneously help an important cause, organize a digitally based event, and participate in a communicative opportunity with a group of people is an excellent way to feel connected during self-isolation. | | | | | | | | | | | | | | | |  |
|  |  |  |  |  |  |  |  |  |  |  |  |  |  |  |  |  |  |
|  |  |  |  |  |  |  |  |  |  |  |  |  |  |  |  |  |  |
|  |  | | | | | | | | | | | | | | | |  |
| Reports\\Coding Summary By Code Report | | | | | | | | | | Page 16 of 123 | | | | | | | |
| 14/07/2021 10:54 | | | | | | | | | | | | | | | | | |
|  | | | **Aggregate** |  | **Classification** |  | **Coverage** |  | **Number Of Coding References** | |  | **Reference Number** |  | **Coded By Initials** |  | **Modified On** |  |
|  | | | **Files\\550 [7Sept20]** | | | | | | | | | | | | | |  |
|  |  |  | No |  | All articles |  | 0.2835 |  | 1 | |  | | | | | |  |
|  | | |  |  |  |  |  |  |  | |  | | | | | | |
|  | | | | | | | | | | | | 1 |  | LF |  | 08/03/2021 15:04 |  |
|  | The Colourkast collection is available across all the company's Cian baths and basins and Satin Rose has proven to be one of the most popular colour choices out of the eight options available. Sally Cutchie, Marketing Manager at BC Designs, says: 'Unfortunately, one of the many negatives of Covid 19 is that charity donations have significantly fallen. 'As a company we wanted to be able to commit to making donations to vital research and felt Breast Cancer Research was a worthy cause. Not only will our team 'wear it pink' in October but hopefully homes up and down the country will too!'Cian | | | | | | | | | | | | | | | |  |
|  |  | | | | | | | | | | | | | | | |  |
|  | | | **Files\\576 [17Jun20]** | | | | | | | | | | | | | |  |
|  |  |  | No |  | All articles |  | 0.2897 |  | 1 | |  | | | | | |  |
|  | | |  |  |  |  |  |  |  | |  | | | | | | |
|  | | | | | | | | | | | | 1 |  | LF |  | 08/03/2021 15:13 |  |
|  | Keanu Reeves is a man every woman loves. The man is known not just for his good looks and supreme acting talent but his heart of gold and his philanthropic ways.  In response to his sister's battle with leukemia Reeves founded a private cancer foundation, which aids children's[1] hospitals and provides cancer research. In his quest to do some good during this unpredictable time that's taken the attention away from people who need the love and care in a world currently fixated over coronavirus. The Matrix Revolution actor has committed to donating 15 minutes of his time as part of an online auction for Camp Rainbow Gold, Idaho-based children's cancer charity[2]. | | | | | | | | | | | | | | | |  |
|  |  | | | | | | | | | | | | | | | |  |
|  | | | **Files\\587 [8Jun20]** | | | | | | | | | | | | | |  |
|  |  |  | No |  | All articles |  | 0.6231 |  | 1 | |  | | | | | |  |
|  | | |  |  |  |  |  |  |  | |  | | | | | | |
|  | | | | | | | | | | | | 1 |  | LF |  | 08/03/2021 15:25 |  |
|  | The annual Australian Alternative Investment Awards, held in conjunction with Hedge Funds Rock, an important annual fund raiser for a variety of charities, will go ahead on, or near, the pre-ordained date of September 10. Given the crisis, this year's awards and the fund raiser are more important than ever. Kim Ivey, chairman of the Alternative Future Foundation which is managed by the Hedge Funds Rock organisation, one of the initial supporters of the fund raiser, a co-founder of what became AIMA Australia, and chair of the awards themselves, said last week: 'Make no mistake; Hedge Funds Rock 2020 will take place this year.'  How it takes place - either virtually or at an actual dinner at a big venue, as normal- and precisely when it takes place, are in the laps of the COVID-19 gods, or devils. Ivey provided the latest committee resolution to industry participants last week (see below). The analytics-providing partner, Mercer, and the major sponsors, are all on board. The awards night, with Hedge Funds Rock, is the biggest event of the year for the alternative funds management industry sector. Last year there were 420 attendees on the night. The annual AIMA Australia conference, held in the same week and which includes familiar faces in its organisation, attracts more than 300 attendees. In Australia, the second week in September is called 'hedge funds week' as many overseas participants at the two events wrap around meetings and their own functions to coincide. Ivey said last week: 'If ever you wanted to hold awards for the best performers in a market downturn, this would be the year to do so.' Getting the managers' performance figures in a relatively short period of time is not easy. Mercer and Lonsec are the repositories for the data and the committee members help in prompting the fund managers. Apparently, if your numbers are good, you don't take as much prompting. Hedge Funds Rock was started in 2002 by Caroline Dodds, who was the principal annual organiser for several years, with her main aim to raise money for cancer research. | | | | | | | | | | | | | | | |  |
|  |  |  |  |  |  |  |  |  |  |  |  |  |  |  |  |  |  |
|  |  |  |  |  |  |  |  |  |  |  |  |  |  |  |  |  |  |
|  |  | | | | | | | | | | | | | | | |  |
|  | | | | | | | | | | | | | | | | | |
|  | | | | | | | | | | | | | | | | | |
| Reports\\Coding Summary By Code Report | | | | | | | | | | Page 17 of 123 | | | | | | | |
| 14/07/2021 10:54 | | | | | | | | | | | | | | | | | |
|  | | | **Aggregate** |  | **Classification** |  | **Coverage** |  | **Number Of Coding References** | |  | **Reference Number** |  | **Coded By Initials** |  | **Modified On** |  |
|  | | | **Files\\588 [31May20]** | | | | | | | | | | | | | |  |
|  |  |  | No |  | All articles |  | 0.0822 |  | 1 | |  | | | | | |  |
|  | | |  |  |  |  |  |  |  | |  | | | | | | |
|  | | | | | | | | | | | | 1 |  | LF |  | 08/03/2021 15:26 |  |
|  | Stewart Kohl has been actively involved in a variety of community and philanthropic organizations, serving as an Honorary Trustee of Oberlin College and Co-Chair of the Museum of Contemporary Art Cleveland. He is also on the Board of Trustees of Cleveland Clinic and Co-Chairs its $2 billion Power of Every One Capital Centennial Campaign. One of Stewart's proudest accomplishments is helping to serve as a founder of VeloSano, a cycling-related charity that has raised more than $21 million to fund cancer research at the Cleveland Clinic since its start in 2014. This year, because of the COVID-19 health crisis, VeloSano[2] will be hosting a virtual 'Bike to Cure' weekend in July. | | | | | | | | | | | | | | | |  |
|  |  | | | | | | | | | | | | | | | |  |
|  | | **PDF** | | | | | | | | | | | | | | |  |
|  | | | **Files\\104 [1 Oct 20]** | | | | | | | | | | | | | |  |
|  |  |  | No |  | All articles |  | 0.2004 |  | 1 | |  | | | | | |  |
|  | | |  |  |  |  |  |  |  | |  | | | | | | |
|  | | | | | | | | | | | | 1 |  | LF |  | 15/02/2021 13:02 |  |
|  | The American Cancer Society has experienced major fundraising losses during the coronavirus pandemic.  "Donations are key right now," said Matheny. "We are possibly going to lose a decade of research because of COVID."  The group will hold COVID-friendly fundraisers this month, including Making Strides of Myrtle Beach. You're encouraged to walk on the beach on Oct. 17.  "We're asking people to be socially safe and wear their pink that day and get out," said Matheny. "Preferably walk on the beach since ours was the beach walk, but if you want to just walk in the neighborhood with your family and friends, you can. Be socially safe." | | | | | | | | | | | | | | | |  |
|  |  | | | | | | | | | | | | | | | |  |
|  | | | **Files\\108 (2) [no date]** | | | | | | | | | | | | | |  |
|  |  |  | No |  | All articles |  | 0.1258 |  | 1 | |  | | | | | |  |
|  | | |  |  |  |  |  |  |  | |  | | | | | | |
|  | | | | | | | | | | | | 1 |  | LF |  | 15/02/2021 13:03 |  |
|  | The annual Paint the Town Pink cancer research fundraiser is underway.  The 2021 effort to raise money for The Hormel Institute began Tuesday night with the Austin Area Chamber of Commerce’s January “Business After Hours” event.  “Despite this environment where businesses and people are still hurting from the effects of COVID-19, we want to celebrate the work that is being done thanks to PTTP and our generous community,” says Gail Dennison, Director of Development and Public Relations. | | | | | | | | | | | | | | | |  |
|  |  | | | | | | | | | | | | | | | |  |
|  | | | **Files\\125 (2) [8 Jan 21]** | | | | | | | | | | | | | |  |
|  |  |  | No |  | All articles |  | 0.1779 |  | 1 | |  | | | | | |  |
|  | | |  |  |  |  |  |  |  | |  | | | | | | |
|  | | | | | | | | | | | | 1 |  | LF |  | 15/02/2021 16:46 |  |
|  | Harborfields High School's 10th grade Student Government members donated $1,500 to the Breast Cancer Research Foundation in honor of Breast Cancer Awareness Month, which was in October, the district announced Thursday.  The team worked to design and sell children's and adult-sized t-shirts, long-sleeved shirts and sweatshirts across the district's schools, raising breast cancer awareness and a sense of community.  "I think it's something that is really impactful especially because there is so little we can do in this time, and it's so strange that we can't be together and gather," Student Government President Nahrahel Louis said. "We thought that this would be a great way to  1/4  bring the community spirit back by collectively coming together and supporting this one thing. It has truly been a team effort." | | | | | | | | | | | | | | | |  |
|  |  |  |  |  |  |  |  |  |  |  |  |  |  |  |  |  |  |
|  |  | | | | | | | | | | | | | | | |  |
| Reports\\Coding Summary By Code Report | | | | | | | | | | Page 18 of 123 | | | | | | | |
| 14/07/2021 10:54 | | | | | | | | | | | | | | | | | |
|  | | | **Aggregate** |  | **Classification** |  | **Coverage** |  | **Number Of Coding References** | |  | **Reference Number** |  | **Coded By Initials** |  | **Modified On** |  |
|  | | | **Files\\127 (2) [no date]** | | | | | | | | | | | | | |  |
|  |  |  | No |  | All articles |  | 0.2957 |  | 1 | |  | | | | | |  |
|  | | |  |  |  |  |  |  |  | |  | | | | | | |
|  | | | | | | | | | | | | 1 |  | LF |  | 15/02/2021 16:50 |  |
|  | Over the past 66 years, the Fifth District Eagles Cancer Telethon has donated more than $18 million to cancer research. The event claims the title of the longest locally run telethon in the U.S., and it’s going to take more than a pandemic to put a stop to that track record.  This Saturday, the telethon — with live hosts but previously recorded presentations and performances — will carry the title onward.  Teresa Chapman, executive director of the Eagles Cancer Telethon, has been involved with the event for the past 24 years.  In its long history, the telethon has seen many changes. In the beginning, everything was done with paper forms. There were no computers or social media, and donations were picked up in person.  Chapman said KTTC’s commitment to the telethon has been invaluable, turning the fundraiser into a 20-hour telethon hosting more than 125 performances each year.  1/3  A crew gets the stage in the Mayo Civic Center Auditorium ready for the telethon Saturday morning, Jan. 18, 2020, in Rochester. (Joe Ahlquist / jahlquist@postbulletin.com)  In a typical year, the telethon’s presentations and performances would last through the night, often into the wee hours of the morning. This year, all the talent will be recorded from past telethons, but hosts will still be live in the KTTC studios during the event. Chapman said the planning required “a lot of rethinking and changes” to make sure everyone would stay safe during the pandemic.  “COVID-19 is here,” she said, “but so is cancer.”  “If we were to just disappear, what would that have said to all those that have been touched by cancer?” she added.  Chapman said the Eagles Cancer Telethon is about supporting its motto, “Together we can make a difference,” and raising funds for cancer research. In a time marked by division, that sense of community spirit and coming together over something bigger than any one individual is more important than ever, she said.  The money raised through the telethon will go to the Mayo Clinic Cancer Center, the Hormel Institute at the University of Minnesota, and the Masonic Cancer Center at the University of Minnesota. | | | | | | | | | | | | | | | |  |
|  |  |  |  |  |  |  |  |  |  |  |  |  |  |  |  |  |  |
|  |  |  |  |  |  |  |  |  |  |  |  |  |  |  |  |  |  |
|  |  |  |  |  |  |  |  |  |  |  |  |  |  |  |  |  |  |
|  |  | | | | | | | | | | | | | | | |  |
|  | | | **Files\\130 [11 Jan 21]** | | | | | | | | | | | | | |  |
|  |  |  | No |  | All articles |  | 0.1308 |  | 1 | |  | | | | | |  |
|  | | |  |  |  |  |  |  |  | |  | | | | | | |
|  | | | | | | | | | | | | 1 |  | LF |  | 15/02/2021 16:59 |  |
|  | Race for Life events across Leicestershire and the rest of the country were cancelled in 2020, due to the coronavirus pandemic.  Safety concerns, and the difficulties around social distancing, meant the Cancer Research UK fund-raiser went remote, and participants were encouraged to take part from their homes.  The charity has now revealed its plans for the event this year. 1/5  Race for Life is returning this year, with social distancing measures in place to keep participants safe, said the charity.  It is now calling on men, women and children to enter a race. Anyone who signs up in January can claim 30 per cent off the entry fee by using the code JAN2021.  Race For Life at Victoria Park Leicester (Image: Peter Fothergill) Participants will set off on the course either alone or in small, socially-distanced groups.  Hand sanitiser will be provided at all events with participants encouraged to use it before and after the event.  And participants will be asked to avoid hugging on the finishing line.  The charity said that if any of the planned 2021 events had to be cancelled, people who had signed up "would be entitled to a refund". | | | | | | | | | | | | | | | |  |
|  |  |  |  |  |  |  |  |  |  |  |  |  |  |  |  |  |  |
|  |  | | | | | | | | | | | | | | | |  |
|  | | | **Files\\41 [14Dec20]** | | | | | | | | | | | | | |  |
|  |  |  | No |  | All articles |  | 0.0177 |  | 1 | |  | | | | | |  |
|  | | |  |  |  |  |  |  |  | |  | | | | | | |
|  | | | | | | | | | | | | 1 |  | LF |  | 07/03/2021 12:01 |  |
|  | David Miller, of Oakland, saw it that way when deciding to swim 1 million yards this year to raise money for cancer research. He reached the finish line Sunday afternoon by swimming 2,000 yards at the Soda Aquatic Center in Moraga, where Miller works as a lifeguard. | | | | | | | | | | | | | | | |  |
|  |  | | | | | | | | | | | | | | | |  |
|  | | | | | | | | | | | | | | | | | |
| Reports\\Coding Summary By Code Report | | | | | | | | | | Page 19 of 123 | | | | | | | |
| 14/07/2021 10:54 | | | | | | | | | | | | | | | | | |
|  | | | **Aggregate** |  | **Classification** |  | **Coverage** |  | **Number Of Coding References** | |  | **Reference Number** |  | **Coded By Initials** |  | **Modified On** |  |
|  | | | **Files\\82 [1Oct20]** | | | | | | | | | | | | | |  |
|  |  |  | No |  | All articles |  | 0.0351 |  | 1 | |  | | | | | |  |
|  | | |  |  |  |  |  |  |  | |  | | | | | | |
|  | | | | | | | | | | | | 1 |  | LF |  | 09/03/2021 09:39 |  |
|  | The Cure Starts Now’s "The Great Relay for Research" Raised Over $175,000 for Homerun Cure™ Cancer Research  Cincinnati, OH | October 1, 2020 — The Cure Starts Now’s ~rst annual “The Great Relay for Research” raised $179,105 for Homerun Cure™ cancer research surpassing their original goal by over 700 percent. | | | | | | | | | | | | | | | |  |
|  |  | | | | | | | | | | | | | | | |  |
|  | | | **Files\\98 [15Nov20]** | | | | | | | | | | | | | |  |
|  |  |  | No |  | All articles |  | 0.4484 |  | 1 | |  | | | | | |  |
|  | | |  |  |  |  |  |  |  | |  | | | | | | |
|  | | | | | | | | | | | | 1 |  | LF |  | 09/03/2021 10:55 |  |
|  | In a year when the COVID-19 pandemic has stalled fundraising efforts for myriad diseases, Jason Furlano is pounding the pavement.  The Ontario man ran a marathon-length distance, about 42 kilometres, every day last week in efforts to raise money for cancer research.  “Cancer didn’t take a break during the pandemic,” the advertising executive told CTV National News. “Cancer continues to plague us all.”  He hopes to complete 5,500 kilometres, the width of Canada, by the end of the year, a goal inspired by Terry Fox, who succumbed to cancer while on a cross-country “marathon of hope.” Furlano has already completed 4,700 kilometres, worn through 12 pairs of running shoes and raised more than $40,000 in donations.  The fundraising mission itself was inspired by the leukemia battle of his childhood friend’s wife, Jillian, who was diagnosed late last year when they were weeks away from getting married. She underwent chemotherapy, received a stem cell transplant, had a wedding ceremony at the Princess Margaret Cancer Centre and is now in remission.  “Seeing Jillian be so strong through that process -- that’s really what pushed me to do something, get out of my comfort zone, and really push my boundaries,” said Furlano, who wasn’t much of a runner before he began his lofty goal to raise money for the Princess Margaret Cancer Centre as a thank-you. He hopes to bring in another $60,000 by the end of the year.  1/2  “It’s amazing. He’s doing a wonderful job,” said Jillian about her friend’s fundraising efforts.  Furlano’s run has apparently inspired others to join “The DIY Challenge,” a movement benefiting the hospital where Jillian was treated. A site dedicated to the challenge has personal fundraising pages from people committed to fundraising efforts like knitting hats, baking bread, or like Furlano, running thousands of kilometres. Between 185 participants, the hospital has raised more than $165,000. | | | | | | | | | | | | | | | |  |
|  |  |  |  |  |  |  |  |  |  |  |  |  |  |  |  |  |  |
|  |  |  |  |  |  |  |  |  |  |  |  |  |  |  |  |  |  |
|  |  | | | | | | | | | | | | | | | |  |
|  | | | | | | | | | | | | | | | | | |
|  | | | | | | | | | | | | | | | | | |
|  | | | | | | | | | | | | | | | | | |
|  | | | | | | | | | | | | | | | | | |
| Reports\\Coding Summary By Code Report | | | | | | | | | | Page 20 of 123 | | | | | | | |
| 14/07/2021 10:54 | | | | | | | | | | | | | | | | | |
|  | | | **Aggregate** |  | **Classification** |  | **Coverage** |  | **Number Of Coding References** | |  | **Reference Number** |  | **Coded By Initials** |  | **Modified On** |  |
|  | **Nodes\\Direct impacts\Availability of resources for cancer research\Financial damage to charities** | | | | | | | | | | | | | | | |  |
|  | | **Document** | | | | | | | | | | | | | | |  |
|  | | | **Files\\563 [16July20]** | | | | | | | | | | | | | |  |
|  |  |  | Yes |  | All articles |  | 0.8687 |  | 1 | |  | | | | | |  |
|  | | |  |  |  |  |  |  |  | |  | | | | | | |
|  | | | | | | | | | | | | 1 |  | LF |  | 08/03/2021 15:08 |  |
|  | Cancer Research UK has developed plans to become a 'leaner, more focused organisation' in the wake of COVID-19's devastating impact on its fundraising income.The charity said it has is making 'difficult decisions' to significantly cut spend on beating cancer, its operations and staff numbers after an anticipated 300 million decline in fundraising income over the next three years.As it adapts financial challenges and a new environment amid the pandemic, the charity said it will have to stop some programmes of work, reduce the amount or scope of other activities, and will be cutting its workforce by 500 roles, (not including trading).The charity also plans to introduce a new research model designed to maximise impact from a lower level of spend, and will reduce its research spend to 250 million within four to five years - a cut of 150 million from what the charity had planned to spend.'We're living through a global crisis unlike any other and, as it's unfolded, it's become clear that there'll be a huge economic impact for years to come,' said Michelle Mitchell, chief executive at Cancer Research UK.'We made some very difficult decisions early on to mitigate the impact on our work; we moved all of our staff to 80% pay, furloughed 60% of staff, and cut 44 million from our research.  But it is with a heavy heart that I can confirm we will have to reduce the size of our workforce, and make significant cuts to our research spend, as a result of the situation we find ourselves in.'This plan sets the direction for a new phase in the life of Cancer Research UK and will help us respond to the changed world, quicker than we've ever done before. We will emerge a streamlined charity, but still with a resolute drive for impact. Together, we will still beat cancer and realise our ambition to improve cancer survival to 3 in 4 by 2034.' | | | | | | | | | | | | | | | |  |
|  |  |  |  |  |  |  |  |  |  |  |  |  |  |  |  |  |  |
|  |  |  |  |  |  |  |  |  |  |  |  |  |  |  |  |  |  |
|  |  | | | | | | | | | | | | | | | |  |
|  | **Nodes\\Direct impacts\Availability of resources for cancer research\Increased reliance on public spending** | | | | | | | | | | | | | | | |  |
|  | | **Document** | | | | | | | | | | | | | | |  |
|  | | | **Files\\25 [30Nov20]** | | | | | | | | | | | | | |  |
|  |  |  | No |  | All articles |  | 0.1783 |  | 2 | |  | | | | | |  |
|  | | |  |  |  |  |  |  |  | |  | | | | | | |
|  | | | | | | | | | | | | 1 |  | LF |  | 17/02/2021 15:02 |  |
|  | Cancer researchers fear advances for patients could be delayed by almost a year and a half because of the effects of the COVID-19 pandemic, a new survey reveals. Scientists at The Institute of Cancer Research, London, told the survey that their own research advances would be pushed back by an average of six months by the initial lockdown, subsequent restrictions on laboratory capacity and the closure of national scientific facilities. With broader effects on charity funding, disruption of collaboration and personal interaction between scientists, and diversion of research efforts to COVID-19, the respondents estimated that major advances in cancer research[1] would be delayed by an average of 17 months. But the researchers said science had now adapted in many ways to the pandemic and that long-lasting damage to cancer research could be mitigated through extra funding from charitable donations or Government support—calling for investment in staffing, new technology such as robotics and computing power. | | | | | | | | | | | | | | | |  |
|  |  |  |  |  |  |  |  |  |  |  |  |  |  |  |  |  |  |
|  |  | | | | | | | | | | | | | | | |  |
|  | | | | | | | | | | | | 2 |  | LF |  | 17/02/2021 15:09 |  |
|  | "Our researchers are passionate about making advances to benefit patients, so it has been hugely frustrating that their work has been so disrupted, although also inspiring to see how well they have adapted to the restrictions the pandemic has imposed on our lives. "It is sobering to see that our researchers are estimating that their own research advances will be delayed by six months—and that the wider impact, because of the interconnectedness of science, is likely to push back major advances for patients by nearly a year and a half. "Our survey though does provide solutions to mitigate the impact—in the form of investment in staffing, new technologies and computing power. For that, we need more of the generous donations we have been receiving to our emergency appeal, along with a commitment from the Government to help fill the funding gap for the life sciences left by the pandemic." | | | | | | | | | | | | | | | |  |
|  |  |  |  |  |  |  |  |  |  |  |  |  |  |  |  |  |  |
|  |  | | | | | | | | | | | | | | | |  |
| Reports\\Coding Summary By Code Report | | | | | | | | | | Page 21 of 123 | | | | | | | |
| 14/07/2021 10:54 | | | | | | | | | | | | | | | | | |
|  | | | **Aggregate** |  | **Classification** |  | **Coverage** |  | **Number Of Coding References** | |  | **Reference Number** |  | **Coded By Initials** |  | **Modified On** |  |
|  | | **PDF** | | | | | | | | | | | | | | |  |
|  | | | **Files\\10 [21 Oct 20]** | | | | | | | | | | | | | |  |
|  |  |  | No |  | All articles |  | 0.0243 |  | 1 | |  | | | | | |  |
|  | | |  |  |  |  |  |  |  | |  | | | | | | |
|  | | | | | | | | | | | | 1 |  | LF |  | 15/02/2021 12:26 |  |
|  | Cameron agreed, adding that public funds from increasingly cash-strapped governments needs to be upheld, since many advancements in treatments haven’t exclusively come from the commercial sector.  “There is clearly still a lot of work to do, and lots of insights we've yet to ﬁnd out and yet to discover,” he said.  But whether diﬀerent sectors band together and put forward the necessary funding for that research in the post-pandemic world is a question that lingers among patients like Hilton.  “If the whole world jumps because of Covid, then can't they do the same for cancer and stop it being such a debilitating disease?” Hilton said. | | | | | | | | | | | | | | | |  |
|  |  | | | | | | | | | | | | | | | |  |
|  | **Nodes\\Direct impacts\Availability of resources for cancer research\Increased reliance on public spending\Political pressure to provide emergency funding for health services** | | | | | | | | | | | | | | | |  |
|  | | **Document** | | | | | | | | | | | | | | |  |
|  | | | **Files\\271 [18Sept20]** | | | | | | | | | | | | | |  |
|  |  |  | No |  | All articles |  | 0.1342 |  | 1 | |  | | | | | |  |
|  | | |  |  |  |  |  |  |  | |  | | | | | | |
|  | | | | | | | | | | | | 1 |  | LF |  | 17/02/2021 15:56 |  |
|  | "The long-term impact delays in diagnosing lung cancer[4] could have on families across the UK is devastating," says Michelle Mitchell, Cancer Research UK's chief executive. "It's as important as ever that anyone with a new cough that's persisted for three weeks, a change to an existing cough or breathlessness, speaks to their GP as soon as possible." Mitchell added that it's also time for the Government to show its commitment to the NHS and its recovery. "In this autumn's Comprehensive Spending Review, further investment is urgently required to make sure the NHS has the staff and equipment it needs to clear the mounting backlog of patients and transform and improve cancer[5] services." | | | | | | | | | | | | | | | |  |
|  |  | | | | | | | | | | | | | | | |  |
|  | | | **Files\\512 [10Jun20]** | | | | | | | | | | | | | |  |
|  |  |  | No |  | All articles |  | 0.3723 |  | 1 | |  | | | | | |  |
|  | | |  |  |  |  |  |  |  | |  | | | | | | |
|  | | | | | | | | | | | | 1 |  | LF |  | 08/03/2021 14:12 |  |
|  | A primary challenge will be dealing with the huge backlog of treatment paused during the novel coronavirus pandemic, with the waiting list for routine procedures already overshooting 4 million 'now certain to rise significantly'.The warning follows that from the BHF that 28,000 inpatient heart procedures had been delayed during the outbreak, and that from Cancer Research UK that around 2.4 million people in the UK are currently waiting for cancer screening, tests or treatment.In a letter to the Prime Minister, the NHS Confederation warns 'it will not be possible to simply 'switch on' NHS services immediately', and calls on the government to manage expectations on how quickly key patient services can be restored.The body also calls for an extension to emergency NHS funding, establishing an ongoing arrangement with the private sector to clear the treatment backlog, and a commitment to acknowledge and address health inequalities through upcoming guidance and policy reform.'Political leaders have a vital role to play in reassuring the public that every step possible is being taken to manage the virus, while safely bringing back services that had to be paused. | | | | | | | | | | | | | | | |  |
|  |  |  |  |  |  |  |  |  |  |  |  |  |  |  |  |  |  |
|  |  | | | | | | | | | | | | | | | |  |
|  | | | | | | | | | | | | | | | | | |
| Reports\\Coding Summary By Code Report | | | | | | | | | | Page 22 of 123 | | | | | | | |
| 14/07/2021 10:54 | | | | | | | | | | | | | | | | | |
|  | | | **Aggregate** |  | **Classification** |  | **Coverage** |  | **Number Of Coding References** | |  | **Reference Number** |  | **Coded By Initials** |  | **Modified On** |  |
|  | **Nodes\\Direct impacts\Availability of resources for cancer research\Increased reliance on public spending\Political pressure to support NGOs** | | | | | | | | | | | | | | | |  |
|  | | **Document** | | | | | | | | | | | | | | |  |
|  | | | **Files\\249 [10May20]** | | | | | | | | | | | | | |  |
|  |  |  | No |  | All articles |  | 0.0698 |  | 1 | |  | | | | | |  |
|  | | |  |  |  |  |  |  |  | |  | | | | | | |
|  | | | | | | | | | | | | 1 |  | LF |  | 17/02/2021 15:01 |  |
|  | Lawmakers are right to provide economic relief to shuttered shops and restaurants. But they also need to assist the soup kitchens, social service organizations, cancer research foundations, and other groups that will support vulnerable Americans throughout the COVID-19 outbreak and beyond. Now more than ever, we need our non-profits. | | | | | | | | | | | | | | | |  |
|  |  | | | | | | | | | | | | | | | |  |
|  | | | **Files\\282 [8Dec20]** | | | | | | | | | | | | | |  |
|  |  |  | No |  | All articles |  | 0.2642 |  | 1 | |  | | | | | |  |
|  | | |  |  |  |  |  |  |  | |  | | | | | | |
|  | | | | | | | | | | | | 1 |  | LF |  | 17/02/2021 17:01 |  |
|  | 'Dr Iain Foulkes, executive director of research and innovation at Cancer Research UK, added: 'As a charity, we fund around half of the UK's publicly funded cancer research. Medical research charities like Cancer Research UK are the life blood of research and development in the UK, and we have all felt the devastating blow of the pandemic on our income.'The recent Government spending review was a step in the right direction for cancer services in the UK, but we need urgent clarification to what measures are being put in place to support medical research charities through the Life Sciences Charity Partnership Fund. As a country that relies so heavily on charity-funded research, the UK risks weakening its reputation as a world-leader in science if charities don't receive the right support.' | | | | | | | | | | | | | | | |  |
|  |  | | | | | | | | | | | | | | | |  |
|  | | | **Files\\299 [24Jun20]** | | | | | | | | | | | | | |  |
|  |  |  | No |  | All articles |  | 0.2645 |  | 1 | |  | | | | | |  |
|  | | |  |  |  |  |  |  |  | |  | | | | | | |
|  | | | | | | | | | | | | 1 |  | LF |  | 19/02/2021 15:01 |  |
|  | 'There is also the wider impact for the UK's role at the forefront of scientific research. Such a steep reduction in investment will inevitably diminish the country's reputation as a world leader in developing medical breakthroughs that save lives. We cannot afford to let this happen during a pandemic which itself has underlined the critical role research plays.'Chief executives from the Association of Medical Research Charities (AMRC), including CR UK, the BHF and Parkinson's UK, are now calling on the government to introduce a Life Sciences Partnership Fund and match charity funded research for the next three years.Medical research charities accounted for 1.9 billion (51%) of non-commercial research funding in the UK last year, but the AMRC is projecting a 310 million shortfall in this spend over the next year and expect it to take nearly five years for funding to return to previous levels.'It is imperative that the Government urgently works with medical research charities to come to a solution, so that decades of investment in UK research is not lost in a matter of months,' stressed Michelle Mitchell, Cancer Research UK's chief executive. | | | | | | | | | | | | | | | |  |
|  |  |  |  |  |  |  |  |  |  |  |  |  |  |  |  |  |  |
|  |  | | | | | | | | | | | | | | | |  |
|  | | | | | | | | | | | | | | | | | |
|  | | | | | | | | | | | | | | | | | |
| Reports\\Coding Summary By Code Report | | | | | | | | | | Page 23 of 123 | | | | | | | |
| 14/07/2021 10:54 | | | | | | | | | | | | | | | | | |
|  | | | **Aggregate** |  | **Classification** |  | **Coverage** |  | **Number Of Coding References** | |  | **Reference Number** |  | **Coded By Initials** |  | **Modified On** |  |
|  | | | **Files\\504 [7Jul20]** | | | | | | | | | | | | | |  |
|  |  |  | No |  | All articles |  | 0.4353 |  | 1 | |  | | | | | |  |
|  | | |  |  |  |  |  |  |  | |  | | | | | | |
|  | | | | | | | | | | | | 1 |  | LF |  | 08/03/2021 11:25 |  |
|  | The UK's medical research charities are calling for urgent financial support following decimation of research income amid the coronavirus pandemic.More than 60 of the country's top cardiovascular disease and cancer research scientists have signed an open letter to the Prime Minister urging that 'swift action to invest in a Life Sciences-Charity Partnership Fund to protect the vital and unique contribution charity-funded biomedical research makes to the UK's R&#38;D ecosystem and the wider economy.'The scientists voice concern that recent funding action from government for universities and charities will not be enough to address the significant shortfall in medical research charity investment in the UK science base.Last year, medical research charities invested 1.9 billion into UK research, but cancelled fundraising events and shop closures due to coronavirus have fuelled an unprecedented funding crisis.Cancer Research UK has already warned that it could be forced to cut 150 million per year from its research funding, while the BHF said it could have to cut its research spend by half this year - from 100 million to around 50 million.'Without immediate action, the UK's research base faces a devastating fall in funding that will delay progress in discovering new ways of preventing, diagnosing and treating diseases including heart attack, stroke and vascular dementia,' said Professor Sir Nilesh Samani, medical director at the BHF.'We also risk losing a generation of promising young researchers and diminishing the UK's standing as a world leader in science. | | | | | | | | | | | | | | | |  |
|  |  |  |  |  |  |  |  |  |  |  |  |  |  |  |  |  |  |
|  |  | | | | | | | | | | | | | | | |  |
|  | **Nodes\\Direct impacts\Availability of resources for cancer research\Long term impacts of funding shortfalls** | | | | | | | | | | | | | | | |  |
|  | | **PDF** | | | | | | | | | | | | | | |  |
|  | | | **Files\\56 [22Aug20]** | | | | | | | | | | | | | |  |
|  |  |  | No |  | All articles |  | 0.0819 |  | 1 | |  | | | | | |  |
|  | | |  |  |  |  |  |  |  | |  | | | | | | |
|  | | | | | | | | | | | | 1 |  | LF |  | 08/03/2021 15:07 |  |
|  | A stark example of the harm Covid-19 has caused UK scientiﬁc research is provided by cancer researchers. “We  had planned to spend £400m on research this year. That ﬁgure will have to be cut to £250m,” Aoife Regan of Cancer Research UK told the Observer. “That is £150m of research on treatments with the potential to save or lengthen lives that will not now take place.”  And that cutback is expected to persist for four or ﬁve more years, she added. “That means hundreds of millions of pounds will be stripped from cancer research in the UK in coming years and the impact will send ripples beyond that. Where are we going to ﬁnd the funds for the next generation of medical researchers? Where will they work? Covid-19 is going to have a major impact on the research landscape for a long time.” | | | | | | | | | | | | | | | |  |
|  |  | | | | | | | | | | | | | | | |  |
|  | | | | | | | | | | | | | | | | | |
|  | | | | | | | | | | | | | | | | | |
|  | | | | | | | | | | | | | | | | | |
| Reports\\Coding Summary By Code Report | | | | | | | | | | Page 24 of 123 | | | | | | | |
| 14/07/2021 10:54 | | | | | | | | | | | | | | | | | |
|  | | | **Aggregate** |  | **Classification** |  | **Coverage** |  | **Number Of Coding References** | |  | **Reference Number** |  | **Coded By Initials** |  | **Modified On** |  |
|  | **Nodes\\Direct impacts\Availability of resources for cancer research\Long term impacts of funding shortfalls\Stunted opportunities for early career researchers** | | | | | | | | | | | | | | | |  |
|  | | **PDF** | | | | | | | | | | | | | | |  |
|  | | | **Files\\12 [11 Jan 21]** | | | | | | | | | | | | | |  |
|  |  |  | No |  | All articles |  | 0.0113 |  | 1 | |  | | | | | |  |
|  | | |  |  |  |  |  |  |  | |  | | | | | | |
|  | | | | | | | | | | | | 1 |  | LF |  | 15/02/2021 15:53 |  |
|  | Ribas expects that the pandemic will continue to be particularly challenging for early-career investigators who are facing a lack of funding and job opportunities in the next year due to budgeting shortfalls at foundations, universities, and research institutions. Other pandemic-related challenges include reduced time in the lab to generate data and the inability to travel to job interviews or establish connections at in-person conferences.  In light of these challenges, Ribas believes supporting early-career investigators should be a priority in 2021. He noted that the AACR COVID-19 and Cancer Task Force is examining ways to address these challenges, such as advocating to lawmakers to increase funding for cancer research. “There are a lot of things we need to act on in a short time,” he said. | | | | | | | | | | | | | | | |  |
|  |  |  |  |  |  |  |  |  |  |  |  |  |  |  |  |  |  |
|  |  | | | | | | | | | | | | | | | |  |
|  | | | **Files\\46 (2) [25Sept20]** | | | | | | | | | | | | | |  |
|  |  |  | No |  | All articles |  | 0.0358 |  | 1 | |  | | | | | |  |
|  | | |  |  |  |  |  |  |  | |  | | | | | | |
|  | | | | | | | | | | | | 1 |  | LF |  | 09/03/2021 11:01 |  |
|  | For young investigators, just at the start of their careers, the COVID crisis puts an enormous financial burden on labs. New labs are like start-up businesses and finances can be extremely tight. Once those labs shut down, young researchers were put in shaky financial situations. “We are at a real risk of losing an entire generation of cancer researchers,” said Dr. Monje. | | | | | | | | | | | | | | | |  |
|  |  | | | | | | | | | | | | | | | |  |
|  | | | **Files\\72 [8April20]** | | | | | | | | | | | | | |  |
|  |  |  | No |  | All articles |  | 0.1207 |  | 1 | |  | | | | | |  |
|  | | |  |  |  |  |  |  |  | |  | | | | | | |
|  | | | | | | | | | | | | 1 |  | LF |  | 08/03/2021 17:20 |  |
|  | Recently, readers of this blog heard from NCI Director Norman E. Sharpless, M.D., who addressed the grantee community in a blog about NCI grant funding and operations during the COVID-19 emergency. Soon after that post, Dr. Sharpless and I discussed the importance of reaching out to our research trainees about the disruptions they are facing due to COVID-19.  In some cases, research trainees may be the principal investigator on their F30, F31, F32, F99/K00, K99/R00, K08, K22 or a legacy K-series award. In other cases, those aspiring to become cancer investigators may be supported by a T32 training grant or are working with principal investigators who have an R01 or some other NCI grant.  Oliver Bogler, Ph.D. Director, Center for Cancer Training Credit: National Cancer Institute  The uncertainty surrounding the rapidly evolving COVID-19 public health emergency is having a disproportionate effect on research trainees. Because their training positions are time-limited, every month counts in the race to gain the skills necessary to become independent investigators. As we quickly discovered during the past few weeks, the reduced ability to engage with other professionals—due to the physical  distancing essential to prevent the spread of COVID-19—raises challenges in many work environments. For those training to become cancer researchers, however, physical distancing and reduced interactions pose special problems.  The always-ticking “career clock” affects eligibility for training awards and the window of time for career transitions supported by NCI awards like the F99/K00, K99/R00, and K22. A pause of several months in research is a hardship for many dedicated to cancer discovery, but for trainees it’s both a challenge and an impediment. | | | | | | | | | | | | | | | |  |
|  |  |  |  |  |  |  |  |  |  |  |  |  |  |  |  |  |  |
|  |  |  |  |  |  |  |  |  |  |  |  |  |  |  |  |  |  |
|  |  | | | | | | | | | | | | | | | |  |
|  | | | | | | | | | | | | | | | | | |
| Reports\\Coding Summary By Code Report | | | | | | | | | | Page 25 of 123 | | | | | | | |
| 14/07/2021 10:54 | | | | | | | | | | | | | | | | | |
|  | | | **Aggregate** |  | **Classification** |  | **Coverage** |  | **Number Of Coding References** | |  | **Reference Number** |  | **Coded By Initials** |  | **Modified On** |  |
|  | | | **Files\\8 (2) [17Dec20]** | | | | | | | | | | | | | |  |
|  |  |  | No |  | All articles |  | 0.0439 |  | 1 | |  | | | | | |  |
|  | | |  |  |  |  |  |  |  | |  | | | | | | |
|  | | | | | | | | | | | | 1 |  | LF |  | 08/03/2021 17:44 |  |
|  | Those in the early stages of their career are particularly susceptible to budget cuts. “When you are just starting out, that is when you are most dependent on institutional support”, said Levine. “If we increase      the disincentives to stay in academia, or to enter the field in the first place, we risk losing a lot of talent.” Levine has particular concerns over the prospects for discovery science. “If you cannot get that extra 10% funding, then perhaps you will not be able to take that risk that might ultimately pay o~”, he explained. “That is a cost of the pandemic that we cannot begin to put a value on.” | | | | | | | | | | | | | | | |  |
|  |  | | | | | | | | | | | | | | | |  |
|  | | | **Files\\80 [15July20]** | | | | | | | | | | | | | |  |
|  |  |  | No |  | All articles |  | 0.0098 |  | 1 | |  | | | | | |  |
|  | | |  |  |  |  |  |  |  | |  | | | | | | |
|  | | | | | | | | | | | | 1 |  | LF |  | 08/03/2021 17:47 |  |
|  | Although some cancer researchers have pivoted their research programs toward understanding and containing COVID-19, many have unfortunately been prevented from doing so, as they were sequestered away from their laboratories in efforts to prevent its spread. This has been particularly damaging to trainees, many of whom are dependent upon continuous research productivity. | | | | | | | | | | | | | | | |  |
|  |  | | | | | | | | | | | | | | | |  |
|  | **Nodes\\Direct impacts\Availability of resources for cancer research\The need for supportive funding to help health systems recover** | | | | | | | | | | | | | | | |  |
|  | | **Document** | | | | | | | | | | | | | | |  |
|  | | | **Files\\4 [10Nov20]** | | | | | | | | | | | | | |  |
|  |  |  | No |  | All articles |  | 0.1172 |  | 1 | |  | | | | | |  |
|  | | |  |  |  |  |  |  |  | |  | | | | | | |
|  | | | | | | | | | | | | 1 |  | LF |  | 07/03/2021 11:53 |  |
|  | Unfortunately, many of the challenges that cancer services are experiencing are not new. Workforce and equipment shortages, particularly for diagnosing cancer, were holding back cancer services long before the pandemic. Cancer services need governments' help[11] to address deep seated challenges so they can come out of this crisis in a stronger position. Put simply, we need more staff, and for them to be fully equipped. Without this, the health services[12]' ability to recover from the pandemic will be hampered and it won't be possible to meet the government's ambitions to improve cancer survival in the long term. The UK Government's spending review is an opportunity to show their commitment to the NHS by giving them the investment needed to provide the best cancer care. With any extra funding, it's vital devolved governments to do the same for health systems across the UK. The spending review is also a crucial moment for Government to protect the role of life-saving research funded by medical research charities, who have been massively hit by COVID-19, with a Life Sciences—Charity Partnership Fund. | | | | | | | | | | | | | | | |  |
|  |  |  |  |  |  |  |  |  |  |  |  |  |  |  |  |  |  |
|  |  | | | | | | | | | | | | | | | |  |
|  | | | | | | | | | | | | | | | | | |
|  | | | | | | | | | | | | | | | | | |
| Reports\\Coding Summary By Code Report | | | | | | | | | | Page 26 of 123 | | | | | | | |
| 14/07/2021 10:54 | | | | | | | | | | | | | | | | | |
|  | | | **Aggregate** |  | **Classification** |  | **Coverage** |  | **Number Of Coding References** | |  | **Reference Number** |  | **Coded By Initials** |  | **Modified On** |  |
|  | | | **Files\\93 [14Sept20]** | | | | | | | | | | | | | |  |
|  |  |  | No |  | All articles |  | 0.0546 |  | 1 | |  | | | | | |  |
|  | | |  |  |  |  |  |  |  | |  | | | | | | |
|  | | | | | | | | | | | | 1 |  | LF |  | 09/03/2021 10:45 |  |
|  | The last six months has been one of the most challenging period in the history of the NHS. And thanks to the dedication of healthcare staff and the work of cancer recovery planners in all four UK nations, the figures are moving in the right direction. But there's a lot of work that still needs to be done to get cancer services back on track and to ensure that cancer screening, diagnosis and treatment will not be as severely impacted by any future waves of COVID-19. We've written before[6] about what needs to happen to make sure that everyone gets the care they need, when they need it. | | | | | | | | | | | | | | | |  |
|  |  | | | | | | | | | | | | | | | |  |
|  | **Nodes\\Direct impacts\COVID measures halting cancer research activity** | | | | | | | | | | | | | | | |  |
|  | | **Document** | | | | | | | | | | | | | | |  |
|  | | | **Files\\151 [26Mar20]** | | | | | | | | | | | | | |  |
|  |  |  | No |  | All articles |  | 0.2354 |  | 1 | |  | | | | | |  |
|  | | |  |  |  |  |  |  |  | |  | | | | | | |
|  | | | | | | | | | | | | 1 |  | LF |  | 17/02/2021 10:01 |  |
|  | For UC San Francisco graduate student Johnny Yu, the novel coronavirus pandemic chased him out of the lab, and out of San Francisco entirely. This academic year, UCSF set graduate student stipends at $40,000, according to the university, which Yu said is too low to afford to live in San Francisco.That's why he lives in a van — a tiny space with a chemical toilet where he can't envision quarantining if he got sick or weathering an extended shelter-in-place order. 'It's suitable for sleeping at night. But for a two-month lockdown, it would be pretty impossible,' Yu said. He fled instead to a rented yurt in Grass Valley with his wife, where they're digging into savings to pay for their stay.Yu studies how a cancer evolves from a primary tumor to become a metastatic tumor, and looks for potential treatments. 'You can treat a primary tumor, you can remove a primary tumor, but if you have a metastatic tumor, it's pretty much the end of the game,' Yu said.He's had to abruptly end his experiments as he prepares to stay away from the lab for the foreseeable future. 'I can't do any of the experiments I had,' Yu said. 'We killed everything. We killed all the mice, we killed all the cells.'He estimates that stopping now will set his experiments back six months — and as other cancer research grinds to a halt, the search for new treatments could be delayed even longer. 'If we can't find the cancer drugs and cancer targets, industry can't take it and develop it into a drug,' he said. 'At least a year, I think it will set back the entire field of cancer.'Yu's Ph.D. mentor, assistant professor Hani Goodarzi[10] (who is not responsible for setting Yu's salary), said the same story is true for many of Yu's colleagues. Half of Goodarzi's lab studies cancer progression from the lab bench, like Yu; the other half models cancer computationally.The experimental researchers, Goodarzi said, are at a scientific standstill right now. 'We actually shut down the lab a few days before UCSF shut down,' he said. 'So we're focused on writing papers, and grants, and added a journal club discussion via Zoom —to keep everyone engaged while they can't be at the bench.'With no ongoing lab research and no way to pivot to studying the novel coronavirus, Goodarzi did what he could to help with the fight. He gathered up all the gloves and surgical masks his team doesn't need now and donated them to UCSF Health.'I wish we could do more,' Goodarzi said. 'We kind of feel useless, to be honest The way that we can contribute is really just like everyone else, just by staying home.'Get | | | | | | | | | | | | | | | |  |
|  |  |  |  |  |  |  |  |  |  |  |  |  |  |  |  |  |  |
|  |  |  |  |  |  |  |  |  |  |  |  |  |  |  |  |  |  |
|  |  | | | | | | | | | | | | | | | |  |
|  | | | | | | | | | | | | | | | | | |
|  | | | | | | | | | | | | | | | | | |
| Reports\\Coding Summary By Code Report | | | | | | | | | | Page 27 of 123 | | | | | | | |
| 14/07/2021 10:54 | | | | | | | | | | | | | | | | | |
|  | | | **Aggregate** |  | **Classification** |  | **Coverage** |  | **Number Of Coding References** | |  | **Reference Number** |  | **Coded By Initials** |  | **Modified On** |  |
|  | | **PDF** | | | | | | | | | | | | | | |  |
|  | | | **Files\\34 [13Jan21]** | | | | | | | | | | | | | |  |
|  |  |  | No |  | All articles |  | 0.0175 |  | 1 | |  | | | | | |  |
|  | | |  |  |  |  |  |  |  | |  | | | | | | |
|  | | | | | | | | | | | | 1 |  | LF |  | 19/02/2021 16:54 |  |
|  | “With the abrupt campus lockdown including our laboratories, experiments that were conducted prior had to be immediately terminated without any plan as to how to proceed forward. Accessing our instrument centers to test our samples also became difficult due to the campus-wide social distancing guidelines. And on the front end, laboratory materials and chemicals now take much longer to ship when ordered,” said Geneus. | | | | | | | | | | | | | | | |  |
|  |  | | | | | | | | | | | | | | | |  |
|  | | | **Files\\46 (2) [25Sept20]** | | | | | | | | | | | | | |  |
|  |  |  | No |  | All articles |  | 0.0950 |  | 1 | |  | | | | | |  |
|  | | |  |  |  |  |  |  |  | |  | | | | | | |
|  | | | | | | | | | | | | 1 |  | LF |  | 09/03/2021 11:01 |  |
|  | When COVID-19 forced all but essential employees to work from home, childhood cancer researchers were faced with the same shutdown. Dr. Michelle Monje, an Alex’s Lemonade Stand Foundation (ALSF)-funded researcher who studies high-grade gliomas, had less than 12 hours to shut down her lab when California put shelter-in-place orders in effect on March 16.  Dr. Monje and her team scrambled to save the things they could not lose—like primary cell cultures from children’s tumors and backed up all data to hard drives. Her lab is open again, but with restrictions and social distancing guidelines in place.  Across the country in Boston, Dr. Steven Dubois’ lab at Dana Farber Cancer Institute experienced a similar emergency shutdown.  With research interrupted, experiments in the lab stopped, which then in turn impacted the pace of pushing experiments out of the lab and into the clinical trial phase. “This is a three-month shutdown, that will lead to a six-month lag,” said Dr. Dubois. | | | | | | | | | | | | | | | |  |
|  |  |  |  |  |  |  |  |  |  |  |  |  |  |  |  |  |  |
|  |  | | | | | | | | | | | | | | | |  |
|  | | | **Files\\74 [14Sept20]** | | | | | | | | | | | | | |  |
|  |  |  | No |  | All articles |  | 0.0175 |  | 1 | |  | | | | | |  |
|  | | |  |  |  |  |  |  |  | |  | | | | | | |
|  | | | | | | | | | | | | 1 |  | LF |  | 08/03/2021 17:22 |  |
|  | “Our own research to develop new treatments for cancer patients was also paused during lockdown, with the majority of ICR laboratories having to close temporarily to ensure the safety of staff,” she continued. “In the current landscape, cancer patients are more vulnerable than ever, and this adds urgency to our need to design smarter, kinder and more effective cancer treatments. For this reason, the ICR has launched a major fundraising appeal to kick-start our work and make up for the many vital research hours lost to the COVID-19 crisis.” | | | | | | | | | | | | | | | |  |
|  |  | | | | | | | | | | | | | | | |  |
|  | | | | | | | | | | | | | | | | | |
|  | | | | | | | | | | | | | | | | | |
|  | | | | | | | | | | | | | | | | | |
| Reports\\Coding Summary By Code Report | | | | | | | | | | Page 28 of 123 | | | | | | | |
| 14/07/2021 10:54 | | | | | | | | | | | | | | | | | |
|  | | | **Aggregate** |  | **Classification** |  | **Coverage** |  | **Number Of Coding References** | |  | **Reference Number** |  | **Coded By Initials** |  | **Modified On** |  |
|  | **Nodes\\Direct impacts\COVID measures halting cancer research activity\Deferrence of non-COVID research by institutions** | | | | | | | | | | | | | | | |  |
|  | | **PDF** | | | | | | | | | | | | | | |  |
|  | | | **Files\\119 (2) [no date]** | | | | | | | | | | | | | |  |
|  |  |  | No |  | All articles |  | 0.0259 |  | 1 | |  | | | | | |  |
|  | | |  |  |  |  |  |  |  | |  | | | | | | |
|  | | | | | | | | | | | | 1 |  | LF |  | 09/03/2021 14:37 |  |
|  | Although many of us have faced the unpleasant reality of having our laboratories impacted by regulatory authority decisions which defer NonCOVID19 research, cancer cannot wait and neither can our research. | | | | | | | | | | | | | | | |  |
|  |  | | | | | | | | | | | | | | | |  |
|  | **Nodes\\Direct impacts\COVID measures halting cancer research activity\Deferrence of non-COVID research by institutions\Delay in setting up new trials** | | | | | | | | | | | | | | | |  |
|  | | **PDF** | | | | | | | | | | | | | | |  |
|  | | | **Files\\30 (2) [16July20]** | | | | | | | | | | | | | |  |
|  |  |  | No |  | All articles |  | 0.0075 |  | 1 | |  | | | | | |  |
|  | | |  |  |  |  |  |  |  | |  | | | | | | |
|  | | | | | | | | | | | | 1 |  | LF |  | 09/03/2021 14:31 |  |
|  | As for new clinical trials, themselves, “We’re thinking about new trials, but we can’t start them,” he says. “It’s like designing a car but not being able to build it – only a million times more important.” | | | | | | | | | | | | | | | |  |
|  |  | | | | | | | | | | | | | | | |  |
|  | **Nodes\\Direct impacts\COVID measures halting cancer research activity\Labs temporarily closing** | | | | | | | | | | | | | | | |  |
|  | | **Document** | | | | | | | | | | | | | | |  |
|  | | | **Files\\379 [30Nov20]** | | | | | | | | | | | | | |  |
|  |  |  | No |  | All articles |  | 0.2265 |  | 1 | |  | | | | | |  |
|  | | |  |  |  |  |  |  |  | |  | | | | | | |
|  | | | | | | | | | | | | 1 |  | LF |  | 23/02/2021 15:55 |  |
|  | Nearly all respondents said that COVID-19 had impacted their work, with 36% reporting a 'moderate impact' and another 36% saying it had a 'substantial' impact.The main problems caused by the impact included closure of labs during lockdown and subsequent restrictions in access to facilities and equipment, with 91% of respondents citing these issues.Respondents also reported that an inability to enrol patients on clinical trials, access to clinical samples or interaction in person with colleagues had impacted their work. | | | | | | | | | | | | | | | |  |
|  |  | | | | | | | | | | | | | | | |  |
|  | | | | | | | | | | | | | | | | | |
| Reports\\Coding Summary By Code Report | | | | | | | | | | Page 29 of 123 | | | | | | | |
| 14/07/2021 10:54 | | | | | | | | | | | | | | | | | |
|  | | | **Aggregate** |  | **Classification** |  | **Coverage** |  | **Number Of Coding References** | |  | **Reference Number** |  | **Coded By Initials** |  | **Modified On** |  |
|  | | **PDF** | | | | | | | | | | | | | | |  |
|  | | | **Files\\30 (2) [16July20]** | | | | | | | | | | | | | |  |
|  |  |  | No |  | All articles |  | 0.0404 |  | 1 | |  | | | | | |  |
|  | | |  |  |  |  |  |  |  | |  | | | | | | |
|  | | | | | | | | | | | | 1 |  | LF |  | 19/02/2021 15:27 |  |
|  | But by that point, Spencer had spent a month preparing her lab for remote work. The Spencer lab uses cutting- edge technologies to watch single, live cells go about their business. Much of her lab’s cancer work is computational – they gather terabytes of data with nifty microscopes, but then it takes time to crunch the numbers to see what they found.  “I’ve been worried about this virus since early January,” Spencer says. “Despite being called Chicken Little by family, I started recommending to my lab to do all possible experiments first and save the analysis for later. Telling people to cram in six experiments without analysis isn’t normally how you would do science, but I was worried labs would shut down. Now, at least people in my lab have stockpiled data. However, we’re not going to last on that forever. Maybe another two months?” Two months was exactly what she needed. In May the labs across campuses started to open back up for research with heightened precautions such as social distancing and mask wearing, but being open is a great step in the right direction for research. | | | | | | | | | | | | | | | |  |
|  |  |  |  |  |  |  |  |  |  |  |  |  |  |  |  |  |  |
|  |  | | | | | | | | | | | | | | | |  |
|  | | | **Files\\34 [13Jan21]** | | | | | | | | | | | | | |  |
|  |  |  | No |  | All articles |  | 0.0175 |  | 1 | |  | | | | | |  |
|  | | |  |  |  |  |  |  |  | |  | | | | | | |
|  | | | | | | | | | | | | 1 |  | LF |  | 19/02/2021 16:57 |  |
|  | “With the abrupt campus lockdown including our laboratories, experiments that were conducted prior had to be immediately terminated without any plan as to how to proceed forward. Accessing our instrument centers to test our samples also became difficult due to the campus-wide social distancing guidelines. And on the front end, laboratory materials and chemicals now take much longer to ship when ordered,” said Geneus. | | | | | | | | | | | | | | | |  |
|  |  | | | | | | | | | | | | | | | |  |
|  | **Nodes\\Direct impacts\COVID measures halting cancer research activity\Labs temporarily closing\Lack of in-person interaction for researchers** | | | | | | | | | | | | | | | |  |
|  | | **Document** | | | | | | | | | | | | | | |  |
|  | | | **Files\\379 [30Nov20]** | | | | | | | | | | | | | |  |
|  |  |  | No |  | All articles |  | 0.2265 |  | 1 | |  | | | | | |  |
|  | | |  |  |  |  |  |  |  | |  | | | | | | |
|  | | | | | | | | | | | | 1 |  | LF |  | 23/02/2021 15:56 |  |
|  | Nearly all respondents said that COVID-19 had impacted their work, with 36% reporting a 'moderate impact' and another 36% saying it had a 'substantial' impact.The main problems caused by the impact included closure of labs during lockdown and subsequent restrictions in access to facilities and equipment, with 91% of respondents citing these issues.Respondents also reported that an inability to enrol patients on clinical trials, access to clinical samples or interaction in person with colleagues had impacted their work. | | | | | | | | | | | | | | | |  |
|  |  | | | | | | | | | | | | | | | |  |
|  | | | | | | | | | | | | | | | | | |
| Reports\\Coding Summary By Code Report | | | | | | | | | | Page 30 of 123 | | | | | | | |
| 14/07/2021 10:54 | | | | | | | | | | | | | | | | | |
|  | | | **Aggregate** |  | **Classification** |  | **Coverage** |  | **Number Of Coding References** | |  | **Reference Number** |  | **Coded By Initials** |  | **Modified On** |  |
|  | **Nodes\\Direct impacts\COVID measures halting cancer research activity\Labs temporarily closing\Some labs stayed open despite concerns** | | | | | | | | | | | | | | | |  |
|  | | **Document** | | | | | | | | | | | | | | |  |
|  | | | **Files\\25 [30Nov20]** | | | | | | | | | | | | | |  |
|  |  |  | No |  | All articles |  | 0.0505 |  | 1 | |  | | | | | |  |
|  | | |  |  |  |  |  |  |  | |  | | | | | | |
|  | | | | | | | | | | | | 1 |  | LF |  | 17/02/2021 15:07 |  |
|  | But the survey nevertheless laid bare the emotional impact of the pandemic on researchers. Some 69 percent of researchers said the impact of the pandemic on their work had left them 'frustrated," 39 percent had been 'saddened' and 25 percent 'depressed." The respondents were strongly supportive of efforts to keep labs open to prevent any further disruption to research advances for cancer patients. The ICR's labs have managed to stay open during the second lockdown period while taking significant measures to help prevent risk of spread. | | | | | | | | | | | | | | | |  |
|  |  | | | | | | | | | | | | | | | |  |
|  | **Nodes\\Direct impacts\COVID measures halting cancer research activity\Suspension of (non-clinical trial) cancer research studies** | | | | | | | | | | | | | | | |  |
|  | | **PDF** | | | | | | | | | | | | | | |  |
|  | | | **Files\\114 (2) [13 May 20]** | | | | | | | | | | | | | |  |
|  |  |  | No |  | All articles |  | 0.4134 |  | 1 | |  | | | | | |  |
|  | | |  |  |  |  |  |  |  | |  | | | | | | |
|  | | | | | | | | | | | | 1 |  | LF |  | 15/02/2021 15:04 |  |
|  | Nearly half of cancer research has been stopped due to the COVID-19 pandemic, according to the results of a survey of American Cancer Society (ACS) grantees.  In early April, the ACS surveyed all of its 744 currently funded researchers to assess the state of their projects during the COVID-19 pandemic; 488 grantees responded to the survey (66 percent response rate).  The researchers found that half of grantees (51 percent) indicated the pandemic had a "high impact," with all research or training activities paused until further notice; 43 percent reported a "modest impact," with only some aspects paused; and 7 percent reported a "low impact," with research or training continuing as planned. Just over half of respondents (54 percent) were working entirely remotely; one-third were working mostly remotely, with occasional in-person visits to the office/campus/lab; and 8 percent were working mostly or entirely in their normal work setting. Similarly, the vast majority (91 percent) reported their institutions only permitted essential personnel, 59 percent  1/2  reported their institutions had closed laboratories, 57 percent reported their institutions had temporarily halted research, and 4 percent reported their institutions remained entirely open.  "It is abundantly clear that the COVID-19 pandemic is having a major impact on cancer research," William C. Phelps, Ph.D., ACS senior vice president of extramural research, said in a statement. "In some labs queried for our survey, all nonessential research had been halted, with research on COVID-19 being the only type of research being encouraged. In addition to the deceleration in progress against cancer, these laboratories and institutions will face significant additional costs associated with restarting the cancer research enterprise in the coming months." | | | | | | | | | | | | | | | |  |
|  |  |  |  |  |  |  |  |  |  |  |  |  |  |  |  |  |  |
|  |  |  |  |  |  |  |  |  |  |  |  |  |  |  |  |  |  |
|  |  | | | | | | | | | | | | | | | |  |
|  | | | | | | | | | | | | | | | | | |
|  | | | | | | | | | | | | | | | | | |
| Reports\\Coding Summary By Code Report | | | | | | | | | | Page 31 of 123 | | | | | | | |
| 14/07/2021 10:54 | | | | | | | | | | | | | | | | | |
|  | | | **Aggregate** |  | **Classification** |  | **Coverage** |  | **Number Of Coding References** | |  | **Reference Number** |  | **Coded By Initials** |  | **Modified On** |  |
|  | | | **Files\\46 (2) [25Sept20]** | | | | | | | | | | | | | |  |
|  |  |  | No |  | All articles |  | 0.0950 |  | 1 | |  | | | | | |  |
|  | | |  |  |  |  |  |  |  | |  | | | | | | |
|  | | | | | | | | | | | | 1 |  | LF |  | 09/03/2021 10:59 |  |
|  | When COVID-19 forced all but essential employees to work from home, childhood cancer researchers were faced with the same shutdown. Dr. Michelle Monje, an Alex’s Lemonade Stand Foundation (ALSF)-funded researcher who studies high-grade gliomas, had less than 12 hours to shut down her lab when California put shelter-in-place orders in effect on March 16.  Dr. Monje and her team scrambled to save the things they could not lose—like primary cell cultures from children’s tumors and backed up all data to hard drives. Her lab is open again, but with restrictions and social distancing guidelines in place.  Across the country in Boston, Dr. Steven Dubois’ lab at Dana Farber Cancer Institute experienced a similar emergency shutdown.  With research interrupted, experiments in the lab stopped, which then in turn impacted the pace of pushing experiments out of the lab and into the clinical trial phase. “This is a three-month shutdown, that will lead to a six-month lag,” said Dr. Dubois. | | | | | | | | | | | | | | | |  |
|  |  |  |  |  |  |  |  |  |  |  |  |  |  |  |  |  |  |
|  |  | | | | | | | | | | | | | | | |  |
|  | | | **Files\\55 [25Mar20]** | | | | | | | | | | | | | |  |
|  |  |  | No |  | All articles |  | 0.0656 |  | 1 | |  | | | | | |  |
|  | | |  |  |  |  |  |  |  | |  | | | | | | |
|  | | | | | | | | | | | | 1 |  | LF |  | 08/03/2021 14:46 |  |
|  | At Yale University in New Haven, Connecticut, lung-cancer researcher Roy Herbst says clinical trials for cancer have been cut to “almost zero” and are allowed only when a participant is deemed to have exceptional need.  “It’s hard to believe that just a month ago, I’d never seen cancer clinical trials better,” says Herbst, citing a list of experimental treatments that were showing promise against some of the deadliest  RELATED  lung cancers. “Now the whole process has really ground to a halt, and I feel bad because there are patients who might have beneﬁted from those trials.”  The coronavirus pandemic in five powerful charts  But the measures are necessary, he adds. Many people with advanced cancer are vulnerable to infection, and trips to the clinic for treatments and assessments could be deadly if patients are exposed to the coronavirus. Some cancer treatments weaken the immune system, and advanced treatments that use genetically modiﬁed cells require intensive medical monitoring —  something that might not be possible in the middle of the outbreak. | | | | | | | | | | | | | | | |  |
|  |  |  |  |  |  |  |  |  |  |  |  |  |  |  |  |  |  |
|  |  | | | | | | | | | | | | | | | |  |
|  | | | **Files\\78 [13May20]** | | | | | | | | | | | | | |  |
|  |  |  | No |  | All articles |  | 0.3543 |  | 2 | |  | | | | | |  |
|  | | |  |  |  |  |  |  |  | |  | | | | | | |
|  | | | | | | | | | | | | 1 |  | LF |  | 08/03/2021 17:38 |  |
|  | According to a survey from the American Cancer Society (ACS), just more than half of grantees reported that their cancer research or training activities had been put on hold due to the COVID-19 pandemic.  In early April 2020, ACS surveyed all of its funded researchers (n=744) to assess the state of their projects and  collect information to guide its response to the COVID-19 pandemic. A total of 488 grantees responded, yielding a 66% response rate.  COVID-19 impact on cancer research When asked how the pandemic has impacted their work, 51% indicate a high impact, with all research or training activities paused until further notice; 43% report a modest impact, with some aspects of research on pause; and 7% report a low impact, with research or training continuing as planned. | | | | | | | | | | | | | | | |  |
|  |  |  |  |  |  |  |  |  |  |  |  |  |  |  |  |  |  |
|  |  | | | | | | | | | | | | | | | |  |
|  | | | | | | | | | | | | 2 |  | LF |  | 08/03/2021 17:39 |  |
|  | “It is abundantly clear that the COVID-19 pandemic is having a major impact on cancer research,”  said William C. Phelps, PhD, ACS senior vice president of extramural research, in a press release. “In some labs queried for our survey, all non-essential research had been halted, with research on COVID-19 being the only type of research being encouraged. In addition to the deceleration in progress against cancer, these laboratories and institutions will face significant additional costs associated with restarting the cancer research enterprise in the coming months.” | | | | | | | | | | | | | | | |  |
|  |  | | | | | | | | | | | | | | | |  |
|  | | | | | | | | | | | | | | | | | |
| Reports\\Coding Summary By Code Report | | | | | | | | | | Page 32 of 123 | | | | | | | |
| 14/07/2021 10:54 | | | | | | | | | | | | | | | | | |
|  | | | **Aggregate** |  | **Classification** |  | **Coverage** |  | **Number Of Coding References** | |  | **Reference Number** |  | **Coded By Initials** |  | **Modified On** |  |
|  | | | **Files\\84 (2) [12Oct20]** | | | | | | | | | | | | | |  |
|  |  |  | No |  | All articles |  | 0.1621 |  | 1 | |  | | | | | |  |
|  | | |  |  |  |  |  |  |  | |  | | | | | | |
|  | | | | | | | | | | | | 1 |  | LF |  | 09/03/2021 09:47 |  |
|  | Recruitment to cancer research trials fell by 87 per cent, and is still recovering  Nearly all clinical trials funded by Cancer Research UK were put on hold at the peak of the Covid-19 pandemic, according to new data released by the charity.  Some 95 per cent of CRUK trials were either paused completely or paused only in some places across the country, it said on 12 October.  Meanwhile, the setup of new trials was also put on hold, with the recruitment of new patients to existing trials falling by 87 per cent in April.  Research slowly started to recover a few months later, but recruitment in August was still 40 per cent lower than pre-pandemic levels. The recovery is also slower compared with other developed countries, the charity said.  Speaking at a Science Media Centre brie~ng on 12 October, Charles Swanton, Cancer Research UK’s chief clinician and group leader at the Francis Crick Institute, told journalists that Covid-19 had a~ected clinical research and trials “very dramatically”.  “During lockdown almost all cancer clinical trials were halted or temporarily stopped,” he said. “Many of these have been reopened but of course recruitment into these studies has been delayed.” | | | | | | | | | | | | | | | |  |
|  |  |  |  |  |  |  |  |  |  |  |  |  |  |  |  |  |  |
|  |  | | | | | | | | | | | | | | | |  |
|  | | | **Files\\96 [29Sept20]** | | | | | | | | | | | | | |  |
|  |  |  | No |  | All articles |  | 0.0577 |  | 1 | |  | | | | | |  |
|  | | |  |  |  |  |  |  |  | |  | | | | | | |
|  | | | | | | | | | | | | 1 |  | LF |  | 09/03/2021 10:52 |  |
|  | As the pandemic ga thered steam in the spring of 2020, a l l non-essentia l research opera tions were suspended a t insti tu tions across the worl d , incl ud ing a t Berkel ey. Research on SARS-CoV-2 was one of the few exceptions permi tted to continue. Five members of the Nomura l ab, who had previousl y been working on cancer research projects, vol unteered to swi tch thei r efforts to the new SARS-CoV-2 M screen. Working in nearl y empty campus bu i l d ings and l abs wi th fu l l PPE and staying d i stanced from thei r col l eagues, these ded ica ted researchers pu t in l ong hours to advance the work as qu ickl y as possibl e | | | | | | | | | | | | | | | |  |
|  |  | | | | | | | | | | | | | | | |  |
|  | **Nodes\\Direct impacts\COVID measures halting cancer research activity\Suspension of clinical trials** | | | | | | | | | | | | | | | |  |
|  | | **Document** | | | | | | | | | | | | | | |  |
|  | | | **Files\\127 [21 Apr 20]** | | | | | | | | | | | | | |  |
|  |  |  | No |  | All articles |  | 0.0388 |  | 1 | |  | | | | | |  |
|  | | |  |  |  |  |  |  |  | |  | | | | | | |
|  | | | | | | | | | | | | 1 |  | LF |  | 15/02/2021 16:48 |  |
|  | The specialist raises another medical concern — how the Covid-19 restrictions may be slashing at important research on cancer and other diseases, as clinical trials confront serious and growing disruptions.[12] The doctor wrote: 'Beyond its effect on standard treatments, the pandemic has also affected trials of experimental therapies. Many clinical trials have stopped enrolling patients over concerns about infection. These suspensions may be devastating for some patients who may not live to see the trials reopen.' | | | | | | | | | | | | | | | |  |
|  |  | | | | | | | | | | | | | | | |  |
|  | | | | | | | | | | | | | | | | | |
| Reports\\Coding Summary By Code Report | | | | | | | | | | Page 33 of 123 | | | | | | | |
| 14/07/2021 10:54 | | | | | | | | | | | | | | | | | |
|  | | | **Aggregate** |  | **Classification** |  | **Coverage** |  | **Number Of Coding References** | |  | **Reference Number** |  | **Coded By Initials** |  | **Modified On** |  |
|  | | | **Files\\143 [19Aug20]** | | | | | | | | | | | | | |  |
|  |  |  | No |  | All articles |  | 0.0506 |  | 1 | |  | | | | | |  |
|  | | |  |  |  |  |  |  |  | |  | | | | | | |
|  | | | | | | | | | | | | 1 |  | LF |  | 15/02/2021 17:35 |  |
|  | While we placed a brief pause on the recruitment of new patients in April, we have since lifted that pause and our operations have experienced minimal disruption to date', said Hugh S. Griffith, NuCana's Founder and Chief Executive Officer. 'We are driving recruitment in the ongoing global Phase III study of Acelarin plus cisplatin in patients with biliary tract cancer. We are also making good progress in the Phase Ib combination and Phase I monotherapy studies of NUC-3373 as well as the Phase I study of NUC-7738.' | | | | | | | | | | | | | | | |  |
|  |  | | | | | | | | | | | | | | | |  |
|  | | | **Files\\165 [30June20]** | | | | | | | | | | | | | |  |
|  |  |  | No |  | All articles |  | 0.8095 |  | 1 | |  | | | | | |  |
|  | | |  |  |  |  |  |  |  | |  | | | | | | |
|  | | | | | | | | | | | | 1 |  | LF |  | 17/02/2021 10:33 |  |
|  | Cancer Research UK is urging Scotland's government and health service providers to restart existing clinical trials for cancer as quickly as possible, following a 95% drop in new patients entering trials among the pandemic.Almost all clinical research trials in Scotland have been paused in response to COVID-19, thus removing innovative treatment options for many people living with cancer, a report to MSPs and charities also found.The Scottish government must boost coronavirus testing so that patients can participate in clinical trials safely and be treated in safe spaces where there is minimal risk of exposure to the virus, the charity noted.'Cancer hasn't stopped because of the pandemic and it's essential that clinical trials are restarted urgently,' said Marion O'Neill, Cancer Research UK's head of external affairs in Scotland. 'The Scottish Government and health boards, together with NHS Research Scotland, needs to move fast to get existing cancer clinical trials that provide a lifeline to patients and their families restarted.'Clinical trials are such an important part of our armoury.  For some of our patients with ovarian cancer, it's the last option for them,' added Professor Charlie Gourley is Clinical Director at the Cancer Research UK Edinburgh Centre.'So many trials are successful now that the pause has taken away hope for some patients when they know that they would be offered a trial under normal circumstances but, because of coronavirus, trials have been closed to new patients. This has been incredibly hard for patients.'The Cross Party Group on Cancer are considering findings of the report, which also assessed the impact of the pandemic on cancer diagnosis, treatment and care.Cancer Research UK recently highlighted concerns about the impact of COVID-19 on the future of cancer research, announcing that it might be forced to cut 150 million a year from its research funding because of a sharp drop in income caused by the pandemic. | | | | | | | | | | | | | | | |  |
|  |  |  |  |  |  |  |  |  |  |  |  |  |  |  |  |  |  |
|  |  |  |  |  |  |  |  |  |  |  |  |  |  |  |  |  |  |
|  |  | | | | | | | | | | | | | | | |  |
|  | | | **Files\\204 [7May20]** | | | | | | | | | | | | | |  |
|  |  |  | No |  | All articles |  | 0.1858 |  | 1 | |  | | | | | |  |
|  | | |  |  |  |  |  |  |  | |  | | | | | | |
|  | | | | | | | | | | | | 1 |  | LF |  | 17/02/2021 11:59 |  |
|  | Triple therapy threat for tumors In several laboratory models, including those using ER+ breast cancer samples from patients, the researchers showed that adding venetoclax to the combination of hormone therapy and a CDK4/6 inhibitor led to a better and longer-term response of the tumor to the therapy, said Professor Lindeman, who is a clinician-scientist at the Institute and a medical oncologist at the Peter MacCallum Cancer Centre. "These promising results provided a justification for starting clinical trials to look at a "triple therapy" combining venetoclax, hormone therapy and a CDK4/6 inhibitor in patients with ER+ breast cancer," Professor Lindeman said. "We have initiated the phase 1 PALVEN trial which will, in the first place, look at whether this triple therapy is safe for patients, and will also consider how patients" tumors respond to the triple therapy[9]. "It would be wonderful to see a new therapy that improves the outcomes of patients with ER+ breast[10] cancer[11]," Professor Lindeman said. Please note that due to COVID-19, the PALVEN trial is currently not accepting new participants. | | | | | | | | | | | | | | | |  |
|  |  |  |  |  |  |  |  |  |  |  |  |  |  |  |  |  |  |
|  |  | | | | | | | | | | | | | | | |  |
|  | | | **Files\\4 [10Nov20]** | | | | | | | | | | | | | |  |
|  |  |  | No |  | All articles |  | 0.0140 |  | 1 | |  | | | | | |  |
|  | | |  |  |  |  |  |  |  | |  | | | | | | |
|  | | | | | | | | | | | | 1 |  | LF |  | 07/03/2021 11:48 |  |
|  | Over 30,000 fewer people started their treatment than the same time last year too, and most cancer clinical trials were disrupted[6]. | | | | | | | | | | | | | | | |  |
|  |  | | | | | | | | | | | | | | | |  |
| Reports\\Coding Summary By Code Report | | | | | | | | | | Page 34 of 123 | | | | | | | |
| 14/07/2021 10:54 | | | | | | | | | | | | | | | | | |
|  | | | **Aggregate** |  | **Classification** |  | **Coverage** |  | **Number Of Coding References** | |  | **Reference Number** |  | **Coded By Initials** |  | **Modified On** |  |
|  | | | **Files\\88 [11Apr20]** | | | | | | | | | | | | | |  |
|  |  |  | No |  | All articles |  | 0.2151 |  | 1 | |  | | | | | |  |
|  | | |  |  |  |  |  |  |  | |  | | | | | | |
|  | | | | | | | | | | | | 1 |  | LF |  | 09/03/2021 09:56 |  |
|  | Rene Roach fired off a quick email in late March for an update on a colorectal cancer clinical trial for which she hoped to qualify.  Worried about the coronavirus, she asked, almost as an afterthought, whether the study had been put on hold because of the pandemic.The answer crushed her: It had been.  "That's when COVID-19 shut down everything," says Roach, 50, of Germantown, Md.  Roach assumed that there would be workarounds for patients like her, who have stage IV cancer. These patients often depend on clinical trials as their best chance to knock cancer out when other therapies have failed.  For now, she's being treated with traditional chemotherapy, but she was counting on the drug cocktail from the clinical trial. She figures if chemo was going to rid her body of cancer for good, it would have done so already.  "It's buying time. But how much time is that? I don't know," she says. "And so if this stops working, what else do I have?"  The COVID-19 pandemic has forced hundreds of clinical trials to grind to a halt, stalling research into cancer, strokes, dementia and more, an NPR analysis of federal clinical trials data has found.  As Americans have been told to stay home to prevent spreading the novel coronavirus, people enrolled in clinical trials are being notified that those trials have been suspended or won't begin as originally planned.  NPR has identified 440 studies that have been suspended since March 1 because of the outbreak. About a quarter of the studies put on hold were for cancer treatment. The studies involved as many as 200,000 people, though it's hard to know precisely how many were active in the studies at the time they were halted.  "It's a whole cascade of effects occurring here that is really interrupting the ability of patients to obtain treatment in clinical trials," says Dr. Richard Schilsky, chief medical officer of the American Society of Clinical Oncology. "We're hearing at least anecdotally that enrollment is falling." | | | | | | | | | | | | | | | |  |
|  |  |  |  |  |  |  |  |  |  |  |  |  |  |  |  |  |  |
|  |  |  |  |  |  |  |  |  |  |  |  |  |  |  |  |  |  |
|  |  | | | | | | | | | | | | | | | |  |
|  | | **PDF** | | | | | | | | | | | | | | |  |
|  | | | **Files\\1 (2) [8th Oct 20]** | | | | | | | | | | | | | |  |
|  |  |  | No |  | All articles |  | 0.0258 |  | 1 | |  | | | | | |  |
|  | | |  |  |  |  |  |  |  | |  | | | | | | |
|  | | | | | | | | | | | | 1 |  | LF |  | 15/02/2021 12:04 |  |
|  | Clinical cancer research, in which potential new therapies are tested in patients, has also experienced diﬃculties as a result of COVID-19. Some cancer centers halted enrollment on clinical trials entirely during the height of the pandemic. A survey of dozens of clinical investigators in March found that nearly 60 percent of respondents had halted screening and/or enrollment in certain trials, and that half of their institutions had ceased collection of blood and other tissue for research purposes. | | | | | | | | | | | | | | | |  |
|  |  | | | | | | | | | | | | | | | |  |
|  | | | **Files\\10 [21 Oct 20]** | | | | | | | | | | | | | |  |
|  |  |  | No |  | All articles |  | 0.0166 |  | 2 | |  | | | | | |  |
|  | | |  |  |  |  |  |  |  | |  | | | | | | |
|  | | | | | | | | | | | | 1 |  | LF |  | 15/02/2021 12:11 |  |
|  | At least 200 interventional oncolo~y trials listed in the U.S. National Institutes of Health global database were suspended between mid-March and early April, the study found. | | | | | | | | | | | | | | | |  |
|  |  | | | | | | | | | | | | | | | |  |
|  | | | | | | | | | | | | 2 |  | LF |  | 15/02/2021 12:14 |  |
|  | David Cameron, a professor of medical oncolo~y specializing in breast cancer at the University of Edinburgh, experienced the issue ﬁrst hand with a European study that was forced to pause recruitment for about six months once the lockdown came into eﬀect in March. | | | | | | | | | | | | | | | |  |
|  |  | | | | | | | | | | | | | | | |  |
|  | | | | | | | | | | | | | | | | | |
| Reports\\Coding Summary By Code Report | | | | | | | | | | Page 35 of 123 | | | | | | | |
| 14/07/2021 10:54 | | | | | | | | | | | | | | | | | |
|  | | | **Aggregate** |  | **Classification** |  | **Coverage** |  | **Number Of Coding References** | |  | **Reference Number** |  | **Coded By Initials** |  | **Modified On** |  |
|  | | | **Files\\114 (2) [13 May 20]** | | | | | | | | | | | | | |  |
|  |  |  | No |  | All articles |  | 0.4134 |  | 1 | |  | | | | | |  |
|  | | |  |  |  |  |  |  |  | |  | | | | | | |
|  | | | | | | | | | | | | 1 |  | LF |  | 15/02/2021 15:04 |  |
|  | Nearly half of cancer research has been stopped due to the COVID-19 pandemic, according to the results of a survey of American Cancer Society (ACS) grantees.  In early April, the ACS surveyed all of its 744 currently funded researchers to assess the state of their projects during the COVID-19 pandemic; 488 grantees responded to the survey (66 percent response rate).  The researchers found that half of grantees (51 percent) indicated the pandemic had a "high impact," with all research or training activities paused until further notice; 43 percent reported a "modest impact," with only some aspects paused; and 7 percent reported a "low impact," with research or training continuing as planned. Just over half of respondents (54 percent) were working entirely remotely; one-third were working mostly remotely, with occasional in-person visits to the office/campus/lab; and 8 percent were working mostly or entirely in their normal work setting. Similarly, the vast majority (91 percent) reported their institutions only permitted essential personnel, 59 percent  1/2  reported their institutions had closed laboratories, 57 percent reported their institutions had temporarily halted research, and 4 percent reported their institutions remained entirely open.  "It is abundantly clear that the COVID-19 pandemic is having a major impact on cancer research," William C. Phelps, Ph.D., ACS senior vice president of extramural research, said in a statement. "In some labs queried for our survey, all nonessential research had been halted, with research on COVID-19 being the only type of research being encouraged. In addition to the deceleration in progress against cancer, these laboratories and institutions will face significant additional costs associated with restarting the cancer research enterprise in the coming months." | | | | | | | | | | | | | | | |  |
|  |  |  |  |  |  |  |  |  |  |  |  |  |  |  |  |  |  |
|  |  |  |  |  |  |  |  |  |  |  |  |  |  |  |  |  |  |
|  |  | | | | | | | | | | | | | | | |  |
|  | | | **Files\\118 [1 May 20]** | | | | | | | | | | | | | |  |
|  |  |  | No |  | All articles |  | 0.0189 |  | 1 | |  | | | | | |  |
|  | | |  |  |  |  |  |  |  | |  | | | | | | |
|  | | | | | | | | | | | | 1 |  | LF |  | 15/02/2021 15:33 |  |
|  | Christian Nguyen (06:13):  We are doing a lot to combat the COVID-19 crisis, but rest assured that's not all that we're doing. The work that we otherwise would be doing as a company in many ways continues. Lilly is working with institutions globally to minimize the impact of the pandemic on ongoing research efforts.  We have taken proactive steps to ensure patient safety and minimizing further stress on healthcare systems including delaying initiation of new studies and pausing enrollment of new patients and most ongoing studies. | | | | | | | | | | | | | | | |  |
|  |  | | | | | | | | | | | | | | | |  |
|  | | | **Files\\22 [30Jun20]** | | | | | | | | | | | | | |  |
|  |  |  | No |  | All articles |  | 0.1207 |  | 1 | |  | | | | | |  |
|  | | |  |  |  |  |  |  |  | |  | | | | | | |
|  | | | | | | | | | | | | 1 |  | LF |  | 17/02/2021 14:12 |  |
|  | In the Netherlands, Professor Martin van den Bent, a neurologist from Erasmus MC – Daniel den Hoed Cancer Center in Rotterdam said, “During the height of the pandemic, clinical research came to a standstill as we tried to avoid patient visits to the hospital. In addition, monitoring and site activation activities were suspended.” “Initially, all clinical studies were put on-hold during the early days of the COVID-19 outbreak to maximize hospital capacity for COVID-19 cases,” continues Dr Gabe Sonke a medical  oncologist from the Netherlands Cancer Institute in Amsterdam. This was due to the uncertainty of the e~ect of experimental medication on the risk of infection and severe sequalae and the fact that they could not ensure compliance to trials due to the outbreak. | | | | | | | | | | | | | | | |  |
|  |  | | | | | | | | | | | | | | | |  |
|  | | | **Files\\24 [14Sept20]** | | | | | | | | | | | | | |  |
|  |  |  | No |  | All articles |  | 0.0289 |  | 2 | |  | | | | | |  |
|  | | |  |  |  |  |  |  |  | |  | | | | | | |
|  | | | | | | | | | | | | 1 |  | LF |  | 17/02/2021 14:28 |  |
|  | For nearly 30 years, Gateway has been funding Phase I and Phase II studies, and in recent years, these studies have increasingly focused on precision oncology. To date, the organization has supported over 180 studies, and currently has more than 50 active trials. Burton estimated that upwards of 70 percent of Gateway-supported studies were impacted by the pandemic. There were some immunotherapy trials where patients were on their last line of treatment that kept going because of the disease severity, but enrollment within many drug trials slowed and some had to be entirely suspended. | | | | | | | | | | | | | | | |  |
| Reports\\Coding Summary By Code Report | | | | | | | | | | Page 36 of 123 | | | | | | | |
| 14/07/2021 10:54 | | | | | | | | | | | | | | | | | |
|  | | | **Aggregate** |  | **Classification** |  | **Coverage** |  | **Number Of Coding References** | |  | **Reference Number** |  | **Coded By Initials** |  | **Modified On** |  |
|  | | | | | | | | | | | | | | | | | |
|  | | | | | | | | | | | | 2 |  | LF |  | 17/02/2021 14:29 |  |
|  | A survey of 34 academic and community cancer research programs conducted in late March by the American Society of Clinical Oncology provides a broader view of the decisions made by cancer centers because of COVID-19. Approximately 60 percent of the respondents' research programs halted screening and enrollment for certain clinical trials, while more than 50 percent said their institutions also prioritized enrollment for some trials based on patients' needs, safety, and disease severity; the burden enrollment would have on patients and the trial site; and availability of resources. | | | | | | | | | | | | | | | |  |
|  |  | | | | | | | | | | | | | | | |  |
|  | | | **Files\\5 [29Jun20]** | | | | | | | | | | | | | |  |
|  |  |  | No |  | All articles |  | 0.0314 |  | 1 | |  | | | | | |  |
|  | | |  |  |  |  |  |  |  | |  | | | | | | |
|  | | | | | | | | | | | | 1 |  | LF |  | 08/03/2021 10:40 |  |
|  | Delays in clinical research activities were common, the survey found. A majority of respondents’ programs stopped cancer screening and/or enrollment for certain clinical trials, including those that were conducted  Collecting Information about COVID-19 and Cancer  NCI is supporting clinical trials to test promising treatments for patients with COVID-19 and to learn more about the disease’s effects on the body. Some of the trials are specifically for patients with cancer. And St. Jude has created an online resource for physicians and researchers from around the world to contribute data on childhood cancer and the coronavirus.  for research purposes only.  “Many cancer centers began to prioritize which trials could continue to enroll patients based on the severity of the disease and the treatment options available to a patient, and also on the center’s own staffing,” said Dr. Waterhouse.  These findings were consistent with the results of a second survey involving 36 investigators that was conducted between March 23 and April 3. The enrollment of patients in active cancer clinical trials decreased during the survey period. In the United States and Europe, only 20% and 14%, respectively, of the institutions continued to enroll patients at the usual rate. | | | | | | | | | | | | | | | |  |
|  |  |  |  |  |  |  |  |  |  |  |  |  |  |  |  |  |  |
|  |  | | | | | | | | | | | | | | | |  |
|  | | | **Files\\77 [11Apr20]** | | | | | | | | | | | | | |  |
|  |  |  | No |  | All articles |  | 0.0484 |  | 1 | |  | | | | | |  |
|  | | |  |  |  |  |  |  |  | |  | | | | | | |
|  | | | | | | | | | | | | 1 |  | LF |  | 08/03/2021 17:36 |  |
|  | Rene Roach fired off a quick email in late March for an update on a colorectal cancer clinical trial for which she hoped to qualify.  Worried about the coronavirus, she asked, almost as an afterthought, whether the study had been put on hold because of the pandemic.The answer crushed her: It had been.  "That's when COVID-19 shut down everything," says Roach, 50, of Germantown, Md.  Roach assumed that there would be workarounds for patients like her, who have stage IV cancer. These patients often depend on clinical trials as their best chance to knock cancer out when other therapies have failed.  For now, she's being treated with traditional chemotherapy, but she was counting on the drug cocktail from the clinical trial. She figures if chemo was going to rid her body of cancer for good, it would have done so already.  "It's buying time. But how much time is that? I don't know," she says. "And so if this stops working, what else do I have?"  The COVID-19 pandemic has forced hundreds of clinical trials to grind to a halt, stalling research into cancer, strokes, dementia and more, an NPR analysis of federal clinical trials data has found. | | | | | | | | | | | | | | | |  |
|  |  |  |  |  |  |  |  |  |  |  |  |  |  |  |  |  |  |
|  |  | | | | | | | | | | | | | | | |  |
|  | | | | | | | | | | | | | | | | | |
|  | | | | | | | | | | | | | | | | | |
| Reports\\Coding Summary By Code Report | | | | | | | | | | Page 37 of 123 | | | | | | | |
| 14/07/2021 10:54 | | | | | | | | | | | | | | | | | |
|  | | | **Aggregate** |  | **Classification** |  | **Coverage** |  | **Number Of Coding References** | |  | **Reference Number** |  | **Coded By Initials** |  | **Modified On** |  |
|  | | | **Files\\89 (2) [24May20]** | | | | | | | | | | | | | |  |
|  |  |  | No |  | All articles |  | 0.2778 |  | 1 | |  | | | | | |  |
|  | | |  |  |  |  |  |  |  | |  | | | | | | |
|  | | | | | | | | | | | | 1 |  | LF |  | 09/03/2021 10:09 |  |
|  | A group of researchers from multinational contract research organization (CRO) IQVIA and the Cancer Research Institute studied COVID-19-related delays of major clinical trials around the world. The impact has been deep and severe. With a focus on oncology research—in many cases, impacting life and death for those severely ill with forms of cancer—over 200 cancerbased clinical trials were suspended between March and April based on surveys and a review of Clincialtrials.gov. For existing studies, enrollment rates have slowed due to pandemic related issues such as lockdowns, safety concerns, research staff shortages as well as potential risks associated with type of cancer treatment and route of administration.  The Research was led by CRO IQVIA and the Cancer Research Institute during the period of March 23, to April 3, 2020. The pair surveyed 36 investigators involved with oncology studies at centers around the world. IQVIA by itself studied a subset of 200 of the CRO’s  1/2  oncology trials in a bid to identify significant risks impacting the studies. The study was published in Nature Reviews Drug Discovery.  Major Impacts to Cancer Trials  Especially in the United States and Europe, the Pandemic hammered the clinical trials sector: Only 20% of sponsors in the U.S. and 14% in Europe continued to enroll patients at the usual rates. For those that continued to enroll with lower enrollment rates, patient care represented a key factor with 9 out of 13 respondents suggesting it as one of the top considerations triggering challenges in ongoing patient enrollment.  Certain cancer therapies involving route of administration represented a key consideration as did concerns about patient safety and the possibility of lack of key research staff and other resources. Investigators suggested in interviews that risk-benefit analysis centering on patients in ongoing trials was high on the mind of whether to proceed, regardless of design or complexity of trial. | | | | | | | | | | | | | | | |  |
|  |  |  |  |  |  |  |  |  |  |  |  |  |  |  |  |  |  |
|  |  |  |  |  |  |  |  |  |  |  |  |  |  |  |  |  |  |
|  |  | | | | | | | | | | | | | | | |  |
|  | **Nodes\\Direct impacts\COVID measures limiting cancer research activity** | | | | | | | | | | | | | | | |  |
|  | | **Document** | | | | | | | | | | | | | | |  |
|  | | | **Files\\151 [26Mar20]** | | | | | | | | | | | | | |  |
|  |  |  | No |  | All articles |  | 0.0504 |  | 1 | |  | | | | | |  |
|  | | |  |  |  |  |  |  |  | |  | | | | | | |
|  | | | | | | | | | | | | 1 |  | LF |  | 17/02/2021 09:52 |  |
|  | 'With California Gov. Gavin Newsom's order for Californians to shelter in place[4], university campuses across the state began gauging the risk of letting research continue. And scientists are finding their work, plans and lives upended by the spread of the novel coronavirus.Some, like Miller, are switching gears to fight the virus — but for others, research is grinding to a halt, includingcritical medical research. Still more are caught in limbo, waiting to find out if they can continue important studies without endangering themselves, or others. | | | | | | | | | | | | | | | |  |
|  |  | | | | | | | | | | | | | | | |  |
|  | | | **Files\\25 [30Nov20]** | | | | | | | | | | | | | |  |
|  |  |  | No |  | All articles |  | 0.1281 |  | 1 | |  | | | | | |  |
|  | | |  |  |  |  |  |  |  | |  | | | | | | |
|  | | | | | | | | | | | | 1 |  | LF |  | 17/02/2021 15:05 |  |
|  | The Institute of Cancer Research (ICR), which has discovered more cancer drugs than any other academic center in the world, has like many research organizations been hit by cuts to its own fundraising income and to grants from other charities. The ICR had to pause much of its work during the initial lockdown, and is now running a major fundraising appeal to help kick-start its research and make up for lost time. The ICR surveyed 239 of its researchers in order to detail the impact the pandemic has had on its research and to point towards ways of moving research forward again as quickly as possible. Respondents said they had lost an average of 10 weeks of research time to the first lockdown itself, and that their own scientific advances would be pushed back by an average of six months. Almost all said COVID-19 had had an impact on their work—with 36 percent saying it had had a 'moderate' impact, another 36 percent a 'substantial' impact and 5 percent an 'extreme' impact. Some 91 percent said the biggest problem had been the closure of labs during lockdown and subsequent restrictions in access to facilities and equipment—citing, for example, closure of major, national research facilities. The average ICR researcher spent 53 percent of their working time in a lab before lockdown, plummeting to 5 percent during lockdown and since recovering to 34 percent. | | | | | | | | | | | | | | | |  |
|  |  |  |  |  |  |  |  |  |  |  |  |  |  |  |  |  |  |
|  |  | | | | | | | | | | | | | | | |  |
| Reports\\Coding Summary By Code Report | | | | | | | | | | Page 38 of 123 | | | | | | | |
| 14/07/2021 10:54 | | | | | | | | | | | | | | | | | |
|  | | | **Aggregate** |  | **Classification** |  | **Coverage** |  | **Number Of Coding References** | |  | **Reference Number** |  | **Coded By Initials** |  | **Modified On** |  |
|  | | | **Files\\582 [5Mar20]** | | | | | | | | | | | | | |  |
|  |  |  | No |  | All articles |  | 0.0313 |  | 1 | |  | | | | | |  |
|  | | |  |  |  |  |  |  |  | |  | | | | | | |
|  | | | | | | | | | | | | 1 |  | LF |  | 08/03/2021 15:16 |  |
|  | The Fred Hutchinson Cancer Research Center in Seattle announced it is canceling events at the complex and requiring nonessential staff to work remotely at least through the end of the month to lessen the chance of infection among patients with weakened immune systems. | | | | | | | | | | | | | | | |  |
|  |  | | | | | | | | | | | | | | | |  |
|  | | **PDF** | | | | | | | | | | | | | | |  |
|  | | | **Files\\1 (2) [8th Oct 20]** | | | | | | | | | | | | | |  |
|  |  |  | No |  | All articles |  | 0.0087 |  | 1 | |  | | | | | |  |
|  | | |  |  |  |  |  |  |  | |  | | | | | | |
|  | | | | | | | | | | | | 1 |  | LF |  | 15/02/2021 12:02 |  |
|  | To limit the opportunities for viral transmission, many research centers enacted policies limiting the number of lab workers allowed on-site, putting many studies on hold. | | | | | | | | | | | | | | | |  |
|  |  | | | | | | | | | | | | | | | |  |
|  | | | **Files\\10 [21 Oct 20]** | | | | | | | | | | | | | |  |
|  |  |  | No |  | All articles |  | 0.0383 |  | 2 | |  | | | | | |  |
|  | | |  |  |  |  |  |  |  | |  | | | | | | |
|  | | | | | | | | | | | | 1 |  | LF |  | 15/02/2021 12:20 |  |
|  | At the University of Edinburgh, Carsten Hansen works with a team of researchers in a lab to identify variations of mesothelioma — a cancer in the lungs caused by asbestos exposure that can kill patients within a year of diagnosis — in order to develop targeted treatment.  But in mid-March, when the country was forced to lock down, Hansen and his team scrambled to preserve what they could and packed up any work that could be done from outside the lab.  “Laboratory-based research were not deemed essential workers,” he said, adding the approach wasn’t universal and some countries such as South Korea and Japan allowed research to continue. | | | | | | | | | | | | | | | |  |
|  |  | | | | | | | | | | | | | | | |  |
|  | | | | | | | | | | | | 2 |  | LF |  | 15/02/2021 12:20 |  |
|  | Even though he’s been back in the lab since mid-July, Hansen said, his team is having to work in a rotation since social distancing only allows facilities to operate up to 50 percent capacity.  “It's clearly a huge challenge,” he said of the impact on collaboration and teaching in the lab. “That, of course, also has an impact on how we can take the project forward.” | | | | | | | | | | | | | | | |  |
|  |  | | | | | | | | | | | | | | | |  |
|  | | | **Files\\118 [1 May 20]** | | | | | | | | | | | | | |  |
|  |  |  | No |  | All articles |  | 0.0178 |  | 1 | |  | | | | | |  |
|  | | |  |  |  |  |  |  |  | |  | | | | | | |
|  | | | | | | | | | | | | 1 |  | LF |  | 15/02/2021 15:31 |  |
|  | As the virus spread around the world, Lilly moved very quickly to protect our employees by restricting travel and practicing social distancing. Additionally, we moved very early to a work from home policy for almost everyone but those who manufacture and maintain the supply of our medicines. This was done yes, to reduce the risk of spreading the virus, yes, to protect our employees, but also to ensure that the millions of patients who rely on Lilly medicines every day continue to have access to them. | | | | | | | | | | | | | | | |  |
|  |  | | | | | | | | | | | | | | | |  |
|  | | | | | | | | | | | | | | | | | |
| Reports\\Coding Summary By Code Report | | | | | | | | | | Page 39 of 123 | | | | | | | |
| 14/07/2021 10:54 | | | | | | | | | | | | | | | | | |
|  | | | **Aggregate** |  | **Classification** |  | **Coverage** |  | **Number Of Coding References** | |  | **Reference Number** |  | **Coded By Initials** |  | **Modified On** |  |
|  | | | **Files\\27 [26Aug20]** | | | | | | | | | | | | | |  |
|  |  |  | No |  | All articles |  | 0.0196 |  | 1 | |  | | | | | |  |
|  | | |  |  |  |  |  |  |  | |  | | | | | | |
|  | | | | | | | | | | | | 1 |  | LF |  | 17/02/2021 15:37 |  |
|  | Your whole career has been focused on cancer research. How does it feel to tackle something completely diﬀerent?  Changing gears was interes~ng. Since we’ve been inundated by COVID-19 in both the lay press and the scien~ﬁc literature, I was already immersed in that space. In the spring when we had to ramp-down in the labs, we had to halt most of our cancer research-related studies. | | | | | | | | | | | | | | | |  |
|  |  | | | | | | | | | | | | | | | |  |
|  | | | **Files\\46 (2) [25Sept20]** | | | | | | | | | | | | | |  |
|  |  |  | No |  | All articles |  | 0.0231 |  | 1 | |  | | | | | |  |
|  | | |  |  |  |  |  |  |  | |  | | | | | | |
|  | | | | | | | | | | | | 1 |  | LF |  | 09/03/2021 11:01 |  |
|  | Oncology research labs are now back at limited capacities, with rotating schedules and distancing guidelines put in place. Researchers are focused on regaining the momentum they had before the shutdown—because children with cancer need cures. | | | | | | | | | | | | | | | |  |
|  |  | | | | | | | | | | | | | | | |  |
|  | **Nodes\\Direct impacts\COVID measures limiting cancer research activity\Decreased access to clinical samples** | | | | | | | | | | | | | | | |  |
|  | | **Document** | | | | | | | | | | | | | | |  |
|  | | | **Files\\25 [30Nov20]** | | | | | | | | | | | | | |  |
|  |  |  | No |  | All articles |  | 0.0278 |  | 1 | |  | | | | | |  |
|  | | |  |  |  |  |  |  |  | |  | | | | | | |
|  | | | | | | | | | | | | 1 |  | LF |  | 09/03/2021 14:28 |  |
|  | The next most cited impacts were inability to enroll patients on clinical trials[2] (60 percent), to access clinical samples (46 percent) or to interact in person with colleagues (41 percent) - with video conferencing seen as a poor substitute for meeting in person at conferences and other events. | | | | | | | | | | | | | | | |  |
|  |  | | | | | | | | | | | | | | | |  |
|  | | | **Files\\379 [30Nov20]** | | | | | | | | | | | | | |  |
|  |  |  | No |  | All articles |  | 0.2265 |  | 1 | |  | | | | | |  |
|  | | |  |  |  |  |  |  |  | |  | | | | | | |
|  | | | | | | | | | | | | 1 |  | LF |  | 09/03/2021 14:28 |  |
|  | Nearly all respondents said that COVID-19 had impacted their work, with 36% reporting a 'moderate impact' and another 36% saying it had a 'substantial' impact.The main problems caused by the impact included closure of labs during lockdown and subsequent restrictions in access to facilities and equipment, with 91% of respondents citing these issues.Respondents also reported that an inability to enrol patients on clinical trials, access to clinical samples or interaction in person with colleagues had impacted their work. | | | | | | | | | | | | | | | |  |
|  |  | | | | | | | | | | | | | | | |  |
|  | | | | | | | | | | | | | | | | | |
| Reports\\Coding Summary By Code Report | | | | | | | | | | Page 40 of 123 | | | | | | | |
| 14/07/2021 10:54 | | | | | | | | | | | | | | | | | |
|  | | | **Aggregate** |  | **Classification** |  | **Coverage** |  | **Number Of Coding References** | |  | **Reference Number** |  | **Coded By Initials** |  | **Modified On** |  |
|  | **Nodes\\Direct impacts\COVID measures limiting cancer research activity\Decreases in participant recruitment, participant attrition** | | | | | | | | | | | | | | | |  |
|  | | **Document** | | | | | | | | | | | | | | |  |
|  | | | **Files\\25 [30Nov20]** | | | | | | | | | | | | | |  |
|  |  |  | Yes |  | All articles |  | 0.0278 |  | 1 | |  | | | | | |  |
|  | | |  |  |  |  |  |  |  | |  | | | | | | |
|  | | | | | | | | | | | | 1 |  | LF |  | 09/03/2021 14:35 |  |
|  | The next most cited impacts were inability to enroll patients on clinical trials[2] (60 percent), to access clinical samples (46 percent) or to interact in person with colleagues (41 percent) - with video conferencing seen as a poor substitute for meeting in person at conferences and other events. | | | | | | | | | | | | | | | |  |
|  |  | | | | | | | | | | | | | | | |  |
|  | | | **Files\\26 [23June20]** | | | | | | | | | | | | | |  |
|  |  |  | Yes |  | All articles |  | 0.0198 |  | 1 | |  | | | | | |  |
|  | | |  |  |  |  |  |  |  | |  | | | | | | |
|  | | | | | | | | | | | | 1 |  | LF |  | 09/03/2021 14:35 |  |
|  | COVID-19 has had a huge impact on the UK's ability to run clinical trials, with the number of new patients being recruit on to UK-based trials falling by 95% in April 2020 compared with April 2019. | | | | | | | | | | | | | | | |  |
|  |  | | | | | | | | | | | | | | | |  |
|  | | | **Files\\292 [2Apr20]** | | | | | | | | | | | | | |  |
|  |  |  | Yes |  | All articles |  | 0.1171 |  | 1 | |  | | | | | |  |
|  | | |  |  |  |  |  |  |  | |  | | | | | | |
|  | | | | | | | | | | | | 1 |  | LF |  | 09/03/2021 14:35 |  |
|  | Helix BioPharma's US Phase 1b/II pancreatic study of L-DOS47 in combination with doxorubicin (LDOS006) continues to enroll patients and has dosed two patients. But the COVID-19 crisis is expected to affect patient enrollment. The Toronto-based company said it is working closely with the clinical trial site and the hospital to ensure the best care is being provided to patients while ensuring Helix complies with the hospital'sCOVID-19 protocols. | | | | | | | | | | | | | | | |  |
|  |  | | | | | | | | | | | | | | | |  |
|  | | **PDF** | | | | | | | | | | | | | | |  |
|  | | | **Files\\10 [21 Oct 20]** | | | | | | | | | | | | | |  |
|  |  |  | Yes |  | All articles |  | 0.0345 |  | 3 | |  | | | | | |  |
|  | | |  |  |  |  |  |  |  | |  | | | | | | |
|  | | | | | | | | | | | | 1 |  | LF |  | 15/02/2021 12:08 |  |
|  | Hilton, 48, was diagnosed with an aggressive form of breast cancer four years ago. After a double mastectomy, surgeries to remove her ovaries and fallopian tubes that were also at risk of cancer due to a BRCA1 gene mutation, and many rounds of chemotherapy, Hilton said her treatment options are dwindling.  In March, she received a prognosis of six to 12 months. Still receiving chemo, she wasn’t looking to enroll in a trial at the height of the pandemic. | | | | | | | | | | | | | | | |  |
|  |  | | | | | | | | | | | | | | | |  |
|  | | | | | | | | | | | | 2 |  | LF |  | 15/02/2021 12:11 |  |
|  | While doctors do everything they can to get patients into appropriate trials, additional restrictions on travel or other factors related to the pandemic make the fears of patients like Hilton valid, she added. | | | | | | | | | | | | | | | |  |
|  |  | | | | | | | | | | | | | | | |  |
| Reports\\Coding Summary By Code Report | | | | | | | | | | Page 41 of 123 | | | | | | | |
| 14/07/2021 10:54 | | | | | | | | | | | | | | | | | |
|  | | | **Aggregate** |  | **Classification** |  | **Coverage** |  | **Number Of Coding References** | |  | **Reference Number** |  | **Coded By Initials** |  | **Modified On** |  |
|  | | | | | | | | | | | | | | | | | |
|  | | | | | | | | | | | | 3 |  | LF |  | 09/03/2021 14:35 |  |
|  | In March, when Europe and the United States were being slammed by the pandemic, a study out of the Cancer Research Institute in New York found only about 14 percent of clinical trials by institutions were recruiting new patients at regular levels. | | | | | | | | | | | | | | | |  |
|  |  | | | | | | | | | | | | | | | |  |
|  | | | **Files\\24 [14Sept20]** | | | | | | | | | | | | | |  |
|  |  |  | Yes |  | All articles |  | 0.0259 |  | 2 | |  | | | | | |  |
|  | | |  |  |  |  |  |  |  | |  | | | | | | |
|  | | | | | | | | | | | | 1 |  | LF |  | 09/03/2021 14:35 |  |
|  | In addition to drug studies, the organization also funds research to find ways of improving cancer patients quality of life, and "right off the bat, one of the first institutions that pulled back in that area was MD Anderson Cancer Center," Burton recalled. Other cancer centers followed, shifting lab testing space and other resources to COVID-19 related care and research, and implementing safety measures to limit SARSCoV-2 transmission that kept patients from accessing trials. | | | | | | | | | | | | | | | |  |
|  |  | | | | | | | | | | | | | | | |  |
|  | | | | | | | | | | | | 2 |  | LF |  | 17/02/2021 14:30 |  |
|  | These data point to the difficult decisions cancer centers had to make quickly to balance access to critical care with safety: Cancer patients are among the most vulnerable to the ravages of coronavirus, with a 16 percent mortality rate, three times higher than in the general population. Although cancer patients are used to accepting a high level of risk in their care, the added fear of a potentially deadly viral infection likely made patients more cautious. "Patients weren't eager to get into planes, trains, and automobiles to get access to their studies," said Burton. | | | | | | | | | | | | | | | |  |
|  |  | | | | | | | | | | | | | | | |  |
|  | | | **Files\\30 (2) [16July20]** | | | | | | | | | | | | | |  |
|  |  |  | Yes |  | All articles |  | 0.0136 |  | 1 | |  | | | | | |  |
|  | | |  |  |  |  |  |  |  | |  | | | | | | |
|  | | | | | | | | | | | | 1 |  | LF |  | 09/03/2021 14:35 |  |
|  | But the more immediate effect of COVID-19 is on current patients, especially the enrollment of patients on clinical trials that test promising new therapies.  “The pandemic has pushed down clinical trial enrollment,” says Schulick. “Some of these clinical trials are going to be positive and when enrollment is down, we may be losing the opportunity to help patients.” | | | | | | | | | | | | | | | |  |
|  |  | | | | | | | | | | | | | | | |  |
|  | | | **Files\\38 (2) [1Dec20]** | | | | | | | | | | | | | |  |
|  |  |  | Yes |  | All articles |  | 0.0454 |  | 1 | |  | | | | | |  |
|  | | |  |  |  |  |  |  |  | |  | | | | | | |
|  | | | | | | | | | | | | 1 |  | LF |  | 09/03/2021 10:56 |  |
|  | Just as patient visits (https://www.aptitudehealth.com/blog/covid-19-impactcancer-patients/) for treatments and monitoring have been disrupted, participation in clinical trials has also decreased. Clinical trials, particularly those evaluating novel agents, mandate frequent tests and physician visits for assessment without a guaranteed return of therapeutic bene~t. In the midst of a pandemic, standard riskbene~t ratios have shifted, as physicians and patients must now also consider the potential increase in risk of contagion associated with the hospital and lab visits needed for assessments. Without a guaranteed return of therapeutic bene~t, this risk may outweigh the bene~ts for some prospective clinical trial participants. | | | | | | | | | | | | | | | |  |
|  |  | | | | | | | | | | | | | | | |  |
|  | | | | | | | | | | | | | | | | | |
|  | | | | | | | | | | | | | | | | | |
| Reports\\Coding Summary By Code Report | | | | | | | | | | Page 42 of 123 | | | | | | | |
| 14/07/2021 10:54 | | | | | | | | | | | | | | | | | |
|  | | | **Aggregate** |  | **Classification** |  | **Coverage** |  | **Number Of Coding References** | |  | **Reference Number** |  | **Coded By Initials** |  | **Modified On** |  |
|  | | | **Files\\50 (2) [no date]** | | | | | | | | | | | | | |  |
|  |  |  | Yes |  | All articles |  | 0.0227 |  | 1 | |  | | | | | |  |
|  | | |  |  |  |  |  |  |  | |  | | | | | | |
|  | | | | | | | | | | | | 1 |  | LF |  | 09/03/2021 14:35 |  |
|  | Adapting Cancer Clinical Trials, Launching New Trials  Not surprisingly, the COVID-19 pandemic has had a substantial impact on cancer clinical trials. I have spoken with directors and other staff from NCI-designated cancer centers and this is a serious concern.  As NCI Deputy Director Jim Doroshow, MD, reported at the board meeting, accrual to NCI-funded treatment clinical trials has dropped by half, a trend that is expected to persist. Accrual to certain trials has continued — for example, those offering life-saving therapies or those for patients who have no other options for therapy — but accrual to most other types of trials has sharply declined. | | | | | | | | | | | | | | | |  |
|  |  | | | | | | | | | | | | | | | |  |
|  | | | **Files\\89 (2) [24May20]** | | | | | | | | | | | | | |  |
|  |  |  | Yes |  | All articles |  | 0.1147 |  | 1 | |  | | | | | |  |
|  | | |  |  |  |  |  |  |  | |  | | | | | | |
|  | | | | | | | | | | | | 1 |  | LF |  | 09/03/2021 10:10 |  |
|  | Especially in the United States and Europe, the Pandemic hammered the clinical trials sector: Only 20% of sponsors in the U.S. and 14% in Europe continued to enroll patients at the usual rates. For those that continued to enroll with lower enrollment rates, patient care represented a key factor with 9 out of 13 respondents suggesting it as one of the top considerations triggering challenges in ongoing patient enrollment.  Certain cancer therapies involving route of administration represented a key consideration as did concerns about patient safety and the possibility of lack of key research staff and other resources. Investigators suggested in interviews that risk-benefit analysis centering on patients in ongoing trials was high on the mind of whether to proceed, regardless of design or complexity of trial. | | | | | | | | | | | | | | | |  |
|  |  | | | | | | | | | | | | | | | |  |
|  | **Nodes\\Direct impacts\COVID measures limiting cancer research activity\Decreases in participant recruitment, participant attrition\Hesitancy of cancer patients to participate in research** | | | | | | | | | | | | | | | |  |
|  | | **PDF** | | | | | | | | | | | | | | |  |
|  | | | **Files\\10 [21 Oct 20]** | | | | | | | | | | | | | |  |
|  |  |  | No |  | All articles |  | 0.0252 |  | 2 | |  | | | | | |  |
|  | | |  |  |  |  |  |  |  | |  | | | | | | |
|  | | | | | | | | | | | | 1 |  | LF |  | 15/02/2021 12:08 |  |
|  | Hilton, 48, was diagnosed with an aggressive form of breast cancer four years ago. After a double mastectomy, surgeries to remove her ovaries and fallopian tubes that were also at risk of cancer due to a BRCA1 gene mutation, and many rounds of chemotherapy, Hilton said her treatment options are dwindling.  In March, she received a prognosis of six to 12 months. Still receiving chemo, she wasn’t looking to enroll in a trial at the height of the pandemic. | | | | | | | | | | | | | | | |  |
|  |  | | | | | | | | | | | | | | | |  |
|  | | | | | | | | | | | | 2 |  | LF |  | 15/02/2021 12:11 |  |
|  | While doctors do everything they can to get patients into appropriate trials, additional restrictions on travel or other factors related to the pandemic make the fears of patients like Hilton valid, she added. | | | | | | | | | | | | | | | |  |
|  |  | | | | | | | | | | | | | | | |  |
|  | | | | | | | | | | | | | | | | | |
| Reports\\Coding Summary By Code Report | | | | | | | | | | Page 43 of 123 | | | | | | | |
| 14/07/2021 10:54 | | | | | | | | | | | | | | | | | |
|  | | | **Aggregate** |  | **Classification** |  | **Coverage** |  | **Number Of Coding References** | |  | **Reference Number** |  | **Coded By Initials** |  | **Modified On** |  |
|  | | | **Files\\24 [14Sept20]** | | | | | | | | | | | | | |  |
|  |  |  | No |  | All articles |  | 0.0141 |  | 1 | |  | | | | | |  |
|  | | |  |  |  |  |  |  |  | |  | | | | | | |
|  | | | | | | | | | | | | 1 |  | LF |  | 17/02/2021 14:30 |  |
|  | These data point to the difficult decisions cancer centers had to make quickly to balance access to critical care with safety: Cancer patients are among the most vulnerable to the ravages of coronavirus, with a 16 percent mortality rate, three times higher than in the general population. Although cancer patients are used to accepting a high level of risk in their care, the added fear of a potentially deadly viral infection likely made patients more cautious. "Patients weren't eager to get into planes, trains, and automobiles to get access to their studies," said Burton. | | | | | | | | | | | | | | | |  |
|  |  | | | | | | | | | | | | | | | |  |
|  | | | **Files\\38 (2) [1Dec20]** | | | | | | | | | | | | | |  |
|  |  |  | No |  | All articles |  | 0.0454 |  | 1 | |  | | | | | |  |
|  | | |  |  |  |  |  |  |  | |  | | | | | | |
|  | | | | | | | | | | | | 1 |  | LF |  | 09/03/2021 10:56 |  |
|  | Just as patient visits (https://www.aptitudehealth.com/blog/covid-19-impactcancer-patients/) for treatments and monitoring have been disrupted, participation in clinical trials has also decreased. Clinical trials, particularly those evaluating novel agents, mandate frequent tests and physician visits for assessment without a guaranteed return of therapeutic bene~t. In the midst of a pandemic, standard riskbene~t ratios have shifted, as physicians and patients must now also consider the potential increase in risk of contagion associated with the hospital and lab visits needed for assessments. Without a guaranteed return of therapeutic bene~t, this risk may outweigh the bene~ts for some prospective clinical trial participants. | | | | | | | | | | | | | | | |  |
|  |  | | | | | | | | | | | | | | | |  |
|  | | | **Files\\89 (2) [24May20]** | | | | | | | | | | | | | |  |
|  |  |  | No |  | All articles |  | 0.1147 |  | 1 | |  | | | | | |  |
|  | | |  |  |  |  |  |  |  | |  | | | | | | |
|  | | | | | | | | | | | | 1 |  | LF |  | 09/03/2021 10:10 |  |
|  | Especially in the United States and Europe, the Pandemic hammered the clinical trials sector: Only 20% of sponsors in the U.S. and 14% in Europe continued to enroll patients at the usual rates. For those that continued to enroll with lower enrollment rates, patient care represented a key factor with 9 out of 13 respondents suggesting it as one of the top considerations triggering challenges in ongoing patient enrollment.  Certain cancer therapies involving route of administration represented a key consideration as did concerns about patient safety and the possibility of lack of key research staff and other resources. Investigators suggested in interviews that risk-benefit analysis centering on patients in ongoing trials was high on the mind of whether to proceed, regardless of design or complexity of trial. | | | | | | | | | | | | | | | |  |
|  |  | | | | | | | | | | | | | | | |  |
|  | | | | | | | | | | | | | | | | | |
|  | | | | | | | | | | | | | | | | | |
|  | | | | | | | | | | | | | | | | | |
| Reports\\Coding Summary By Code Report | | | | | | | | | | Page 44 of 123 | | | | | | | |
| 14/07/2021 10:54 | | | | | | | | | | | | | | | | | |
|  | | | **Aggregate** |  | **Classification** |  | **Coverage** |  | **Number Of Coding References** | |  | **Reference Number** |  | **Coded By Initials** |  | **Modified On** |  |
|  | **Nodes\\Direct impacts\COVID measures limiting cancer research activity\Delayed or cancelled trial participant visits** | | | | | | | | | | | | | | | |  |
|  | | **PDF** | | | | | | | | | | | | | | |  |
|  | | | **Files\\10 [21 Oct 20]** | | | | | | | | | | | | | |  |
|  |  |  | No |  | All articles |  | 0.0257 |  | 1 | |  | | | | | |  |
|  | | |  |  |  |  |  |  |  | |  | | | | | | |
|  | | | | | | | | | | | | 1 |  | LF |  | 09/03/2021 14:34 |  |
|  | The lead author of the study, Samik Upadhaya, said patients already enrolled were also aﬀected.  “Nearly 60 percent of the investigators we surveyed reported that the pandemic had a moderate or a high impact in delaying or canceling patient visits to these trials,” he said. “There's a signiﬁcant disruption in terms of collecting crucial patient data.”  It will be very diﬃcult to measure the impact these disruptions will have on the advancement of medicine and on the lives of patients in the months and years ahead, Ian Walker, director of research for the charity Cancer Research U.K., said.  But the eﬀects will be “signiﬁcant” and “will undoubtedly set us back,” he said. | | | | | | | | | | | | | | | |  |
|  |  | | | | | | | | | | | | | | | |  |
|  | **Nodes\\Direct impacts\COVID measures limiting cancer research activity\Diversion of research resources away from cancer toward COVID** | | | | | | | | | | | | | | | |  |
|  | | **Document** | | | | | | | | | | | | | | |  |
|  | | | **Files\\68 [29Apr20]** | | | | | | | | | | | | | |  |
|  |  |  | Yes |  | All articles |  | 0.1472 |  | 2 | |  | | | | | |  |
|  | | |  |  |  |  |  |  |  | |  | | | | | | |
|  | | | | | | | | | | | | 1 |  | LF |  | 08/03/2021 16:32 |  |
|  | Credit: Cancer Research UK Since the outbreak of COVID-19, many of our scientists have been using the techniques and approaches developed over decades of cancer research to understand and defeat the virus. While beating cancer remains our priority, we will not be able to fully focus on our mission until COVID-19 is beaten. | | | | | | | | | | | | | | | |  |
|  |  | | | | | | | | | | | | | | | |  |
|  | | | | | | | | | | | | 2 |  | LF |  | 08/03/2021 16:36 |  |
|  | It's a technique the lab has been perfecting for several years, but their work was brought to a temporary halt by the COVID-19 outbreak. The COVID-19 pandemic has forced universities to partially close, and a lot of Parker's lab work had to be shut down. Like many other scientists working at home, they wondered how they could use their skills to aid research into the virus. After receiving a phone call[5] from a colleague who wanted to develop tests to determine if people infected with COVID-19 had developed neutralising antibodies, Parker had a realisation—the technique his lab uses to help the immune system recognise cancer cells could also be used to train the immune system to recognise and destroy the COVID-19 virus. | | | | | | | | | | | | | | | |  |
|  |  | | | | | | | | | | | | | | | |  |
|  | | | **Files\\70 [1Jun20]** | | | | | | | | | | | | | |  |
|  |  |  | Yes |  | All articles |  | 0.0260 |  | 1 | |  | | | | | |  |
|  | | |  |  |  |  |  |  |  | |  | | | | | | |
|  | | | | | | | | | | | | 1 |  | LF |  | 08/03/2021 16:51 |  |
|  | Victoria is a PhD student who normally works on lung cancer research, but since the pandemic, has returned to the frontline as a doctor at the The Christie NHS Foundation Trust in Manchester. | | | | | | | | | | | | | | | |  |
|  |  | | | | | | | | | | | | | | | |  |
|  | | | | | | | | | | | | | | | | | |
| Reports\\Coding Summary By Code Report | | | | | | | | | | Page 45 of 123 | | | | | | | |
| 14/07/2021 10:54 | | | | | | | | | | | | | | | | | |
|  | | | **Aggregate** |  | **Classification** |  | **Coverage** |  | **Number Of Coding References** | |  | **Reference Number** |  | **Coded By Initials** |  | **Modified On** |  |
|  | | **PDF** | | | | | | | | | | | | | | |  |
|  | | | **Files\\20 (2) [15May20]** | | | | | | | | | | | | | |  |
|  |  |  | Yes |  | All articles |  | 0.0422 |  | 1 | |  | | | | | |  |
|  | | |  |  |  |  |  |  |  | |  | | | | | | |
|  | | | | | | | | | | | | 1 |  | LF |  | 17/02/2021 11:49 |  |
|  | As leaders in immunotherapy, Memorial Sloan Kettering researchers have spent decades studying how the immune system responds to cancer. During the COVID-19 pandemic, many of them are applying their insights toward gaining a deeper understanding of how the immune system reacts to the coronavirus that causes COVID-19.  In an article published April 30 in the Journal of Experimental Medicine, MSK physician-scientists Jedd Wolchok and Santosha Vardhana share what they’ve learned on the front lines of caring for people with both cancer and COVID-19. They explain how the immune system’s response leads to the symptoms of COVID-19. They also outline how that response has pointed to potential therapies for COVID-19. Some of these treatments are already being tested in clinical trials at MSK and other hospitals.  Santosha Vardhana | | | | | | | | | | | | | | | |  |
|  |  |  |  |  |  |  |  |  |  |  |  |  |  |  |  |  |  |
|  |  | | | | | | | | | | | | | | | |  |
|  | | | **Files\\27 [26Aug20]** | | | | | | | | | | | | | |  |
|  |  |  | Yes |  | All articles |  | 0.0265 |  | 1 | |  | | | | | |  |
|  | | |  |  |  |  |  |  |  | |  | | | | | | |
|  | | | | | | | | | | | | 1 |  | LF |  | 17/02/2021 15:37 |  |
|  | Your whole career has been focused on cancer research. How does it feel to tackle something completely diﬀerent?  Changing gears was interes~ng. Since we’ve been inundated by COVID-19 in both the lay press and the scien~ﬁc literature, I was already immersed in that space. In the spring when we had to ramp-down in the labs, we had to halt most of our cancer research-related studies. This was an opportunity to pursue a gene that is involved in prostate cancer progression, that we have studied at great length already. | | | | | | | | | | | | | | | |  |
|  |  | | | | | | | | | | | | | | | |  |
|  | | | **Files\\47 (2) [21Dec20]** | | | | | | | | | | | | | |  |
|  |  |  | Yes |  | All articles |  | 0.0414 |  | 1 | |  | | | | | |  |
|  | | |  |  |  |  |  |  |  | |  | | | | | | |
|  | | | | | | | | | | | | 1 |  | LF |  | 09/03/2021 11:02 |  |
|  | By taking a lesson from prostate cancer, researchers now have a promising lead on a treatment for COVID-19.  Two proteins, ACE2 and TMPRSS2, help the coronavirus gain entry and replicate within cells. TMPRSS2 is wellknown to Arul Chinnaiyan, M.D., Ph.D. His lab discovered that TMPRSS2 fuses with the ETS gene to drive more than half of all prostate cancers. They also knew that TMPRSS2 was regulated by the androgen receptor.  So when cancer research shut down in the spring, Chinnaiyan's lab turned its attention to the coronavirus. With a grant from the National Cancer Institute, the team used its existing knowledge and resources to determine how TMPRSS2 was regulated in the lungs. | | | | | | | | | | | | | | | |  |
|  |  | | | | | | | | | | | | | | | |  |
|  | | | **Files\\50 (2) [no date]** | | | | | | | | | | | | | |  |
|  |  |  | Yes |  | All articles |  | 0.0182 |  | 1 | |  | | | | | |  |
|  | | |  |  |  |  |  |  |  | |  | | | | | | |
|  | | | | | | | | | | | | 1 |  | LF |  | 09/03/2021 11:04 |  |
|  | Redirecting Resources and Expertise at Frederick National Lab   The FNLCR is the only Federally Funded Research and Development Center (FFRDC) dedicated to biomedical research. Located in Frederick, Maryland, FNLCR is a research powerhouse with top-notch scientists and cutting-edge technologies. As NCI Deputy Director Doug Lowy, MD, explained during last week’s board meeting, FNLCR is perfectly suited to respond to a crisis like the coronavirus pandemic. And NCI is bringing those resources to bear in response to COVID-19. | | | | | | | | | | | | | | | |  |
|  |  | | | | | | | | | | | | | | | |  |
|  | | | | | | | | | | | | | | | | | |
| Reports\\Coding Summary By Code Report | | | | | | | | | | Page 46 of 123 | | | | | | | |
| 14/07/2021 10:54 | | | | | | | | | | | | | | | | | |
|  | | | **Aggregate** |  | **Classification** |  | **Coverage** |  | **Number Of Coding References** | |  | **Reference Number** |  | **Coded By Initials** |  | **Modified On** |  |
|  | | | **Files\\69 (2) [29June20]** | | | | | | | | | | | | | |  |
|  |  |  | Yes |  | All articles |  | 0.3448 |  | 1 | |  | | | | | |  |
|  | | |  |  |  |  |  |  |  | |  | | | | | | |
|  | | | | | | | | | | | | 1 |  | LF |  | 08/03/2021 16:43 |  |
|  | When the COVID-19 pandemic started, researchers at the Case Comprehensive Cancer Center immediately created plans to shift activities towards combating this deadly disease. Here are three examples of the impact on member research.  Identifying potential therapeutic agents against SARSCoV-2 Over the past 10 years, the Berdis laboratory, led by Anthony Berdis, PhD (/cancer/members/member-directory/anthony-j-berdis), has developed several new chemical agents that function as anti-cancer agents against acute lymphoblastic leukemia (ALL) and glioblastoma multiforme (GBM). The primary focus of this work is the development and pre-clinical testing of artificial deoxyribonucleotides that target DNA polymerase activity which is responsible for the hyperproliferative nature of these cancers. Viruses such as SARSCoV-2, the causative agent of the COVID-19 pandemic, are also classified as hyperproliferative organisms as they replicate uncontrollably. To combat SARS-CoV2, the Berdis lab has begun efforts to develop unique ribonucleoside analogs designed to efficiently and selectively inhibit viral replication catalyzed by the viral RNA-dependent RNA polymerase. To date, the lab has cloned the viral RNA-dependent RNA polymerase gene, introduced the cloned gene into an E. coli overexpression system, and purified the enzyme to ~95% homogeneity. They are currently assessing the ability of our analogs to function as chain-  terminating substrates for the polymerase. This represents an important first step in the drug discovery process to develop therapeutic agents against SARS-CoV-2 and other potential coronaviruses that can infect humans and other mammals.  Developing innovative strategies for treatment of SARS-CoV-2 infection Zheng-Rong Lu, PhD (/cancer/members/memberdirectory/zheng-rong-lu) leads a lab that is primarily dedicated to cancer research, focused on the translational development of 1) peptide-conjugated imaging probes specifically targeted to molecular markers of the tumor microenvironment for MRI- and PET-based detection and risk-stratification of neoplasms and 2) nanoparticle-mediated delivery of macromolecular therapeutics like siRNA, miRNA, lncRNA, CRISPR/Cas, and plasmid DNA for treatment of cancers and ocular diseases. With the beginning of the devastating COVID-19 pandemic, the lab began employing its technical and intellectual resources towards designing and developing innovative strategies for treatment of SARS-CoV-2 infection. Partnering with collaborators, the lab is customizing a versatile nanoparticle platform to deliver siRNAs and miRNAs to target the SARS-CoV-2 genome, and are evaluating its feasibility of inhibiting viral replication with RNAi therapy. Some promising results have been obtained to inhibiting viral replication. Using their expertise in peptide research, the lab has identified and generated novel peptides with high binding affinity to coronaviral epitopes to develop synthetic antibodies for potentially blocking and neutralizing the coronaviral particles. In this way, in addition to the lab's regular research, they are also endeavoring to address the challenges of the current pandemic and those of any future viral outbreaks.  Flow Cytometry facility maintains functionality to support critical research As the COVID19 shut down approached Case Western Reserve University (CWRU), the team of cytometrists in the Cancer Center Flow Cytometry and Imaging Microscopy Shared Resource (/cancer/research/shared-resources/cytometry-andmicroscopy) worked to make a plan to maintain functionality of the Core while keeping machines open for critical research. Through the use of masks, limitation of user overlap, religious cleaning regimes, and expansion of device usage over seven days a week coupled with expanded usage hours, CWRU was one of a handful of comprehensive cancer center cytometry cores in the US to remain fully operational through the shutdown. This was only made possible as a result of  the dedication of the Core Staff: Mike Sramkoski (Manager), D'Arbra Blankenship (Assistant Manager) and Kyla Johnson who worked on alternating schedules to meet the needs of the research community. As a result of their dedication to the mission of the Cancer Center and recognition of the great need for these technologies, our researchers are better positioned for the upcoming grant deadlines than those who had no or very limited access to research facilities. | | | | | | | | | | | | | | | |  |
|  |  |  |  |  |  |  |  |  |  |  |  |  |  |  |  |  |  |
|  |  |  |  |  |  |  |  |  |  |  |  |  |  |  |  |  |  |
|  |  |  |  |  |  |  |  |  |  |  |  |  |  |  |  |  |  |
|  |  |  |  |  |  |  |  |  |  |  |  |  |  |  |  |  |  |
|  |  |  |  |  |  |  |  |  |  |  |  |  |  |  |  |  |  |
|  |  | | | | | | | | | | | | | | | |  |
|  | | | **Files\\70 (2) [14April20]** | | | | | | | | | | | | | |  |
|  |  |  | Yes |  | All articles |  | 0.1426 |  | 1 | |  | | | | | |  |
|  | | |  |  |  |  |  |  |  | |  | | | | | | |
|  | | | | | | | | | | | | 1 |  | LF |  | 08/03/2021 17:18 |  |
|  | Last week, the National Cancer Institute (NCI) advisory meeting was focused on how the NCI is involved in the attack against COVID-19 while maintaining a commitment to cancer research and cancer care. This was the first virtual Joint Meeting (https://deainfo.nci.nih.gov/advisory/bsa/bsameetings.htm) (see presentation links) of the NCI Board of Scientific Advisors (BSA) and the National Cancer Advisory Board. The decision to open this meeting to the general public shows a commitment to information-sharing and transparency by NCI Director Ned Sharpless . The links provide access to three of the presentations that outline the benefits to research funding to NIH by Congress to focus on COVID-19 and interesting cysteine-like tether technology developed at the Frederick National Laboratory for Cancer Research (FNLCR) that is being applied to COVID protein inhibition. They are worth a read.  Other examples include providing information for people with cancer through the Cancer Information Service, pivoting some cancer research activities  to COVID-19/SARS-CoV-2 research and advocating for cancer researchers and providing them with means to share information and continue their research (https://www.cancer.gov/contact/emergencypreparedness/coronavirus-researchers#extramural-funding). National cancer registries are collecting data on the impact of COVID infection. | | | | | | | | | | | | | | | |  |
|  |  |  |  |  |  |  |  |  |  |  |  |  |  |  |  |  |  |
|  |  | | | | | | | | | | | | | | | |  |
| Reports\\Coding Summary By Code Report | | | | | | | | | | Page 47 of 123 | | | | | | | |
| 14/07/2021 10:54 | | | | | | | | | | | | | | | | | |
|  | | | **Aggregate** |  | **Classification** |  | **Coverage** |  | **Number Of Coding References** | |  | **Reference Number** |  | **Coded By Initials** |  | **Modified On** |  |
|  | | | **Files\\74 [14Sept20]** | | | | | | | | | | | | | |  |
|  |  |  | Yes |  | All articles |  | 0.0112 |  | 1 | |  | | | | | |  |
|  | | |  |  |  |  |  |  |  | |  | | | | | | |
|  | | | | | | | | | | | | 1 |  | LF |  | 08/03/2021 17:26 |  |
|  | “In parallel to the decreases in cancer care demand and capacity, there has been a significant reduction in clinical trial capacity and activity as hospitals, cancer researchers and research infrastructure have refocused on COVID-19,” explained Dr Davie. “Clinical trials have been delayed and most laboratory-based research has been interrupted.” | | | | | | | | | | | | | | | |  |
|  |  | | | | | | | | | | | | | | | |  |
|  | | | **Files\\76 [3Apr20]** | | | | | | | | | | | | | |  |
|  |  |  | Yes |  | All articles |  | 0.1892 |  | 2 | |  | | | | | |  |
|  | | |  |  |  |  |  |  |  | |  | | | | | | |
|  | | | | | | | | | | | | 1 |  | LF |  | 08/03/2021 17:34 |  |
|  | A team of Cardiff University scientists has switched from researching cancer to work that could help towards a vaccine for coronavirus.  The team at the School of Medicine usually work on reprogramming viruses so they can target and kill cancer - but are now focusing their efforts to help in the ﬁght against the new virus which is gripping the world.  Dr Alan Parker and his team, whose work on cancer is funded by Cancer Research UK, are drawing on their expertise in viruses to seek out “tools” which could be used to deliver a vaccine.  Their work over the past seven years has centred on modiﬁed adenoviruses such as the common cold as viral vectors - or carriers - that can seek out and destroy cancer cells.  Over this time, Dr Parker and his team have a vast bank of different adenoviruses, and now they plan to hunt out - and recommission - the viruses that may potentially be used to deliver a vaccine for coronavirus. | | | | | | | | | | | | | | | |  |
|  |  |  |  |  |  |  |  |  |  |  |  |  |  |  |  |  |  |
|  |  | | | | | | | | | | | | | | | |  |
|  | | | | | | | | | | | | 2 |  | LF |  | 08/03/2021 17:34 |  |
|  | “Our aim is to produce potential vaccines and then pass these on to immunologists to test to see if they are able to induce an immune response that can protect against coronavirus infection,” said Dr Parker.  “As scientists, we’re all wondering how we can usefully contribute. Everyone feels the same. Our role is just a small part of the huge effort that is under way to help ﬁght this virus.”  Dr Parker and his team are already back at work in the labs at a University research building at the University Hospital of Wales - but adhering strictly to social distancing.  “Being back in the lab is really weird. It’s so empty and really quite eerie,” said Dr Parker.  “There are four of us on the team who have now been granted ‘essential worker status’ and we’re obviously having to maintain social distancing so are working apart. It’s very strange.  “But thankfully for us we’re in our comfort zone when it comes to our research. Our expertise is in tinkering with viral vectors for therapeutic beneﬁt. We’ve changed tack slightly - from ﬁghting cancer to infectious disease - but we’re still doing what we’re good at and drawing on what we know.  “It’s not a change of direction I could have predicted a month ago but we’re all hoping it is temporary. | | | | | | | | | | | | | | | |  |
|  |  |  |  |  |  |  |  |  |  |  |  |  |  |  |  |  |  |
|  |  | | | | | | | | | | | | | | | |  |
|  | | | **Files\\80 [15July20]** | | | | | | | | | | | | | |  |
|  |  |  | Yes |  | All articles |  | 0.0068 |  | 1 | |  | | | | | |  |
|  | | |  |  |  |  |  |  |  | |  | | | | | | |
|  | | | | | | | | | | | | 1 |  | LF |  | 08/03/2021 17:48 |  |
|  | Although some cancer researchers have pivoted their research programs toward understanding and containing COVID-19, many have unfortunately been prevented from doing so, as they were sequestered away from their laboratories in efforts to prevent its spread. | | | | | | | | | | | | | | | |  |
|  |  | | | | | | | | | | | | | | | |  |
|  | | | | | | | | | | | | | | | | | |
| Reports\\Coding Summary By Code Report | | | | | | | | | | Page 48 of 123 | | | | | | | |
| 14/07/2021 10:54 | | | | | | | | | | | | | | | | | |
|  | | | **Aggregate** |  | **Classification** |  | **Coverage** |  | **Number Of Coding References** | |  | **Reference Number** |  | **Coded By Initials** |  | **Modified On** |  |
|  | **Nodes\\Direct impacts\COVID measures limiting cancer research activity\Increased 'thinking time'** | | | | | | | | | | | | | | | |  |
|  | | **PDF** | | | | | | | | | | | | | | |  |
|  | | | **Files\\30 (2) [16July20]** | | | | | | | | | | | | | |  |
|  |  |  | No |  | All articles |  | 0.0216 |  | 1 | |  | | | | | |  |
|  | | |  |  |  |  |  |  |  | |  | | | | | | |
|  | | | | | | | | | | | | 1 |  | LF |  | 19/02/2021 15:34 |  |
|  | “We can rethink things, analyze data every which way to see if it’s telling us things we hadn’t seen before,” says Richer. “Having the time to think about your projects – that downtime can really be valuable. It can give you the headspace to think about something in a new way and reevaluate your approach.”  According to Richer, the time to step back and think may lead to new, creative ideas emerging from the pandemic.  “My lab is getting way more thinking time,” agrees Spencer. “They’re reading all the papers they never had time for when they were focused on experimental work.” | | | | | | | | | | | | | | | |  |
|  |  | | | | | | | | | | | | | | | |  |
|  | | | **Files\\34 [13Jan21]** | | | | | | | | | | | | | |  |
|  |  |  | No |  | All articles |  | 0.0146 |  | 1 | |  | | | | | |  |
|  | | |  |  |  |  |  |  |  | |  | | | | | | |
|  | | | | | | | | | | | | 1 |  | LF |  | 19/02/2021 16:57 |  |
|  | “It is quite difficult to accurately estimate how much the Covid-19 pandemic has delayed my research progress. Obtaining accurate data from laboratory experiments that are conducted are the biggest factor driving such setbacks. However, what is in my control is the time spent on completing my firstauthor research paper for publication,” said Geneus. | | | | | | | | | | | | | | | |  |
|  |  | | | | | | | | | | | | | | | |  |
|  | | | **Files\\46 (2) [25Sept20]** | | | | | | | | | | | | | |  |
|  |  |  | No |  | All articles |  | 0.0300 |  | 1 | |  | | | | | |  |
|  | | |  |  |  |  |  |  |  | |  | | | | | | |
|  | | | | | | | | | | | | 1 |  | LF |  | 09/03/2021 11:00 |  |
|  | After Dr. Monje’s lab shutdown and her team was sent home, they had every reason to take a break. However, they also had even bigger reasons to keep progressing forward. Her team pivoted from lab research and used the time for data analysis, research paper writing and also to push two new clinical trials forward. | | | | | | | | | | | | | | | |  |
|  |  | | | | | | | | | | | | | | | |  |
|  | | | | | | | | | | | | | | | | | |
|  | | | | | | | | | | | | | | | | | |
|  | | | | | | | | | | | | | | | | | |
| Reports\\Coding Summary By Code Report | | | | | | | | | | Page 49 of 123 | | | | | | | |
| 14/07/2021 10:54 | | | | | | | | | | | | | | | | | |
|  | | | **Aggregate** |  | **Classification** |  | **Coverage** |  | **Number Of Coding References** | |  | **Reference Number** |  | **Coded By Initials** |  | **Modified On** |  |
|  | **Nodes\\Direct impacts\COVID measures limiting cancer research activity\Increased 'thinking time'\Diminishing returns from 'thinking time'** | | | | | | | | | | | | | | | |  |
|  | | **PDF** | | | | | | | | | | | | | | |  |
|  | | | **Files\\30 (2) [16July20]** | | | | | | | | | | | | | |  |
|  |  |  | No |  | All articles |  | 0.0099 |  | 1 | |  | | | | | |  |
|  | | |  |  |  |  |  |  |  | |  | | | | | | |
|  | | | | | | | | | | | | 1 |  | LF |  | 19/02/2021 15:36 |  |
|  | “Some people are having more focus time, but there’s also a lot lost,” says Ford. “Now we’re six weeks in and at this point, we’re getting diminishing returns from taking time off to think, and it’s time to get back to doing. People are itching to get back into the lab.” | | | | | | | | | | | | | | | |  |
|  |  | | | | | | | | | | | | | | | |  |
|  | **Nodes\\Direct impacts\Disruption to supply chains\Disruption to drug supply chains and demand** | | | | | | | | | | | | | | | |  |
|  | | **Document** | | | | | | | | | | | | | | |  |
|  | | | **Files\\147 [16Oct20]** | | | | | | | | | | | | | |  |
|  |  |  | No |  | All articles |  | 0.0423 |  | 1 | |  | | | | | |  |
|  | | |  |  |  |  |  |  |  | |  | | | | | | |
|  | | | | | | | | | | | | 1 |  | LF |  | 15/02/2021 17:37 |  |
|  | The report underlines the current market data verified and validated by the industry experts. The report is furnished with an updated market view in regards to the currently unfolding COVID-19 pandemic. The impact of the health crisis has been massive on the Cancer Immunotherapy sector with disruption in the supply chains and changes in the trends and demand patterns. | | | | | | | | | | | | | | | |  |
|  |  | | | | | | | | | | | | | | | |  |
|  | **Nodes\\Direct impacts\Disruption to supply chains\Disruption to regulatory activities** | | | | | | | | | | | | | | | |  |
|  | | **Document** | | | | | | | | | | | | | | |  |
|  | | | **Files\\149 [17Nov20]** | | | | | | | | | | | | | |  |
|  |  |  | No |  | All articles |  | 0.0551 |  | 1 | |  | | | | | |  |
|  | | |  |  |  |  |  |  |  | |  | | | | | | |
|  | | | | | | | | | | | | 1 |  | LF |  | 15/02/2021 17:45 |  |
|  | CandidateBristol-Myers Squibb Co (NYSE: BMY[35]) said the FDA has informed it that its review of the BLA for lisocabtagene maraleucel (liso-cel) for the treatment of adults with relapsed or refractory large B-cell lymphoma after at least two prior therapies will not be completed by the PDUFA action date of Nov. 16. This is due to the agency's inability to inspect a third-party manufacturing facility in Texas during the current review cycle due to travel restrictions related to the COVID-19 pandemic. The FDA did not provide a new anticipated action date. | | | | | | | | | | | | | | | |  |
|  |  | | | | | | | | | | | | | | | |  |
|  | | | | | | | | | | | | | | | | | |
| Reports\\Coding Summary By Code Report | | | | | | | | | | Page 50 of 123 | | | | | | | |
| 14/07/2021 10:54 | | | | | | | | | | | | | | | | | |
|  | | | **Aggregate** |  | **Classification** |  | **Coverage** |  | **Number Of Coding References** | |  | **Reference Number** |  | **Coded By Initials** |  | **Modified On** |  |
|  | | **PDF** | | | | | | | | | | | | | | |  |
|  | | | **Files\\55 [25Mar20]** | | | | | | | | | | | | | |  |
|  |  |  | No |  | All articles |  | 0.0587 |  | 1 | |  | | | | | |  |
|  | | |  |  |  |  |  |  |  | |  | | | | | | |
|  | | | | | | | | | | | | 1 |  | LF |  | 08/03/2021 15:03 |  |
|  | For now, it’s unclear what long-term eﬀects the outbreak will have on drug regulation. “There will be a disruption, obviously,” says Bierer. “And whether that delay manifests in delaying ﬁnal approvals is unknowable today.”  It’s that uncertainty that haunts Nizar. She worries that her concerns might sound selﬁsh in the face of the global suﬀering caused by the pandemic. But she also knows that the delay to her clinical trial could last well beyond the months of social isolation and lockdowns.  Her best hope now, she says, is that regulators will learn from the speed and urgency with which a candidate vaccine for the virus that causes COVID-19 has been rushed into clinical trials, forgoing some of the pre-trial animal tests that regulators typically require. Nizar wants to see therapies for rare diseases treated with the same urgency.  “Our lives have always been in panic mode,” she says. “Now the world has a glimpse into what our reality is.” | | | | | | | | | | | | | | | |  |
|  |  |  |  |  |  |  |  |  |  |  |  |  |  |  |  |  |  |
|  |  | | | | | | | | | | | | | | | |  |
|  | **Nodes\\Direct impacts\Forced adaptation of research protocols\Adaptation of regulatory requirements** | | | | | | | | | | | | | | | |  |
|  | | **Document** | | | | | | | | | | | | | | |  |
|  | | | **Files\\272 [8Apr20]** | | | | | | | | | | | | | |  |
|  |  |  | No |  | All articles |  | 0.0251 |  | 1 | |  | | | | | |  |
|  | | |  |  |  |  |  |  |  | |  | | | | | | |
|  | | | | | | | | | | | | 1 |  | LF |  | 17/02/2021 15:57 |  |
|  | Finally, Congress has expanded which hospitals with significant cash flow problems may request accelerated payments. This expansion now includes hospitals with inpatient populations predominantly under the age of 18, hospitals operating a demonstration project, hospitals recognized as comprehensive cancer centers, cancer research centers, or clinical centers, and critical access hospitals. These hospitals may request accelerated payments on a periodic or lump sum basis. | | | | | | | | | | | | | | | |  |
|  |  | | | | | | | | | | | | | | | |  |
|  | | | **Files\\317 [25Oct20]** | | | | | | | | | | | | | |  |
|  |  |  | No |  | All articles |  | 0.1326 |  | 1 | |  | | | | | |  |
|  | | |  |  |  |  |  |  |  | |  | | | | | | |
|  | | | | | | | | | | | | 1 |  | LF |  | 19/02/2021 16:22 |  |
|  | In light of the ongoing COVID-19 pandemic, the HHS Office for Human Research Protections has issued an exception to the single institutional review board policy—provided that the government sponsor agrees and 'where reliance' on an sIRB would not be practical, OHRP announced Oct. 8. The exception applies to cooperative research 'that is ongoing or initially reviewed by the IRB' during the pandemic and that would be in effect 'for the duration of the research.''The COVID-19 public health emergency has created unprecedented burdens and disruption to the research enterprise, while at the same time requiring urgent research responses that necessitate flexible approaches to oversight in order to provide vital information and to allow other research to continue where possible,' OHRP said. 'This exception represents an effort to prioritize the health and safety of both research subjects and investigators, and provides flexibility to institutions in seeking IRB review due to the unique challenges created by the COVID-19 outbreak.' | | | | | | | | | | | | | | | |  |
|  |  |  |  |  |  |  |  |  |  |  |  |  |  |  |  |  |  |
|  |  | | | | | | | | | | | | | | | |  |
|  | | | | | | | | | | | | | | | | | |
| Reports\\Coding Summary By Code Report | | | | | | | | | | Page 51 of 123 | | | | | | | |
| 14/07/2021 10:54 | | | | | | | | | | | | | | | | | |
|  | | | **Aggregate** |  | **Classification** |  | **Coverage** |  | **Number Of Coding References** | |  | **Reference Number** |  | **Coded By Initials** |  | **Modified On** |  |
|  | | | **Files\\4 [10Nov20]** | | | | | | | | | | | | | |  |
|  |  |  | No |  | All articles |  | 0.0265 |  | 1 | |  | | | | | |  |
|  | | |  |  |  |  |  |  |  | |  | | | | | | |
|  | | | | | | | | | | | | 1 |  | LF |  | 07/03/2021 11:51 |  |
|  | While this may all sound challenging, there have been some positive developments in this crisis. Innovative diagnostics[10] and treatments are being fast-tracked into the system, and new ways of working embedded that could help services in the long run. | | | | | | | | | | | | | | | |  |
|  |  | | | | | | | | | | | | | | | |  |
|  | | **PDF** | | | | | | | | | | | | | | |  |
|  | | | **Files\\1 (2) [8th Oct 20]** | | | | | | | | | | | | | |  |
|  |  |  | No |  | All articles |  | 0.0182 |  | 1 | |  | | | | | |  |
|  | | |  |  |  |  |  |  |  | |  | | | | | | |
|  | | | | | | | | | | | | 1 |  | LF |  | 15/02/2021 12:05 |  |
|  | “The relaxation of some of the regulatory requirements associated with clinical research has been accomplished without compromising patient safety,” Bakouny said. “Many investigators see this as a plus — something that could be part of clinical research going forward, to reduce the cost of trials and facilitate the arrival of new therapies for patients.” | | | | | | | | | | | | | | | |  |
|  |  | | | | | | | | | | | | | | | |  |
|  | | | **Files\\24 [14Sept20]** | | | | | | | | | | | | | |  |
|  |  |  | No |  | All articles |  | 0.0637 |  | 3 | |  | | | | | |  |
|  | | |  |  |  |  |  |  |  | |  | | | | | | |
|  | | | | | | | | | | | | 1 |  | LF |  | 17/02/2021 14:31 |  |
|  | The US Food and Drug Administration recognized early on that patients wouldn't be able to travel to clinical trial sites due to lockdown rules, and in March issued guidance allowing protocol adjustments that would allow trials to decentralize. The agency told sponsors to consider conducting drug safety evaluations via phone or use labs and imaging centers closer to patients for response monitoring. For drugs that must be administered in a healthcare setting, the agency was open to nurses or trained non-medical personnel administering them to patients at home.  The Centers for Medicare & Medicaid Services also expanded reimbursement for telehealth services and said states could waive in-state licensure requirements for physicians seeing Medicare patients remotely and across state lines, via telemedicine.  These emergency guidelines allowed cancer researchers to quickly pivot to telemedicine and remote monitoring strategies for some trials. According to the ASCO survey, more than 90 percent of respondents said they reviewed patients' symptoms using remote strategies and nearly 88 percent said they engaged with patients via telemedicine whenever possible. | | | | | | | | | | | | | | | |  |
|  |  |  |  |  |  |  |  |  |  |  |  |  |  |  |  |  |  |
|  |  | | | | | | | | | | | | | | | |  |
|  | | | | | | | | | | | | 2 |  | LF |  | 17/02/2021 14:37 |  |
|  | Pandemic changes here to stay?  From March to May, Roychowdhury was using telemedicine to check in with almost all his cancer patients. In September, he is using telemedicine to see between 5 percent and 15 percent of patients because quarantine and safety restrictions are still in place in some cases. Importantly, during the critical months of the pandemic, he was able to keep up with all his patients enrolled in an active study via telemedicine thanks to emergency amendments and cooperation from the drug industry.  Flexibility from regulators and payors also helped. "The pandemic happened and literally overnight [CMS] said it will reimburse telemedicine visits, which clearly proves that the infrastructure was all there," said Camidge. "There had to be some motivation to actually do it." | | | | | | | | | | | | | | | |  |
|  |  |  |  |  |  |  |  |  |  |  |  |  |  |  |  |  |  |
|  |  | | | | | | | | | | | | | | | |  |
|  | | | | | | | | | | | | 3 |  | LF |  | 17/02/2021 14:39 |  |
|  | Based on guidance from CMS, states have also been allowed to waive physician state-licensing requirements during the pandemic so they can provide telemedicine to patients residing in other states. This is particularly important in the cancer clinical trial setting, because inter-state travel is so common. As of September, 46 states have waived state licensing requirements for doctors providing telemedicine, though some of these waivers are slated to expire soon and commercial insurers' coverage requirements vary. As such, hospitals may approach telemedicine with more caution from a liability or revenue standpoint. | | | | | | | | | | | | | | | |  |
|  |  | | | | | | | | | | | | | | | |  |
| Reports\\Coding Summary By Code Report | | | | | | | | | | Page 52 of 123 | | | | | | | |
| 14/07/2021 10:54 | | | | | | | | | | | | | | | | | |
|  | | | **Aggregate** |  | **Classification** |  | **Coverage** |  | **Number Of Coding References** | |  | **Reference Number** |  | **Coded By Initials** |  | **Modified On** |  |
|  | | | **Files\\5 [29Jun20]** | | | | | | | | | | | | | |  |
|  |  |  | No |  | All articles |  | 0.0756 |  | 3 | |  | | | | | |  |
|  | | |  |  |  |  |  |  |  | |  | | | | | | |
|  | | | | | | | | | | | | 1 |  | LF |  | 08/03/2021 10:34 |  |
|  | For telehealth and virtual research visits to continue beyond the pandemic, said Mutz, some of the temporary changes that had been made to regulations on the use of telehealth during COVID-19 would need to become permanent. | | | | | | | | | | | | | | | |  |
|  |  | | | | | | | | | | | | | | | |  |
|  | | | | | | | | | | | | 2 |  | LF |  | 08/03/2021 10:36 |  |
|  | In the past, such changes might have been viewed as violations of study protocols. But, in March, NCI provided guidance for clinical trial activities affected by the coronavirus. The Food and Drug Administration also issued a guidance on clinical trials of medical products during the pandemic.  Laden with backpacks filled with medical supplies, (from left) St. Jude nurses Shelly Veloz, Shana Ramey, and Teresa Neal head to their next appointment at patient housing. Credit: St. Jude Children's Research Hospital  “We have been closely monitoring NCI-sponsored clinical trials to understand how we can make accommodations so that patients can receive care while the clinical trial investigators can, as much as possible, continue the studies,” said Meg Mooney, M.D., of NCI’s Cancer Therapy Evaluation Program.  NCI has given trial sites flexibility in the operations of trials, including in the timing of when patient tests and assessments must be done.  “Investigators leading clinical trials can work with the Institutional Review Board that oversees each study protocol to make necessary adjustments,” Dr. Mooney explained. “These modifications will provide flexibility without compromising patient safety or the validity of the data being collected by the trial.” | | | | | | | | | | | | | | | |  |
|  |  |  |  |  |  |  |  |  |  |  |  |  |  |  |  |  |  |
|  |  | | | | | | | | | | | | | | | |  |
|  | | | | | | | | | | | | 3 |  | LF |  | 08/03/2021 11:14 |  |
|  | Clinical trials are monitored to assure the quality of the data being collected by researchers and compliance with regulations for clinical research. Before the pandemic, monitors would typically travel to trial locations  and use on-site computers to review data from the studies, such as patient electronic medical records (EMRs).  In response to the pandemic, however, NCI’s Cancer Therapy Evaluation Program has begun to adopt remote approaches to monitoring studies.  “Before COVID-19, only a handful of institutions running NCI-sponsored trials would allow auditors to access EMRs remotely,” said Gary L. Smith, who leads NCI’s Clinical Trials Monitoring Branch, which sets guidelines and standards for the conduct of NCI-sponsored clinical trials. “But we are definitely seeing more and more remote audits.”  Now that most medical records are electronic and can be accessed from a computer anywhere, “it doesn’t make sense to me to have monitors traveling to sites,” Smith said. “I don’t think we can completely replace on-site audits, but I think we can augment these audits with remote monitoring approaches.”  Some sites have recently been allowing auditors to visit in person, he noted. But trial sites have set up facilities within their institutions that are apart from patients to reduce the possible spread of COVID-19.  “Our main concern,” Smith said, “has always been the safety of the monitors who have to travel to the sites and the safety of patients in trials who might be exposed to the coronavirus by an asymptomatic but infected monitor.” | | | | | | | | | | | | | | | |  |
|  |  |  |  |  |  |  |  |  |  |  |  |  |  |  |  |  |  |
|  |  |  |  |  |  |  |  |  |  |  |  |  |  |  |  |  |  |
|  |  | | | | | | | | | | | | | | | |  |
|  | | | **Files\\55 [25Mar20]** | | | | | | | | | | | | | |  |
|  |  |  | No |  | All articles |  | 0.0824 |  | 1 | |  | | | | | |  |
|  | | |  |  |  |  |  |  |  | |  | | | | | | |
|  | | | | | | | | | | | | 1 |  | LF |  | 08/03/2021 15:02 |  |
|  | Government agencies have released guidance for investigators who need to suspend or modify trials. The US Food and Drug Administration, for example, has issued guidance for trials that might have to pause, change their study plans or make do with incomplete data because of the COVID-19 pandemic. Ethics committees are working overtime as researchers ﬁle requests to alter their clinical-trial plans in ways that minimize how often participants need to venture into the clinic, says Barbara Bierer, who directs the Multi-Regional Clinical Trials Center of Brigham and Women’s Hospital and Harvard in Boston, Massachusetts.  RELATED  Agencies and clinical-trial funders have shown remarkable ﬂexibility, says Charles Blanke, an oncologist at Oregon Health & Science University in Portland and leader of the publicly funded SWOG Cancer Research Network. The US National Cancer Institute announced on 23 March that it would allow the investigators it funds to assess trial participants' health remotely where possible. Some doctors’ assessments may be carried out over video calls instead of in person, and some audits of clinical-  Why does the coronavirus spread so easily between people?  trial procedures will be conducted virtually, with inspectors examining the paperwork online rather than physically visiting the clinic to assess standards. | | | | | | | | | | | | | | | |  |
|  |  |  |  |  |  |  |  |  |  |  |  |  |  |  |  |  |  |
|  |  | | | | | | | | | | | | | | | |  |
| Reports\\Coding Summary By Code Report | | | | | | | | | | Page 53 of 123 | | | | | | | |
| 14/07/2021 10:54 | | | | | | | | | | | | | | | | | |
|  | | | **Aggregate** |  | **Classification** |  | **Coverage** |  | **Number Of Coding References** | |  | **Reference Number** |  | **Coded By Initials** |  | **Modified On** |  |
|  | | | **Files\\74 [14Sept20]** | | | | | | | | | | | | | |  |
|  |  |  | No |  | All articles |  | 0.0215 |  | 1 | |  | | | | | |  |
|  | | |  |  |  |  |  |  |  | |  | | | | | | |
|  | | | | | | | | | | | | 1 |  | LF |  | 08/03/2021 17:29 |  |
|  | “We have also benefited from coordination across the different UK healthcare agencies. When Novartis identified the potential for one of its cancer medicines, ruxolitinib, to treat COVID-19-related pneumonia, the efficient collaboration between the MHRA, the UK Ethics Committee, the NHS, the NIHR and the hospitals enabled a clinical trial to start in an unprecedented three weeks. For a process with an average time to approval of 12 weeks, the UK was the first country, globally, to achieve first patient first visit and first patient first treatment milestones for the Phase 3 RUXCOVID trial. The learnings from this record success will be key for future trials.” | | | | | | | | | | | | | | | |  |
|  |  | | | | | | | | | | | | | | | |  |
|  | **Nodes\\Direct impacts\Forced adaptation of research protocols\Adaptation of regulatory requirements\Adaptation of cancer care protocols** | | | | | | | | | | | | | | | |  |
|  | | **Document** | | | | | | | | | | | | | | |  |
|  | | | **Files\\258 [26June20]** | | | | | | | | | | | | | |  |
|  |  |  | No |  | All articles |  | 0.1058 |  | 1 | |  | | | | | |  |
|  | | |  |  |  |  |  |  |  | |  | | | | | | |
|  | | | | | | | | | | | | 1 |  | LF |  | 17/02/2021 15:18 |  |
|  | The Institute of Cancer Research, London, has expressed disappointment at the decision by NICE not to recommend abiraterone as a first-line treatment on the NHS for newly diagnosed, advanced prostate cancer. The decision puts the NHS in England and Wales out of step with Scotland, where the drug was approved as a first-line treatment at the start of this year. Abiraterone was also made available first line on an interim basis for patients who aren't suitable for enzalutamide as part of NHS England guidance in May in response to the COVID-19 pandemic, to spare some men with prostate cancer[1] the need for chemotherapy in hospital. | | | | | | | | | | | | | | | |  |
|  |  | | | | | | | | | | | | | | | |  |
|  | | | **Files\\312 [6May20]** | | | | | | | | | | | | | |  |
|  |  |  | No |  | All articles |  | 0.7094 |  | 2 | |  | | | | | |  |
|  | | |  |  |  |  |  |  |  | |  | | | | | | |
|  | | | | | | | | | | | | 1 |  | LF |  | 19/02/2021 16:13 |  |
|  | The Institute of Cancer Research, London, has welcomed approval of targeted hormone therapies enzalutamide and abiraterone as first-line NHS treatments for men with advanced prostate cancer.Standard treatment for men first diagnosed with advanced prostate cancer is hormone therapy - either as monotherapy or together with docetaxel chemotherapy.Docetaxel is generally given as six three-weekly infusions in hospital and can significantly weaken patients' immune system and cause inflammation of the lungs - putting men at risk during the current COVID-19 pandemic, ICR experts note.They say treatment with enzalutamide or abiraterone can not only prevent patients having to receive docetaxel chemotherapy but is also given as tablets which men can take at home - avoiding unnecessary pressures on the NHS.NHS England has now updated its interim guidance on treatment change options during the COVID-19 pandemic to offer enzalutamide for men with newly diagnosed, advanced prostate cancer, and abiraterone for patients who cannot tolerate enzalutamide.The move follows pressure from experts to widen access to abiraterone during the novel coronavirus pandemic, given that first-line treatment with the drug is available on the NHS in Scotland, and data show survival and quality of life improvements over hormone therapy/docetaxel.'Offering enzalutamide or abiraterone to men as first-line treatment for prostate cancer will greatly lower the risk of exposing vulnerable patients to the coronavirus, and lightens the load on our hard-pressed hospitals. | | | | | | | | | | | | | | | |  |
|  |  |  |  |  |  |  |  |  |  |  |  |  |  |  |  |  |  |
|  |  | | | | | | | | | | | | | | | |  |
|  | | | | | | | | | | | | | | | | | |
| Reports\\Coding Summary By Code Report | | | | | | | | | | Page 54 of 123 | | | | | | | |
| 14/07/2021 10:54 | | | | | | | | | | | | | | | | | |
|  | | | **Aggregate** |  | **Classification** |  | **Coverage** |  | **Number Of Coding References** | |  | **Reference Number** |  | **Coded By Initials** |  | **Modified On** |  |
|  | | | | | | | | | | | | | | | | | |
|  | | | | | | | | | | | | 2 |  | LF |  | 19/02/2021 16:13 |  |
|  | Men can take their tablets at home and have their bloods checked by their GP - and unlike chemotherapy enzalutamide and abiraterone have no significant effects on patients' immune system,' said Professor Nick James, Professor of prostate and bladder cancer research at the ICR, and consultant clinical oncologist at The Royal Marsden NHS Foundation Trust.'It is however frustrating that during this anxious lockdown period it has taken so many weeks to agree extended access to targeted hormone therapies in place of chemotherapy, and that NHS England has chosen to focus on enzalutamide as the initial therapy rather than leaving clinicians to decide on an individual patient basis.'It's becoming increasingly clear that the COVID-19 pandemic will have a long-term impact on the way we live our lives, and I would urge NHS regulators to consider other areas in which cancer care can be modernised, to move towards managing patients at home where possible.'Abiraterone | | | | | | | | | | | | | | | |  |
|  |  |  |  |  |  |  |  |  |  |  |  |  |  |  |  |  |  |
|  |  | | | | | | | | | | | | | | | |  |
|  | | | **Files\\32 [29May20]** | | | | | | | | | | | | | |  |
|  |  |  | No |  | All articles |  | 0.2182 |  | 1 | |  | | | | | |  |
|  | | |  |  |  |  |  |  |  | |  | | | | | | |
|  | | | | | | | | | | | | 1 |  | LF |  | 19/02/2021 16:25 |  |
|  | "This initial report defines some of the major risk factors and outcomes for certain patient subsets, and several other CCC19 projects are ongoing to further expand this knowledge with the goal to inform cancer patients and providers." The other senior authors are Warner and Gary H. Lyman, MD, MPH, professor of Medicine-Oncology at the University of Washington. These early data showed no statistical association between 30-day mortality[1] and cancer treatments, suggesting that surgery, adjuvant chemotherapy[2] and maintenance chemotherapy could continue during the pandemic with "extreme caution." "While older patients and those with major comorbid conditions are at substantially increased risk of dying from COVID-19, our early findings are encouraging news for patients without major medical conditions who receive their cancer therapy within four weeks of their infection. However, more data are needed to reliably assess individual higher risk therapies," said Nicole Kuderer, MD, with the Advanced Cancer Research Group in Seattle, one of the study's lead authors. | | | | | | | | | | | | | | | |  |
|  |  |  |  |  |  |  |  |  |  |  |  |  |  |  |  |  |  |
|  |  | | | | | | | | | | | | | | | |  |
|  | | | **Files\\36 [18Aug20]** | | | | | | | | | | | | | |  |
|  |  |  | No |  | All articles |  | 0.0806 |  | 1 | |  | | | | | |  |
|  | | |  |  |  |  |  |  |  | |  | | | | | | |
|  | | | | | | | | | | | | 1 |  | LF |  | 23/02/2021 15:15 |  |
|  | Many respondents said that their treatment may have been modified instead of totally canceled, the researchers said. One example they noted was that hormone therapies may have been spaced out more to reduce the number of clinic visits. "Overall, we see that there is a serious gap in disaster preparedness[6] when it comes to providing critical, and often time-sensitive care for breast cancer patients," Hamlish said. | | | | | | | | | | | | | | | |  |
|  |  | | | | | | | | | | | | | | | |  |
|  | | | **Files\\398 [26Jun20]** | | | | | | | | | | | | | |  |
|  |  |  | No |  | All articles |  | 0.6613 |  | 2 | |  | | | | | |  |
|  | | |  |  |  |  |  |  |  | |  | | | | | | |
|  | | | | | | | | | | | | 1 |  | LF |  | 07/03/2021 11:24 |  |
|  | NICE will not be recommending Janssen's Zytiga (abiraterone acetate) with prednisone or prednisolone plus androgen deprivation therapy (ADT), within its marketing authorisation, for treating newly diagnosed high-risk metastatic hormone-sensitive prostate cancer (mHSPC) in adults.Around 4,000 men newly diagnosed with high-risk metastatic hormone-sensitive prostate cancer (mHSPC) would have been eligible for treatment abiraterone acetate in combination with prednisone/prednisolone and ADT.Currently, men first diagnosed with advanced prostate cancer are treated with hormone therapy with docetaxel chemotherapy, or hormone therapy on its own, but because chemotherapy can come with serious side effects, around three quarters are unable to tolerate it or choose not to have it.NICE's rejection of NHS funds for the drug in England and Wales highlights the postcode lottery of care that exists across the UK, as the Scottish Medicines Consortium (SMC) has approved use of the drug in this setting, based on the same evidence base.The Final Appraisal Document (FAD) also seems to be in direct contrast to interim guidance issued by NHS England last month, which recognised Zytiga as an alternative option by agreeing to provide it for an interim period to those who are unable to have chemotherapy due to the current COVID-19 pandemic.The Institute of Cancer Research (ICR) has expressed disappointment with the decision, and is now calling for NHS England and drug's manufacturer, Janssen, to come to an agreement on pricing that would allow its use on the NHS. | | | | | | | | | | | | | | | |  |
|  |  |  |  |  |  |  |  |  |  |  |  |  |  |  |  |  |  |
|  |  | | | | | | | | | | | | | | | |  |
|  | | | | | | | | | | | | | | | | | |
| Reports\\Coding Summary By Code Report | | | | | | | | | | Page 55 of 123 | | | | | | | |
| 14/07/2021 10:54 | | | | | | | | | | | | | | | | | |
|  | | | **Aggregate** |  | **Classification** |  | **Coverage** |  | **Number Of Coding References** | |  | **Reference Number** |  | **Coded By Initials** |  | **Modified On** |  |
|  | | | | | | | | | | | | | | | | | |
|  | | | | | | | | | | | | 2 |  | LF |  | 07/03/2021 11:25 |  |
|  | ''I would urge the manufacturer of abiraterone and NHS England to urgently negotiate a suitable patient access scheme - the STAMPEDE and LATITUDE trial results were published in 2017 so this is long overdue. This would allow many men with advanced prostate cancer in England and Wales to be offered abiraterone from the outset and live well with their cancer for as long as possible.'Sarah Scanlon, business unit director Oncology, Janssen-Cilag Limited, said: 'We are extremely disappointed with this decision, and particularly for those patients and clinicians who were anticipating access to this innovative therapy."It is particularly surprising in light of the recent decision by NHS England which, due to the current COVID-19 pandemic, has recognised abiraterone acetate as an alternative option by agreeing to provide it for an interim period to those who are intolerant to other treatments."We are currently reviewing the FAD in detail and we will assess our options for a potential challenge to this decision, particularly given the evidence supporting the value of abiraterone in patients who are ineligible for chemotherapy.' | | | | | | | | | | | | | | | |  |
|  |  |  |  |  |  |  |  |  |  |  |  |  |  |  |  |  |  |
|  |  | | | | | | | | | | | | | | | |  |
|  | | | **Files\\4 [10Nov20]** | | | | | | | | | | | | | |  |
|  |  |  | No |  | All articles |  | 0.0265 |  | 1 | |  | | | | | |  |
|  | | |  |  |  |  |  |  |  | |  | | | | | | |
|  | | | | | | | | | | | | 1 |  | LF |  | 07/03/2021 11:51 |  |
|  | While this may all sound challenging, there have been some positive developments in this crisis. Innovative diagnostics[10] and treatments are being fast-tracked into the system, and new ways of working embedded that could help services in the long run. | | | | | | | | | | | | | | | |  |
|  |  | | | | | | | | | | | | | | | |  |
|  | | | **Files\\46 [17Apr20]** | | | | | | | | | | | | | |  |
|  |  |  | No |  | All articles |  | 0.3228 |  | 1 | |  | | | | | |  |
|  | | |  |  |  |  |  |  |  | |  | | | | | | |
|  | | | | | | | | | | | | 1 |  | LF |  | 07/03/2021 12:08 |  |
|  | National Cancer Institute, National Institutes of Health In Nature Medicine, the seven comprehensive cancer centers of Cancer Core Europe (CCE), including the Netherlands Cancer Institute and Cancer Research UK Cambridge center, have shared how they rapidly reorganised their oncological healthcare systems during the COVID-19 pandemic. In this joint effort, the centers offer guidance to institutions globally by outlining their general consensus measures and organisational strategies adopted to make their operations "pandemic proof."  In the space of just a few weeks, the European cancer[1] centers have had to drastically revise and reorganise their patient care[2] and scientific research due to the coronavirus crisis. For example, treatments have been postponed or adjusted to protect the immune systems of patients with cancer; contact moments have been limited or converted to remote interactions, complete with as many guarantees as possible that this will not affect patient care; and clinical studies[3] have been reassessed to see if they should continue. CCE's seven cancer centers have taken steps to maintain high standards of care despite facing shortages of personal protective equipment, beds, staff and other resources. "In this situation the centers have not, as they're used to, been able to rely upon the knowledge they've built up over years of thorough research," said Emile Voest, Medical Director of the Netherlands Cancer Institute. "Our medical staff suddenly find themselves in a parallel universe, where they have to use all their know-how, experience and wisdom to make critical clinical decisions in line with new medical and ethical considerations. That's a drastic change. And why it's essential that other medical institutions can draw upon our experiences and choices so that we all become 'pandemic-proof' in a responsible manner. Not least with an eye to the future." | | | | | | | | | | | | | | | |  |
|  |  |  |  |  |  |  |  |  |  |  |  |  |  |  |  |  |  |
|  |  |  |  |  |  |  |  |  |  |  |  |  |  |  |  |  |  |
|  |  | | | | | | | | | | | | | | | |  |
|  | | | **Files\\47 [7Mar20]** | | | | | | | | | | | | | |  |
|  |  |  | No |  | All articles |  | 0.2862 |  | 1 | |  | | | | | |  |
|  | | |  |  |  |  |  |  |  | |  | | | | | | |
|  | | | | | | | | | | | | 1 |  | LF |  | 07/03/2021 16:55 |  |
|  | The NHS is working tirelessly and has been forced to make difficult decisions during an unprecedented time. They have committed to prioritising cancer care, but Cancer Research UK is concerned that the NHS will not be able to cope with the large backlog of cancer care needed as services reopen, unless steps are taken to plan for the future, including COVID-19 testing. The NHS in England is developing 'COVID-free' centres and hospitals which will be the key to ensuring that the most urgent cancer patients can receive their treatment quickly, but this will only be possible with widescale and frequent testing of NHS staff and patients. Although some patients are being tested for COVID-19, testing in some areas is reserved for those who are seriously ill. The concern is that a number of people with the virus may be infectious to others before they show symptoms, so for a hospital to be truly safe to treat cancer patients, we need rapid testing available for all staff and patients, whether showing symptoms of COVID-19 or not, occurring on a routine and repetitive basis. NHS staff testing for COVID-19 has been boosted by repurposing the Francis Crick Institute's laboratory facilities as a testing facility, to help combat the spread of infection and allow healthcare workers to perform lifesaving duties while remaining safe. The institute, as well as other locations around the country, could accept even more samples for testing, to contribute towards the national COVID screening effort. | | | | | | | | | | | | | | | |  |
|  |  |  |  |  |  |  |  |  |  |  |  |  |  |  |  |  |  |
|  |  | | | | | | | | | | | | | | | |  |
| Reports\\Coding Summary By Code Report | | | | | | | | | | Page 56 of 123 | | | | | | | |
| 14/07/2021 10:54 | | | | | | | | | | | | | | | | | |
|  | | | **Aggregate** |  | **Classification** |  | **Coverage** |  | **Number Of Coding References** | |  | **Reference Number** |  | **Coded By Initials** |  | **Modified On** |  |
|  | | | **Files\\60 [29Apr20]** | | | | | | | | | | | | | |  |
|  |  |  | No |  | All articles |  | 0.1678 |  | 2 | |  | | | | | |  |
|  | | |  |  |  |  |  |  |  | |  | | | | | | |
|  | | | | | | | | | | | | 1 |  | LF |  | 08/03/2021 15:30 |  |
|  | A one-week course of radiotherapy in fewer but larger daily doses was found to be as safe and effective as standard three-week therapy for women following surgery for early stage breast cancer. The protocol is being eagerly sought by hospitals to help reduce demands on the NHS during the COVID-19 pandemic. Women with early stage breast[1] cancer can be treated with fewer but larger daily doses of radiotherapy[2] delivered in a shorter overall duration compared with the current standard, research finds. A pioneering study involving more than 4,000 patients evaluated the effectiveness of two different radiotherapy doses each delivered over five days in one week compared with standard radiotherapy currently delivered in 15 doses over three weeks. | | | | | | | | | | | | | | | |  |
|  |  | | | | | | | | | | | | | | | |  |
|  | | | | | | | | | | | | 2 |  | LF |  | 08/03/2021 15:31 |  |
|  | Professor Judith Bliss, Professor of Clinical Trials at The Institute of Cancer Research, London, and Director of its Clinical Trials and Statistics Unit, and joint senior author of the study, said: "We're always looking for ways to refine and enhance cancer treatment so we can make it more effective and improve the experience for patients. No one would want to come up to hospital for three weeks of radiotherapy if they can get the same benefit in just one week. "We expect these findings will be incorporated into breast cancer treatment guidelines around the world and we're already seeing NHS hospitals wanting to move to the 5-dose schedule because of the challenges they're facing during the coronavirus pandemic." | | | | | | | | | | | | | | | |  |
|  |  | | | | | | | | | | | | | | | |  |
|  | | | **Files\\63 [6Aug20]** | | | | | | | | | | | | | |  |
|  |  |  | No |  | All articles |  | 0.2206 |  | 1 | |  | | | | | |  |
|  | | |  |  |  |  |  |  |  | |  | | | | | | |
|  | | | | | | | | | | | | 1 |  | LF |  | 08/03/2021 15:41 |  |
|  | A new breast cancer study brings reassuring findings for women with early-stage breast cancer who were forced to delay their cancer operations because of the Coronavirus Disease 2019 (COVID-19) pandemic. A longer time from diagnosis to surgical treatment does not lower overall survival of women with early-stage breast cancer who underwent delayed operations before the pandemic, according to the study results, which are published as an "article in press" on the Journal of the American College of Surgeons website in advance of print. The researchers also found no survival decrease with operative delays in women with estrogen-sensitive, early-stage breast[1] cancer who received neoadjuvant endocrine therapy (NET). NET is preoperative hormone treatment with anti-estrogen drugs such as tamoxifen or aromatase inhibitors. Patients with estrogen receptor (ER)-positive breast cancer, whose cancer is fueled by estrogen, typically receive anti-estrogen therapy after surgical removal of the tumor or breast.  However, endocrine therapy was recommended nationwide as the initial treatment of ER-positive breast cancer during pandemic-related surgical delays, said lead study author Christina Minami, MD, MS, an associate surgeon at Brigham and Women's Hospital, Boston. "Usually we take these patients with very small tumors directly to surgery, so it is a big change in practice to first put those patients on tamoxifen or an aromatase inhibitor," Dr. Minami said. "What we can say from our findings is that despite the delay[2] in surgical therapy, because you were on neoadjuvant endocrine therapy, we do not think that your survival will at all be impacted." | | | | | | | | | | | | | | | |  |
|  |  |  |  |  |  |  |  |  |  |  |  |  |  |  |  |  |  |
|  |  | | | | | | | | | | | | | | | |  |
|  | | | **Files\\64 [21Jul20]** | | | | | | | | | | | | | |  |
|  |  |  | No |  | All articles |  | 0.0888 |  | 1 | |  | | | | | |  |
|  | | |  |  |  |  |  |  |  | |  | | | | | | |
|  | | | | | | | | | | | | 1 |  | LF |  | 08/03/2021 16:13 |  |
|  | "We know that complex surgery has been affected, but right now we don't have the evidence to suggest how this will affect outcomes, especially with other treatment options being used," says Shelton. "For some slow growing tumors, there may not be much difference in outcomes, but for other cancer sites, outcomes could vary much more." The study didn't account for the fact that some people who would usually have surgery have had radiotherapy instead, to reduce the size of the tumor until it's safe to operate. Shelton says that the impact of these changes for some patients may be more to do with side effects or quality of life, rather than survival. Something that a single figure of excess deaths will never be able to encapsulate. | | | | | | | | | | | | | | | |  |
|  |  | | | | | | | | | | | | | | | |  |
|  | | | | | | | | | | | | | | | | | |
| Reports\\Coding Summary By Code Report | | | | | | | | | | Page 57 of 123 | | | | | | | |
| 14/07/2021 10:54 | | | | | | | | | | | | | | | | | |
|  | | | **Aggregate** |  | **Classification** |  | **Coverage** |  | **Number Of Coding References** | |  | **Reference Number** |  | **Coded By Initials** |  | **Modified On** |  |
|  | | **PDF** | | | | | | | | | | | | | | |  |
|  | | | **Files\\30 (2) [16July20]** | | | | | | | | | | | | | |  |
|  |  |  | No |  | All articles |  | 0.0157 |  | 1 | |  | | | | | |  |
|  | | |  |  |  |  |  |  |  | |  | | | | | | |
|  | | | | | | | | | | | | 1 |  | LF |  | 19/02/2021 15:35 |  |
|  | According to Schulick, COVID-19 has also helped doctors and researchers learn new ways of communicating and delivering care that may have benefits long after the pandemic ends.  “Overnight, we’ve learned how to do things remotely. I’m seeing patients from very remote places and it’s almost as good as if they had driven 10 hours, stayed in a hotel, saw me for a short appointment, and then drove back 10 hours,” Schulick says. | | | | | | | | | | | | | | | |  |
|  |  | | | | | | | | | | | | | | | |  |
|  | | | **Files\\5 [29Jun20]** | | | | | | | | | | | | | |  |
|  |  |  | No |  | All articles |  | 0.0090 |  | 1 | |  | | | | | |  |
|  | | |  |  |  |  |  |  |  | |  | | | | | | |
|  | | | | | | | | | | | | 1 |  | LF |  | 08/03/2021 11:15 |  |
|  | During the pandemic, Dr. Small and his colleagues at UCSF dramatically changed their approach to treating patients with cancer, including those in clinical trials. Care decisions were driven, he explained, by whether a health care professional deemed a patient’s treatment essential. Such patients continued to come into the hospital and to enroll on clinical trials. | | | | | | | | | | | | | | | |  |
|  |  | | | | | | | | | | | | | | | |  |
|  | **Nodes\\Direct impacts\Forced adaptation of research protocols\Adaptation of regulatory requirements\Adaptation of clinical trials** | | | | | | | | | | | | | | | |  |
|  | | **Document** | | | | | | | | | | | | | | |  |
|  | | | **Files\\190 [26Mar20]** | | | | | | | | | | | | | |  |
|  |  |  | No |  | All articles |  | 0.3043 |  | 1 | |  | | | | | |  |
|  | | |  |  |  |  |  |  |  | |  | | | | | | |
|  | | | | | | | | | | | | 1 |  | LF |  | 17/02/2021 11:22 |  |
|  | BriaCell Therapeutics announces that the Phase I/IIa clinical study of Bria-IMT™ with Incyte Corporation's immune checkpoint inhibitor, is ongoing. Phase I/IIa clinical study of BriaCell's lead candidate, Bria-IMT™, with Incyte Corporation's immune checkpoint inhibitor, INCMGA00012, is ongoing and recruiting patients amidst the COVID-19 pandemic. Clinical and immune activity of Bria-IMT™, alone or in combination with checkpoint inhibitors, KEYTRUDAand INCMGA00012, in advanced breast cancer will be presented at the American Association for Cancer Research (AACR) Annual Meeting, postponed to August 2020. BriaCell Therapeutics Corp.[1] ('BriaCell' or the 'Company') (TSXV:BCT[2], OTCQB:BCTXF), a clinical-stage biotechnology company specializing in targeted immunotherapy for advanced breast cancer, announce that the Phase I/IIa clinical study of Bria-IMT™ with Incyte Corporation's immune checkpoint inhibitor, INCMGA00012 is ongoing and recruiting patients amidst the COVID-19 pandemic.  In fact, BriaCell has enrolled a new patient in the study this week. 'We are determined to provide safe and effective treatments for advanced breast cancer patients with no effective treatment options. Our experts believe that our novel immunotherapy may work additively or synergistically with checkpoint inhibitors to boost the body's immune response to cancer cells and we are firmly committed to continuing our scientific and clinical advancements,' said Dr. Bill Williams, BriaCell's President & CEO. 'BriaCell's clinical trial is ongoing throughout the COVID-19 pandemic. We are confident that the participating clinical sites in BriaCell's clinical trial are fully compliant with state-specific regulations, and will take every appropriate measure to meet and exceed safety standards for their patients amid the COVID-19 pandemic.' | | | | | | | | | | | | | | | |  |
|  |  |  |  |  |  |  |  |  |  |  |  |  |  |  |  |  |  |
|  |  |  |  |  |  |  |  |  |  |  |  |  |  |  |  |  |  |
|  |  | | | | | | | | | | | | | | | |  |
|  | | | | | | | | | | | | | | | | | |
| Reports\\Coding Summary By Code Report | | | | | | | | | | Page 58 of 123 | | | | | | | |
| 14/07/2021 10:54 | | | | | | | | | | | | | | | | | |
|  | | | **Aggregate** |  | **Classification** |  | **Coverage** |  | **Number Of Coding References** | |  | **Reference Number** |  | **Coded By Initials** |  | **Modified On** |  |
|  | | **PDF** | | | | | | | | | | | | | | |  |
|  | | | **Files\\1 (2) [8th Oct 20]** | | | | | | | | | | | | | |  |
|  |  |  | No |  | All articles |  | 0.0204 |  | 1 | |  | | | | | |  |
|  | | |  |  |  |  |  |  |  | |  | | | | | | |
|  | | | | | | | | | | | | 1 |  | LF |  | 15/02/2021 12:05 |  |
|  | Despite these challenges, investigators found a variety of ways to adapt to straitened circumstances so trials could continue. These included leveraging telehealth to limit in-person visits, use of e-signatures for trial documentation, shipping oral medications to trial participants rather than requiring them to be picked up at the clinic, and allowing laboratory tests to be done at outside labs. | | | | | | | | | | | | | | | |  |
|  |  | | | | | | | | | | | | | | | |  |
|  | | | **Files\\10 [21 Oct 20]** | | | | | | | | | | | | | |  |
|  |  |  | No |  | All articles |  | 0.0307 |  | 2 | |  | | | | | |  |
|  | | |  |  |  |  |  |  |  | |  | | | | | | |
|  | | | | | | | | | | | | 1 |  | LF |  | 15/02/2021 12:18 |  |
|  | Above all, the risk of exposing vulnerable cancer patients to the coronavirus during visits to health facilities had to be considered, he added. That calculation allowed for his trials for bowel cancer treatment to continue.  “We needed to assess … where the balance of safety versus risk was in favor of the treatment,” he said. “It's very clear that not being able to cure this cancer can actually have very, very severe consequences for patients.” | | | | | | | | | | | | | | | |  |
|  |  | | | | | | | | | | | | | | | |  |
|  | | | | | | | | | | | | 2 |  | LF |  | 15/02/2021 12:19 |  |
|  | Similarly, Cameron said, one of his breast cancer trials was able to continue despite the pandemic. It was just a matter of adapting practices to the restrictions of the virus.  “Let's be creative, without changing the quality of what we do,” he said, pointing to the example of the adoption of telemedicine for consultations that were previously done in person. | | | | | | | | | | | | | | | |  |
|  |  | | | | | | | | | | | | | | | |  |
|  | | | **Files\\118 [1 May 20]** | | | | | | | | | | | | | |  |
|  |  |  | No |  | All articles |  | 0.0718 |  | 2 | |  | | | | | |  |
|  | | |  |  |  |  |  |  |  | |  | | | | | | |
|  | | | | | | | | | | | | 1 |  | LF |  | 15/02/2021 15:30 |  |
|  | As the virus spread around the world, Lilly moved very quickly to protect our employees by restricting travel and practicing social distancing. Additionally, we moved very early to a work from home policy for almost everyone but those who manufacture and maintain the supply of our medicines. This was done yes, to reduce the risk of spreading the virus, yes, to protect our employees, but also to ensure that the millions of patients who rely on Lilly medicines every day continue to have access to them. | | | | | | | | | | | | | | | |  |
|  |  | | | | | | | | | | | | | | | |  |
|  | | | | | | | | | | | | 2 |  | LF |  | 15/02/2021 15:34 |  |
|  | It's important to point out that not all studies have been paused and any kind of pause is considered temporary by us. Our goal is to get as many studies enrolling again as soon as possible for patients that are already enrolled in trials. We recognize the importance of keeping them engaged. Discontinuation would disrupt their treatment and also potentially diminish the value of the research information that could help patients in the future, and we don't want to do that. Therefore, in line with [U.S. Food and Drug Administration] guidance and regulatory guidance from other countries, we're actively assessing trials on a study-by-study basis and [putting] mitigation plans in place to maintain integrity of these studies while protecting patients and investigators, and I can share with you some specific examples of what Lilly's doing in this area.  We really care about the health and safety of clinical trial participants, their healthcare providers, and support staff, but we also want to make their life as easy as possible. Some of the flexibility that we've put into our protocols include leveraging virtual visits when and where possible, using telemedicine, for example, permitting required lab work to be done locally instead of centrally, minimizing travel and other inconveniences, and providing alternate methods of getting medicines to patients — for example, by delivering directly to their homes. As you said, we know this is a top question and it's something that we're thinking about every single day. | | | | | | | | | | | | | | | |  |
|  |  |  |  |  |  |  |  |  |  |  |  |  |  |  |  |  |  |
|  |  | | | | | | | | | | | | | | | |  |
|  | | | | | | | | | | | | | | | | | |
| Reports\\Coding Summary By Code Report | | | | | | | | | | Page 59 of 123 | | | | | | | |
| 14/07/2021 10:54 | | | | | | | | | | | | | | | | | |
|  | | | **Aggregate** |  | **Classification** |  | **Coverage** |  | **Number Of Coding References** | |  | **Reference Number** |  | **Coded By Initials** |  | **Modified On** |  |
|  | | | **Files\\12 [11 Jan 21]** | | | | | | | | | | | | | |  |
|  |  |  | No |  | All articles |  | 0.0190 |  | 1 | |  | | | | | |  |
|  | | |  |  |  |  |  |  |  | |  | | | | | | |
|  | | | | | | | | | | | | 1 |  | LF |  | 15/02/2021 15:49 |  |
|  | Prior to the pandemic, clinical trials required numerous in-person visits, not only for receiving treatment, but also for completing paperwork and undergoing routine imaging and blood draws, Ribas explained. However, as medical facilities began to scale back operations last spring, clinical trials were quickly adapted to allow participants to continue their involvement without having to visit the clinic. Patients were able to provide consent remotely, utilize telemedicine for appointments, and receive oral medications and other treatments at home. For procedures that required in-person visits, such as imaging or blood tests, patients were able to visit local facilities rather than having to travel to distant research facilities. “The pandemic is going to have lasting impacts on cancer research. One benefit of the pandemic is that it has forced clinical researchers to determine which procedures are absolutely necessary to do in person and which can be completed remotely,” Ribas noted. Although he acknowledged that there has been reduced clinical trial enrollment during the COVID-19 pandemic, he expects that in the long run, “these changes will broaden the ability of people to participate in clinical trials, especially for patients who do not live near research centers and may otherwise not have the means to travel for multiple visits.” | | | | | | | | | | | | | | | |  |
|  |  |  |  |  |  |  |  |  |  |  |  |  |  |  |  |  |  |
|  |  | | | | | | | | | | | | | | | |  |
|  | | | **Files\\24 [14Sept20]** | | | | | | | | | | | | | |  |
|  |  |  | No |  | All articles |  | 0.0548 |  | 3 | |  | | | | | |  |
|  | | |  |  |  |  |  |  |  | |  | | | | | | |
|  | | | | | | | | | | | | 1 |  | LF |  | 17/02/2021 14:27 |  |
|  | From March to June, many cancer drug trials had to stop enrollment because of the COVID19 pandemic, but emergency amendments enabling virtual evaluations, local monitoring, and at-home treatment enabled some research to continue despite lockdowns and safety measures limiting in-person care. | | | | | | | | | | | | | | | |  |
|  |  | | | | | | | | | | | | | | | |  |
|  | | | | | | | | | | | | 2 |  | LF |  | 17/02/2021 14:31 |  |
|  | The US Food and Drug Administration recognized early on that patients wouldn't be able to travel to clinical trial sites due to lockdown rules, and in March issued guidance allowing protocol adjustments that would allow trials to decentralize. The agency told sponsors to consider conducting drug safety evaluations via phone or use labs and imaging centers closer to patients for response monitoring. For drugs that must be administered in a healthcare setting, the agency was open to nurses or trained non-medical personnel administering them to patients at home.  The Centers for Medicare & Medicaid Services also expanded reimbursement for telehealth services and said states could waive in-state licensure requirements for physicians seeing Medicare patients remotely and across state lines, via telemedicine.  These emergency guidelines allowed cancer researchers to quickly pivot to telemedicine and remote monitoring strategies for some trials. According to the ASCO survey, more than 90 percent of respondents said they reviewed patients' symptoms using remote strategies and nearly 88 percent said they engaged with patients via telemedicine whenever possible. | | | | | | | | | | | | | | | |  |
|  |  |  |  |  |  |  |  |  |  |  |  |  |  |  |  |  |  |
|  |  | | | | | | | | | | | | | | | |  |
|  | | | | | | | | | | | | 3 |  | LF |  | 17/02/2021 14:38 |  |
|  | From March to May, Roychowdhury was using telemedicine to check in with almost all his cancer patients. In September, he is using telemedicine to see between 5 percent and 15 percent of patients because quarantine and safety restrictions are still in place in some cases. Importantly, during the critical months of the pandemic, he was able to keep up with all his patients enrolled in an active study via telemedicine thanks to emergency amendments and cooperation from the drug industry.  Flexibility from regulators and payors also helped. "The pandemic happened and literally overnight [CMS] said it will reimburse telemedicine visits, which clearly proves that the infrastructure was all there," said Camidge. "There had to be some motivation to actually do it." | | | | | | | | | | | | | | | |  |
|  |  | | | | | | | | | | | | | | | |  |
|  | | | **Files\\30 (2) [16July20]** | | | | | | | | | | | | | |  |
|  |  |  | No |  | All articles |  | 0.0353 |  | 1 | |  | | | | | |  |
|  | | |  |  |  |  |  |  |  | |  | | | | | | |
|  | | | | | | | | | | | | 1 |  | LF |  | 19/02/2021 15:29 |  |
|  | “If a clinical trial is not deemed critical to survival, those trials are on pause. But unlike some other areas of medicine, cancer clinical trials may be keeping our patients alive,” Lieu says. However, cancer patients are almost universally immunocompromised, whether from the disease itself or from treatments like chemotherapy, radiation and surgery. For these patients, catching COVID-19 could very well be fatal and visiting a hospital with an influx of COVID-19 patients is imperfect, to say the least. Still, cancer doesn’t care about a pandemic and patients still need treatment. “In some cases, the benefits outweigh the risks,” Lieu says. “The idea is striking a balance between not harming someone by withholding care, but also not needlessly increasing exposure.” Some trials even remained open for new enrollment. “To put a new patient on trial, it has to be a very critical situation right now, but those situations exist in cancer,” Lieu says. | | | | | | | | | | | | | | | |  |
|  |  |  |  |  |  |  |  |  |  |  |  |  |  |  |  |  |  |
|  |  | | | | | | | | | | | | | | | |  |
| Reports\\Coding Summary By Code Report | | | | | | | | | | Page 60 of 123 | | | | | | | |
| 14/07/2021 10:54 | | | | | | | | | | | | | | | | | |
|  | | | **Aggregate** |  | **Classification** |  | **Coverage** |  | **Number Of Coding References** | |  | **Reference Number** |  | **Coded By Initials** |  | **Modified On** |  |
|  | | | **Files\\38 (2) [1Dec20]** | | | | | | | | | | | | | |  |
|  |  |  | No |  | All articles |  | 0.1204 |  | 2 | |  | | | | | |  |
|  | | |  |  |  |  |  |  |  | |  | | | | | | |
|  | | | | | | | | | | | | 1 |  | LF |  | 09/03/2021 10:57 |  |
|  | Despite the risks of COVID-19, there remains a general consensus among multidisciplinary teams that life-saving and value-proven interventions should still be offered to patients with cancer. Clinical trials are essential, but may need to be adapted and adjusted to help mitigate risk. Trial protocol writers and investigators may need to rethink and revise some practices, such as converting to digital signatures vs paper/ink and implementing telehealth visits when feasible. Although physical examinations remain a crucial assessment tool, in-person visits may increase the potential exposure risk both for patients and providers. Investigators should reevaluate which in-person physical examinations are necessary and consider whether some could be conducted as telehealth visits (https://www.aptitudehealth.com/blog/cancer-patient-caretelemedicine/) without compromising vigilance. Investigators might also consider whether having tests performed in local certi~ed labs would suf~ce, rather than requiring patients to go to certain trial locations. | | | | | | | | | | | | | | | |  |
|  |  |  |  |  |  |  |  |  |  |  |  |  |  |  |  |  |  |
|  |  | | | | | | | | | | | | | | | |  |
|  | | | | | | | | | | | | 2 |  | LF |  | 09/03/2021 10:58 |  |
|  | As the future course of the pandemic remains uncertain, resources must be prudently allocated so that current trials can remain on track. Many research laboratories were forced to close during the period of widespread COVID-19, which resulted in some clinical trials being paused or even terminated. Adapting trial designs and increasing use of technologies may be necessary in order to save time and resources. For new trials, enrollment might be increased by expanding eligibility criteria and eliminating those of unproven value. Remote practices such as telehealth visits and virtual tumor boards, as well as permitting administration of treatment in local satellite centers, may also help to broaden the scope of upcoming clinical trials. By embracing new technologies to supplement old practices, oncology researchers will be able to continue on the course toward better patient outcomes. | | | | | | | | | | | | | | | |  |
|  |  |  |  |  |  |  |  |  |  |  |  |  |  |  |  |  |  |
|  |  | | | | | | | | | | | | | | | |  |
|  | | | **Files\\46 (2) [25Sept20]** | | | | | | | | | | | | | |  |
|  |  |  | No |  | All articles |  | 0.0901 |  | 1 | |  | | | | | |  |
|  | | |  |  |  |  |  |  |  | |  | | | | | | |
|  | | | | | | | | | | | | 1 |  | LF |  | 09/03/2021 11:00 |  |
|  | Traveling to reach a clinical trial is hard enough for families facing a cancer diagnosis. Add a pandemic to the mix and travel becomes downright daunting. The pandemic lowered new clinical trial enrollments. Patients enrolled in current trials continued treatment—sometimes at the clinic site and other times locally, with their trial doctor coordinating with the local medical team. “We didn’t stop clinical trials. They are the cornerstone of what we do,” said Dr. Dubois.  However, with patients receiving treatment at secondary locations and hospital visits limited by pandemic hospital protocols some of the information gathering could have been compromised. “One goal of a trial is to learn as much as possible from every child that’s on trial, meaning getting extra blood samples, tumor material, surveys, questionnaires to learn as much as possible,” said Dr. Dubois. “Those additional surveys and blood draws had to be shut down.” | | | | | | | | | | | | | | | |  |
|  |  |  |  |  |  |  |  |  |  |  |  |  |  |  |  |  |  |
|  |  | | | | | | | | | | | | | | | |  |
|  | | | **Files\\5 [29Jun20]** | | | | | | | | | | | | | |  |
|  |  |  | No |  | All articles |  | 0.0504 |  | 3 | |  | | | | | |  |
|  | | |  |  |  |  |  |  |  | |  | | | | | | |
|  | | | | | | | | | | | | 1 |  | LF |  | 08/03/2021 10:32 |  |
|  | When the coronavirus pandemic reached the United States, thousands of people with cancer were participating in clinical trials, including many at St. Jude Children’s Research Hospital in Memphis, Tennessee.  At St. Jude, investigators took steps to reduce the risk that children participating in clinical trials, including studies testing new cancer drugs, would become infected with the virus that causes COVID-19. Limiting patient visits to the hospital was one strategy for preventing infections.  So, instead of having patients come to the hospital for routine assessments, nurses went to the patients, who were staying with their families in housing provided by St. Jude.  Teams of nurses, carrying backpacks with medical supplies and personal protective equipment, met with the children and provided basic care, such as collecting blood samples. The nurses essentially brought the clinical trials to the patients, an idea that has attracted new interest among researchers during the pandemic. | | | | | | | | | | | | | | | |  |
|  |  |  |  |  |  |  |  |  |  |  |  |  |  |  |  |  |  |
|  |  | | | | | | | | | | | | | | | |  |
|  | | | | | | | | | | | | 2 |  | LF |  | 08/03/2021 10:35 |  |
|  | Other changes to trials during the pandemic that could be incorporated into future studies include the use of electronic signatures for patient consent forms, remote monitoring of clinical trial results, and shipping oral medications directly to patients participating in clinical trials.  “We’ve learned some lessons,” said William Dahut, M.D., who is scientific director for clinical research at NCI’s Center for Cancer Research and conducts clinical trials. “My hope is that we emerge from this period with improvements in the way we conduct cancer clinical trials.” | | | | | | | | | | | | | | | |  |
|  |  | | | | | | | | | | | | | | | |  |
| Reports\\Coding Summary By Code Report | | | | | | | | | | Page 61 of 123 | | | | | | | |
| 14/07/2021 10:54 | | | | | | | | | | | | | | | | | |
|  | | | **Aggregate** |  | **Classification** |  | **Coverage** |  | **Number Of Coding References** | |  | **Reference Number** |  | **Coded By Initials** |  | **Modified On** |  |
|  | | | | | | | | | | | | | | | | | |
|  | | | | | | | | | | | | 3 |  | LF |  | 08/03/2021 10:37 |  |
|  | At the same time, Dr. Dahut and his colleagues have been trying to reduce the number of required tests and patient visits to the Clinical Center without compromising patient safety and the scientific integrity of the clinical trials.  To do this, the researchers carefully evaluate each patient and consult with the Institutional Review Board and sponsor of the trial, according to a commentary cowritten by Dr. Dahut on what clinical trials might look like after COVID-19. | | | | | | | | | | | | | | | |  |
|  |  | | | | | | | | | | | | | | | |  |
|  | | | **Files\\65 [5Aug20]** | | | | | | | | | | | | | |  |
|  |  |  | No |  | All articles |  | 0.2295 |  | 1 | |  | | | | | |  |
|  | | |  |  |  |  |  |  |  | |  | | | | | | |
|  | | | | | | | | | | | | 1 |  | LF |  | 08/03/2021 16:22 |  |
|  | The authors wrote in the background that cancer is associated with a higher probability of severe forms of COVID-19. Phase I clinical trials trials require an intensive monitoring of patients with multiple in-patient and out-patient unit visits which may represent an additional risk factor for SARS-CoV-2 infection. The occupancy of intensive care units (ICUs) by COVID-19 patients is a signi~cant additional factor to consider when enrolling cancer patients in phase I clinical trials due to frequent life-threatening complications, such as cytokine release syndrome (CRS) in trials evaluating CAR-T cells or bispeci~c antibodies or in those undergoing complicating tumour biopsies.  Currently, there are no recommendations for the management of COVID-19-positive cancer patients included in phase I clinical trials. The authors wrote that their re~ections are based on their experience only. The phase I drug safety pro~le should be considered in the decision of maintaining a COVID-19 positive patient in the trial. The investigational drug should be temporarily or permanently halted in case of any doubt of increased safety risk.  The authors suggested that any candidate for phase I clinical trial should be screened for SARS-CoV-2 by using PCR prior starting the experimental treatment. The patients with a positive PCR should be excluded from the clinical trial, even if asymptomatic at the time of diagnosis. Furthermore, they recommended screening weekly for SARS-CoV-2 by PCR during the dose limiting toxicity (DLT) period and replacing patients who develop a clinical or biological (e.g. asymptomatic but PCR positive) SARS-CoV-2 infection during the DLT period. Beyond the screening phase and the DLT period, they suggested to perform a regular SARS-CoV-2 PCR at the end of the DLT period and subsequently every four cycles and/or upon clinical symptoms. Patients who develop COVID-19 after the DLT period or during the dose-expansion phase may stay in the trial, as long as the treatments received for the management of the COVID-19 do not interfere with the phase I drug metabolism or e~cacy.  In terms of clinical research priorities, the authors wrote that from a regulatory point of view and by law, a patient cannot be included in two different therapeutic interventional trials. However, inclusion in a study evaluating treatments against COVID-19 should probably be favoured at current time.  The authors wrote that in case of an asymptomatic or non-severe COVID-19 con~rmed in a patient included in the phase I trial outside the DLT period, the patient could stay in the trial in most cases. However, adjustments should be done whenever feasible until the patient has recovered clinically and biologically, after discussion and approval of the trial sponsor, including minor deviations in order to limit hospital visits, teleconsulting at increased frequency with the phase I investigator, combined with regular home visits from the patient’s local general practitioner and blood tests at a local facility, treatment shipping to the patient’s domicile, skipping of some surveillance clinics at the hospital when the patient has been stable and on trial for more than 6 months and does not present any evolving drug-related adverse event, stronger personal protection for the patient, active and regular reminder to strictly avoid any self-medication and over the    counter drugs. In order to limit the risk of COVID-19 in cancer patients included in phase I li i  l t i l th ti t d it h h ld h ld l b l l i d d f t i tl  In a perspective article published on 27 July 2020 in the European Journal of Cancer, Dr Sophie Postel-Vinay and colleagues of the Drug Development Department (DITEP), Gustave Roussy in Villejuif, France discuss the key factors to be considered in conducting the phase I clinical trials during the COVID-19 pandemic, as well as in the management of COVID-19-positive cancer patients who are enrolled in those trials. In particular, the authors present the risks and challenges in COVID-19-positive cancer patients enrolled in the phase I clinical trials from the aspects of safety, toxicity causality assessment, drug e~cacy evaluation and clinical research priorities. They proposed the guidelines for conducting phase I clinical trials and management of COVID-19 positive | | | | | | | | | | | | | | | |  |
|  |  |  |  |  |  |  |  |  |  |  |  |  |  |  |  |  |  |
|  |  |  |  |  |  |  |  |  |  |  |  |  |  |  |  |  |  |
|  |  |  |  |  |  |  |  |  |  |  |  |  |  |  |  |  |  |
|  |  |  |  |  |  |  |  |  |  |  |  |  |  |  |  |  |  |
|  |  |  |  |  |  |  |  |  |  |  |  |  |  |  |  |  |  |
|  |  | | | | | | | | | | | | | | | |  |
|  | | | | | | | | | | | | | | | | | |
|  | | | | | | | | | | | | | | | | | |
| Reports\\Coding Summary By Code Report | | | | | | | | | | Page 62 of 123 | | | | | | | |
| 14/07/2021 10:54 | | | | | | | | | | | | | | | | | |
|  | | | **Aggregate** |  | **Classification** |  | **Coverage** |  | **Number Of Coding References** | |  | **Reference Number** |  | **Coded By Initials** |  | **Modified On** |  |
|  | | | **Files\\74 [14Sept20]** | | | | | | | | | | | | | |  |
|  |  |  | No |  | All articles |  | 0.0270 |  | 1 | |  | | | | | |  |
|  | | |  |  |  |  |  |  |  | |  | | | | | | |
|  | | | | | | | | | | | | 1 |  | LF |  | 08/03/2021 17:29 |  |
|  | So clinical trial operations are slowly opening up again, but what strategies are the pharma industry leaning on to make this happen? Pharmafocus caught up with Mari Scheiffele, General Manager UK & Ireland at Novartis Oncology, to find out  how the company has weathered the storm and pushed through the disruption. “With the support of the UK’s Medicines and Healthcare products Regulatory Agency (MHRA), we’ve developed innovative ways to continue many of our existing clinical trials, including creating secure online systems that allow our Clinical Research Associates to work remotely,” she explained. “We have also partnered closely with NHS trusts and healthcare professionals to establish processes for activities like remote follow-ups, which ensure the most at-risk  patients can still safely participate in clinical trials. | | | | | | | | | | | | | | | |  |
|  |  |  |  |  |  |  |  |  |  |  |  |  |  |  |  |  |  |
|  |  | | | | | | | | | | | | | | | |  |
|  | **Nodes\\Direct impacts\Forced adaptation of research protocols\Adaptation of regulatory requirements\Adaptation of clinical trials\Reduced visits** | | | | | | | | | | | | | | | |  |
|  | | **PDF** | | | | | | | | | | | | | | |  |
|  | | | **Files\\5 [29Jun20]** | | | | | | | | | | | | | |  |
|  |  |  | Yes |  | All articles |  | 0.0116 |  | 1 | |  | | | | | |  |
|  | | |  |  |  |  |  |  |  | |  | | | | | | |
|  | | | | | | | | | | | | 1 |  | LF |  | 08/03/2021 10:37 |  |
|  | At the same time, Dr. Dahut and his colleagues have been trying to reduce the number of required tests and patient visits to the Clinical Center without compromising patient safety and the scientific integrity of the clinical trials.  To do this, the researchers carefully evaluate each patient and consult with the Institutional Review Board and sponsor of the trial, according to a commentary cowritten by Dr. Dahut on what clinical trials might look like after COVID-19. | | | | | | | | | | | | | | | |  |
|  |  | | | | | | | | | | | | | | | |  |
|  | | | **Files\\53a [nodate]** | | | | | | | | | | | | | |  |
|  |  |  | Yes |  | All articles |  | 0.0308 |  | 1 | |  | | | | | |  |
|  | | |  |  |  |  |  |  |  | |  | | | | | | |
|  | | | | | | | | | | | | 1 |  | LF |  | 08/03/2021 14:38 |  |
|  | “I think everybody will have a slightly different take on it,” he says. “COVID has certainly brought to the fore discussions on how you operationalise trials. I don’t think it is at this point having any impact on trial design or endpoint selection, but there’s very healthy discussion going on about running trials differently, right from the patient experience through to how we manage data.  “Thinking about patient centricity, for example, some patients may be fearful to go into hospitals as a result of the COVID-19 situation, and those that do are finding the number of appointments available are reduced to ensure social distancing. You can see that there’s a benefit to the patient if they have to go into the clinic less – perhaps once every two months instead of once every four weeks. We just need to make sure we’re not compromising safety.  “All that is on the table and is being feverishly discussed. The actual application of it might be a bit slower.” | | | | | | | | | | | | | | | |  |
|  |  |  |  |  |  |  |  |  |  |  |  |  |  |  |  |  |  |
|  |  | | | | | | | | | | | | | | | |  |
|  | | | | | | | | | | | | | | | | | |
|  | | | | | | | | | | | | | | | | | |
| Reports\\Coding Summary By Code Report | | | | | | | | | | Page 63 of 123 | | | | | | | |
| 14/07/2021 10:54 | | | | | | | | | | | | | | | | | |
|  | | | **Aggregate** |  | **Classification** |  | **Coverage** |  | **Number Of Coding References** | |  | **Reference Number** |  | **Coded By Initials** |  | **Modified On** |  |
|  | **Nodes\\Direct impacts\Forced adaptation of research protocols\Adaptation of regulatory requirements\Adaptation of clinical trials\Remote visits** | | | | | | | | | | | | | | | |  |
|  | | **PDF** | | | | | | | | | | | | | | |  |
|  | | | **Files\\5 [29Jun20]** | | | | | | | | | | | | | |  |
|  |  |  | No |  | All articles |  | 0.0399 |  | 2 | |  | | | | | |  |
|  | | |  |  |  |  |  |  |  | |  | | | | | | |
|  | | | | | | | | | | | | 1 |  | LF |  | 08/03/2021 10:33 |  |
|  | When the coronavirus pandemic reached the United States, thousands of people with cancer were participating in clinical trials, including many at St. Jude Children’s Research Hospital in Memphis, Tennessee.  At St. Jude, investigators took steps to reduce the risk that children participating in clinical trials, including studies testing new cancer drugs, would become infected with the virus that causes COVID-19. Limiting patient visits to the hospital was one strategy for preventing infections.  So, instead of having patients come to the hospital for routine assessments, nurses went to the patients, who were staying with their families in housing provided by St. Jude.  Teams of nurses, carrying backpacks with medical supplies and personal protective equipment, met with the children and provided basic care, such as collecting blood samples. The nurses essentially brought the clinical trials to the patients, an idea that has attracted new interest among researchers during the pandemic. | | | | | | | | | | | | | | | |  |
|  |  |  |  |  |  |  |  |  |  |  |  |  |  |  |  |  |  |
|  |  | | | | | | | | | | | | | | | |  |
|  | | | | | | | | | | | | 2 |  | LF |  | 08/03/2021 11:14 |  |
|  | With a smaller staff at the hospital, some of the standard assessments for children on clinical trials of new treatments had to be delayed. “Some families understandably began to wonder whether their children would be able to receive their treatments on schedule and then learn whether the treatments were effective,” said Dr. Fox. “This was another source of stress.”  Researchers at St. Jude have also begun to ship oral drugs directly to the families of patients testing these medicines, sparing them a trip to the hospital. “This is something we’d like to continue in future trials of oral drugs,” said Dr. Fox. | | | | | | | | | | | | | | | |  |
|  |  | | | | | | | | | | | | | | | |  |
|  | **Nodes\\Direct impacts\Forced adaptation of research protocols\Adaptation of regulatory requirements\Adaptation of clinical trials\Risk assessment** | | | | | | | | | | | | | | | |  |
|  | | **PDF** | | | | | | | | | | | | | | |  |
|  | | | **Files\\10 [21 Oct 20]** | | | | | | | | | | | | | |  |
|  |  |  | No |  | All articles |  | 0.0170 |  | 1 | |  | | | | | |  |
|  | | |  |  |  |  |  |  |  | |  | | | | | | |
|  | | | | | | | | | | | | 1 |  | LF |  | 15/02/2021 12:17 |  |
|  | Above all, the risk of exposing vulnerable cancer patients to the coronavirus during visits to health facilities had to be considered, he added. That calculation allowed for his trials for bowel cancer treatment to continue.  “We needed to assess … where the balance of safety versus risk was in favor of the treatment,” he said. “It's very clear that not being able to cure this cancer can actually have very, very severe consequences for patients.” | | | | | | | | | | | | | | | |  |
|  |  | | | | | | | | | | | | | | | |  |
|  | | | **Files\\22 [30Jun20]** | | | | | | | | | | | | | |  |
|  |  |  | No |  | All articles |  | 0.0545 |  | 1 | |  | | | | | |  |
|  | | |  |  |  |  |  |  |  | |  | | | | | | |
|  | | | | | | | | | | | | 1 |  | LF |  | 17/02/2021 14:13 |  |
|  | “Studies could get a waiver from a designated hospital committee to restart accrual if expected benefits for patients are  large and impact on hospital resources is limited. As the number of infected patients in our centre remained low and the hospital had taken actions to screen patients entering the building, we were able to re-open all studies,” | | | | | | | | | | | | | | | |  |
|  |  | | | | | | | | | | | | | | | |  |
| Reports\\Coding Summary By Code Report | | | | | | | | | | Page 64 of 123 | | | | | | | |
| 14/07/2021 10:54 | | | | | | | | | | | | | | | | | |
|  | | | **Aggregate** |  | **Classification** |  | **Coverage** |  | **Number Of Coding References** | |  | **Reference Number** |  | **Coded By Initials** |  | **Modified On** |  |
|  | | | **Files\\30 (2) [16July20]** | | | | | | | | | | | | | |  |
|  |  |  | No |  | All articles |  | 0.0415 |  | 1 | |  | | | | | |  |
|  | | |  |  |  |  |  |  |  | |  | | | | | | |
|  | | | | | | | | | | | | 1 |  | LF |  | 19/02/2021 15:29 |  |
|  | “If a clinical trial is not deemed critical to survival, those trials are on pause. But unlike some other areas of medicine, cancer clinical trials may be keeping our patients alive,” Lieu says. However, cancer patients are almost universally immunocompromised, whether from the disease itself or from treatments like chemotherapy, radiation and surgery. For these patients, catching COVID-19 could very well be fatal and visiting a hospital with an influx of COVID-19 patients is imperfect, to say the least. Still, cancer doesn’t care about a pandemic and patients still need treatment. “In some cases, the benefits outweigh the risks,” Lieu says. “The idea is striking a balance between not harming someone by withholding care, but also not needlessly increasing exposure.” Some trials even remained open for new enrollment. “To put a new patient on trial, it has to be a very critical situation right now, but those situations exist in cancer,” Lieu says.  “Leadership on our campus is doing a great job weighing the various risks and benefits of how we provide cancer care, and working together to find the right balance,” | | | | | | | | | | | | | | | |  |
|  |  |  |  |  |  |  |  |  |  |  |  |  |  |  |  |  |  |
|  |  | | | | | | | | | | | | | | | |  |
|  | **Nodes\\Direct impacts\Forced adaptation of research protocols\Adaptation of regulatory requirements\Adaptation of clinical trials\Use of communications technology** | | | | | | | | | | | | | | | |  |
|  | | **PDF** | | | | | | | | | | | | | | |  |
|  | | | **Files\\1 (2) [8th Oct 20]** | | | | | | | | | | | | | |  |
|  |  |  | No |  | All articles |  | 0.0204 |  | 1 | |  | | | | | |  |
|  | | |  |  |  |  |  |  |  | |  | | | | | | |
|  | | | | | | | | | | | | 1 |  | LF |  | 15/02/2021 12:19 |  |
|  | Despite these challenges, investigators found a variety of ways to adapt to straitened circumstances so trials could continue. These included leveraging telehealth to limit in-person visits, use of e-signatures for trial documentation, shipping oral medications to trial participants rather than requiring them to be picked up at the clinic, and allowing laboratory tests to be done at outside labs. | | | | | | | | | | | | | | | |  |
|  |  | | | | | | | | | | | | | | | |  |
|  | | | **Files\\10 [21 Oct 20]** | | | | | | | | | | | | | |  |
|  |  |  | No |  | All articles |  | 0.0137 |  | 1 | |  | | | | | |  |
|  | | |  |  |  |  |  |  |  | |  | | | | | | |
|  | | | | | | | | | | | | 1 |  | LF |  | 15/02/2021 12:19 |  |
|  | Similarly, Cameron said, one of his breast cancer trials was able to continue despite the pandemic. It was just a matter of adapting practices to the restrictions of the virus.  “Let's be creative, without changing the quality of what we do,” he said, pointing to the example of the adoption of telemedicine for consultations that were previously done in person. | | | | | | | | | | | | | | | |  |
|  |  | | | | | | | | | | | | | | | |  |
|  | | | **Files\\118 [1 May 20]** | | | | | | | | | | | | | |  |
|  |  |  | No |  | All articles |  | 0.0239 |  | 1 | |  | | | | | |  |
|  | | |  |  |  |  |  |  |  | |  | | | | | | |
|  | | | | | | | | | | | | 1 |  | LF |  | 15/02/2021 15:34 |  |
|  | Therefore, in line with [U.S. Food and Drug Administration] guidance and regulatory guidance from other countries, we're actively assessing trials on a study-by-study basis and [putting] mitigation plans in place to maintain integrity of these studies while protecting patients and investigators, and I can share with you some specific examples of what Lilly's doing in this area.  We really care about the health and safety of clinical trial participants, their healthcare providers, and support staff, but we also want to make their life as easy as possible. Some of the flexibility that we've put into our protocols include leveraging virtual visits when and where possible | | | | | | | | | | | | | | | |  |
|  |  | | | | | | | | | | | | | | | |  |
| Reports\\Coding Summary By Code Report | | | | | | | | | | Page 65 of 123 | | | | | | | |
| 14/07/2021 10:54 | | | | | | | | | | | | | | | | | |
|  | | | **Aggregate** |  | **Classification** |  | **Coverage** |  | **Number Of Coding References** | |  | **Reference Number** |  | **Coded By Initials** |  | **Modified On** |  |
|  | | | **Files\\24 [14Sept20]** | | | | | | | | | | | | | |  |
|  |  |  | No |  | All articles |  | 0.0548 |  | 3 | |  | | | | | |  |
|  | | |  |  |  |  |  |  |  | |  | | | | | | |
|  | | | | | | | | | | | | 1 |  | LF |  | 17/02/2021 14:27 |  |
|  | From March to June, many cancer drug trials had to stop enrollment because of the COVID19 pandemic, but emergency amendments enabling virtual evaluations, local monitoring, and at-home treatment enabled some research to continue despite lockdowns and safety measures limiting in-person care. | | | | | | | | | | | | | | | |  |
|  |  | | | | | | | | | | | | | | | |  |
|  | | | | | | | | | | | | 2 |  | LF |  | 17/02/2021 14:31 |  |
|  | The US Food and Drug Administration recognized early on that patients wouldn't be able to travel to clinical trial sites due to lockdown rules, and in March issued guidance allowing protocol adjustments that would allow trials to decentralize. The agency told sponsors to consider conducting drug safety evaluations via phone or use labs and imaging centers closer to patients for response monitoring. For drugs that must be administered in a healthcare setting, the agency was open to nurses or trained non-medical personnel administering them to patients at home.  The Centers for Medicare & Medicaid Services also expanded reimbursement for telehealth services and said states could waive in-state licensure requirements for physicians seeing Medicare patients remotely and across state lines, via telemedicine.  These emergency guidelines allowed cancer researchers to quickly pivot to telemedicine and remote monitoring strategies for some trials. According to the ASCO survey, more than 90 percent of respondents said they reviewed patients' symptoms using remote strategies and nearly 88 percent said they engaged with patients via telemedicine whenever possible. | | | | | | | | | | | | | | | |  |
|  |  |  |  |  |  |  |  |  |  |  |  |  |  |  |  |  |  |
|  |  | | | | | | | | | | | | | | | |  |
|  | | | | | | | | | | | | 3 |  | LF |  | 17/02/2021 14:38 |  |
|  | From March to May, Roychowdhury was using telemedicine to check in with almost all his cancer patients. In September, he is using telemedicine to see between 5 percent and 15 percent of patients because quarantine and safety restrictions are still in place in some cases. Importantly, during the critical months of the pandemic, he was able to keep up with all his patients enrolled in an active study via telemedicine thanks to emergency amendments and cooperation from the drug industry.  Flexibility from regulators and payors also helped. "The pandemic happened and literally overnight [CMS] said it will reimburse telemedicine visits, which clearly proves that the infrastructure was all there," said Camidge. "There had to be some motivation to actually do it." | | | | | | | | | | | | | | | |  |
|  |  | | | | | | | | | | | | | | | |  |
|  | | | **Files\\5 [29Jun20]** | | | | | | | | | | | | | |  |
|  |  |  | No |  | All articles |  | 0.0193 |  | 1 | |  | | | | | |  |
|  | | |  |  |  |  |  |  |  | |  | | | | | | |
|  | | | | | | | | | | | | 1 |  | LF |  | 08/03/2021 10:34 |  |
|  | Another change made by St. Jude was to begin virtual research visits between health care providers and patients, also known as telehealth or telemedicine. Some of the nurses who visited patients staying at St. Jude had been trained to help with telehealth visits.  For telehealth and virtual research visits to continue beyond the pandemic, said Mutz, some of the temporary changes that had been made to regulations on the use of telehealth during COVID-19 would need to become permanent.  A greater use of telemedicine has emerged as one of the positive changes to cancer clinical trials that could be continued after the pandemic has passed,  according to the results of two surveys of clinical trial investigators and a series of recent commentaries by leaders of clinical trials. | | | | | | | | | | | | | | | |  |
|  |  |  |  |  |  |  |  |  |  |  |  |  |  |  |  |  |  |
|  |  | | | | | | | | | | | | | | | |  |
|  | | | **Files\\50 (2) [no date]** | | | | | | | | | | | | | |  |
|  |  |  | No |  | All articles |  | 0.0278 |  | 1 | |  | | | | | |  |
|  | | |  |  |  |  |  |  |  | |  | | | | | | |
|  | | | | | | | | | | | | 1 |  | LF |  | 09/03/2021 11:08 |  |
|  | Silver Linings and Next Steps One thing that stands out to me is that some positive developments have emerged from this otherwise terrible situation.  Among them, we have seen that telehealth is having its moment. As I said at the meeting, researchers involved in implementation science (https://www.cancer.gov/research/implementation-science-infographic) will have a unique opportunity to analyze the impact that telehealth has had on our ability to manage patient care during this pandemic and how telehealth can be most effectively used, and expanded, going forward.  I predict that those being treated for cancer are going to like having some of their care delivered by telehealth, and that many will want to continue using telehealth for some aspects of their care long after the pandemic has ended. | | | | | | | | | | | | | | | |  |
|  |  |  |  |  |  |  |  |  |  |  |  |  |  |  |  |  |  |
|  |  | | | | | | | | | | | | | | | |  |
| Reports\\Coding Summary By Code Report | | | | | | | | | | Page 66 of 123 | | | | | | | |
| 14/07/2021 10:54 | | | | | | | | | | | | | | | | | |
|  | | | **Aggregate** |  | **Classification** |  | **Coverage** |  | **Number Of Coding References** | |  | **Reference Number** |  | **Coded By Initials** |  | **Modified On** |  |
|  | | | **Files\\53a [nodate]** | | | | | | | | | | | | | |  |
|  |  |  | No |  | All articles |  | 0.0241 |  | 1 | |  | | | | | |  |
|  | | |  |  |  |  |  |  |  | |  | | | | | | |
|  | | | | | | | | | | | | 1 |  | LF |  | 08/03/2021 14:36 |  |
|  | Many would argue that the biggest catalyst for change in cancer research has been the COVID-19 pandemic, which has forced a shift towards remote monitoring, virtual arms and other uses of digital tools as global lockdowns reduce the ability of patients to go to clinics.  Lachs, however, says it’s still too early to tell whether these changes will be permanent.  “I think everybody will have a slightly different take on it,” he says. “COVID has certainly brought to the fore discussions on how you operationalise trials. I don’t think it is at this point having any impact on trial design or endpoint selection, but there’s very healthy discussion going on about running trials differently, right from the patient experience through to how we manage data. | | | | | | | | | | | | | | | |  |
|  |  | | | | | | | | | | | | | | | |  |
|  | | | **Files\\62 [27Nov20]** | | | | | | | | | | | | | |  |
|  |  |  | No |  | All articles |  | 0.1587 |  | 1 | |  | | | | | |  |
|  | | |  |  |  |  |  |  |  | |  | | | | | | |
|  | | | | | | | | | | | | 1 |  | LF |  | 08/03/2021 15:40 |  |
|  | A survey of Dutch cancer patients in the PROFILES registry highlights the impact of the COVID-19 crisis on the care of individuals with cancer, with approximately a third reporting postponed or cancelled appointments.  “Longitudinal evaluation will reveal whether this has an association with their long-term health outcomes”, say Lonneke van de Poll-Franse, from the Netherlands Comprehensive Cancer Organisation in Utrecht, and colleagues.  As described in JAMA Oncology, the online questionnaire was completed by 4094 cancer patients and 2351 individuals from the general Dutch population in April or May 2020, with age and sex matching to cancer patients achieved for 977 of the controls.  Overall, 10.8% of the 886 patients who were undergoing or due to start cancer therapy had their treatment postponed or cancelled, and 18.1% had a consultation changed to a telephone or video call.  Similarly, 10.8% of 2725 cancer patients undergoing follow-up had an appointment postponed or cancelled and 8.6% had an appointment switched to a telephone or video call.  Both patients and individuals from the general population said they were slower than usual to contact their general practitioners (20.9 vs 22.3%) or medical specialist or nurse (14.4 vs 14.7%) with complaints, questions or concerns during the COVID-19 crisis.  Of 394 patients who had a face-to-face appointment replaced with a telephone or video call, just 58.1% said their appointment was suitable for the remote approach; 74.4% preferred a face-to-face meeting, although 38.3% said they would accept a telephone or video appointment in the future.  “This ~nding may help change our care for patients not only during the potentially longlasting COVID-19 period, but also beyond”, the researchers comment. | | | | | | | | | | | | | | | |  |
|  |  |  |  |  |  |  |  |  |  |  |  |  |  |  |  |  |  |
|  |  |  |  |  |  |  |  |  |  |  |  |  |  |  |  |  |  |
|  |  | | | | | | | | | | | | | | | |  |
|  | **Nodes\\Direct impacts\Forced adaptation of research protocols\Adaptation of regulatory requirements\Concern about potential intransigence of regulators post-pandemic** | | | | | | | | | | | | | | | |  |
|  | | **PDF** | | | | | | | | | | | | | | |  |
|  | | | **Files\\24 [14Sept20]** | | | | | | | | | | | | | |  |
|  |  |  | No |  | All articles |  | 0.0293 |  | 2 | |  | | | | | |  |
|  | | |  |  |  |  |  |  |  | |  | | | | | | |
|  | | | | | | | | | | | | 1 |  | LF |  | 17/02/2021 14:39 |  |
|  | While oncologists, drugmakers, researchers, and patients may be willing to make more use of telemedicine and remote strategies, other decision makers in the community haven't yet committed to such changes long term. "There is concern about the FDA's position on this once the pandemic is in the rearview mirror, and the position of the payors because they have been flexible with reimbursement for telemedicine," said Gateway's Burton. | | | | | | | | | | | | | | | |  |
|  |  | | | | | | | | | | | | | | | |  |
| Reports\\Coding Summary By Code Report | | | | | | | | | | Page 67 of 123 | | | | | | | |
| 14/07/2021 10:54 | | | | | | | | | | | | | | | | | |
|  | | | **Aggregate** |  | **Classification** |  | **Coverage** |  | **Number Of Coding References** | |  | **Reference Number** |  | **Coded By Initials** |  | **Modified On** |  |
|  | | | | | | | | | | | | | | | | | |
|  | | | | | | | | | | | | 2 |  | LF |  | 17/02/2021 14:40 |  |
|  | When the pandemic hit, Camidge, like Roychowdhury and most other doctors concerned about the welfare of their patients, used telemedicine to provide care in and outside of research settings. "I had a duty of care to my patients, so I would phone them and put a note into the electronic medical record," he said. While Camidge did this without giving much thought to whether that interaction was going to be billed and reimbursed, the hospital might feel differently.  "The physicians mostly just want to look after their patients, and if patients are choosing to stay in another state when they're doing that, the physicians probably don't care," he said. "But the health system and the medical legal experts may care. And that's what we have to figure out." | | | | | | | | | | | | | | | |  |
|  |  | | | | | | | | | | | | | | | |  |
|  | **Nodes\\Direct impacts\Forced adaptation of research protocols\Adoption of new technologies** | | | | | | | | | | | | | | | |  |
|  | | **Document** | | | | | | | | | | | | | | |  |
|  | | | **Files\\460 [4Dec20]** | | | | | | | | | | | | | |  |
|  |  |  | No |  | All articles |  | 0.2755 |  | 1 | |  | | | | | |  |
|  | | |  |  |  |  |  |  |  | |  | | | | | | |
|  | | | | | | | | | | | | 1 |  | LF |  | 07/03/2021 12:10 |  |
|  | 'Proscia's commercial traction comes amid a surge in digital pathology adoption. Laboratories have increasingly shifted to digital to overcome the manual and subjective nature of the traditional standard of care and keep pace with the rising cancer burden. In the U.S. alone, pathologists have faced a 42% rise in diagnostic workload over the last decade, a challenge that will continue to intensify as the total number of cancer cases is projected to increase by 55% by 2030. Recently, laboratories have been implementing digital pathology to maintain operations during the COVID-19 pandemic, as digitization is the only means by which they can continue to serve patients.'Digital pathology is quickly becoming theexpectedstandard of diagnosis,' said Steve Holloway, Company Director &#38; Principal Analyst at Signify Research. 'The global pandemic has exacerbated longstanding pressures, accelerating adoption. This market has now crossed an inflection point, with a critical mass of digital laboratories creating the momentum formorewidespread implementation of digital pathologyandaugmentationby artificial intelligence.'Proscia was represented by Troutman Pepperin connection with the Series B financing.Learn MoreAbout Proscia Proscia is a software company that is changing the way the world practices pathology to transform cancer research and diagnosis. With the company's Concentriq digital pathology platform and pipeline of AI-powered applications, laboratories are leveraging new kinds of data to accelerate discoveries and improve patient outcomes. Proscia's team of technologists, scientists, and pathologists is bringing a fresh approach to an outdated industry, helping the world to keep pace with the increasing demand for pathology services and fulfill the promise of precision diagnostics. | | | | | | | | | | | | | | | |  |
|  |  |  |  |  |  |  |  |  |  |  |  |  |  |  |  |  |  |
|  |  |  |  |  |  |  |  |  |  |  |  |  |  |  |  |  |  |
|  |  | | | | | | | | | | | | | | | |  |
|  | | | **Files\\485 [1Dec20]** | | | | | | | | | | | | | |  |
|  |  |  | No |  | All articles |  | 0.1286 |  | 1 | |  | | | | | |  |
|  | | |  |  |  |  |  |  |  | |  | | | | | | |
|  | | | | | | | | | | | | 1 |  | LF |  | 07/03/2021 17:03 |  |
|  | PathologyProscia's commercial traction comes amid a surge in digital pathology adoption. Laboratories have increasingly shifted to digital to overcome the manual and subjective nature of the traditional standard of care and keep pace with the rising cancer burden. In the U.S. alone, pathologists have faced a 42% rise in diagnostic workload over the last decade, a challenge that will continue to intensify as the total number of cancer cases is projected to increase by 55% by 2030. Recently, laboratories have been implementing digital pathology to maintain operations during the COVID-19 pandemic, as digitization is the only means by which they can continue to serve patients. | | | | | | | | | | | | | | | |  |
|  |  | | | | | | | | | | | | | | | |  |
|  | | | | | | | | | | | | | | | | | |
| Reports\\Coding Summary By Code Report | | | | | | | | | | Page 68 of 123 | | | | | | | |
| 14/07/2021 10:54 | | | | | | | | | | | | | | | | | |
|  | | | **Aggregate** |  | **Classification** |  | **Coverage** |  | **Number Of Coding References** | |  | **Reference Number** |  | **Coded By Initials** |  | **Modified On** |  |
|  | | | **Files\\498 [13Jun20]** | | | | | | | | | | | | | |  |
|  |  |  | No |  | All articles |  | 0.3241 |  | 1 | |  | | | | | |  |
|  | | |  |  |  |  |  |  |  | |  | | | | | | |
|  | | | | | | | | | | | | 1 |  | LF |  | 07/03/2021 17:11 |  |
|  | NHS England is accelerating the launch of a new type of radiotherapy that requires fewer doses than current approaches, thus cutting the number of hospital visits for potentially vulnerable cancer patients.Stereotactic ablative radiotherapy (SABR) is a very precise method using a high dose of radiations with only around five outpatient visits, compared to conventional radiotherapy which needs 20 - 30 treatments.Rather than full rollout by 2022, it will now be available across the NHS by the end of this financial year, with every part of the country offering SABR treatment for non-small cell lung cancer and those with lung, lymph nodes and non-spine bone oligometastatic disease, in radiotherapy units nationwide.Further rollout for other disease types is planned for 2021/22.The move to offer more convenient and potentially safer forms of treatment comes as cancer services are struggling to cope with a backlog of patients who are more vulnerable to coronavirus and thus have been unable to get treatment during the pandemic. | | | | | | | | | | | | | | | |  |
|  |  |  |  |  |  |  |  |  |  |  |  |  |  |  |  |  |  |
|  |  | | | | | | | | | | | | | | | |  |
|  | | **PDF** | | | | | | | | | | | | | | |  |
|  | | | **Files\\49 (2) [6Jan20]** | | | | | | | | | | | | | |  |
|  |  |  | No |  | All articles |  | 0.2878 |  | 1 | |  | | | | | |  |
|  | | |  |  |  |  |  |  |  | |  | | | | | | |
|  | | | | | | | | | | | | 1 |  | LF |  | 07/03/2021 17:07 |  |
|  | The pandemic and its socioeconomic ramifications have led to “a substantial, immediateterm decrease in accrual to both diagnostic and therapeutic cancer investigations as well as substantive alterations in patterns of oncologic care,” the leaders said (https://doi.org/10.1093/jnci/djaa162). But it creates an opportunity to make changes to clinical trial designs that benefit society long after the pandemic.  Leaders from NCI’s Cancer Therapy Evaluation Program, Center for Cancer Research, Community Oncology Research Program, Coordinating Center for Clinical Trials, and Division of Cancer Treatment and Diagnosis recommended the following strategies:  Use telemedicine to enhance patient access. Improve data integration into electronic health records. Simplify statistical designs.  Minimize and expedite the review process, particularly for rare cancers. Adjust personnel requirements and use electronic auditing.  Work with the U.S. Food and Drug Administration to simplify the regulatory framework.  Minimize the need for nonessential tests. Use electronic consent forms that can be completed remotely.  “At the crossroads of cancer, COVID-19, and growing social inequities, substantive opportunities to improve the current methodology of oncologic clinical investigation have manifested themselves in dramatic fashion,” the leaders wrote (https://doi.org/10.1093/jnci/djaa162). “Now is the time to make fundamental changes that will lead to sustainable improvements in our approach to and conduct of clinical cancer research, changes that will provide broad, long-lasting benefit to all patients and to society.” | | | | | | | | | | | | | | | |  |
|  |  |  |  |  |  |  |  |  |  |  |  |  |  |  |  |  |  |
|  |  |  |  |  |  |  |  |  |  |  |  |  |  |  |  |  |  |
|  |  | | | | | | | | | | | | | | | |  |
|  | | | | | | | | | | | | | | | | | |
|  | | | | | | | | | | | | | | | | | |
|  | | | | | | | | | | | | | | | | | |
| Reports\\Coding Summary By Code Report | | | | | | | | | | Page 69 of 123 | | | | | | | |
| 14/07/2021 10:54 | | | | | | | | | | | | | | | | | |
|  | | | **Aggregate** |  | **Classification** |  | **Coverage** |  | **Number Of Coding References** | |  | **Reference Number** |  | **Coded By Initials** |  | **Modified On** |  |
|  | **Nodes\\Direct impacts\Forced adaptation of research protocols\Continuity of work\Continuation of cancer research findings** | | | | | | | | | | | | | | | |  |
|  | | **Document** | | | | | | | | | | | | | | |  |
|  | | | **Files\\124 [24 July 20]** | | | | | | | | | | | | | |  |
|  |  |  | No |  | All articles |  | 0.1645 |  | 1 | |  | | | | | |  |
|  | | |  |  |  |  |  |  |  | |  | | | | | | |
|  | | | | | | | | | | | | 1 |  | LF |  | 15/02/2021 16:31 |  |
|  | But there is good news in all this darkness, both when it comes to COVID-19 and other illnesses. The latest noteworthy medical development concerns cancer, as researchers have devised a test that can detect several types of cancer up to four years before symptoms appear. If accurate, such a test could help in diagnosing some of the most common types of cancer and prepare the patient and their physicians for treating the illness. An international team of scientists published a new study in Nature Communications[3], explaining that their assay could diagnose stomach, esophageal, colorectal, lung, and liver cancer with the help of a single blood sample. The results could be available up to four years before the patients start developing symptoms. 'What we showed is: up to four years before these people walk into the hospital, there are already signatures in their blood that show they have cancer,'University of California bioengineer Kun Zhang told Scientific American[4]. 'That's never been done before.' | | | | | | | | | | | | | | | |  |
|  |  |  |  |  |  |  |  |  |  |  |  |  |  |  |  |  |  |
|  |  | | | | | | | | | | | | | | | |  |
|  | | | **Files\\212 [2Jun20]** | | | | | | | | | | | | | |  |
|  |  |  | No |  | All articles |  | 0.1800 |  | 1 | |  | | | | | |  |
|  | | |  |  |  |  |  |  |  | |  | | | | | | |
|  | | | | | | | | | | | | 1 |  | LF |  | 17/02/2021 14:11 |  |
|  | Despite a global pandemic, Sanofi SA (NASDAQ: SNY[1]) is able to continue its research and development — and it is "essential" to continue showing success in trials, CEO Paul Hudson said on CNBC's "Squawk Box."[2]Sanofi's Momentum In OncologySanofi's existing oncology trials are ongoing, and this helped create some new momentum, Hudson said. Most notably, a trial of Sanofi and Regeneron Pharmaceuticals Inc's (NASDAQ: REGN[3]) lung cancer drug Libtayo cut the risk of death by more than 30% among certain patients, he said. | | | | | | | | | | | | | | | |  |
|  |  | | | | | | | | | | | | | | | |  |
|  | **Nodes\\Direct impacts\Forced adaptation of research protocols\Continuity of work\Continuation of clinical trials initiated before the pandemic** | | | | | | | | | | | | | | | |  |
|  | | **Document** | | | | | | | | | | | | | | |  |
|  | | | **Files\\320 [6June20]** | | | | | | | | | | | | | |  |
|  |  |  | No |  | All articles |  | 0.1590 |  | 1 | |  | | | | | |  |
|  | | |  |  |  |  |  |  |  | |  | | | | | | |
|  | | | | | | | | | | | | 1 |  | LF |  | 19/02/2021 16:40 |  |
|  | UpdateOn the earnings call, the company stated that clinical studies in oncology conducted by the company have not experienced any significant delays. However, the company cautioned that uncertainties concerning COVID-19 may delay late-stage studies or enrollment of new patients in clinical studies in the future. Moreover, it expects a delay of approximately three months related to the previously anticipated timelines for certain earlier-stage studies, including the phase I/II PROPEL study evaluating bempegaldesleukin in combination with Merck's Keytruda in patients with select advanced or metastatic solid tumors.Meanwhile, the company expects a delay of three to six months in the anticipated timeline of its partner-sponsored clinical studies. | | | | | | | | | | | | | | | |  |
|  |  | | | | | | | | | | | | | | | |  |
|  | | | | | | | | | | | | | | | | | |
| Reports\\Coding Summary By Code Report | | | | | | | | | | Page 70 of 123 | | | | | | | |
| 14/07/2021 10:54 | | | | | | | | | | | | | | | | | |
|  | | | **Aggregate** |  | **Classification** |  | **Coverage** |  | **Number Of Coding References** | |  | **Reference Number** |  | **Coded By Initials** |  | **Modified On** |  |
|  | | | **Files\\52 [25May20]** | | | | | | | | | | | | | |  |
|  |  |  | No |  | All articles |  | 0.0151 |  | 1 | |  | | | | | |  |
|  | | |  |  |  |  |  |  |  | |  | | | | | | |
|  | | | | | | | | | | | | 1 |  | LF |  | 08/03/2021 14:16 |  |
|  | Pivotal Israeli pancreatic cancer study hits benchmark amid corona[44] Rafael Pharmaceuticals has reached 85% of the 500-patient enrollment needed for the pivotal phase-3 clinical trial of its metastatic pancreatic cancer treatment despite the coronavirus crisis, the company told The Jerusalem Post. In addition, it has launched a COVID-19 task force to ensure that appropriate medical centers hosting the trials are implementing safety measures so that testing could continue.Many of the participating patients are from Israel, according to Sanjeev Luther, Rafael's president and CEO. He explained that the trial is evaluating the efficacy and safety of the company's lead compound CPI-613 in combination with modified FOLFIRINOX as a first-line therapy. | | | | | | | | | | | | | | | |  |
|  |  | | | | | | | | | | | | | | | |  |
|  | | | **Files\\92 [12May20]** | | | | | | | | | | | | | |  |
|  |  |  | No |  | All articles |  | 0.0923 |  | 1 | |  | | | | | |  |
|  | | |  |  |  |  |  |  |  | |  | | | | | | |
|  | | | | | | | | | | | | 1 |  | LF |  | 09/03/2021 10:14 |  |
|  | BriaCell Therapeutics announces that the Phase I/IIa clinical study of Bria-IMT™, for breast cancer treatment, with Incyte Corporation's immune checkpoint inhibitor, INCMGA00012, is ongoing and recruiting patients amidst the COVID-19 pandemic. Phase I/IIa clinical study of BriaCell's lead candidate, Bria-IMT™, for breast cancer treatment, with Incyte Corporation's immune checkpoint inhibitor, INCMGA00012, is ongoing and recruiting patients amidst the COVID-19 pandemic. BriaCell is currently evaluating a number of business strategies to develop its potential treatments for breast cancer on its own or in partnership. | | | | | | | | | | | | | | | |  |
|  |  | | | | | | | | | | | | | | | |  |
|  | | **PDF** | | | | | | | | | | | | | | |  |
|  | | | **Files\\5 [29Jun20]** | | | | | | | | | | | | | |  |
|  |  |  | No |  | All articles |  | 0.0254 |  | 1 | |  | | | | | |  |
|  | | |  |  |  |  |  |  |  | |  | | | | | | |
|  | | | | | | | | | | | | 1 |  | LF |  | 08/03/2021 10:37 |  |
|  | Clinical trials are critical for progress against cancer. And trials that evaluate new therapies are often the best, or the only, treatment option for some patients. During the pandemic, NCI continued to enroll clinical trial participants for whom the NIH Clinical Center, located in Bethesda, MD, had the most appropriate treatments.  The biggest challenge in the first weeks of the pandemic “was a sense of the unknown,” said Dr. Dahut. Travel restrictions and other measures put in place to reduce the spread of the novel coronavirus raised questions about how to care for patients who travel long distances to come to their appointments. Should these patients even make the trip to Bethesda? Should their treatments be given on a different schedule, such as every 6 weeks rather than every 4 weeks?  “We were trying to find the balance between treating patients for their cancers and ensuring that the patients were safe while traveling,” he said. But the researchers had limited information to guide their decisions, he added | | | | | | | | | | | | | | | |  |
|  |  |  |  |  |  |  |  |  |  |  |  |  |  |  |  |  |  |
|  |  | | | | | | | | | | | | | | | |  |
|  | | | | | | | | | | | | | | | | | |
|  | | | | | | | | | | | | | | | | | |
| Reports\\Coding Summary By Code Report | | | | | | | | | | Page 71 of 123 | | | | | | | |
| 14/07/2021 10:54 | | | | | | | | | | | | | | | | | |
|  | | | **Aggregate** |  | **Classification** |  | **Coverage** |  | **Number Of Coding References** | |  | **Reference Number** |  | **Coded By Initials** |  | **Modified On** |  |
|  | | | **Files\\50 (2) [no date]** | | | | | | | | | | | | | |  |
|  |  |  | No |  | All articles |  | 0.0227 |  | 1 | |  | | | | | |  |
|  | | |  |  |  |  |  |  |  | |  | | | | | | |
|  | | | | | | | | | | | | 1 |  | LF |  | 09/03/2021 11:07 |  |
|  | Adapting Cancer Clinical Trials, Launching New Trials  Not surprisingly, the COVID-19 pandemic has had a substantial impact on cancer clinical trials. I have spoken with directors and other staff from NCI-designated cancer centers and this is a serious concern.  As NCI Deputy Director Jim Doroshow, MD, reported at the board meeting, accrual to NCI-funded treatment clinical trials has dropped by half, a trend that is expected to persist. Accrual to certain trials has continued — for example, those offering life-saving therapies or those for patients who have no other options for therapy — but accrual to most other types of trials has sharply declined. | | | | | | | | | | | | | | | |  |
|  |  | | | | | | | | | | | | | | | |  |
|  | **Nodes\\Direct impacts\Forced adaptation of research protocols\Continuity of work\Continuation of private (industry) funding** | | | | | | | | | | | | | | | |  |
|  | | **Document** | | | | | | | | | | | | | | |  |
|  | | | **Files\\291 [5Nov20]** | | | | | | | | | | | | | |  |
|  |  |  | No |  | All articles |  | 0.7862 |  | 1 | |  | | | | | |  |
|  | | |  |  |  |  |  |  |  | |  | | | | | | |
|  | | | | | | | | | | | | 1 |  | LF |  | 17/02/2021 17:25 |  |
|  | The Oncology Development programme is also receiving funding from Innovate UK and Cancer Research UK, as part of Cancer Research UK's Entrepreneurial Programmes Initiative.The aim of the programme is to advance viable oncology projects more quickly to increase their chances of commercial success.Also collaborating on the project is major pharmaceutical companies AstraZeneca, Johnson &#38; Johnson Innovation, GlaxoSmithKline and Roche, as well as The Christie NHS Foundation Trust, Medicines Discovery Catapult and the Manchester Cancer Research Centre.They will support researchers and start-ups looking to develop early-stage innovations within the oncology space.The nine-month programme's ultimate goal is to provide potential investors with detailed plans of how companies are planning to deliver key data and value milestone during the next two to three years.Participating researchers and companies who progress through the programme will be eligible to receive up to 60,000 of grant funding to support further development of their business plans.'This is a really exciting and unique project that will help turn some of the most promising ideas in cancer research today into investable projects that will improve the way that cancer patients are diagnosed and treated during the course of their disease,' said Kath Mackay, managing director of Bruntwood SciTech - Alderley Park.'It's no secret that while the life sciences sector and indeed the wider world has understandably been focused on the pandemic, funding and research into beating cancer has suffered.  Our new programme couldn't be launching at a more critical time and, as with the race to find a Covid-19 vaccine, it signals a major collaborative effort involving global leaders,' she added. | | | | | | | | | | | | | | | |  |
|  |  |  |  |  |  |  |  |  |  |  |  |  |  |  |  |  |  |
|  |  |  |  |  |  |  |  |  |  |  |  |  |  |  |  |  |  |
|  |  | | | | | | | | | | | | | | | |  |
|  | | | | | | | | | | | | | | | | | |
|  | | | | | | | | | | | | | | | | | |
|  | | | | | | | | | | | | | | | | | |
| Reports\\Coding Summary By Code Report | | | | | | | | | | Page 72 of 123 | | | | | | | |
| 14/07/2021 10:54 | | | | | | | | | | | | | | | | | |
|  | | | **Aggregate** |  | **Classification** |  | **Coverage** |  | **Number Of Coding References** | |  | **Reference Number** |  | **Coded By Initials** |  | **Modified On** |  |
|  | | **PDF** | | | | | | | | | | | | | | |  |
|  | | | **Files\\119 (2) [no date]** | | | | | | | | | | | | | |  |
|  |  |  | No |  | All articles |  | 0.0154 |  | 1 | |  | | | | | |  |
|  | | |  |  |  |  |  |  |  | |  | | | | | | |
|  | | | | | | | | | | | | 1 |  | LF |  | 15/02/2021 16:26 |  |
|  | We all feel incredibly grateful for the unwavering support of our industry sponsors who stand by us every step of the way. | | | | | | | | | | | | | | | |  |
|  |  | | | | | | | | | | | | | | | |  |
|  | **Nodes\\Direct impacts\Forced adaptation of research protocols\Continuity of work\Continuation of research meetings** | | | | | | | | | | | | | | | |  |
|  | | **Document** | | | | | | | | | | | | | | |  |
|  | | | **Files\\89 [2June20]** | | | | | | | | | | | | | |  |
|  |  |  | No |  | All articles |  | 0.1437 |  | 1 | |  | | | | | |  |
|  | | |  |  |  |  |  |  |  | |  | | | | | | |
|  | | | | | | | | | | | | 1 |  | LF |  | 09/03/2021 09:57 |  |
|  | The American Society of Clinical Oncology meeting is the field's most important gathering each spring, providing a stage for major pharmaceutical companies to unveil major findings and tout promising treatments. It's also an annual opportunity for all kinds of researchers, doctors, executives and investors to rub elbows. With COVID-19 making travel uncomfortable and splashy conferences impossible this year, the summit was mostly a virtual affair.  Still, it delivered many of the kinds of important victories and stinging setbacks it often does. And the meeting showed that even as the drug industry races to identify virus treatments and vaccines, cancer[1] remains perhaps its most important business overall. "When the coronavirus wanes and we have a vaccine, and this infectious disease is brought under control, we will still have cancer and the need for new treatments," said Richard Schilsky, ASCO's chief medical officer, in an interview. "We have millions of patients around the world who need new and improved treatments for cancer." | | | | | | | | | | | | | | | |  |
|  |  |  |  |  |  |  |  |  |  |  |  |  |  |  |  |  |  |
|  |  | | | | | | | | | | | | | | | |  |
|  | | **PDF** | | | | | | | | | | | | | | |  |
|  | | | **Files\\118 [1 May 20]** | | | | | | | | | | | | | |  |
|  |  |  | No |  | All articles |  | 0.0168 |  | 1 | |  | | | | | |  |
|  | | |  |  |  |  |  |  |  | |  | | | | | | |
|  | | | | | | | | | | | | 1 |  | LF |  | 15/02/2021 15:35 |  |
|  | One, scientific meetings and medical meetings are still taking place. They may be a little bit delayed, or in most cases, they're going virtual. We're having one this week in fact. That venue being open allows the scientific community to continue to connect and share information, which in the long run is better for patients.  Here at Lilly. We are not delaying the data that we're disclosing. We're just finding new and creative ways to share them with the broader community. | | | | | | | | | | | | | | | |  |
|  |  | | | | | | | | | | | | | | | |  |
|  | | | | | | | | | | | | | | | | | |
| Reports\\Coding Summary By Code Report | | | | | | | | | | Page 73 of 123 | | | | | | | |
| 14/07/2021 10:54 | | | | | | | | | | | | | | | | | |
|  | | | **Aggregate** |  | **Classification** |  | **Coverage** |  | **Number Of Coding References** | |  | **Reference Number** |  | **Coded By Initials** |  | **Modified On** |  |
|  | | | **Files\\12 [11 Jan 21]** | | | | | | | | | | | | | |  |
|  |  |  | No |  | All articles |  | 0.0126 |  | 1 | |  | | | | | |  |
|  | | |  |  |  |  |  |  |  | |  | | | | | | |
|  | | | | | | | | | | | | 1 |  | LF |  | 15/02/2021 15:51 |  |
|  | An important goal identified by Ribas for the upcoming year is supporting cancer research through the challenges brought on by the pandemic. “As the pandemic began, many of the activities that were considered the norm in cancer research had to be changed,” Ribas said, citing the cancellations or adaptations of scientific conferences as one key example. “The first large virtual conference in the field was the AACR Virtual Annual Meeting, which had to be organized within just a month without any road map for this type of meeting.” Twenty-five smaller conferences organized by the AACR were also converted to virtual meetings in 2020. “It was a big change for the organization and the broader scientific community, but it also provided an opportunity to embrace new technologies to reach more people and make the latest cancer research content available to people around the world,” Ribas noted. | | | | | | | | | | | | | | | |  |
|  |  |  |  |  |  |  |  |  |  |  |  |  |  |  |  |  |  |
|  |  | | | | | | | | | | | | | | | |  |
|  | | | **Files\\121 [no date]** | | | | | | | | | | | | | |  |
|  |  |  | No |  | All articles |  | 0.1531 |  | 1 | |  | | | | | |  |
|  | | |  |  |  |  |  |  |  | |  | | | | | | |
|  | | | | | | | | | | | | 1 |  | LF |  | 15/02/2021 16:24 |  |
|  | For the first time since 2010, the American Society of Clinical Oncology will not hold its annual conference in Chicago's McCormick Place, announcing Tuesday it will move the meeting online in the latest sign the business impact of the coronavirus will extend for months.  "As public health safety measures related to COVID-19 extend, the ASCO Board has concluded that the Annual Meeting, scheduled for May 29-June 2 in Chicago, cannot occur in person as planned," the group said in a Mar. 24 statement. With at least 16 states, including Illinois, now asking their residents to shelter in place, the chances of ASCO holding a meeting that regularly draws tens of thousands of doctors, scientists and researchers as planned was remote. Its cancellation is the latest in a lengthening list of medical meetings to be called of as a result of the coronavirus outbreak. | | | | | | | | | | | | | | | |  |
|  |  |  |  |  |  |  |  |  |  |  |  |  |  |  |  |  |  |
|  |  | | | | | | | | | | | | | | | |  |
|  | **Nodes\\Direct impacts\Forced adaptation of research protocols\Continuity of work\Continuation of research-related work** | | | | | | | | | | | | | | | |  |
|  | | **Document** | | | | | | | | | | | | | | |  |
|  | | | **Files\\291 [5Nov20]** | | | | | | | | | | | | | |  |
|  |  |  | No |  | All articles |  | 0.7862 |  | 1 | |  | | | | | |  |
|  | | |  |  |  |  |  |  |  | |  | | | | | | |
|  | | | | | | | | | | | | 1 |  | LF |  | 17/02/2021 17:25 |  |
|  | The Oncology Development programme is also receiving funding from Innovate UK and Cancer Research UK, as part of Cancer Research UK's Entrepreneurial Programmes Initiative.The aim of the programme is to advance viable oncology projects more quickly to increase their chances of commercial success.Also collaborating on the project is major pharmaceutical companies AstraZeneca, Johnson &#38; Johnson Innovation, GlaxoSmithKline and Roche, as well as The Christie NHS Foundation Trust, Medicines Discovery Catapult and the Manchester Cancer Research Centre.They will support researchers and start-ups looking to develop early-stage innovations within the oncology space.The nine-month programme's ultimate goal is to provide potential investors with detailed plans of how companies are planning to deliver key data and value milestone during the next two to three years.Participating researchers and companies who progress through the programme will be eligible to receive up to 60,000 of grant funding to support further development of their business plans.'This is a really exciting and unique project that will help turn some of the most promising ideas in cancer research today into investable projects that will improve the way that cancer patients are diagnosed and treated during the course of their disease,' said Kath Mackay, managing director of Bruntwood SciTech - Alderley Park.'It's no secret that while the life sciences sector and indeed the wider world has understandably been focused on the pandemic, funding and research into beating cancer has suffered.  Our new programme couldn't be launching at a more critical time and, as with the race to find a Covid-19 vaccine, it signals a major collaborative effort involving global leaders,' she added. | | | | | | | | | | | | | | | |  |
|  |  |  |  |  |  |  |  |  |  |  |  |  |  |  |  |  |  |
|  |  |  |  |  |  |  |  |  |  |  |  |  |  |  |  |  |  |
|  |  | | | | | | | | | | | | | | | |  |
| Reports\\Coding Summary By Code Report | | | | | | | | | | Page 74 of 123 | | | | | | | |
| 14/07/2021 10:54 | | | | | | | | | | | | | | | | | |
|  | | | **Aggregate** |  | **Classification** |  | **Coverage** |  | **Number Of Coding References** | |  | **Reference Number** |  | **Coded By Initials** |  | **Modified On** |  |
|  | | | **Files\\570 [15May20]** | | | | | | | | | | | | | |  |
|  |  |  | No |  | All articles |  | 0.0545 |  | 1 | |  | | | | | |  |
|  | | |  |  |  |  |  |  |  | |  | | | | | | |
|  | | | | | | | | | | | | 1 |  | LF |  | 08/03/2021 15:12 |  |
|  | ric Qumneur, Pharm.D., Ph.D., Executive VP, Chief Scientific Officer of Transgene, added: 'The preclinical results generated with BT-001 have been remarkable and we remain extremely confident in its ability to change the treatment landscape for a significant number of solid tumors. We have submitted our first clinical trial application for BT-001 in March 2020 and are working hard to make sure we can take our first multifunctional Invir.IO™ oncolytic into the clinic before the end of 2020, despite uncertainties caused by the Covid-19 pandemic.' | | | | | | | | | | | | | | | |  |
|  |  | | | | | | | | | | | | | | | |  |
|  | | | **Files\\89 [2June20]** | | | | | | | | | | | | | |  |
|  |  |  | No |  | All articles |  | 0.0517 |  | 1 | |  | | | | | |  |
|  | | |  |  |  |  |  |  |  | |  | | | | | | |
|  | | | | | | | | | | | | 1 |  | LF |  | 09/03/2021 09:57 |  |
|  | Even Gilead Sciences Inc., which has been in the headlines because of its potential coronavirus treatment remdesivir, has been getting cancer-focused deals done against the backdrop of the pandemic. Last week, Gilead agreed to work on immunotherapy drugs with biotech Arcus Biosciences Inc. And earlier this year it agreed to buy cancer-drug maker Forty Seven for $4.9 billion. | | | | | | | | | | | | | | | |  |
|  |  | | | | | | | | | | | | | | | |  |
|  | | **PDF** | | | | | | | | | | | | | | |  |
|  | | | **Files\\118 [1 May 20]** | | | | | | | | | | | | | |  |
|  |  |  | No |  | All articles |  | 0.0287 |  | 2 | |  | | | | | |  |
|  | | |  |  |  |  |  |  |  | |  | | | | | | |
|  | | | | | | | | | | | | 1 |  | LF |  | 15/02/2021 15:32 |  |
|  | We are doing a lot to combat the COVID-19 crisis, but rest assured that's not all that we're doing. The work that we otherwise would be doing as a company in many ways continues. Lilly is working with institutions globally to minimize the impact of the pandemic on ongoing research efforts. | | | | | | | | | | | | | | | |  |
|  |  | | | | | | | | | | | | | | | |  |
|  | | | | | | | | | | | | 2 |  | LF |  | 15/02/2021 15:37 |  |
|  | Christian Nguyen (10:47):  Yes, first and foremost, I want you to know that for us working from home does not mean the work has stopped at all. We continue to do very important work. There are literally hundreds, if not thousands, of employees within our company, across the whole industry who are still planning for and preparing for new trials. We're still writing protocols. We're still putting things in place such that when we do come back we're ready to do it as soon as possible, because we know people are waiting. | | | | | | | | | | | | | | | |  |
|  |  | | | | | | | | | | | | | | | |  |
|  | | | **Files\\119 (2) [no date]** | | | | | | | | | | | | | |  |
|  |  |  | No |  | All articles |  | 0.1067 |  | 1 | |  | | | | | |  |
|  | | |  |  |  |  |  |  |  | |  | | | | | | |
|  | | | | | | | | | | | | 1 |  | LF |  | 15/02/2021 16:26 |  |
|  | Although many of us have faced the unpleasant reality of having our laboratories impacted by regulatory authority decisions which defer NonCOVID19 research, cancer cannot wait and neither can our research. Our research groups have taken the necessary steps to secure our infrastructure, protect our research nurses, technicians, staff and trainees while allowing for critical research projects to continue. Patients enrolled in clinical trials continue to receive the best possible care both in person and via telemedicine options. Our laboratories continue to hold weekly lab meetings. We exchange project ideas, collect and analyze data, as well as continue to write, submit and review manuscripts to advance the scientific process. Within the forums and committees of our society, we work on research education, guidelines and white papers. | | | | | | | | | | | | | | | |  |
|  |  | | | | | | | | | | | | | | | |  |
| Reports\\Coding Summary By Code Report | | | | | | | | | | Page 75 of 123 | | | | | | | |
| 14/07/2021 10:54 | | | | | | | | | | | | | | | | | |
|  | | | **Aggregate** |  | **Classification** |  | **Coverage** |  | **Number Of Coding References** | |  | **Reference Number** |  | **Coded By Initials** |  | **Modified On** |  |
|  | | | **Files\\12 [11 Jan 21]** | | | | | | | | | | | | | |  |
|  |  |  | No |  | All articles |  | 0.0179 |  | 1 | |  | | | | | |  |
|  | | |  |  |  |  |  |  |  | |  | | | | | | |
|  | | | | | | | | | | | | 1 |  | LF |  | 15/02/2021 15:47 |  |
|  | 2020 was filled with unexpected challenges for cancer research and patient care. As many of us shifted our lives online in the wake of the COVID-19 pandemic, cancer research was paused, clinical trials were put on hold, appointments were rescheduled, and conferences were canceled or reformatted.  All this occurred amidst a global pandemic that has killed more than 1.9 million people worldwide, a plummeting economy with record unemployment levels, and a national reckoning with racism and racial disparities in all facets of society—including health care.  But there was also progress.  An unprecedented level of scientific collaboration and dissemination led to rapid research advances, culminating in the authorization of two vaccines against SARS-CoV-2 less than 12 months after the first reported COVID-19 cases. Twenty-one novel oncology drugs were approved by the U.S. Food and Drug Administration (FDA) in 2020, including for difficult-to-treat cancers such as triple-negative breast cancer and certain gastrointestinal stromal tumors. The first liquid biopsy next-generation sequencing tests were approved, the first-in-human trial of off-the-shelf CAR T-cell therapy was launched, and the first comprehensive report on cancer disparities was released by the AACR. | | | | | | | | | | | | | | | |  |
|  |  |  |  |  |  |  |  |  |  |  |  |  |  |  |  |  |  |
|  |  | | | | | | | | | | | | | | | |  |
|  | | | **Files\\19 [no date]** | | | | | | | | | | | | | |  |
|  |  |  | No |  | All articles |  | 0.4694 |  | 1 | |  | | | | | |  |
|  | | |  |  |  |  |  |  |  | |  | | | | | | |
|  | | | | | | | | | | | | 1 |  | LF |  | 17/02/2021 11:21 |  |
|  | We all have been affected by the COVID-19 pandemic, and the Ludwig Cancer Research community is doing its part to address the setbacks caused by this unprecedented global crisis. As part of that effort, and to ensure the wellbeing of our teams, the staff at our New York and Zurich o~ces have been working remotely to help limit the transmission of the coronavirus in our respective regions. Scientists at Ludwig Branches and Centers are following the guidance of their host institutions. It is during such challenging times that we are reminded of the vital importance of scienti~c and public health research. We remain con~dent that the scientists and physicians now on the front lines of the public health response will furnish a lasting solution to the unfolding pandemic. Until they do, we will adapt to the changes induced by this crisis—not least, the disruptions to ongoing research— and press forward in our commitment to ease the suffering caused by cancer. | | | | | | | | | | | | | | | |  |
|  |  |  |  |  |  |  |  |  |  |  |  |  |  |  |  |  |  |
|  |  | | | | | | | | | | | | | | | |  |
|  | **Nodes\\Direct impacts\Forced adaptation of research protocols\Expedited approval for new drugs** | | | | | | | | | | | | | | | |  |
|  | | **Document** | | | | | | | | | | | | | | |  |
|  | | | **Files\\521 [8Sept20]** | | | | | | | | | | | | | |  |
|  |  |  | No |  | All articles |  | 0.1578 |  | 1 | |  | | | | | |  |
|  | | |  |  |  |  |  |  |  | |  | | | | | | |
|  | | | | | | | | | | | | 1 |  | LF |  | 08/03/2021 14:29 |  |
|  | In addition to these medicines, SMC has also accepted the following medicines through an expedited approach to minimise delay in patient access following the early phase of the COVID-19 pandemic: Roche's Perjeta (pertuzumab) for the adjuvant treatment of early stage breast cancerSanofi's Cablivi (caplacizumab) for a blood clotting disorder known as acquired thrombotic thrombocytopenic purpura (aTTP)AstraZeneca's Lokelma (sodium zirconium cyclosilicate) for the treatment of hyperkalaemiaNovartis' Beovu (brolucizumab) for the treatment of the 'wet' form of age-related macular degeneration (AMD) The following medicines have been accepted for interim use subject to ongoing evaluation and future reassessment by SMC: Portola's Ondexxya (andexanet alfa) to stop life-threatening or uncontrolled bleeding in adults taking certain medicines used to thin the bloodRoche's Polivy (polatuzumab) to treat a type of lymphoma in patients who are unsuitable for a stem cell transplantChiesi's Holoclar, a stem-cell treatment used to replace damaged cells in the eye after chemical or physical burns | | | | | | | | | | | | | | | |  |
|  |  |  |  |  |  |  |  |  |  |  |  |  |  |  |  |  |  |
|  |  | | | | | | | | | | | | | | | |  |
|  | | | | | | | | | | | | | | | | | |
| Reports\\Coding Summary By Code Report | | | | | | | | | | Page 76 of 123 | | | | | | | |
| 14/07/2021 10:54 | | | | | | | | | | | | | | | | | |
|  | | | **Aggregate** |  | **Classification** |  | **Coverage** |  | **Number Of Coding References** | |  | **Reference Number** |  | **Coded By Initials** |  | **Modified On** |  |
|  | **Nodes\\Direct impacts\Forced adaptation of research protocols\Implementation of new technologies by necessity** | | | | | | | | | | | | | | | |  |
|  | | **Document** | | | | | | | | | | | | | | |  |
|  | | | **Files\\202 [3Nov20]** | | | | | | | | | | | | | |  |
|  |  |  | No |  | All articles |  | 0.2641 |  | 1 | |  | | | | | |  |
|  | | |  |  |  |  |  |  |  | |  | | | | | | |
|  | | | | | | | | | | | | 1 |  | LF |  | 17/02/2021 11:55 |  |
|  | The COVID-19 pandemic had led hospitals to invest in telemedicine or virtual care, which has helped patients[3] who need medical attentionbut notneedless visits.An article by the American Association of Retired Persons (AARP[4]) discussed what patients might get from virtual care:Telemedicine cuts the waiting and travel time of patients using online appointments. Be sure to be prepared and avoid being late on the day of your appointment.High-tech gadgets may let doctors and nurses check on their patients in real time without their physical presence. This lets them know what's happening to thosewith chronic conditions. If something goes wrong, they can act immediately.But, for acough or mild fever, patients can report itfirst online. They no longer have to leave their house to seek medical treatment immediately; the consulting doctor can advise on what the next stepshould be.And if patients have internet service and a smartphone, they can use themto schedule prescription refills.Overall, digital systems in healthcare may work in your favor. You can consult your doctor in just a few taps without going through the hassles of in-person visits. But remember, telemedicine still does not replace the benefits of regular in-person doctorvisits. | | | | | | | | | | | | | | | |  |
|  |  |  |  |  |  |  |  |  |  |  |  |  |  |  |  |  |  |
|  |  | | | | | | | | | | | | | | | |  |
|  | | | **Files\\265 [31July20]** | | | | | | | | | | | | | |  |
|  |  |  | No |  | All articles |  | 0.1338 |  | 1 | |  | | | | | |  |
|  | | |  |  |  |  |  |  |  | |  | | | | | | |
|  | | | | | | | | | | | | 1 |  | LF |  | 17/02/2021 15:34 |  |
|  | Lead researcher Professor Rebecca Fitzgerald, based at the University of Cambridge and funded by Cancer Research UK, said: "It's taken almost a decade of research and testing thousands of patients to show that we've developed a better route to diagnosing Barrett's esophagus. And the sponge could also be a game-changer in how we diagnose and ensure more people survive esophageal cancer. Compared with endoscopies performed in hospital, the Cytosponge causes minimal discomfort and is a quick, simple test[4] that can be done by a GP. Our test is already being piloted around the country, so we hope more people across the UK could benefit from it." Because COVID-19 has reduced the number of endoscopies that can be carried out by the NHS, one UK hospital has already fast-tracked the Cytosponge into use in order to help identify priority cases with suspected cancer who need further tests urgently. | | | | | | | | | | | | | | | |  |
|  |  |  |  |  |  |  |  |  |  |  |  |  |  |  |  |  |  |
|  |  | | | | | | | | | | | | | | | |  |
|  | | | **Files\\273 [8Jun20]** | | | | | | | | | | | | | |  |
|  |  |  | No |  | All articles |  | 0.4200 |  | 2 | |  | | | | | |  |
|  | | |  |  |  |  |  |  |  | |  | | | | | | |
|  | | | | | | | | | | | | 1 |  | LF |  | 17/02/2021 16:57 |  |
|  | A new approach to better select breast cancer patients in need of urgent surgery or chemotherapy during the COVID-19 pandemic has been developed by researchers at The Royal Marsden and the Breast Cancer Now Research Centre at The Institute of Cancer Research, London, in collaboration with colleagues in the UK, Germany and US. The innovative algorithm, using data from multiple international trials, can identify postmenopausal patients with primary ER+HER2- breast cancer[1] (c.70% of cases) who have less endocrine-sensitive tumors and who should be prioritized for early surgery[2] or neoadjuvant chemotherapy[3]. The COVID-19 pandemic has led to an international need to prioritize the number of cancer surgeries and chemotherapy treatments to the most urgent patients to protect staff and vulnerable patients. While patients diagnosed with triple negative and Her2 positive breast cancer have still been going forward for urgent surgery or chemotherapy, for a large group of patients deferring these treatments and prescribing neoadjuvant endocrine therapy (NeoET), i.e. treatment to reduce the stimulation of the disease by estrogen without the surgical removal of the breast tumor, has been identified as the best course of treatment. | | | | | | | | | | | | | | | |  |
|  |  |  |  |  |  |  |  |  |  |  |  |  |  |  |  |  |  |
|  |  | | | | | | | | | | | | | | | |  |
|  | | | | | | | | | | | | | | | | | |
| Reports\\Coding Summary By Code Report | | | | | | | | | | Page 77 of 123 | | | | | | | |
| 14/07/2021 10:54 | | | | | | | | | | | | | | | | | |
|  | | | **Aggregate** |  | **Classification** |  | **Coverage** |  | **Number Of Coding References** | |  | **Reference Number** |  | **Coded By Initials** |  | **Modified On** |  |
|  | | | | | | | | | | | | | | | | | |
|  | | | | | | | | | | | | 2 |  | LF |  | 17/02/2021 16:58 |  |
|  | "The speed and openness of this collaboration to help our patients as rapidly as possible has been unparalleled in my 30 years' experience." Consultant Breast Surgeon at The Royal Marsden, Peter Barry said: "It is important we treat as many patients that require urgent treatment /surgery as safely as possible during the COVID-19 pandemic. This innovative algorithm will help clinicians offer the best treatment for their patients during these unprecedented times. I have already identified patients that would have been deferred to receive NeoET, that may well have been at risk of progression within the six months." Baroness Delyth Morgan, Chief Executive at Breast Cancer Now, said: "It's fantastic that this approach could help guide the best possible treatment for thousands of NHS breast[5] cancer[6] patients during the pandemic, and could also now help inform best practice globally. "This landmark guidance could now help to identify women that must be prioritized for surgery or chemotherapy urgently, and those that could safely be given hormone therapy to delay further treatment during the pandemic. It is a real testament not only to UK science but to the rapid collaboration of researchers globally to help ensure breast cancer patients[7] can get the best possible care while minimizing the risks to them at this time." | | | | | | | | | | | | | | | |  |
|  |  |  |  |  |  |  |  |  |  |  |  |  |  |  |  |  |  |
|  |  | | | | | | | | | | | | | | | |  |
|  | **Nodes\\Direct impacts\Forced adaptation of research protocols\Protection of research capacity by public institutions** | | | | | | | | | | | | | | | |  |
|  | | **Document** | | | | | | | | | | | | | | |  |
|  | | | **Files\\184 [8Oct20]** | | | | | | | | | | | | | |  |
|  |  |  | No |  | All articles |  | 0.7511 |  | 1 | |  | | | | | |  |
|  | | |  |  |  |  |  |  |  | |  | | | | | | |
|  | | | | | | | | | | | | 1 |  | LF |  | 17/02/2021 11:16 |  |
|  | Today, the U.S. Food and Drug Administration announced that it has awarded six new clinical trial research grants to principal investigators from academia and industry totaling over $16 million over the next four years. These trial research grants, awarded through the Congressionally-funded Orphan Products Grants Program[1], enhance the development of medical products for patients with rare diseases.'Now, more than ever, we see the important role of these FDA grants to support clinical trials of potentially life-changing treatments for patients with rare diseases,' said FDA Commissioner Stephen M. Hahn, M.D. 'As interest in the program has grown, so has the hope for promising approved therapies for patients with rare diseases that currently have no treatment options. These important clinical trials are at a critical time where additional resources are needed to support rare disease research during the COVID-19 pandemic.'The FDA received 47 clinical trial grant applications that were reviewed and evaluated for scientific and technical merit by more than 90 rare disease and clinical trial experts, including members of academia.  The grants awarded support clinical studies of products that address unmet needs in rare diseases or conditions, or provide highly significant improvements in treatment or diagnosis. Below is a complete list in alphabetical order: Acucela, Inc. (Seattle, Washington), Ryo Kubota, phase 3 study of emixustat hydrochloride for the treatment of Stargardt disease - $1.6 million over three years. Fred Hutchinson Cancer Research Center (Seattle, Washington), Stephanie Lee, phase 2 study of ustekinumab for the prevention of graft versus host disease - $3.5 million over four years. Seattle Children's Hospital (Seattle, Washington), Christopher Goss, phase 1b study of IV gallium nitrate for the treatment of cystic fibrosis patients colonized with nontuberculosis mycobacterium - $3 million over four years. State University of New York Stony Brook (Stony Brook, New York), Huda Salman, phase 1 study of CD4 redirected chimeric antigen receptor T cell therapy for the treatment of CD4 positive T cell neoplasms - $3.1 million over four years. University of Cincinnati (Cincinnati, Ohio), Devendra Sohal, phase 1/2 study of ABTL0812 (a small molecule with anti-cancer activity) for the treatment of pancreatic cancer - $1.9 million over four years. University of Virginia (Charlottesville, Virginia), Owen O'Connor, phase 2 study of oral azacytidine plus romidepsin for the treatment of peripheral T-cell lymphoma - $3.2 million over four years.'Since its inception over 35 years ago, the Office of Orphan Products Development[2] has successfully continued to support necessary research for rare diseases to inform product development, support scientific advancements, contribute to the development of treatment guidelines, and result in marketing approvals especially for rare diseases without prior-approved therapies. This grants program continues to fill important gaps in rare disease product development, such as supporting recent FDA approvals that help address unmet needs in the treatment of rare diseases,' said Janet Maynard, M.D., M.H.S., director of OOPD.Examples of recent approvals supported by the grants program include teprotumumab, for the treatment of a rare thyroid eye disease, and triheptanoin, a source of calories and fatty acids for the treatment of pediatric and adult patients with molecularly confirmed long-chain fatty acid oxidation disorders.This year, as there are new challenges and increased costs for clinical trials due to the COVID-19 pandemic, the FDA remains committed to supporting rare disease research by providing existing grantees with additional funding. These new resources allow ongoing studies to implement necessary steps to allow their research to continue and assure the safety of study participants, to maintain compliance with good clinical practice, and to minimize risks to trial integrity. | | | | | | | | | | | | | | | |  |
|  |  |  |  |  |  |  |  |  |  |  |  |  |  |  |  |  |  |
|  |  |  |  |  |  |  |  |  |  |  |  |  |  |  |  |  |  |
|  |  |  |  |  |  |  |  |  |  |  |  |  |  |  |  |  |  |
|  |  |  |  |  |  |  |  |  |  |  |  |  |  |  |  |  |  |
|  |  | | | | | | | | | | | | | | | |  |
| Reports\\Coding Summary By Code Report | | | | | | | | | | Page 78 of 123 | | | | | | | |
| 14/07/2021 10:54 | | | | | | | | | | | | | | | | | |
|  | | | **Aggregate** |  | **Classification** |  | **Coverage** |  | **Number Of Coding References** | |  | **Reference Number** |  | **Coded By Initials** |  | **Modified On** |  |
|  | | **PDF** | | | | | | | | | | | | | | |  |
|  | | | **Files\\13 [1 June 20]** | | | | | | | | | | | | | |  |
|  |  |  | No |  | All articles |  | 0.0935 |  | 1 | |  | | | | | |  |
|  | | |  |  |  |  |  |  |  | |  | | | | | | |
|  | | | | | | | | | | | | 1 |  | LF |  | 15/02/2021 16:58 |  |
|  | As the impact of the COVID-19 pandemic continues to grow, with labs shuttered and clinical trials on hold, investigators' worries about the long-term effects on grants and funding are mounting.  “I think there are going to be wide, sweeping effects,” says Eileen White, PhD, deputy director of the Rutgers Cancer Institute of New Jersey in New Brunswick. “This is going to have an impact at an institutional and at a federal level. I don't think we know what the impact will be, but I think everyone is bracing for a difficult time.”  The NIH and NCI have released guidelines about federal grants and funding during the pandemic. “As a general principle, the NCI is trying to provide maximal flexibility to investigators,” says the agency's director, Norman “Ned” Sharpless, MD. “We really want to avoid work stoppages, we want to avoid layoffs, we want to avoid a loss of research capacity for cancer research.”  To that end, the agencies are extending deadlines on grant applications and reporting requirements and are offering research extensions. The agencies are also providing flexibility on how money is spent, allowing investigators to use grants to pay salaries and stipends or to cover unanticipated research costs. “I think it will be enormously helpful,” White says.  PDF Help | | | | | | | | | | | | | | | |  |
|  |  |  |  |  |  |  |  |  |  |  |  |  |  |  |  |  |  |
|  |  | | | | | | | | | | | | | | | |  |
|  | **Nodes\\Direct impacts\Impacts on cancer diagnosis, cases, and services\Decrease in diagnoses or screening** | | | | | | | | | | | | | | | |  |
|  | | **Document** | | | | | | | | | | | | | | |  |
|  | | | **Files\\115 [22 July 20]** | | | | | | | | | | | | | |  |
|  |  |  | No |  | All articles |  | 0.4806 |  | 1 | |  | | | | | |  |
|  | | |  |  |  |  |  |  |  | |  | | | | | | |
|  | | | | | | | | | | | | 1 |  | LF |  | 15/02/2021 15:08 |  |
|  | Alarm bells are ringing again over the impact of the COVID-19 pandemic on cancer care in the UK, after Cancer Research UK unveiled new data showing a steep rise in the number of people waiting for diagnostic tests, and a study published in The Lancet warned that thousands may lose their lives because of the growing bottleneck.According to the charity, at the end of May there were more than 180,000 people in England waiting for an endoscopy, marking a rise of 44% over the same time last year. Of these, 66% have been waiting six weeks or longer for testing.The data also show that, compared to last year: 51% more people are waiting for colonoscopies and 46% more for flexi-sigmoidoscopies (used to detect bowel cancer); 44% more patients are waiting for gastroscopies (used to diagnose oesophageal and stomach cancer); and 23% more people are waiting for a cystoscopy (used for bladder cancer diagnosis).CR UK said it is particularly concerned about these cancer types because endoscopies 'are proving particularly challenging to get back on track', as they are more invasive and need more stringent infection control measures.However, the findings indicate that there are significant issues across the cancer diagnosis spectrum, as around 2.3 million fewer tests that help detect the disease have taken place since lockdown compared to the same time last year.From March 1 to May 30 there was a 46% drop in numbers for seven tests that are commonly used to help spot the disease, including MRI scans, CT scans and ultrasound, as well as endoscopies, the charity revealed.'We're over the peak of the pandemic now, so it's worrying there is an increasing number of patients whose lives are on pause while they wait for tests that could impact their chances of survival,' said Michelle Mitchell, CR UK's chief executive.'It's crucial the government works closely with the NHS to ensure it has the staff and equipment it needs to get services back on track before this situation gets even worse.  But part of the reason the number of tests has reduced so dramatically is that people are delaying seeking help if they are worried about symptoms. | | | | | | | | | | | | | | | |  |
|  |  |  |  |  |  |  |  |  |  |  |  |  |  |  |  |  |  |
|  |  |  |  |  |  |  |  |  |  |  |  |  |  |  |  |  |  |
|  |  | | | | | | | | | | | | | | | |  |
|  | | | | | | | | | | | | | | | | | |
| Reports\\Coding Summary By Code Report | | | | | | | | | | Page 79 of 123 | | | | | | | |
| 14/07/2021 10:54 | | | | | | | | | | | | | | | | | |
|  | | | **Aggregate** |  | **Classification** |  | **Coverage** |  | **Number Of Coding References** | |  | **Reference Number** |  | **Coded By Initials** |  | **Modified On** |  |
|  | | | **Files\\127 [21 Apr 20]** | | | | | | | | | | | | | |  |
|  |  |  | No |  | All articles |  | 0.0381 |  | 1 | |  | | | | | |  |
|  | | |  |  |  |  |  |  |  | |  | | | | | | |
|  | | | | | | | | | | | | 1 |  | LF |  | 15/02/2021 16:47 |  |
|  | KHN interviewed Dr.Len Lichtenfeld[7], the American Cancer Society's deputy chief medical officer and he others observed: 'This is uncharted territory for cancer care Hospitals are making these 'decisions on the fly' in response to how the pandemic looks in a particular community. 'There is no single national standard that can be applied. I am afraid this is going to become much more common in the coming weeks.' The cancer societyrecommends[8]that people postpone their routine cancer screenings — for now. | | | | | | | | | | | | | | | |  |
|  |  | | | | | | | | | | | | | | | |  |
|  | | | **Files\\172 [2June2020]** | | | | | | | | | | | | | |  |
|  |  |  | No |  | All articles |  | 0.8369 |  | 1 | |  | | | | | |  |
|  | | |  |  |  |  |  |  |  | |  | | | | | | |
|  | | | | | | | | | | | | 1 |  | LF |  | 17/02/2021 10:51 |  |
|  | Around 2.4 million people in the UK are currently waiting for cancer screening, tests or cancer treatment, a new analysis by Cancer Research UK has found.The charity says COVID-19 has caused 'enormous disruption' to cancer services throughout the UK, with delays to cancer treatment, screening and diagnosis, and 'huge decreases' in urgent referrals to hospital of patients with suspected cancer symptoms.The charity has found a backlog of around 2.1 million people left waiting for breast, bowel or cervical screening, and notes that during this time, 3,800 cancers would normally be diagnosed through screening.Urgent cancer referrals have been 'severely impacted' with up to 290,000 people missing out on further testing, which would detect up to 20,300 cancers in the same time period, it warns, also highlighting a backlog of treatment to catch up on, with up to 12,750 fewer patients receiving surgery, 6,000 fewer for chemotherapy and 2,800 fewer receiving radiotherapy since lockdown began.The charity says 'COVID-protected' safe spaces will be a crucial part of tackling this backlog and ensuring more people can safely receive treatment or be diagnosed quickly, but stresses that this will only be feasible if all cancer patients and healthcare staff - whether symptomatic or asymptomatic - are tested regularly for the virus.It also calls for a 'clear national plan' for testing to facilitate the recovery and restoration of cancer services across the country.'The enormous strain COVID-19 has placed on cancer services is of great concern to us,' said Michelle Mitchell, Cancer Research UK's chief executive.'To get cancer services back to normal levels while ensuring no one is put at risk, frequent testing of NHS staff and patients, including those without symptoms, is vital.  We now need clear national leadership and guidance for the NHS to dramatically increase testing levels. At the moment, we don't know who is responsible for making cancer services safe and it's patients who are suffering in the meantime. The Government must work closely with the NHS to ramp up testing provision with rapid results, as quickly as possible once practical.'Prompt diagnosis and treatment remain crucial to give people with cancer the greatest chances of survival and prevent the pandemic taking even more lives.' | | | | | | | | | | | | | | | |  |
|  |  |  |  |  |  |  |  |  |  |  |  |  |  |  |  |  |  |
|  |  |  |  |  |  |  |  |  |  |  |  |  |  |  |  |  |  |
|  |  | | | | | | | | | | | | | | | |  |
|  | | | **Files\\18 [17Sept20]** | | | | | | | | | | | | | |  |
|  |  |  | No |  | All articles |  | 0.1562 |  | 1 | |  | | | | | |  |
|  | | |  |  |  |  |  |  |  | |  | | | | | | |
|  | | | | | | | | | | | | 1 |  | LF |  | 17/02/2021 10:55 |  |
|  | National Cancer Institute Director Ned Sharpless told scientists at a July roundtable that just looking at two cancers (breast and colorectal), there will likely be 10,000 additional deaths over the next decade due to the drop in screening and treatments during the pandemic. "One thing we're very worried about in particular is the impact of hospital closures and reduced clinical capacity on patients with cancer, the reductions in screenings, the reductions in patient care," Sharpless said.Carpten, who also is chair of both the report's steering committee and the AACR Minorities in Cancer Research Council, agrees. "If you had to put off your diagnosis by even six months, that cancer could grow and progress and maybe even metastasize in that timeframe." Cancer research has also seen an "unprecedented disruption[3]" from the pandemic, NIAID Director Anthony Fauci has said. | | | | | | | | | | | | | | | |  |
|  |  |  |  |  |  |  |  |  |  |  |  |  |  |  |  |  |  |
|  |  | | | | | | | | | | | | | | | |  |
|  | | | | | | | | | | | | | | | | | |
|  | | | | | | | | | | | | | | | | | |
| Reports\\Coding Summary By Code Report | | | | | | | | | | Page 80 of 123 | | | | | | | |
| 14/07/2021 10:54 | | | | | | | | | | | | | | | | | |
|  | | | **Aggregate** |  | **Classification** |  | **Coverage** |  | **Number Of Coding References** | |  | **Reference Number** |  | **Coded By Initials** |  | **Modified On** |  |
|  | | | **Files\\203 [4June20]** | | | | | | | | | | | | | |  |
|  |  |  | No |  | All articles |  | 0.0676 |  | 1 | |  | | | | | |  |
|  | | |  |  |  |  |  |  |  | |  | | | | | | |
|  | | | | | | | | | | | | 1 |  | LF |  | 17/02/2021 11:58 |  |
|  | Cancer Research UKhaspublished figuresestimating thatmore thantwo million people in the UK have found themselveswaiting for screening, tests or treatments for cancer. Itschief clinician,Charles Swanton, says of the situation: 'Delays to diagnosis and treatment could mean that some cancers will become inoperable.' For Cancer Research UK, coronavirus testingisvital to getting cancer matters back on track.'We're seeing cancer services across the UK adapting to COVID-19, with COVID-protected safe spaces being set up to allow cancer treatment to be delivered safely,' the charity says. 'But for this to work, it requires regular testing for COVID-19 in all patients and staff working in these places, whether they have COVID-19 symptoms or not.' | | | | | | | | | | | | | | | |  |
|  |  | | | | | | | | | | | | | | | |  |
|  | | | **Files\\26 [23June20]** | | | | | | | | | | | | | |  |
|  |  |  | No |  | All articles |  | 0.0758 |  | 2 | |  | | | | | |  |
|  | | |  |  |  |  |  |  |  | |  | | | | | | |
|  | | | | | | | | | | | | 1 |  | LF |  | 17/02/2021 15:23 |  |
|  | Conversations about COVID-19 are changing. While the impact of COVID-19 on healthcare services across the UK is likely to continue for many months to come, talk is shifting to how to restore healthcare services. But at a time when our health services[1] are recovering from one major health crisis, we are in danger of creating another—a cancer crisis. Over 2 million people in the UK were left waiting for cancer screening[2], tests and treatments in the first 10 weeks of lockdown, and that figure is growing. | | | | | | | | | | | | | | | |  |
|  |  | | | | | | | | | | | | | | | |  |
|  | | | | | | | | | | | | 2 |  | LF |  | 17/02/2021 15:25 |  |
|  | All national cancer screening programs were effectively paused in each UK nation by the coronavirus pandemic, which means up to 1.2 million invitations to take part in bowel, breast and cervical screening were not being sent out each month. | | | | | | | | | | | | | | | |  |
|  |  | | | | | | | | | | | | | | | |  |
|  | | | **Files\\271 [18Sept20]** | | | | | | | | | | | | | |  |
|  |  |  | No |  | All articles |  | 0.1520 |  | 1 | |  | | | | | |  |
|  | | |  |  |  |  |  |  |  | |  | | | | | | |
|  | | | | | | | | | | | | 1 |  | LF |  | 17/02/2021 15:54 |  |
|  | At least 14,000 fewer people have been urgently referred for lung cancer tests since March. Of all cancer types[1], referrals for suspected lung[2] cancer have been the hardest hit according to weekly two-week wait (2ww) figures[3] in England. At the end of August, the number of people sent for urgent review and tests in England was still at only around 60% of pre lockdown figures.  It's thought that initial advice for people to stay at home and isolate if they had a new, continuous cough could have led to some people delaying seeking help. Issues on multiple fronts Our survey of 1,000 GPs across the UK in July revealed problems on multiple fronts. 44% of GPs said fewer people were coming to them with coughs or breathing problems, some of the main symptoms of lung cancer. | | | | | | | | | | | | | | | |  |
|  |  | | | | | | | | | | | | | | | |  |
|  | | | **Files\\288 [18May20]** | | | | | | | | | | | | | |  |
|  |  |  | No |  | All articles |  | 0.0446 |  | 1 | |  | | | | | |  |
|  | | |  |  |  |  |  |  |  | |  | | | | | | |
|  | | | | | | | | | | | | 1 |  | LF |  | 17/02/2021 17:17 |  |
|  | Cancer Research UK, a charity, estimates that 2,000 cancers are falling under the radar each week and could become later inoperable. It is only a matter of time until many non-urgent surgeries become urgent. | | | | | | | | | | | | | | | |  |
|  |  | | | | | | | | | | | | | | | |  |
|  | | | | | | | | | | | | | | | | | |
| Reports\\Coding Summary By Code Report | | | | | | | | | | Page 81 of 123 | | | | | | | |
| 14/07/2021 10:54 | | | | | | | | | | | | | | | | | |
|  | | | **Aggregate** |  | **Classification** |  | **Coverage** |  | **Number Of Coding References** | |  | **Reference Number** |  | **Coded By Initials** |  | **Modified On** |  |
|  | | | **Files\\328 [2June20]** | | | | | | | | | | | | | |  |
|  |  |  | No |  | All articles |  | 0.1372 |  | 1 | |  | | | | | |  |
|  | | |  |  |  |  |  |  |  | |  | | | | | | |
|  | | | | | | | | | | | | 1 |  | LF |  | 19/02/2021 16:42 |  |
|  | 'During the COVID-19 crisis, people often forget to talk about the suffering brought to families around the world by breast cancer. But because of the strain on healthcare systems, mammograms have been cancelled, resulting in breast cancers (which could have been detected at an early, treatable stage) being missed,' CEO and cofounder Jonas Muff told VentureBeat. 'In Germany alone, around 500,000 screenings have not been conducted during the last two months, which has led to approximately 3,500 cancers not being detected as early as they could have been. Similarly, the Dutch Cancer Registry shows a decline of up to 50% diagnosed cancers because of COVID-19 incurred cancellations. Vara is now commercially available in Europe to help screening providers offer timely screenings to women whose exams have been cancelled during the lockdown.' | | | | | | | | | | | | | | | |  |
|  |  |  |  |  |  |  |  |  |  |  |  |  |  |  |  |  |  |
|  |  | | | | | | | | | | | | | | | |  |
|  | | | **Files\\341 [8Oct20]** | | | | | | | | | | | | | |  |
|  |  |  | No |  | All articles |  | 0.1835 |  | 1 | |  | | | | | |  |
|  | | |  |  |  |  |  |  |  | |  | | | | | | |
|  | | | | | | | | | | | | 1 |  | LF |  | 19/02/2021 17:05 |  |
|  | Although the number of people going to their GP with symptoms of cancer and being urgently referred to a specialist is on the up as lockdown eases, the figure is still almost 20% lower than the same time last year.The pandemic has also caused disruption across the entirety of cancer services, ranging from delays in scans to referrals to a cancer specialist.'These cancer charities are needed now more than ever. | | | | | | | | | | | | | | | |  |
|  |  | | | | | | | | | | | | | | | |  |
|  | | | **Files\\362 [12Oct20]** | | | | | | | | | | | | | |  |
|  |  |  | No |  | All articles |  | 0.5625 |  | 1 | |  | | | | | |  |
|  | | |  |  |  |  |  |  |  | |  | | | | | | |
|  | | | | | | | | | | | | 1 |  | LF |  | 23/02/2021 15:22 |  |
|  | Lung cancer screenings have seen a significant decline as a result of the global COVID-19 pandemic, The Guardian reports. According to figures fromCancerResearch UK, about 350,000 fewer people than normal received urgent suspected cancer referrals across all cancers since the end of March, while 3 million people have missed out on cancer screening.Dr Neil Smith, Cancer Research UK's GP for the Lancashire and South Cumbria Cancer Alliance, said the lack of urgent cancer referrals is currently the 'black hole' in cancer services.'The biggest thing I have noticed during coronavirus is that fewer of my patients are actually coming forwards to tell me about the signs and the symptoms of cancer. They seem to be reluctant to do so,' he said. | | | | | | | | | | | | | | | |  |
|  |  | | | | | | | | | | | | | | | |  |
|  | | | **Files\\4 [10Nov20]** | | | | | | | | | | | | | |  |
|  |  |  | No |  | All articles |  | 0.0357 |  | 1 | |  | | | | | |  |
|  | | |  |  |  |  |  |  |  | |  | | | | | | |
|  | | | | | | | | | | | | 1 |  | LF |  | 07/03/2021 11:49 |  |
|  | For some cancer types, such as lung cancer or urological cancers, referral numbers are still well below where they were last year. While trials are getting back up and running, this is happening slower than we would like. And even with increasing activity, there's still huge numbers of people waiting for screening, diagnosis and treatment. | | | | | | | | | | | | | | | |  |
|  |  | | | | | | | | | | | | | | | |  |
|  | | | | | | | | | | | | | | | | | |
|  | | | | | | | | | | | | | | | | | |
| Reports\\Coding Summary By Code Report | | | | | | | | | | Page 82 of 123 | | | | | | | |
| 14/07/2021 10:54 | | | | | | | | | | | | | | | | | |
|  | | | **Aggregate** |  | **Classification** |  | **Coverage** |  | **Number Of Coding References** | |  | **Reference Number** |  | **Coded By Initials** |  | **Modified On** |  |
|  | | | **Files\\512 [10Jun20]** | | | | | | | | | | | | | |  |
|  |  |  | No |  | All articles |  | 0.3723 |  | 1 | |  | | | | | |  |
|  | | |  |  |  |  |  |  |  | |  | | | | | | |
|  | | | | | | | | | | | | 1 |  | LF |  | 08/03/2021 14:13 |  |
|  | A primary challenge will be dealing with the huge backlog of treatment paused during the novel coronavirus pandemic, with the waiting list for routine procedures already overshooting 4 million 'now certain to rise significantly'.The warning follows that from the BHF that 28,000 inpatient heart procedures had been delayed during the outbreak, and that from Cancer Research UK that around 2.4 million people in the UK are currently waiting for cancer screening, tests or treatment.In a letter to the Prime Minister, the NHS Confederation warns 'it will not be possible to simply 'switch on' NHS services immediately', and calls on the government to manage expectations on how quickly key patient services can be restored.The body also calls for an extension to emergency NHS funding, establishing an ongoing arrangement with the private sector to clear the treatment backlog, and a commitment to acknowledge and address health inequalities through upcoming guidance and policy reform.'Political leaders have a vital role to play in reassuring the public that every step possible is being taken to manage the virus, while safely bringing back services that had to be paused. | | | | | | | | | | | | | | | |  |
|  |  |  |  |  |  |  |  |  |  |  |  |  |  |  |  |  |  |
|  |  | | | | | | | | | | | | | | | |  |
|  | | | **Files\\58 [20Jul20]** | | | | | | | | | | | | | |  |
|  |  |  | No |  | All articles |  | 0.1461 |  | 1 | |  | | | | | |  |
|  | | |  |  |  |  |  |  |  | |  | | | | | | |
|  | | | | | | | | | | | | 1 |  | LF |  | 08/03/2021 15:14 |  |
|  | More people are waiting to get tests used to diagnose four common cancers compared to last year, according to Cancer Research UK analysis, as the charity urges the public to support its vital work. As of May 30th, there were more than 180,000 people in England waiting for an endoscopy—a rise of 44% from the same time in 2019. And of these people, 66% are waiting six weeks or longer for these vital tests. Compared to last year:51% more people are waiting for colonoscopies and 46% more for flexi-sigmoidoscopies, which are used to detect bowel cancer44% more patients are waiting for gastroscopies, which help diagnose oesophageal and stomach cancer23% more people are waiting for a cystoscopy, used for bladder cancer diagnosisCancer Research UK is concerned about these cancer types because endoscopies are proving particularly challenging to get back on track. | | | | | | | | | | | | | | | |  |
|  |  |  |  |  |  |  |  |  |  |  |  |  |  |  |  |  |  |
|  |  | | | | | | | | | | | | | | | |  |
|  | | | **Files\\64 [21Jul20]** | | | | | | | | | | | | | |  |
|  |  |  | No |  | All articles |  | 0.0710 |  | 1 | |  | | | | | |  |
|  | | |  |  |  |  |  |  |  | |  | | | | | | |
|  | | | | | | | | | | | | 1 |  | LF |  | 08/03/2021 16:12 |  |
|  | "We know there is going to be an impact on cancer survival from COVID-19, and it can only be a negative impact," says Jon Shelton, Cancer Research UK's senior intelligence manager. "Screening effectively stopped during the pandemic, the number of people being referred for diagnostic tests[3] dropped massively and a lot of tests and treatments for cancer were postponed." Shelton says the impact of COVID-19 has been felt across the board, with certain diagnostic procedures—like endoscopies—being stopped because they were considered too risky[4] for both patients and healthcare staff. | | | | | | | | | | | | | | | |  |
|  |  | | | | | | | | | | | | | | | |  |
|  | | **PDF** | | | | | | | | | | | | | | |  |
|  | | | **Files\\1 (2) [8th Oct 20]** | | | | | | | | | | | | | |  |
|  |  |  | No |  | All articles |  | 0.0134 |  | 1 | |  | | | | | |  |
|  | | |  |  |  |  |  |  |  | |  | | | | | | |
|  | | | | | | | | | | | | 1 |  | LF |  | 15/02/2021 12:02 |  |
|  | On the negative side, studies have shown a sharp decline in cancer diagnoses and screening during the  peak of the pandemic. One study found that routine screening dropped by 85-90 percent. Delays in diagnosing cancer are likely to have long-term ripple eﬀects. | | | | | | | | | | | | | | | |  |
|  |  | | | | | | | | | | | | | | | |  |
| Reports\\Coding Summary By Code Report | | | | | | | | | | Page 83 of 123 | | | | | | | |
| 14/07/2021 10:54 | | | | | | | | | | | | | | | | | |
|  | | | **Aggregate** |  | **Classification** |  | **Coverage** |  | **Number Of Coding References** | |  | **Reference Number** |  | **Coded By Initials** |  | **Modified On** |  |
|  | | | **Files\\117 (2) [17 Dec 20]** | | | | | | | | | | | | | |  |
|  |  |  | No |  | All articles |  | 0.1384 |  | 1 | |  | | | | | |  |
|  | | |  |  |  |  |  |  |  | |  | | | | | | |
|  | | | | | | | | | | | | 1 |  | LF |  | 15/02/2021 15:18 |  |
|  | The current surge in coronavirus disease 2019 (COVID19) cases poses challenges for providers and institutions in delivering care to infected patients while also placing demands on them to keep up with timely and sometimes critical care for patients with cancer, heart disease and other serious illnesses who might experience advanced complications and/or earlier death if they have lapses in their care. Reporting on how deferred care worsened outcomes for lung cancer patients when the COVID-19 pandemic first surged in the spring of 2020, researchers from the University of Cincinnati explained that they have identified a framework that could help people with serious health conditions keep up their appointments during the current surge. The study has been selected for the 2020 Southern Surgical Association Program and published as an “article in press” on the Journal of the American College of Surgeons website in advance of print.  The researchers compared monthly visits for low-dose computed tomography (LDCT) screening for lung cancer during the three months in which COVID-19 restrictions were in place with the number of monthly visits from the three years before that. LDCT is an imaging modality known to reduce mortality from lung cancer by at least 20 percent in high-risk patients. The institution suspended LDCT on March 13 and began a phased reopening on May 5 with a full opening on June 1.  “We had 800 scans cancelled during that time and even during the resumed period, we had a decreased total volume of patients scanned and also noted a decreased number of new patients who were scanned for their lung cancer screening,” said lead author Robert M. Van Haren, MD, MSPH, FACS, an assistant professor and thoracic surgeon at the University of Cincinnati College of Medicine and a member of Cincinnati Research in Outcomes and Safety in Surgery (CROSS) within the department of surgery. | | | | | | | | | | | | | | | |  |
|  |  |  |  |  |  |  |  |  |  |  |  |  |  |  |  |  |  |
|  |  |  |  |  |  |  |  |  |  |  |  |  |  |  |  |  |  |
|  |  | | | | | | | | | | | | | | | |  |
|  | | | **Files\\12 [11 Jan 21]** | | | | | | | | | | | | | |  |
|  |  |  | No |  | All articles |  | 0.0101 |  | 1 | |  | | | | | |  |
|  | | |  |  |  |  |  |  |  | |  | | | | | | |
|  | | | | | | | | | | | | 1 |  | LF |  | 15/02/2021 16:20 |  |
|  | Identifying and addressing the obstacles to cancer screening will also be important. In 2021, a major hurdle will be the ongoing COVID-19 pandemic, which has already led to a dramatic decline in screening rates. “During the early stages of the pandemic, the last thing anyone wanted to do was go to a clinic and risk exposure, so people started putting off routine screenings,” said DuBois. “Now that we’ve realized that we’re going to be living with this virus for some time, we are trying to encourage patients to resume screenings.” Despite this encouragement, screening rates are still far below pre-pandemic levels. “That’s a huge problem, as it means many cancers may not be detected until it’s too late,” DuBois noted. | | | | | | | | | | | | | | | |  |
|  |  | | | | | | | | | | | | | | | |  |
|  | | | **Files\\29 (2) [22Jul20]** | | | | | | | | | | | | | |  |
|  |  |  | No |  | All articles |  | 0.1899 |  | 2 | |  | | | | | |  |
|  | | |  |  |  |  |  |  |  | |  | | | | | | |
|  | | | | | | | | | | | | 1 |  | LF |  | 17/02/2021 17:21 |  |
|  | More than a quarter of the respondents said they had experienced a delay in healthcare:  31.7% of people who had been diagnosed with breast cancer reported a delay in care; of these people, 22% said they had a delay in screening and 9.3% reported a delay in treatment 26.7% of people who had never been diagnosed with breast cancer reported a delay in care; of these people, 31.5% said they had a delay in screening and 1.6% said they had a delay in treatment, though since they hadn’t been diagnosed with breast cancer, the researchers assumed this was a delay in some other type of treatment  “Our survey shows that the COVID-19 pandemic has disrupted breast cancer screening, including surveillance among women who have been diagnosed with breast cancer,” said Erica Warner, MPH, assistant professor of medicine at Harvard Medical School, who presented the results. “Our next steps will be to see if the affect of the pandemic on breast screening differed by demographic, geographic, or other respondent characteristics.”  Warner pointed out that many doctors are concerned about the long-term effects of delays in breast cancer screening.  “Delays in screening can lead to delays in diagnoses,” she said. “In his June 19 editorial in Science, Dr. Norman Sharpless, director of the National Cancer Institute, said that modeling predicts an excess of 10,000 deaths from breast cancer and colorectal cancer over the next 10 years because of the pandemic.  “We need to implement strategies to help people return to care,” she added. “We need to emphasize how important screening is and let them know about the measures being taken to ensure their safety.” | | | | | | | | | | | | | | | |  |
|  |  |  |  |  |  |  |  |  |  |  |  |  |  |  |  |  |  |
|  |  |  |  |  |  |  |  |  |  |  |  |  |  |  |  |  |  |
|  |  | | | | | | | | | | | | | | | |  |
| Reports\\Coding Summary By Code Report | | | | | | | | | | Page 84 of 123 | | | | | | | |
| 14/07/2021 10:54 | | | | | | | | | | | | | | | | | |
|  | | | **Aggregate** |  | **Classification** |  | **Coverage** |  | **Number Of Coding References** | |  | **Reference Number** |  | **Coded By Initials** |  | **Modified On** |  |
|  | | | | | | | | | | | | | | | | | |
|  | | | | | | | | | | | | 2 |  | LF |  | 17/02/2021 17:22 |  |
|  | The second study was done by researchers at the University of Massachusetts Medical School. The researchers looked at the medical records of women and men age 30 to 85 who had not been diagnosed with breast or prostate cancer. By looking at the codes in their medical records, the researchers determined how many people had either breast cancer or prostate cancer screening tests or a biopsy. All the people were patients of the same healthcare group.  The researchers compared the monthly rate per 1,000 people of breast/prostate cancer screening or biopsy from January to May 2019 to the number of people who had the same procedures from January to May 2020.  For this Breastcancer.org review, we will only focus on the breast cancer screening and biopsy results. For this study, breast cancer screening included 2D mammograms and digital tomosynthesis (3D mammograms).  Overall, 80,629 women were included in the 2019 analysis and 82,695 women were included in the 2020 analysis. About half the women were younger than 52 and half the women were older.  The researchers found that breast cancer screening decreased dramatically from 2019 to 2020:  an average of 13.6 2D mammograms per 1,000 women per month were done from January to May 2019 an average of 6.1 2D mammograms per 1,000 women were done in March 2020 an average of 0.25 2D mammograms per 1,000 women were done in April 2020 an average of 1.1 2D mammograms per 1,000 women were done in May 2020 an average of 34.7 3D mammograms per 1,000 women were done from January to May 2019 an average of 14.6 3D mammograms per 1,000 women were done in March 2020 an average of 1.4 3D mammograms per 1,000 women were done in April 2020 an average of 1.5 3D mammograms per 1,000 women were done in May 2020  Breast biopsies also declined from an average of 0.9 per 1,000 women per month in 2019 to:  0.8 per 1,000 women in March 2020 0.4 per 1,000 women in April 2020 0.1 per 1,000 women in May 2020  Mara Epstein, ScD, assistant professor of medicine at the University of Massachusetts, echoed Warner’s concerns about delays in screening leading to delays in diagnosis, which will likely lead to worse breast cancer outcomes.  “In central Massachusetts, we saw a precipitous decline in preventive and diagnostic procedures for breast and prostate cancer during the peak of COVID-19,” said Epstein, who presented the research. “The long-term impact on delay in cancer diagnosis and cancer prognosis is unknown, but Dr. Sharpless’s models estimate a large number of excess deaths.” | | | | | | | | | | | | | | | |  |
|  |  |  |  |  |  |  |  |  |  |  |  |  |  |  |  |  |  |
|  |  |  |  |  |  |  |  |  |  |  |  |  |  |  |  |  |  |
|  |  |  |  |  |  |  |  |  |  |  |  |  |  |  |  |  |  |
|  |  | | | | | | | | | | | | | | | |  |
|  | | | | | | | | | | | | | | | | | |
|  | | | | | | | | | | | | | | | | | |
|  | | | | | | | | | | | | | | | | | |
|  | | | | | | | | | | | | | | | | | |
|  | | | | | | | | | | | | | | | | | |
|  | | | | | | | | | | | | | | | | | |
| Reports\\Coding Summary By Code Report | | | | | | | | | | Page 85 of 123 | | | | | | | |
| 14/07/2021 10:54 | | | | | | | | | | | | | | | | | |
|  | | | **Aggregate** |  | **Classification** |  | **Coverage** |  | **Number Of Coding References** | |  | **Reference Number** |  | **Coded By Initials** |  | **Modified On** |  |
|  | | | **Files\\68 (2) [14May20]** | | | | | | | | | | | | | |  |
|  |  |  | No |  | All articles |  | 0.3532 |  | 1 | |  | | | | | |  |
|  | | |  |  |  |  |  |  |  | |  | | | | | | |
|  | | | | | | | | | | | | 1 |  | LF |  | 08/03/2021 16:39 |  |
|  | Sabine Siesling of the Department of Research and Development, Netherlands Comprehensive Cancer Organisation, Utrecht and the Department of Health Technology and Services Research, Technical Medical Centre, University of Twente in Enschede, Netherlands and colleagues analyzed data from the Netherlands Cancer Registry from 24 February 2020 to 12 April 2020 based on initial case declaration through pathological cancer noti~cations in the Nationwide Network of Histopathology and Cytopathology.  They describe multifactorial reasons for their ~ndings from the point of view of the patient, doctor and healthcare system.  Citizens in the Netherlands have universal access to essential healthcare services, but the general practitioner is a gatekeeper to secondary care. Individuals with non-speci~c cancer symptoms may feel that they are wasting the general practitioner's time on nonCOVID-19-related symptoms. Furthermore, they may assume that essential non-COVID19-related healthcare services are limited. Last, but not least, they may feel anxious about becoming infected with COVID-19 while visiting healthcare facilities.  Most of the general practitioner consultations for non-acute issues were switched to telehealth. Therefore, a general practitioner might postpone initial examinations for symptoms that are not immediately associated with a potential cancer diagnosis, leading to delays in further diagnostic referrels.  In terms of the impact on healthcare facilities, many hospital-based resources have been allocated to dealing with the COVID-19 pandemic. Furthermore, national screening programmes for breast, colorectal and cervical cancer were temporarily halted as of 16 March 2020. The effect of this pause in cancer diagnosis might be more evident after an extended follow-up period.  This observation of fewer cancer diagnoses during the COVID-19 outbreak has been disseminated within the Dutch community by the Netherlands Comprehensive Cancer Organisation, which hosts the Netherlands Cancer Registry, to create awareness of this important issue. Individuals were encouraged to consult their general practitioner in case of continued symptoms. General practitioners were also encouraged to refer patients with suspected cancer to cancer specialistsand an appeal was made to restart national cancer screening programmes. Misconceptions were eliminated about a heightened risk of contracting COVID-19 in a healthcare setting because of inadequate policies for infection control and resource limitations to deliver essential cancer care.  The Netherlands Cancer Registry plans to complete the registration of current and new cases via retrospective review of medical records. More detailed data including COVID19 positivity status, tumour types and details on anticancer treatments, along with furtherfollow-up, will lead to a better understanding of the effects of the COVID-19 outbreak on cancer care in the Netherlands.  Reference  Dinmohamed AG, Visser O, Verhoeven RH, et al. Fewer cancer diagnoses during the COVID-19 epidemic in the Netherlands. The Lancet Oncology; Published online 30 April 2020; DOI: https://doi.org/10.1016/S1470-2045(20)30265-5   Legal Useful links  On 30 April 2020 in The Lancet Oncology, a group of researchers from the Netherlands reported on a notable decrease in cancer diagnoses in the country since the start of the COVID-19 pandemic. Substantial decreases in cancer diagnosis were reported across all age groups, geographical regions and in almost all types of cancer, although a higher | | | | | | | | | | | | | | | |  |
|  |  |  |  |  |  |  |  |  |  |  |  |  |  |  |  |  |  |
|  |  |  |  |  |  |  |  |  |  |  |  |  |  |  |  |  |  |
|  |  |  |  |  |  |  |  |  |  |  |  |  |  |  |  |  |  |
|  |  |  |  |  |  |  |  |  |  |  |  |  |  |  |  |  |  |
|  |  | | | | | | | | | | | | | | | |  |
|  | | | **Files\\74 [14Sept20]** | | | | | | | | | | | | | |  |
|  |  |  | No |  | All articles |  | 0.0132 |  | 1 | |  | | | | | |  |
|  | | |  |  |  |  |  |  |  | |  | | | | | | |
|  | | | | | | | | | | | | 1 |  | LF |  | 08/03/2021 17:23 |  |
|  | The pandemic has also dealt a devastating blow to the delivery of cancer services in the UK. There reasons are many, from services and facilities having to be shut down or run at reduced capacity due to the risk of spreading COVID-19, to the reluctance of patients to visit hospitals or diagnostic centres for fear of catching the virus, or for not wanting to add weight to an already overburdened heath service. | | | | | | | | | | | | | | | |  |
|  |  | | | | | | | | | | | | | | | |  |
|  | | | | | | | | | | | | | | | | | |
|  | | | | | | | | | | | | | | | | | |
| Reports\\Coding Summary By Code Report | | | | | | | | | | Page 86 of 123 | | | | | | | |
| 14/07/2021 10:54 | | | | | | | | | | | | | | | | | |
|  | | | **Aggregate** |  | **Classification** |  | **Coverage** |  | **Number Of Coding References** | |  | **Reference Number** |  | **Coded By Initials** |  | **Modified On** |  |
|  | **Nodes\\Direct impacts\Impacts on cancer diagnosis, cases, and services\Decrease in diagnoses or screening\Hesitancy on the part of the community** | | | | | | | | | | | | | | | |  |
|  | | **Document** | | | | | | | | | | | | | | |  |
|  | | | **Files\\115 [22 July 20]** | | | | | | | | | | | | | |  |
|  |  |  | No |  | All articles |  | 0.4806 |  | 1 | |  | | | | | |  |
|  | | |  |  |  |  |  |  |  | |  | | | | | | |
|  | | | | | | | | | | | | 1 |  | LF |  | 15/02/2021 15:08 |  |
|  | Alarm bells are ringing again over the impact of the COVID-19 pandemic on cancer care in the UK, after Cancer Research UK unveiled new data showing a steep rise in the number of people waiting for diagnostic tests, and a study published in The Lancet warned that thousands may lose their lives because of the growing bottleneck.According to the charity, at the end of May there were more than 180,000 people in England waiting for an endoscopy, marking a rise of 44% over the same time last year. Of these, 66% have been waiting six weeks or longer for testing.The data also show that, compared to last year: 51% more people are waiting for colonoscopies and 46% more for flexi-sigmoidoscopies (used to detect bowel cancer); 44% more patients are waiting for gastroscopies (used to diagnose oesophageal and stomach cancer); and 23% more people are waiting for a cystoscopy (used for bladder cancer diagnosis).CR UK said it is particularly concerned about these cancer types because endoscopies 'are proving particularly challenging to get back on track', as they are more invasive and need more stringent infection control measures.However, the findings indicate that there are significant issues across the cancer diagnosis spectrum, as around 2.3 million fewer tests that help detect the disease have taken place since lockdown compared to the same time last year.From March 1 to May 30 there was a 46% drop in numbers for seven tests that are commonly used to help spot the disease, including MRI scans, CT scans and ultrasound, as well as endoscopies, the charity revealed.'We're over the peak of the pandemic now, so it's worrying there is an increasing number of patients whose lives are on pause while they wait for tests that could impact their chances of survival,' said Michelle Mitchell, CR UK's chief executive.'It's crucial the government works closely with the NHS to ensure it has the staff and equipment it needs to get services back on track before this situation gets even worse.  But part of the reason the number of tests has reduced so dramatically is that people are delaying seeking help if they are worried about symptoms. | | | | | | | | | | | | | | | |  |
|  |  |  |  |  |  |  |  |  |  |  |  |  |  |  |  |  |  |
|  |  |  |  |  |  |  |  |  |  |  |  |  |  |  |  |  |  |
|  |  | | | | | | | | | | | | | | | |  |
|  | | | **Files\\271 [18Sept20]** | | | | | | | | | | | | | |  |
|  |  |  | No |  | All articles |  | 0.1660 |  | 2 | |  | | | | | |  |
|  | | |  |  |  |  |  |  |  | |  | | | | | | |
|  | | | | | | | | | | | | 1 |  | LF |  | 17/02/2021 15:53 |  |
|  | At least 14,000 fewer people have been urgently referred for lung cancer tests since March. Of all cancer types[1], referrals for suspected lung[2] cancer have been the hardest hit according to weekly two-week wait (2ww) figures[3] in England. At the end of August, the number of people sent for urgent review and tests in England was still at only around 60% of pre lockdown figures.  It's thought that initial advice for people to stay at home and isolate if they had a new, continuous cough could have led to some people delaying seeking help. | | | | | | | | | | | | | | | |  |
|  |  | | | | | | | | | | | | | | | |  |
|  | | | | | | | | | | | | 2 |  | LF |  | 17/02/2021 15:54 |  |
|  | But the drop in urgent referrals could also be due to people's concerns about visiting hospital. In July, 63% of GPs surveyed said that some of their patients had not wanted to go to hospital for tests in the previous month, even though most hospitals should have established COVID-protected safe spaces by then. | | | | | | | | | | | | | | | |  |
|  |  | | | | | | | | | | | | | | | |  |
|  | | | **Files\\404 [26Oct20]** | | | | | | | | | | | | | |  |
|  |  |  | No |  | All articles |  | 0.4537 |  | 1 | |  | | | | | |  |
|  | | |  |  |  |  |  |  |  | |  | | | | | | |
|  | | | | | | | | | | | | 1 |  | LF |  | 07/03/2021 11:58 |  |
|  | According to Cancer Research UK, around 47,800 people are diagnosed with lung cancer in the UK every year. But referrals for suspected lung cancer plummeted during lockdown and were still only 60% of pre-Covid levels by the end of August.'It's thought that initial advice for people to stay at home and isolate if they had a new, continuous cough could have led to some people delaying seeking help,'CRUK has warned.'It is a problem,' said Dr David Gilligan, consultant clinical oncologist at Addenbrooke's and Papworth hospitals in Cambridge, and a trustee of the Roy Castle Lung Cancer Foundation. | | | | | | | | | | | | | | | |  |
|  |  | | | | | | | | | | | | | | | |  |
| Reports\\Coding Summary By Code Report | | | | | | | | | | Page 87 of 123 | | | | | | | |
| 14/07/2021 10:54 | | | | | | | | | | | | | | | | | |
|  | | | **Aggregate** |  | **Classification** |  | **Coverage** |  | **Number Of Coding References** | |  | **Reference Number** |  | **Coded By Initials** |  | **Modified On** |  |
|  | | **PDF** | | | | | | | | | | | | | | |  |
|  | | | **Files\\12 [11 Jan 21]** | | | | | | | | | | | | | |  |
|  |  |  | No |  | All articles |  | 0.0101 |  | 1 | |  | | | | | |  |
|  | | |  |  |  |  |  |  |  | |  | | | | | | |
|  | | | | | | | | | | | | 1 |  | LF |  | 15/02/2021 16:20 |  |
|  | Identifying and addressing the obstacles to cancer screening will also be important. In 2021, a major hurdle will be the ongoing COVID-19 pandemic, which has already led to a dramatic decline in screening rates. “During the early stages of the pandemic, the last thing anyone wanted to do was go to a clinic and risk exposure, so people started putting off routine screenings,” said DuBois. “Now that we’ve realized that we’re going to be living with this virus for some time, we are trying to encourage patients to resume screenings.” Despite this encouragement, screening rates are still far below pre-pandemic levels. “That’s a huge problem, as it means many cancers may not be detected until it’s too late,” DuBois noted. | | | | | | | | | | | | | | | |  |
|  |  | | | | | | | | | | | | | | | |  |
|  | | | **Files\\74 [14Sept20]** | | | | | | | | | | | | | |  |
|  |  |  | No |  | All articles |  | 0.0132 |  | 1 | |  | | | | | |  |
|  | | |  |  |  |  |  |  |  | |  | | | | | | |
|  | | | | | | | | | | | | 1 |  | LF |  | 08/03/2021 17:23 |  |
|  | The pandemic has also dealt a devastating blow to the delivery of cancer services in the UK. There reasons are many, from services and facilities having to be shut down or run at reduced capacity due to the risk of spreading COVID-19, to the reluctance of patients to visit hospitals or diagnostic centres for fear of catching the virus, or for not wanting to add weight to an already overburdened heath service. | | | | | | | | | | | | | | | |  |
|  |  | | | | | | | | | | | | | | | |  |
|  | **Nodes\\Direct impacts\Impacts on cancer diagnosis, cases, and services\Decrease in diagnoses or screening\Indirect cancer deaths due to the pandemic** | | | | | | | | | | | | | | | |  |
|  | | **Document** | | | | | | | | | | | | | | |  |
|  | | | **Files\\50 [21May20]** | | | | | | | | | | | | | |  |
|  |  |  | No |  | All articles |  | 0.3791 |  | 1 | |  | | | | | |  |
|  | | |  |  |  |  |  |  |  | |  | | | | | | |
|  | | | | | | | | | | | | 1 |  | LF |  | 08/03/2021 11:19 |  |
|  | Scientists at the Institute of Cancer Research (ICR) are warning that delays to cancer surgery and other treatment caused by the COVID-19 pandemic could be linked to thousands of additional deaths in England.Cancer patients could face delays of several months to their treatment in the wake of the pandemic, including in operations to remove tumours. Those whose cancer will have progressed during the delay and who might otherwise have been cured by surgery could now be at risk of their cancer returning and shortening survival.Scientists at The ICR analysed existing Public Health England data on delays to cancer surgery on patients' five-year survival rates to estimate the effect of three-month or six-month delays, respectively.Their modelling, which took into account the risk of hospital-acquired COVID-19-infection, showed 'dramatic differences in the impact of delay on cancer survival depending on patients' age, their cancer type and whether it was earlier- or later-stage cancer', the Institute said.According to the team's findings, a delay of three months across all 94,912 patients who would have had surgery to remove their cancer over the course of a year would lead to an additional 4,755 deaths. | | | | | | | | | | | | | | | |  |
|  |  |  |  |  |  |  |  |  |  |  |  |  |  |  |  |  |  |
|  |  | | | | | | | | | | | | | | | |  |
|  | | | | | | | | | | | | | | | | | |
| Reports\\Coding Summary By Code Report | | | | | | | | | | Page 88 of 123 | | | | | | | |
| 14/07/2021 10:54 | | | | | | | | | | | | | | | | | |
|  | | | **Aggregate** |  | **Classification** |  | **Coverage** |  | **Number Of Coding References** | |  | **Reference Number** |  | **Coded By Initials** |  | **Modified On** |  |
|  | **Nodes\\Direct impacts\Impacts on cancer diagnosis, cases, and services\Decrease in diagnoses or screening\Levels of screening have not recovered** | | | | | | | | | | | | | | | |  |
|  | | **Document** | | | | | | | | | | | | | | |  |
|  | | | **Files\\4 [10Nov20]** | | | | | | | | | | | | | |  |
|  |  |  | No |  | All articles |  | 0.0357 |  | 1 | |  | | | | | |  |
|  | | |  |  |  |  |  |  |  | |  | | | | | | |
|  | | | | | | | | | | | | 1 |  | LF |  | 07/03/2021 11:49 |  |
|  | For some cancer types, such as lung cancer or urological cancers, referral numbers are still well below where they were last year. While trials are getting back up and running, this is happening slower than we would like. And even with increasing activity, there's still huge numbers of people waiting for screening, diagnosis and treatment. | | | | | | | | | | | | | | | |  |
|  |  | | | | | | | | | | | | | | | |  |
|  | | **PDF** | | | | | | | | | | | | | | |  |
|  | | | **Files\\12 [11 Jan 21]** | | | | | | | | | | | | | |  |
|  |  |  | No |  | All articles |  | 0.0072 |  | 1 | |  | | | | | |  |
|  | | |  |  |  |  |  |  |  | |  | | | | | | |
|  | | | | | | | | | | | | 1 |  | LF |  | 15/02/2021 16:20 |  |
|  | “During the early stages of the pandemic, the last thing anyone wanted to do was go to a clinic and risk exposure, so people started putting off routine screenings,” said DuBois. “Now that we’ve realized that we’re going to be living with this virus for some time, we are trying to encourage patients to resume screenings.” Despite this encouragement, screening rates are still far below pre-pandemic levels. “That’s a huge problem, as it means many cancers may not be detected until it’s too late,” DuBois noted. | | | | | | | | | | | | | | | |  |
|  |  | | | | | | | | | | | | | | | |  |
|  | **Nodes\\Direct impacts\Impacts on cancer diagnosis, cases, and services\Decrease in diagnoses or screening\Suspension of screening programs** | | | | | | | | | | | | | | | |  |
|  | | **Document** | | | | | | | | | | | | | | |  |
[truncated: 146,333 more chars]
